# Recognition of a structural domain (RWDBD) in Gcn1 proteins that interacts with the RWD domain containing proteins

**DOI:** 10.1186/s13062-017-0184-3

**Published:** 2017-05-19

**Authors:** Ramachandran Rakesh, Rangachari Krishnan, Evelyn Sattlegger, Narayanaswamy Srinivasan

**Affiliations:** 1 Molecular Biophysics Unit, Indian Institute of Science, Bangalore, 560012 India; 2 Institute of Natural and Mathematical Sciences (INMS), Massey University, Auckland, 0745 New Zealand

**Keywords:** Remote relationship, Gcn1, Gcn2, RWDBD, Sequence and structure analysis, Protein evolution and conservation

## Abstract

**Abstract:**

The protein Gcn1 (General control non-derepressible 1) is found in virtually all eukaryotes, and is a key component of the general amino acid control signal transduction pathway. This pathway is best known for its importance for cells to sense and overcome amino acid starvation. Gcn1 directly binds to the RWD (RING finger-containing proteins, WD-repeat-containing proteins, and yeast DEAD (DEXD)-like helicases) domain of the protein kinase Gcn2, and this is essential for delivering the starvation signal to Gcn2. Gcn2, and thus the GAAC (General Amino Acid Control) pathway, then becomes activated enabling the cell to cope and overcome the starvation condition. Using sensitive homology detection and fold recognition methods a conserved structural domain in Gcn1, RWD Binding Domain (RWDBD), has been recognized that encompasses the region experimentally shown previously to be involved in Gcn2 binding. Further, the structural fold for this domain has been recognized as the ARM (Armadillo) domain, and residues likely to be involved in the binding of Gcn2 RWD domain have been identified within this structural domain. Thus, the current analysis provides a structural basis of Gcn1-Gcn2 association.

**Reviewers:**

This article was reviewed by Dr Oliviero Carugo and Dr Michael Gromiha.

**Electronic supplementary material:**

The online version of this article (doi:10.1186/s13062-017-0184-3) contains supplementary material, which is available to authorized users.

## Background

In eukaryotic cells phosphorylation of the α-subunit of eIF2, a translation initiation factor, leads to the regulation of protein synthesis in response to stress conditions [1]. In *S. cerevisiae,* it has been shown that amino acid starvation leads to the accumulation of uncharged tRNAs in the cell, which is detected by Gcn2 (General control non-derepressible 2) [1, 2]. This causes the activation of the Gcn2 protein kinase domain and subsequent phosphorylation of eIF2α [1, 3]. For Gcn2 to sense amino acid starvation, direct binding of Gcn1 protein to the RWD domain of Gcn2 is required [4].

In yeast, as found for Gcn2, Gcn1 is a large cytoplasmic protein that is not required for cell growth under normal laboratory conditions but is required for coping with various stress conditions [5]. Gcn1 shows no significant sequence similarity to any other protein except for its middle region (amino acids 1330–1641) which is homologous to the N-terminal HEAT repeat domain of fungal translation elongation factor 3 (eEF3) [6]. Gcn1 contains binding sites for Gcn2, Gcn20 (binds to the Gcn1 eEF3 like region) and the ribosome (binds to the N-terminal 3/4^th^ of the protein) [5]. Gcn1 forms a trimeric complex with Gcn2 and Gcn20. It was the first protein that was found to assist the functionality of Gcn2 in yeast and is absolutely essential for the *in vivo* activation of Gcn2 in response to amino acid starvation [6]. The Gcn1-Gcn2 interaction is mediated by a region in Gcn1 present in amino acids 2048–2383 [7] and 2052–2428 [4]. Both stretches of amino acids were shown to be sufficient for Gcn2 binding *in vitro*, and amino acids 2052–2428 were also shown *in vivo* to be necessary for Gcn2 binding as well as sufficient for disrupting Gcn1-Gcn2 interaction *in vivo* using co-immunoprecipitation assays [4, 7]. This suggests that amino acids 2052–2383 encompasses the Gcn2 binding site, however, the specific domain in Gcn1 that interacts with RWD domain of Gcn2 has not yet been identified. It has been noted that substitution of Arg-2259 of Gcn1 by Ala specifically impairs its binding to the RWD domain of Gcn2 *in vivo* and *in vitro*, but not the binding to other interaction partners such as the ribosome and Gcn20 [4]. The same substitution impairs Gcn2 activation *in vivo*, and this can be rescued by overexpressing Gcn2 in the cell [4]. This strongly suggests that Arg-2259 specifically mediates Gcn1-Gcn2 interaction, and that amino acids in addition to Arg-2259 are also involved in mediating Gcn1-Gcn2 interaction.

In the last decade powerful homology detection methods have been developed to establish structure-function as well as evolutionary relationships utilizing only protein sequence information. These methods are highly sensitive and employ either structure-based or sequence-based profiles to detect evolutionary relationships between proteins which are remotely related [8]. Hence, they are extensively used to shed light on the protein structure as well as function. In the current study, these methods were used to recognize a conserved structural domain in Gcn1 proteins that encompasses the region found experimentally to interact with Gcn2, henceforth called as RWD Binding Domain (RWDBD). RWDBD was recognized to contain ARM structural repeats and adopts the helical repeat fold reminiscent of other ARM repeat proteins. Further, potential interface residues have been identified within this domain that potentially interact with the Gcn2 RWD domain.

### Recognition of ARM domain in the C-terminal region of Gcn1

Yeast Gcn1 (Uniprot ID: P33892) is a large protein consisting of 2672 residues. Therefore, it is likely to have a number of protein domains. In order to recognize the domains, sensitive remote homology detection methods, domain databases and fold recognition approaches were employed. To identify the domains in yeast Gcn1 an initial search was performed in the Pfam version 30.0 database. The Pfam database provides classification of protein families and its domains including their annotations, and multiple sequence alignments [9]. Pfam assigned the DUF3554 domain family to the regions 391–504 (E-value 6.4e^−12^) and 533–670 (E-value 2.1e^−11^) in the Gcn1 sequence, a domain of unknown function. No domain assignment was made by Pfam for the region containing the residue Arg-2259 which is known to interact with the Gcn2 RWD domain [4] as well as the previously identified Gcn1 eEF3 like region [5, 6].

Hence, the SUPERFAMILY resource was employed [10–12] to recognize the structural domains in yeast Gcn1. The SUPERFAMILY database contains a library of HMMs of protein domains of known structure. A search in this HMM database could provide domain assignments based on SCOP for a given protein sequence. For the yeast Gcn1 sequence, the database assigned structural domains to four regions, each of which belonged to the ARM (Armadillo) repeat superfamily – *ARM-1* (1249–2313, E-value: 1.03e^−124^); *ARM-2* (2209–2591, E-value : 1.5e^−25^); *ARM-3* (7–192, 262–438, 473–684, 837–907, 935–946, E-value: 1.73e^−20^); *ARM-4* (957–1299, E-value: 7.09e^−17^). As the *ARM-2* assigned region contained Arg-2259, known to be important for binding to Gcn2, further structural and sequence analysis was performed around this region. The superfamily assignment for this region was further narrowed down to HEAT (Huntingtin, elongation factor 3 (EF3), protein phosphatase 2A (PP2A), and the yeast kinase TOR1) repeat family. This superfamily has structures which form a super helical or solenoid arrangement of approximately 40 residue repeating α-helical structural motifs. The structures in this superfamily have different degrees of curvature ranging from straight rods to curved shapes and in each HEAT repeat containing protein the curvature mostly depends on the molecules that can bind to them [13–15].

Since the HEAT repeat containing proteins are known to have varying number of HEAT repeat motif structural units and based on secondary structure prediction, an additional 50 residues on either side of the above region was included for further analysis. Hence, the Gcn1 region encompassing amino acids 2159–2641 was analysed using HHPred [16] which is a sensitive profile-profile comparison method and identifies significant matches to proteins of known structure by matching their HMMs. HHPred analysis resulted in high confident identification of HEAT repeat containing proteins (E-value: 2.8e^−23^ to 3.4e^−11^ among top 100 hits), though the related proteins of known structure have low sequence identities (9% to 19% among top 100 hits) with the related region in Gcn1.

**Fig. 1:**
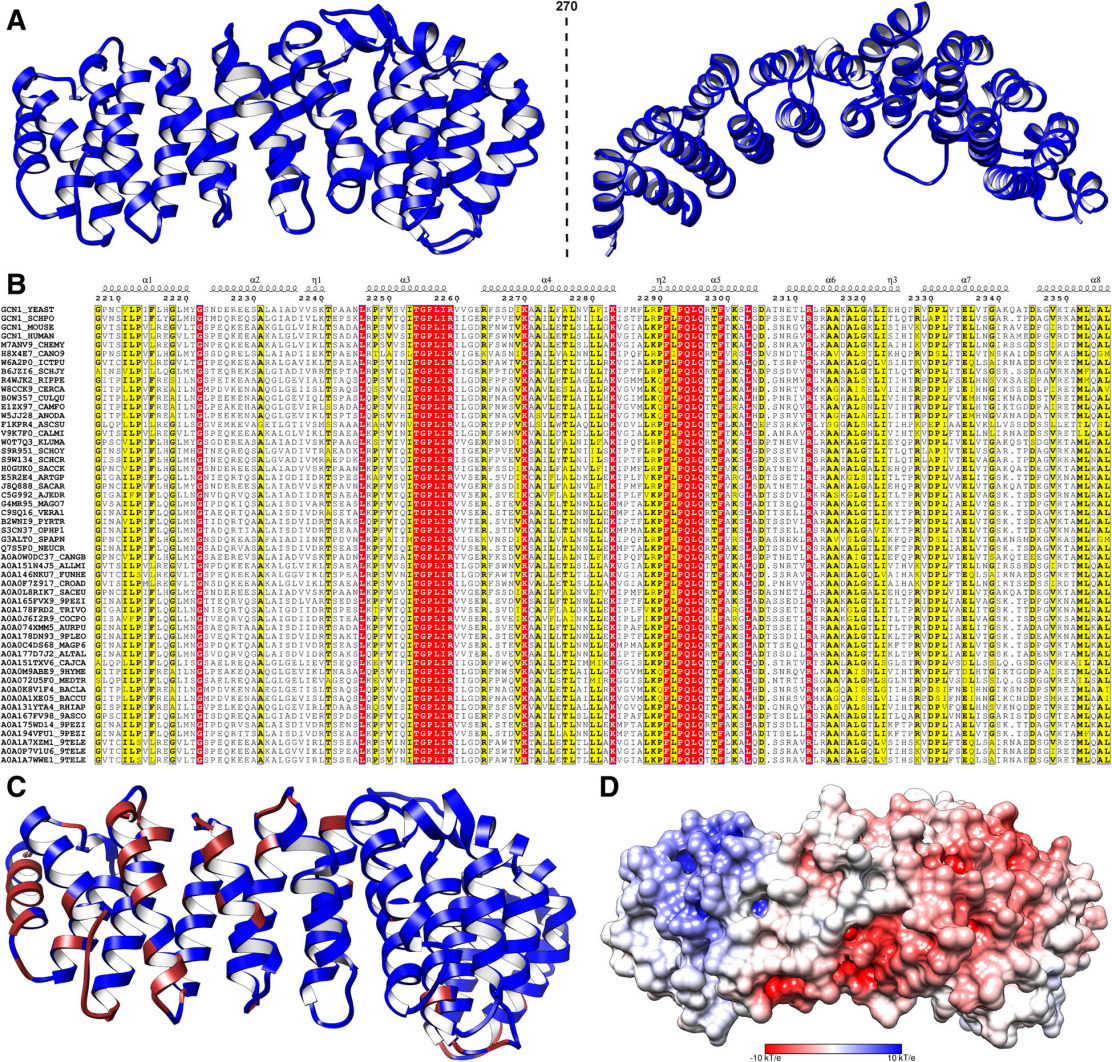
*Structural and sequence features of* RWD Binding Domain (RWDBD)*.* **a** Structural model of yeast Gcn1 shown in two views obtained from fold recognition and homology modeling. **b** Representative alignment of the RWDBD domain family also showing the characteristic sequence motif ITGPLIR which is required for Gcn2 binding, generated using ESPript server (http://espript.ibcp.fr) [40]. The columns shaded dark red show absolute conservation of residues and those shaded yellow show high conservation but contain minor substitution for residues which show highest proportion in the respective columns. The full alignment of this domain region is provided as HMM in Additional file 5. **c** Gcn1 along with the highly conserved and exposed residues are shown in brown shading. Most of the conserved and exposed residues are concentrated around Arg-2259, thus they can be potential protein-protein interface residues. **d** Electrostatic surface potential representation of Gcn1 homology model with the positive surface colored blue; negative surface colored red within the limits ± 10 kT/e. Arg-2259 of Gcn1 is located on the left side of the model and this surface is highly dominated by positive charge followed by a neutral region

As the templates obtained using HHPred were of low sequence identity and in order to gain confidence on the fold assignment, further the same region of Gcn1 was analysed using other fold recognition methods such as LOMETS [17] and Phyre2 [18]. All the independent HMM matching and fold recognition methods employed consistently predicted HEAT repeat containing proteins as related to the 2159–2641 region of Gcn1 (Additional file 1). In particular, all the prediction methods independently pointed at the structure of Kap121p protein, a karyopherin (PDB code: 3W3W in which the structure of Kap121p from *Saccharomyces cerevisiae* is available bound to Ste12p (Sterile 12p), a transcription factor) as one of the top hits (Table 1). Since this protein has HEAT repeats and also belongs to the same superfamily as Gcn1, this template was selected for modeling. Finally, the fact that each of the individual methods gave high confidence predictions, and identified the same set of proteins (with HEAT repeats) as being related, this strongly suggests that the C-terminal ARM domain of Gcn1 is made up of HEAT repeats. Based on the results from different fold prediction servers (Table 1 and Additional file 1) and homology modeling using the manually adjusted HHPred sequence-structure alignment (Additional file 2), a high quality model (ProSA Z-score of−8.29) was obtained for the region 2207–2602 of Gcn1 (Fig. 1a) which also exhibited the characteristic curvature typically seen in HEAT repeat containing proteins.

**Table 1:** Fold recognition for the C-terminal domain of Gcn1

Fold recognition methods	Confidence scores for ARM domain
*Method*	*Confidence measure in %*
HHPred	99.9
Phyre2	99.9
*Methods in LOMETS Meta-server*	*Confidence measure in Z-score*
SPARKS-X	9.3
FFAS-3D	82.6
HHSEARCH2	20.1
HHSEARCH I	18.4
Neff-PPAS	13.7
wdPPAS	9.0
HHSEARCH	18.4
SP3	18.4
FFAS03	33.8
cdPPAS	8.8
pGenTHREADER	22.3
PRC	94.6

### RWD Binding Domain (RWDBD) in Gcn1 proteins

Direct binding of Gcn1 to Gcn2 RWD domain, and Arg-2259 in yeast Gcn1, are important for the activation of Gcn2 under amino acid starvation conditions [4]. Sequence alignment of the region immediately surrounding Arg-2259, and experimental studies strongly suggest that the same is true for virtually the entire eukaryotic kingdom, implying that Gcn1-Gcn2 interaction is highly conserved [5]. Hence, identification of Gcn1 proteins in other eukaryotic organisms was pursued, and after retaining only a non-redundant set of 52 sequences including yeast Gcn1 were obtained (Additional file 3). Further, multiple sequence alignment (MSA) was performed to align all the sequences to obtain their corresponding matching regions to yeast Gcn1 sequence (Additional file 4). Gcn1 Arg-2259 resides within the conserved sequence motif ITGPLIR [4]. From the structural fold assigned to yeast Gcn1 and MSA of the Gcn1 proteins in various organisms which contains the characteristic TGPLIR motif (Fig. 1b), with TGPLIR being absolutely conserved, a conserved domain in the Gcn1 proteins encompassing the Gcn2 interacting region was identified, henceforth called as RWD Binding Domain (RWDBD).

As RWDBD contains Arg-2259 which is known to be crucial for binding to Gcn2 from mutational studies [4], the potential binding residues within this domain were identified that may interact with Gcn2, using conservation as well as solvent accessibility criteria, and mapped on to the yeast Gcn1 model (Fig. 1c). Mutation of Arg-2259 with Ala in Gcn1 has been shown to impair the Gcn1-Gcn2 interaction, but its interaction with ribosome and Gcn20 is unaffected [4]. Arg-2259 is solvent exposed in the modeled structure, hence the Arg to Ala mutation is unlikely to alter the overall structure of Gcn1. Further, the charge distribution was calculated around the protein surface based on the yeast Gcn1 model (Fig. 1d). It was found that the surface around Arg-2259 is highly positively charged, followed by a surrounding neutral region. Also, there are highly conserved solvent exposed residues which are spatially proximal to Arg-2259. In addition, earlier studies have proposed the involvement of negatively charged residues in the RWD domain of Gcn2 in binding to Gcn1 [19, 20]. Hence, the highly conserved, solvent exposed and positively charged residues including Arg-2259 of Gcn1 RWDBD might help in strengthening the interaction with the negatively charged residues in Gcn2 RWD domain.

Also, the structural model and conservation information further facilitated the interpretation of previously reported mutations proposed to affect the Gcn1-Gcn2 interaction. Out of the reported mutations [21], F2291L and L2353P might destabilize the helix-helix interactions within their corresponding HEAT repeats as they are buried in the structure and show high degree of conservation (Fig. 1b), implying their functional importance in maintaining the correct overall 3D structure of the RWDBD. The other reported mutation S2304P [21] is a surface residue at the terminus of a loop between two HEAT repeats and it shows high degree of conservation as well, thus this mutation might restrict Gcn2 binding either by reducing the flexibility of the loop or disrupting a potential interaction with Gcn2. Apart from this other experiments have suggested that substituting the E2263 and R2264, respectively, does not affect Gcn1-Gcn2 interaction [4]. From the analysis, these residues are surface exposed and show high conservation (Figs. 1b and c) with proximity to Arg-2259. Though these residues are proximal to Arg-2259, it is most likely that these residues do not contribute to the interacting surface of Gcn1 to interact with RWD domain of Gcn2, thus are not affected by the mutations. Alternatively, these amino acids are only minor contributors to the Gcn1-Gcn2 interaction, and the experimental methods employed in this study were not sensitive enough to detect such weak contributions.

Taken together, we predicted the structure of Gcn1 region 2207–2602 with high confidence, that encompasses the Gcn2-binding site, and the structural as well as conservation analysis identified amino acids potentially interacting with Gcn2, supporting the idea that this region can serve as the interaction module for Gcn2 RWD domain. Future experimental studies targeting this domain will help validate the predicted charged amino acids for their contribution to Gcn1-Gcn2 interaction, determine the N- and C-terminal border of the RWD-BD, and further reveal the functionality of this domain with regards to Gcn2 binding.

## Methods

### Multiple sequence alignment (MSA) generation for query – template alignment

First, homologues of proteins involved in this analysis have been identified using HHblits [22] for the Gcn1 region 2159–2641 employing 3 iterations with an E-value cut-off of 0.0001, coverage cut-off of 70% and probability cut-off of 90% in the local alignment mode, along with MAC (maximum accuracy) realignment threshold of 0.5. Moreover during searches for homologues using HHblits, only sequences with identities in the range 30% to 90% to the query were preserved in the HHblits alignment. Similarly, to avoid redundancies, the data set was scrutinized so that the identity between any two homologous sequences is not more than 90%.

Further the alignment obtained from HHblits was examined with the help of UGENE software [23] to exclude putative, predicted and uncharacterized sequences even if they satisfied the above criteria. This was done to ensure only well characterized sequences are present in the multiple sequence alignment and to perform minor alignment corrections. This is important since there is a chance that unrelated sequences may be included in the alignment inadvertently.

### Structural modeling of yeast Gcn1 sequence

An initial sequence-structure alignment was obtained by aligning the multiple sequence alignment of Gcn1 (previous step) to its template, a Kap121p protein, a karyopherin (PDB ID: 3W3W) using HHpred [16]. Finally this alignment was manually adjusted using JOY [24] and used to generate 3-D models using MODELLER 9v12 [25] for the Gcn1 region 2207–2602. Manual adjustments in the alignment were made to disallow secondary structure breaks and, as far as possible, the buried residues in the template were aligned with the hydrophobic residues in the target. Ten models were generated and the best model was selected based on the Modeller’s normalized DOPE score. The model quality was verified using ProSA-Web [26, 27] and energy minimized with GROMACS [28] in CHARMM27 force field [29] using steepest descent minimization method.

### Identification of Gcn1 proteins and the RWDBD region in other eukaryotes

To identify Gcn1 proteins in other eukaryotic organisms, the yeast Gcn1 sequence (Uniprot ID: P33892) was searched against UniprotKB Eukaryota database [30] which contains more than 22 million sequences using PSI-Search [31, 32] at an E-value threshold of 0.000001, sequence identity cut-off of 25%, three iterations and query coverage cut-off of 75%. At each iteration putative, predicted and uncharacterized sequences were excluded even if they satisfied the above criteria. The sequence hits containing uncharacteristic symbols or ambiguities were removed and rest of the sequences were clustered at 95% sequence identity and 75% sequence length coverage using BLASTCLUST [33] to obtain 97 non-redundant representative Gcn1 sequences.

As this sequence set contains both reviewed sequences from Swiss-Prot and unreviewed sequences from TrEMBL, first multiple sequence alignment was performed using only the five Swiss-Prot sequences with PROMALS3D (PROfile Multiple Alignment with predicted Local Structures and three-dimensional constraints) [34] to obtain an initial alignment. Subsequently all the 97 sequences were aligned using PROMALS3D with initial Swiss-Prot based MSA used as constraint. The MSA obtained was manually checked using UGENE and sequences comprising large insertions in the alignment were removed in order to maximize the contiguous blocks in the alignment. Thus a final set of 53 sequences were obtained which were further aligned using PROMALS3D with the same initial Swiss-Prot based MSA used as constraint. Finally from the yeast Gcn1 structural model and MSA, the RWD Binding Domain (RWDBD) was identified; the HMM of this domain region is provided in the Additional file 5.

### Solvent accessibility calculation on the yeast Gcn1 model

The solvent accessibility calculation was performed on the Gcn1 structural model using the NACCESS program to identify the buried and solvent exposed residues. The solvent exposed residues were identified based on the criteria of ≥10% residue solvent accessibility and buried residues were identified based on the accessibility criteria ≤7%.

### Identification of potential protein-protein interface residues

The conserved residues in a protein were identified using the Consurf server [35, 36]. This method uses a multiple sequence alignment of homologous proteins and calculates the conservation for each residue using an empirical Bayesian method weighted using the phylogenetic distance between sequences. The MSA of the RWDBD region obtained in the previous step was analysed using Consurf server and the normalized conservation scores were obtained for each residue. Finally, the conservation scores of residues (Consurf color scale >8) were mapped on the surface exposed residues. This was used to identify conserved and solvent exposed residues in the ARM domain model of yeast Gcn1 and considered to be potential protein interface residues. Necessary programs in this protocol were developed in python language.

### Electrostatic potential surface representation and molecular visualization

The electrostatic potential surface for the yeast Gcn1 model was calculated using the Adaptive Poisson-Boltzmann Solver [37, 38] and visualized using Chimera [39]. The modeled structure was prepared and visualized using Chimera.

## Reviewer’s comments

### Reviewer’s report 1: Oliviero Carugo

Reviewer comments: The manuscript submitted by E. Sattlegger and N. Srinivasan describes the discovery and the 3D modelling of the RWD binding domain of Gcn1. Technically sound, it needs, in my opinion, only minor modifications.

Comment 1. The biological source of the PDB entry 3w3w should be indicated (line 117).

Response*: We thank Dr. Oliviero Carugo for his valuable comments. As suggested, the biological source has now been mentioned.*

Comment 2. The protein Ste12p should be called with its name (line 117).

Response*: The name of this protein itself is Ste12p (Uniprot ID: P13574) where Ste stands for Sterile. It is a transcription factor which is now mentioned in the manuscript.*

Comment 3. The results of the fold recognitions (just listed in Table 1) should be summarized in the supplementary material. This would explain why the Authors selected Kap121p as a template (lines 196–197).

Response*: The fold recognition results are now summarized in the Additional File* *1* *which is a newly included Additional file. Kap121p is a hit with high reliability measures in many of the fold recognition methods used.*

Comment 4. Which manual adjustments were done with JOY (line 196)?

Response: *The manual adjustments were performed to disallow secondary structure breaks in the target-template alignment and also, as far as possible, the hydrophobic residues in the target are aligned with buried residues in the template. We have included these points in the revised version of the manuscript.*

Comment 5. Might the Authors use also other structure validation tools beside ProSA (line 199).

Response*: As our objective is to ensure that the overall fold of the model is correct, any reasonable structure validation program would do. Therefore we have used only ProSA to validate the modeled structure.*

Comment 6. Which energy minimization protocol was followed (line 200)?

Response: *The minimization was performed using steepest descent method.*

Comment 7. Does the use of two thresholds (7% and 10%) imply that some residues are not classified (line 226–228)? And also, why do the Authors selected these threshold values?

Response*: The thresholds used here are stringent ones where ≥10% yields residues which are on the surface of the protein and are well exposed. The threshold ≤7% yields residues which are highly buried and are often in the core of the protein. The residues having solvent accessibility between these two thresholds are classified as partially buried. Since the analysis required identification of potentially interacting residues and interpretation of mutational data, these threshold values were used. These thresholds have been well-established by T.J.P. Hubbard and T.L. Blundell in “Comparison of solvent-inaccessible cores of homologous proteins: definitions useful for protein modelling” (1987) Protein Engng, 1, 159–171.*

Comment 8. Is the phylogenetic distribution sufficiently wide to allow a reliable detection of conservation with Consurf (line 230)?

Response*: Consurf uses Bayesian inference methods and appropriate evolutionary models to calculate the* conservation scores, *thus the score calculation negates any phylogenetic distribution bias. Moreover, sources of the proteins used in this conservation analysis range from Fungi to Metazoans (Additional file* *3* *). The pairwise sequence identity among the sequences in the multiple sequence alignment for the RWDBD region ranges from 23% to 96%. Therefore we believe that the phylogenetic distribution is sufficiently wide to detect conserved regions of structural or functional importance.*

Comment 9. In Fig. 1c, it would be better to use not only the yellow but also the red, according to Fig. 1b.

Response*: The coloring of residues as yellow in Fig.* *1* *c is based on both Consurf color scale ≥8 for highly conserved residues and solvent accessibility ≥10% for highly exposed surface residues. Moreover, the criteria for coloring in Fig.* *1* *b and c is different as mentioned in the legend to Fig.* *1* *. Hence, in order to avoid comparison between the panels Figs.* *1* *b and c, the highly conserved and exposed surface residues are now colored brown in Figs.* *1* *c.*

Comment 10. What is the yellow region at the bottom-right of Fig. 1c.

Response*: This region comprises of highly conserved and exposed surface residues whose color has now been changed to brown.*

Comment 11. I note in Fig. 1d that the positively charged region (top left) is surrounded by a white, neutral crown. This might be relevant, since it confers a low dielectric constant that might reinforce electrostatic interactions of the positively charged region.

Response*: We thank the reviewer for this observation. The residues forming the patch with low dielectric constant are generally well conserved although they are solvent exposed. Further, these residues are spatially proximal to Arg 2259 which was previously suggested, by mutational experiments, to bind to Gcn2. Therefore, we believe that this region proximal to Arg2259 in Gcn1 is likely to participate in binding to Gcn2. This point is now discussed in the manuscript.*

### Reviewer’s report 2: Michael Gromiha

Reviewer comments: In this work, the authors provided a structural basis of Gcn1-Gcn2 association using homology detection and fold recognition methods. They have identified the interacting domain and the residues involved in the binding of Gcn2 RWD domain. The work is interesting and the analysis deepens our knowledge for understanding the general amino acid control signal transduction pathway in Gcn1. The following suggestions may be carried out for improvements.

Comment 1. It has been stated that no domain assignment was made by Pfam for the region containing Arg-2259. The agreement using other well performing methods could be presented.

Response*: We thank Dr. Michael Gromiha for his comments and suggestions. The search for domains in Gcn1 using other sequence based databases such as InterPro database v.62, the Conserved domain database (CDD) and SMART yielded similar results as Pfam with no domains assigned to the region containing Arg-2259.*

Comment 2. Mutational analysis of Arg2259 could be reported on the basis of structure, folding and function.

Response*: The implications of Arg2259 mutation from structural and functional point of view is now discussed in the manuscript.*

Comment 3. Several abbreviations are used in the abstract, which requires explanations.

Response*: The expansions to the various abbreviations are now been mentioned in the abstract and towards the end of the manuscript.*

## Additional files

Additional file 1: Summary of the results obtained from different fold recognition methods. (DOCX 47 kb)

Additional file 2: Sequence-structural alignment between yeast Gcn1 sequence and the template PDB ID 3W3W Chain A represented using JOY. (PDF 2278 kb)

Additional file 3: Description of the 52 eukaryotic Gcn1 sequences used in the study. (XLSX 12 kb)

Additional file 4: Multiple sequence alignment of the 53 eukaryotic Gcn1 sequences generated using PROMALS3D and exported in ClustalW format. (PDF 370 kb)

Additional file 5: Hidden Markov model of the RWD Binding Domain (RWDBD) region. (PDF 65 kb)

## Abbreviations

CHARMM: Chemistry at Harvard molecular mechanics
DUF: Domains of unknown function
Gcn1: General control non-depressible 1
GROMACS: Groningen machine for chemical simulations
HEAT: Huntingtin, elongation factor 3 (EF3), protein phosphatase 2A (PP2A), and the yeast kinase TOR1
HMM: Hidden Markov model
MSA: Multiple sequence alignment
RWD: Domain found in RING finger-containing proteins, WD-repeat-containing proteins, and yeast DEAD (DEXD)-like helicases, ARM, Armadillo repeat
