# Supplementary material for: Recognition of a structural domain (RWDBD) in Gcn1 proteins that interacts with the RWD domain containing proteins
Source: Biol Direct. 2017 May 19;12:12. doi: 10.1186/s13062-017-0184-3 (PMC5438488; doi:10.1186/s13062-017-0184-3)
Supplement: Supplementary file 1 — Summary of the results obtained from different fold recognition methods. (DOCX 47 kb) [file 13062_2017_184_MOESM1_ESM.docx]

Additional file 1: Summary of the results obtained from different fold recognition methods

**1) HHPred**

| Rank | Template | Aligned length | Confidence measure (%) | Sequence identity (%) |
| --- | --- | --- | --- | --- |
| 1 | 1b3u_A | 426 | 100.0 | 15 |
| 2 | 1b3u_A | 427 | 99.9 | 13 |
| 3 | **3w3w_A** | **439** | **99.9** | **11** |
| 4 | 4uqi_B | 420 | 99.9 | 11 |
| 5 | 2bpt_A | 423 | 99.9 | 13 |
| 6 | 4rxh_B | 405 | 99.9 | 11 |
| 7 | 1qgr_A | 418 | 99.9 | 10 |
| 8 | 2jdq_A | 395 | 99.9 | 11 |
| 9 | 4uad_A | 397 | 99.9 | 12 |
| 10 | 4uae_A | 396 | 99.9 | 10 |

| **2) Phyre2** |
| --- |
| \| Rank \| Template \| Aligned length \| Confidence measure (%) \| Sequence identity (%) \| \| --- \| --- \| --- \| --- \| --- \| \| 1 \| **3w3z_A** \| **434** \| **99.9** \| **13** \| \| 2 \| 1u6g_C \| 450 \| 99.9 \| 15 \| \| 3 \| 4a0c_B \| 444 \| 99.9 \| 14 \| \| 4 \| 2qna_A \| 429 \| 99.8 \| 14 \| \| 5 \| 1b3u_A \| 428 \| 99.8 \| 16 \| \| 6 \| 1gw5_B \| 452 \| 99.8 \| 11 \| \| 7 \| 1m5n_S \| 407 \| 99.8 \| 14 \| \| 8 \| 2ot8_B \| 405 \| 99.7 \| 12 \| \| 9 \| 1qbk_B \| 398 \| 99.7 \| 11 \| \| 10 \| 4rv1_F \| 372 \| 99.7 \| 13 \| |

**3) LOMETS Meta-server**

**a) SPARKS-X**

| Rank | Template | Aligned length | Confidence measure (Z-score) | Sequence identity (%) |
| --- | --- | --- | --- | --- |
| 1 | 1u6g_C | 474 | 10.430 | 15 |
| 2 | 3fga_A | 462 | 10.420 | 13 |
| 3 | 2bpt_A | 462 | 10.380 | 11 |
| 4 | 3nd2_A | 462 | 10.200 | 11 |
| 5 | 2npp_A | 459 | 10.140 | 13 |
| 6 | 3k7v_A | 461 | 9.730 | 11 |
| 7 | 2qna_A | 479 | 9.420 | 10 |
| 8 | 1ee4_A | 402 | 9.380 | 11 |
| 9 | **3w3t_A** | **476** | **9.310** | **12** |
| 10 | 3ea5_B | 479 | 9.290 | 10 |

**b) FFAS-3D**

| Rank | Template | Aligned length | Confidence measure (Z-score) | Sequence identity (%) |
| --- | --- | --- | --- | --- |
| 1 | 3nd2_A | 454 | 91.400 | 10 |
| 2 | 1ukl_A | 473 | 88.800 | 12 |
| 3 | 2iae_A | 458 | 87.400 | 13 |
| 4 | 2bku_B | 467 | 84.300 | 10 |
| 5 | **3w3z_A** | **467** | **82.600** | **13** |
| 6 | 1qbk_B | 438 | 80.651 | 14 |
| 7 | 4b18_A | 402 | 80.400 | 11 |
| 8 | 4a0c_A | 462 | 79.924 | 15 |
| 9 | 4uae_A | 389 | 79.700 | 11 |
| 10 | 3now_A | 439 | 78.800 | 11 |

**c) HHSEARCH2**

| Rank | Template | Aligned length | Confidence measure (Z-score) | Sequence identity (%) |
| --- | --- | --- | --- | --- |
| 1 | **3w3w_A** | **444** | **20.130** | **14** |
| 2 | 2bpt_A | 446 | 19.189 | 12 |
| 3 | 4fdd_A | 451 | 19.179 | 12 |
| 4 | 1qgr_A | 453 | 18.338 | 12 |
| 5 | 1qgr_A | 445 | 18.328 | 13 |
| 6 | 1b3u_A | 426 | 17.933 | 12 |
| 7 | 4fdd_A | 447 | 17.380 | 13 |
| 8 | 2bpt_A | 449 | 16.958 | 11 |
| 9 | 1u6g_C | 456 | 16.948 | 12 |
| 10 | 1b3u_A | 454 | 16.938 | 13 |

**d) MUSTER**

| Rank | Template | Aligned length | Confidence measure (Z-score) | Sequence identity (%) |
| --- | --- | --- | --- | --- |
| 1 | 1b3u_A | 452 | 7.570 | 0.14 |
| 2 | 2yns_A | 399 | 7.559 | 0.12 |
| 3 | 4uae_A | 398 | 7.549 | 0.09 |
| 4 | 4rxh_B | 399 | 7.454 | 0.12 |
| 5 | 1ial_A | 416 | 7.431 | 0.10 |
| 6 | 2jdq_B | 402 | 7.259 | 0.11 |
| 7 | 1wa5_B | 429 | 7.183 | 0.11 |
| 8 | 4rv1_A | 393 | 7.008 | 0.15 |
| 9 | 2z6g_A | 471 | 6.674 | 0.11 |
| 10 | 4r0z_A | 457 | 6.570 | 0.13 |

**e) HHSEARCH I**

| Rank | Template | Aligned length | Confidence measure (Z-score) | Sequence identity (%) |
| --- | --- | --- | --- | --- |
| 1 | 2bpt_A | 480 | 19.856 | 11 |
| 2 | 1qgr_A | 478 | 19.743 | 11 |
| 3 | 1qgr_A | 463 | 19.330 | 11 |
| 4 | 2bpt_A | 479 | 19.320 | 12 |
| 5 | 1b3u_A | 452 | 19.310 | 13 |
| 6 | **3w3w_A** | **465** | **18.383** | **12** |
| 7 | **3w3w_A** | **463** | **18.148** | **12** |
| 8 | 1u6g_C | 471 | 18.138 | 13 |
| 9 | 4fdd_A | 467 | 18.128 | 12 |
| 10 | 1b3u_A | 463 | 17.895 | 13 |

**f) Neff-PPAS**

| Rank | Template | Aligned length | Confidence measure (Z-score) | Sequence identity (%) |
| --- | --- | --- | --- | --- |
| 1 | 1b3u_A | 450 | 17.287 | 14 |
| 2 | 4rxh_B | 410 | 15.122 | 12 |
| 3 | 4uae_A | 401 | 14.992 | 9 |
| 4 | 2yns_A | 402 | 14.478 | 12 |
| 5 | 1ial_A | 406 | 13.810 | 10 |
| 6 | **3w3t_A** | **429** | **13.696** | **11** |
| 7 | 2jdq_B | 401 | 13.504 | 12 |
| 8 | 2z6g_A | 453 | 13.407 | 11 |
| 9 | 1wa5_B | 413 | 13.176 | 9 |
| 10 | 4rv1_A | 383 | 12.515 | 15 |

**g) wdPPAS**

| Rank | Template | Aligned length | Confidence measure (Z-score) | Sequence identity (%) |
| --- | --- | --- | --- | --- |
| 1 | 1b3u_A | 448 | 10.884 | 13 |
| 2 | 2jdq_B | 403 | 10.248 | 12 |
| 3 | 4uae_A | 393 | 10.203 | 9 |
| 4 | 4rxh_B | 397 | 10.089 | 12 |
| 5 | 2yns_A | 394 | 10.089 | 12 |
| 6 | 1ial_A | 408 | 9.930 | 10 |
| 7 | 1qbk_B | 407 | 9.900 | 13 |
| 8 | 1wa5_B | 425 | 9.779 | 09 |
| 9 | 4rv1_A | 392 | 9.665 | 15 |
| 10 | **3w3t_A** | **380** | **9.021** | **10** |

**h) HHSEARCH**

| Rank | Template | Aligned length | Confidence measure (Z-score) | Sequence identity (%) |
| --- | --- | --- | --- | --- |
| 1 | 1qgr_A | 477 | 19.282 | 12 |
| 2 | 2bpt_A | 479 | 18.944 | 11 |
| 3 | 2bpt_A | 467 | 18.676 | 11 |
| 4 | **3w3w_A** | **461** | **18.419** | **12** |
| 5 | 1qgr_A | 456 | 18.409 | 13 |
| 6 | 1b3u_A | 462 | 18.399 | 13 |
| 7 | 1u6g_C | 470 | 18.389 | 14 |
| 8 | 4fdd_A | 475 | 18.379 | 11 |
| 9 | 4fdd_A | 455 | 17.170 | 11 |
| 10 | 4uqi_B | 451 | 17.160 | 11 |

**i) SP3**

| Rank | Template | Aligned length | Confidence measure (Z-score) | Sequence identity (%) |
| --- | --- | --- | --- | --- |
| 1 | 1b3u_A | 452 | 20.453 | 14 |
| 2 | 1u6g_A | 477 | 20.329 | 14 |
| 3 | 4rv1_A | 396 | 19.177 | 16 |
| 4 | **3w3t_A** | **465** | **18.420** | **15** |
| 5 | 2bku_B | 479 | 18.393 | 11 |
| 6 | 1qgr_A | 481 | 17.983 | 11 |
| 7 | 4mz5_E | 408 | 15.787 | 11 |
| 8 | 4fdr_B | 408 | 15.632 | 11 |
| 9 | 1gw5_B | 452 | 15.365 | 10 |
| 10 | 3oqs_A | 406 | 14.937 | 11 |

**j) FFAS03**

| Rank | Template | Aligned length | Confidence measure (Z-score) | Sequence identity (%) |
| --- | --- | --- | --- | --- |
| 1 | 2iae_A | 453 | 40.000 | 12 |
| 2 | 2qna_A | 466 | 38.200 | 11 |
| 3 | 2h4m_A | 460 | 37.900 | 11 |
| 4 | 1qgk_A | 467 | 37.800 | 10 |
| 5 | 1qbk_B | 455 | 37.200 | 11 |
| 6 | 1ukl_A | 467 | 36.700 | 10 |
| 7 | 1u6g_C | 476 | 36.600 | 15 |
| 8 | 2bku_B | 481 | 34.700 | 11 |
| 9 | **3w3t_A** | **460** | **33.800** | **11** |
| 10 | **3w3t_A** | **371** | **30.600** | **11** |

**k) cdPPAS**

| Rank | Template | Aligned length | Confidence measure (Z-score) | Sequence identity (%) |
| --- | --- | --- | --- | --- |
| 1 | 1u6g_C | 477 | 9.947 | 15 |
| 2 | 1qgr_A | 478 | 9.089 | 11 |
| 3 | 2bku_B | 478 | 9.071 | 10 |
| 4 | 1b3u_A | 452 | 8.983 | 14 |
| 5 | **3w3t_A** | **473** | **8.817** | **15** |
| 6 | 4rv1_A | 391 | 8.760 | 17 |
| 7 | 4htv_A | 405 | 7.628 | 11 |
| 8 | 3rz9_A | 403 | 7.619 | 11 |
| 9 | 3uvu_A | 406 | 7.561 | 11 |
| 10 | 3oqs_A | 403 | 7.435 | 11 |

**l) pGenTHREADER**

| Rank | Template | Aligned length | Confidence measure (Z-score) | Sequence identity (%) |
| --- | --- | --- | --- | --- |
| 1 | 1b3u_A | 453 | 23.942 | 14 |
| 2 | 4fdd_A | 455 | 22.333 | 15 |
| 3 | **3w3w_A** | **450** | **22.333** | **13** |
| 4 | 2bpt_A | 443 | 21.080 | 12 |
| 5 | 1u6g_C | 397 | 0.384 | 13 |
| 6 | 1u6g_C | 50 | 0.384 | 20 |
| 7 | 4tnm_A | 360 | 19.625 | 13 |
| 8 | 4mz6_E | 362 | 19.625 | 12 |
| 9 | 4b8j_A | 371 | 19.625 | 12 |
| 10 | 1wa5_B | 365 | 18.932 | 12 |

**m) PROSPECT2**

| Rank | Template | Aligned length | Confidence measure (Z-score) | Sequence identity (%) |
| --- | --- | --- | --- | --- |
| 1 | 1b3u_A | 466 | 8.733 | 13 |
| 2 | 1gw5_B | 446 | 7.871 | 11 |
| 3 | 1jdh_A | 448 | 7.718 | 12 |
| 4 | 1ejl_I | 409 | 7.628 | 11 |
| 5 | 1ee4_A | 406 | 7.617 | 10 |
| 6 | 1qgr_A | 481 | 7.616 | 11 |
| 7 | 2bku_B | 478 | 7.396 | 14 |
| 8 | 1w63_A | 462 | 7.264 | 11 |
| 9 | 1u6g_C | 482 | 7.250 | 13 |
| 10 | 1gw5_A | 440 | 7.090 | 12 |

**l) PRC**

| Rank | Template | Aligned length | Confidence measure (Z-score) | Sequence identity (%) |
| --- | --- | --- | --- | --- |
| 1 | 1b3u_A | 444 | 110.700 | 12 |
| 2 | 1u6g_C | 459 | 108.600 | 13 |
| 3 | 3ea5_B | 466 | 97.000 | 10 |
| 4 | **3w3t_A** | **420** | **94.600** | **15** |
| 5 | 1qbk_B | 385 | 94.000 | 15 |
| 6 | 1qgk_A | 458 | 89.700 | 11 |
| 7 | 1qbk_B | 443 | 89.600 | 9 |
| 8 | 2iwh_B | 300 | 70.200 | 12 |
| 9 | 2ix8_A | 235 | 67.700 | 14 |
| 10 | 4rv1_A | 385 | 66.900 | 11 |

PDB Chains 3w3w_A, 3w3t_A and 3w3z_A correspond to the same Kap121p protein from *Saccharomyces cerevisiae*. These templates were ranked among top 10 hits in all the fold recognition methods except for MUSTER and PROSPECT2 in the LOMETS Meta-server.
