# Supplementary material for: Recognition of a structural domain (RWDBD) in Gcn1 proteins that interacts with the RWD domain containing proteins
Source: Biol Direct. 2017 May 19;12:12. doi: 10.1186/s13062-017-0184-3 (PMC5438488; doi:10.1186/s13062-017-0184-3)
Supplement: Supplementary file 4 — Multiple sequence alignment of the 53 eukaryotic Gcn1 sequences generated using PROMALS3D and exported in ClustalW format. (PDF 370 kb) [file 13062_2017_184_MOESM4_ESM.pdf]

Additional file 4: Multiple sequence alignment of the 53 eukaryotic Gcn1 sequences generated using PROMALS3D and exported in ClustalW format

```
CLUSTAL W 2.0 multiple sequence alignment

GCN1_YEAST          M---TAILNWEDI--------------------SPVLEKGTRESHVSKRVPFLQD 55
GCN1_SCHPO          MSVEEPGIEAHGH--------------KDRMLYAMLLSKDTSLAFLGSKKIMIDI
GCN1_DICDI          MNEENPSSSIILDDQ---------QQQDSFYSEIKELEKSIQSNKLSERKNTLTR
GCN1_MOUSE          MAA-----------------------DTQVSETLKRFAVKVTTASVKERREILSE
GCN1_HUMAN          MAA-----------------------DTQVSETLKRFAGKVTTASVKERREILSE
M7ANV9_CHEMY        M------------------------------------------------------
H8X4E7_CANO9        M---SEIVEWEQL--------------------RPVYEKGVKSSSLKDRLSTLKS
W6A2P0_ICTPU        MP-----------------------------------------------------
B6JZI6_SCHJY        MQAEDSTESVTSG-------------AESLRLSNVVKTREMLLSFTGSSEKILNY
R4WJK2_RIPPE        MAS-----------------------NEELTKVLKDLPLKIQTSRKSERKLLFEK
W8CCK9_CERCA        MA------------------------NVELSTALRDLPNRVLSVSYDERTNLFYN
B0W357_CULQU        MA------------------------DAELAKALKDLPNRVLNVPVEERPELFRN
E1ZX97_CAMFO        MA------------------------DVELAKALKDLPNRVQTASKKERREILQN
W5JJ28_ANODA        MA------------------------DAELAKALKDLPNRVLNVPVEERAELFQN
F1KPR4_ASCSU        MSGMEAIVDGTVNDSEDN--KKSDEQQQQFRDALRKFAEHFNESSVRQQKINTSL
V9K7F0_CALMI        MVT-----------------------GSQMSDTLKRFAAKVTTSSVKERWQIMSE
W0T7Q3_KLUMA        MS------KWEEI--------------------EPTLYRDCHNSLLKVRLPALTN
S9R951_SCHOY        MTVEGPGMEAFQN--------------EKKTLYSTLISNETVVAFLGTKKVMLDI
S9W134_SCHCR        MAVEGPGIEAFQN--------------EKKTLYSTLISSETILAFLGTKKVMLDI
H0GUK0_SACCK        M---TAVLNWEEI--------------------SPVLEKGARESLVSKRVPFLQS
E5R2E4_ARTGP        M----DEEQWDEV----------------RKGSLIPLEPVVFSSSTSLRCQLLHQ
J8Q888_SACAR        M---TAILNWEDI--------------------FPVLEKGTRESLVSQRVPFLQD
C5G992_AJEDR        M----DEIEWNNV----------------SEGSLESLERVVLSGSTARRCHGLHE
G4MR95_MAGO7        M----TADVVNGD--------------GPGQVDLVAIKQVLLSSSTKTRISQLRI
C9SQ16_VERA1        MNEVAAEPVANGA------------DSGSANVDFVALKGALTSSSTRVRVDQLRA
B2WNI9_PYRTR        MAA-----NGDAE---------------SQAISRDGLRQKLITSSTKRRITELSA
S3CN37_OPHP1        M----TDSTSTSG-------------SGAAMLDPVATRVALSTSSTSSRIAQLRT
G3ALT0_SPAPN        MTGSTNIESWDQL--------------------EPIFEKGIQDSLTTKRIVILKA
Q7S5P0_NEUCR        M----AEDAAASPAPA------STPSNAPADFDLVAAKAALTSSSTSARISQLRS
A0A165FVX9_9PEZI    M-------EGNDS-------------GGAPNWESNSLPLRLLSSSTNARKAELHT
A0A178DN93_9PLEO    MAV-----NGDAN---------------SPAIDRAELRQKLITSSTKRRTTELAG
A0A177D7J2_ALTAL    M----AAVNGDAE---------------SRAISRDELRPKLITSSTKRRVNELSG
A0A0C4DS68_MAGP6    M----SEDAVNGE--------------AAGQIDLAATKQALLSSSTSARTAQLRL
A0A151N4J5_ALLMI    MAA-----------------------DTQVSDTLKRFAVKVTTASVKERREILNE
A0A146NKU7_FUNHE    MAA-----------------------DTQVSDTLKKFAVKVTTASIKERKEIYGD
A0A0F7Z917_CROAD    MAA-----------------------DTQVSETLKRFAVKVTTSSVKERREILSE
A0A0W0DC37_CANGB    M---SNPRSWEVL--------------------EPELGKLCYDAKISNRIPILTE
A0A0L8RIK7_SACEU    M---TATLNWADI--------------------SPVLEKGTRESLVSKRVPFLQD
A0A178FRD2_TRIVO    M----DEEQWDEV----------------KKGSLTPLEPVVFSSSTSRRCQLLHQ
A0A175WD14_9PEZI    M----TEAAANPT---------------PGNLDLEQAKAALTSSSTAARIAQLRA
A0A194VFU1_9PEZI    M----TEVAVNGA---------------APALDLKATKAALTSSSTSSRIAQLRA
A0A151TXV6_CAJCA    MT--------------------------ESLQSLVSLSELVSTSSTKQRFRIFHR
A0A0M9ABE9_9HYME    MA------------------------DVELTKALKDLPNRIQTASKNERRVLLQN
A0A072U5F0_MEDTR    MA--------------------------ESLQSLVSLSELVSTSSTNQRLRIFQR
A0A0K8V1F4_BACLA    MA------------------------EIELSKALRDLPNRVLSAKCDERKDLFQS
A0A0A1XE05_BACCU    MA------------------------DIELSKALRDLPNRVQTATCDERITLFQN
A0A131YTA4_RHIAP    MA------------------------GVEVPPVFKDLPVKVQTSKLVTRKELFCQ
A0A0J6I2R9_COCPO    M----DEIKWNNV----------------HDGMLESLKPVIFSGSTSRRRAGLHE
A0A074XMM5_AURPU    M----AEGEVNGE----------------QTLDAGRLRQVALSSSTKKRSTELLS
A0A167FV98_9ASCO    M------------------------------------------------------
A0A1A7XZM1_9TELE    MAA-----------------------DTQVSDTLKKFAVRVTTASVNERKEIYRD
A0A0P7V1U6_9TELE    MAGRESAALMDSGSLFHHMIEARTKNQQTVSETLKKFALKVTSASVKERRDILQE
A0A1A7WWE1_9TELE    MAA-----------------------DTQVSDTLKKFAVRVTTASVNERKEIYRD
                    *
```

```
GCN1_YEAST          -----ISQLVRQET--LEKPQLSEIAF------------VLLNTFTIYEDNRSKS 110
GCN1_SCHPO          -----LQHICRTQD--IDEESAIAALE------------DIFETLPRNLSRDARK
GCN1_DICDI          -----INSIGKHEL-STQENDATTTTTTTTVDLEEKLKVLIYLYFTSYSIGPDSQ
GCN1_MOUSE          -----LGRCIAGKD--LPEGAVKGLCK------------LFCLTLHRYRDAASRR
GCN1_HUMAN          -----LGKCVAGKD--LPEGAVKGLCK------------LFCLTLHRYRDAASRR
M7ANV9_CHEMY        ------GHSAMSVD--DTEPPSPVGTK------------MFPFL---YRDAASRR
H8X4E7_CANO9        -----IESNLDD----IEKAELQEVTF------------VLLNTYNFYQDTDSRN
W6A2P0_ICTPU        -------------------------------------------------------
B6JZI6_SCHJY        -----IRVVDSTHE--FDNELIECILK------------FYAETLHRTLPRRVRV
R4WJK2_RIPPE        -----LRSAISNSG--FSENIVRGLCR------------VLQMTLPRYCDSASQG
W8CCK9_CERCA        -----VAAVLDNPG--MNESIIKGICK------------VIGTTLTKYKDSASQL
B0W357_CULQU        -----VTAVLPNPG--INATIVRGICK------------VIGTTLTKYKDPASQA
E1ZX97_CAMFO        -----VVNVLSNPG--INDKIVNGICK------------VVSLTLHRYKDSASRS
W5JJ28_ANODA        -----VIAVLPNPG--INATVVRGICK------------VIGTTLTKYKDPESQA
F1KPR4_ASCSU        -----LIGALKNVS-EVSEPIMRGMIR-----------GSVTACFTRFTQRRSFE
V9K7F0_CALMI        -----LGSCVKEKD--LPDSAVKGLCK------------LLCLTLHRYRDAKSRR
W0T7Q3_KLUMA        -----VLQWLKLEDSKTNEAALGQIFE------------CVLGTYDFYQDKESKN
S9R951_SCHOY        -----LRHVNQIND--IDEKTIVVIID------------NIMEVLPRNVSRECRL
S9W134_SCHCR        -----LRHVNQTNE--IDEKSIVAIID------------NIMEVLPRNVSRECRL
H0GUK0_SACCK        -----ISELVRQET--LEKPQLSEIAS------------CLLNTFVLYEDNSSKS
E5R2E4_ARTGP        -----LQDNIASDS-QLPHQLYKKVLL------------LLFRTYPVFIDRESRH
J8Q888_SACAR        -----ISQLVRQET--LEKPQLSEIAS------------VLLKTFVVYEDTKSKS
C5G992_AJEDR        -----LHEKLSGLQ--ISQNLFRSIVK------------LLFRTYPFYVDRASRQ
G4MR95_MAGO7        -----VEDKISQNA--LDQASLSKLLG------------VLFLTHSSYADRPSRR
C9SQ16_VERA1        -----LQERITQKK--FDLSLVPKLLQ------------LLFWTHAFYVDRQSRL
B2WNI9_PYRTR        -----LQHQVADDS--LVNADLQAILE------------NLFETYPLYEDRESRR
S3CN37_OPHP1        -----IDDKINSGN--LEPAAIAKFLP------------IMFWTHASYEDRPSRR
G3ALT0_SPAPN        -----IQQLLNA----IPESDLTKISL------------LLLKTYNYYHDADSRN
Q7S5P0_NEUCR        -----IEEKLSQKA--LDRPTTLAVLK------------VLFWTHDFYHDRPSRT
A0A165FVX9_9PEZI    -----LQERISHQD--IPQKSFSLILA------------SLFETYPLYIDRASRQ
A0A178DN93_9PLEO    -----LQHQVGHDS--LVASDLQALLE------------LLFETYPLYDDRESRR
A0A177D7J2_ALTAL    -----LQHQVADDS--LVTADLQALLE------------LLFETYPLYDDRESRR
A0A0C4DS68_MAGP6    -----VEDKLSQDA--IDQTGLSKLLP------------LFFLTHAFYHDRPSRR
A0A151N4J5_ALLMI    -----LGRCVTGKD--LPEGAVKALCK------------LFCLTLHRYRDAASRR
A0A146NKU7_FUNHE    -----LKECLKGKE--LPEPAVRGLCK------------LFCLTPHRYRDAASRR
A0A0F7Z917_CROAD    -----LGRCITGKD--LPEGAVKGLCK------------LFCLTLHRYRDAASHR
A0A0W0DC37_CANGB    -----LKEIVKTNN--LSREQLLNLAS------------VLLNTYTIYKDHESKK
A0A0L8RIK7_SACEU    -----ISQLVRQET--LEKPQLSEIAS------------VLLNTFVVYEDTKSKS
A0A178FRD2_TRIVO    -----LQDTIARDS-QLPPQLYKTVLL------------LLFRTYPVYIDRESRH
A0A175WD14_9PEZI    -----IDEKISQKS--LDQASTRSLLG------------VLFWTHAFYNDRPSRQ
A0A194VFU1_9PEZI    -----VDDKISQNA--LDKQIQLQLLK------------LLFWTHSFYGDRESRR
A0A151TXV6_CAJCA    EIPAFLNSCTSE----MSTELASLLTD------------VIFRTVPIYDDLRSRK
A0A0M9ABE9_9HYME    -----VVSVLSNPG--INEKIVNGICK------------TISSTLHRYKDTASQS
A0A072U5F0_MEDTR    EVPAFLNSSSTSDE--MSTELASLLTD------------IIFRTVAIYDDRRSRK
A0A0K8V1F4_BACLA    -----VTSVLNNPG--VNASIVKGLCK------------VIGTTLTKYKDTPSQI
A0A0A1XE05_BACCU    -----VTSVLDNPG--INASIVKGICK------------VIGTTLTKYKDTPSQI
A0A131YTA4_RHIAP    -----LEEAAKTGS--LADPVVRGLCQ------------VLRLTLPRYHGSTSRH
A0A0J6I2R9_COCPO    -----LHEKILSHE--LPKELDRPLLD------------LLFGTYPIYVDRDSRR
A0A074XMM5_AURPU    -----LHDKIVSKE--YPIDQIPNALN------------VLFDTYPLYVDTQSRQ
A0A167FV98_9ASCO    -------------------------------------------------------
A0A1A7XZM1_9TELE    -----LKQCLKG----VPEPAVKGLCK------------LFCLTPHRYRDAASRR
A0A0P7V1U6_9TELE    -----LKQCVTGKD--LPEPAIKGLCK------------LFCLALHRYRDAASRR
A0A1A7WWE1_9TELE    -----LKQCLKG----VPEPAVKGLCK------------LFCLTPHRYRDAASRR
```

```
GCN1_YEAST          LVTSILLDILNLE-PCLLEN-----------------FIRFISDVVISNP--ATK 165
GCN1_SCHPO          QIEITINHLVSRL-PSIVLPF----------------LVRRLTTIAGRLDR-FRS
GCN1_DICDI          WIFSLVQSLKQLF-KDISSATNTSLVTDELKINIVKFTLREIGRLAKLLPT-NKR
GCN1_MOUSE          ALQAAIQQLAEAQ-PEATAKNLLH-------------SLQSSGVGSKACVP-SKS
GCN1_HUMAN          ALQAAIQQLAEAQ-PEATAKNLLH-------------SLQSSGIGSKAGVP-SKS
M7ANV9_CHEMY        ALQSALQQLAESQ-PEATAKNLLQ-------------SLQSLGISGKAGVP-SKS
H8X4E7_CANO9        AVLEALLRISKVD-GRF--------------------LSYYVEFIKKETSG-TTL
W6A2P0_ICTPU        ----------------------------------------------------GKS
B6JZI6_SCHJY        AIVRSLKHLVNCT-AHTVKC-----------------LASFIMKTVVRAER-AHY
R4WJK2_RIPPE        YVRSLVKALANIH-PNWTIKYLTP-------------ILAETANYNTSIIA-TKS
W8CCK9_CERCA        LVRNLIVDIIKQH-HDVAIIHMTT-------------VIKTILSKELSKLP-PQK
B0W357_CULQU        LVRDLIVALLQNH-ADLAYEHFNN-------------VLKALVTKDLVAAP-PLK
E1ZX97_CAMFO        YVRNLIIELLKKQ-PEATIKHMTT-------------VISEQATWHKNVVA-TLN
W5JJ28_ANODA        LVKELLVAVLKQH-PDLAYEHFNA-------------VLKALLTKDLAAAP-PLK
F1KPR4_ASCSU        VIFSLIEALTERN-AIAVSKYISE-------------TLANVFTPTRTAIT--KW
V9K7F0_CALMI        ALQSVIQQLAESH-PEATTKHLLQ-------------CLQSSGIICKNAIP-SKS
W0T7Q3_KLUMA        AAISILVQLFEID-DSFLKK-----------------ITDFVIVTGSSHSG-GSK
S9R951_SCHOY        ELCATLAHITKLS-PSIALTA----------------LLHRLQSISNRLERVPSS
S9W134_SCHCR        QLCATLSHITKLS-PSIALCA----------------LVNRLRSTSIRLERIPSS
H0GUK0_SACCK        LVTSILLDILNLE-PCLLES-----------------FVQFISDVVISNP--ATK
E5R2E4_ARTGP        AAQSCAKALFPSI-PCAD-------------------LPAFAQLLRKESSN-AAV
J8Q888_SACAR        LVTSILLDILKLE-PCLLES-----------------FVQSISDTVISNP--ATK
C5G992_AJEDR        AVQKCLRSLFRIP-TANPD------------------LQYFIQELKKECSK-STI
G4MR95_MAGO7        AVQRCLVLLAKAK-EEKL-------------------LTALVAAIRKESQK-PTI
C9SQ16_VERA1        GAQKCLSALLASGLEPEL-------------------LAKLASILRAESVK-PGI
B2WNI9_PYRTR        AVEAVLKSLVEGP-HGDTV------------------LPPIVKFLKQECSK-KGL
S3CN37_OPHP1        AVQKCLVSICDRK-EPAL-------------------LTPLVNAIRTDAAK-INT
G3ALT0_SPAPN        ELLKTLQAIADAK-HHY--------------------LSGFIKFINHQVSA-TTL
Q7S5P0_NEUCR        AVQKCLVALAQNR-EPEI-------------------LTPLVAAIGQEIKK-GAI
A0A165FVX9_9PEZI    AVQQCLLAIVNSG-IKSGLD-----------------LSPLIKLIKDEAAK-STI
A0A178DN93_9PLEO    AVEGVLKSLVNGP-HGDTA------------------LPPIVRFLKQECLK-KGI
A0A177D7J2_ALTAL    AVEGVLKSLVNGP-HGDKV------------------LPPIVKFLTNECSK-KAI
A0A0C4DS68_MAGP6    AVQRCLVIFSQKG-DEKL-------------------LTALVGAIKREAQK-PTI
A0A151N4J5_ALLMI    ALQLALQQLAESQ-PEATAKNLLQ-------------SLQSSGISGKAGVP-SKS
A0A146NKU7_FUNHE    ELLSVIGQLAERQ-PDALVSGLLQ-------------GLLNCGVISRNGEP-SKS
A0A0F7Z917_CROAD    ALQTALRQLAECQ-PEVTAKNLLH-------------SLQSSGISSKSGGS-SKS
A0A0W0DC37_CANGB    AVCDIYDEMITID-GSFIAI-----------------FLDHIVETLGDKI--GTK
A0A0L8RIK7_SACEU    LVTSILLDILRLE-PCLLEN-----------------FVQFISGVVTSNP--ATK
A0A178FRD2_TRIVO    AAQRCAKALFPSI-PCTD-------------------LPAFAQLLRKESSN-TAV
A0A175WD14_9PEZI    AVQRCFVLICQTR-DVDI-------------------LGRLVTAIRQETQK-QGI
A0A194VFU1_9PEZI    GVQKCLISICKEG-DADL-------------------LTPLVEAIRREAAK-PSI
A0A151TXV6_CAJCA    AVDDVIVK-------SLSRTVFMK-------------TFAGALVQNMEKQS-KFQ
A0A0M9ABE9_9HYME    YVKNLIEQLLKKQ-PVPTIKHMTN-------------VIVEQATWHKNVVP-TIN
A0A072U5F0_MEDTR    AVDDVIVK-------SLSGTVFMK-------------TFAAALVQSMEKQL-KSQ
A0A0K8V1F4_BACLA    LVRNLIVDIVKQH-HDVATESLSS-------------VVRTILNKEILILS-PQR
A0A0A1XE05_BACCU    LVRNLIVDIVKQH-HDVAIECLSS-------------VVKTVLNKEISVLT-PQK
A0A131YTA4_RHIAP    LVEQLVDSLLASQ-PSSA-GHLIS-------------TILDVSRDHKPLFI-TKN
A0A0J6I2R9_COCPO    DAQRCIRDIFQAP-IASVD------------------LKDFANKLHKECYK-PAI
A0A074XMM5_AURPU    AAEQCLVAIFSTA-DTEPH------------------VSTFISAVETETKK-GSL
A0A167FV98_9ASCO    -------------------------------------------------------
A0A1A7XZM1_9TELE    ELLSVIGQLAESH-PDVLVTSLLH-------------CLLNSGVISKNGEP-SKS
A0A0P7V1U6_9TELE    ALHSVIGTLAVAQ-PVVTATSLLN-------------CLLNSGIISKSGVP-SKS
A0A1A7WWE1_9TELE    ELLSVIGQLAESH-PDVLVTSLLH-------------CLLNSGVISKNGEP-SKS
```

```
GCN1_YEAST          AVADYLNLLDWIN-SFLIFVSHNSNLF-EEYIPKLLVAHSYATFGVETILDNQEE 220
GCN1_SCHPO          TVSVSFDCLNWVNSMI-------PNLPEKELQYWILELLPLQSSFLSYAL-----
GCN1_DICDI          SKYVPQNTLMSFSLVLLNYFIDQIQSNSDLT-TLLFAAQELLYRELTLQH-----
GCN1_MOUSE          SGSAALLALTWTCLLVRIVFPLKAKRQGDIW-NKLVEVQCLLLLEVLGGS-----
GCN1_HUMAN          SGSAALLALTWTCLLVRIVFPSRAKRQGDIW-NKLVEVQCLLLLEVLGGS-----
M7ANV9_CHEMY        SGSAALLALSWICLLVRIVFPTRDKREGETW-KKL--------------------
H8X4E7_CANO9        AITDYLTLLQWIN-SFNIELAKAKNLKGSC----VALQEKLFVNCTEYEG-----
W6A2P0_ICTPU        TASASCCALPWTCLIVRVVFPTLENRVDPQW-KKLVEVQSLLLAEVMGGA-----
B6JZI6_SCHJY        PPGFLLTTLSWLNDVVMS-----TCTSAEVCESLVKNLCPIQAKLLFMCL-----
R4WJK2_RIPPE        TAQGSLQALSWSSILVSSALNKCSDENKQDF-QTILNAQSLLYSSVLAST-----
W8CCK9_CERCA        CAKFSLIALGWTDLIIRNGTSN-SNVYKTEY-PKLVEYQSILYQHILLSC-----
B0W357_CULQU        GAQAAVLALGWANLVALHGDRE-TAVGKKEF-PKLIENQAALYQLSLTSG-----
E1ZX97_CAMFO        TSLTAYTALKWSNLVILHGYNELDNVEKNEFLAKLIEAQANLSSAALASV-----
W5JJ28_ANODA        GAQAAVLALGWANTVALNADHE-SAVGKKEF-PKLLEVQAGLYQLALTSG-----
F1KPR4_ASCSU        SGWMSTVGVKW---LVFVGQKADLLQHKDLIDFFVSSLSSVAFYSVADKK-----
V9K7F0_CALMI        SGSAATLALGWTLLLVKTVFAVPEKRAGEMW-MRLVELQCLLLAEVLGGC-----
W0T7Q3_KLUMA        AIMDYLNLMEWIL-VFMDLVFKKHDASFEAHWTDIIQAYIALVGAIETMLDTHET
S9R951_SCHOY        AISVSFNALSWVNCIL-------SNLPSNEMKYWMLEFLPLQSQFLNAVI-----
S9W134_SCHCR        SISISFNALIWVNCIL-------SNLPSNEFKYWMLEILPLQSQFLNVVL-----
H0GUK0_SACCK        AVADYLNLLDWIN-SFLVFVSRNSMLF-EEYLPKLLVAHSYATFGVETILDNQED
E5R2E4_ARTGP        APANAFVLVEWCA-TLLQYL----STRLDDHSPLVLETVSGIGKALETCL-----
J8Q888_SACAR        AVADYLNLLDWIN-SFLVFVSRNSNLS-EEYIPKLLVAHSYATFGVETILDNQED
C5G992_AJEDR        AATNAFVLAEWCS-LLLQLL-----SEVPDFTQSILEILSADAKVVEICL-----
G4MR95_MAGO7        APANAFVLLEWSS-LLIEHL-----GETELWEKLGKELVLATSDCLEKCL-----
C9SQ16_VERA1        AITSAFVLVEWCS-IFMQEL-----SGTPSWHQVAPQILLANADSLEKCQ-----
B2WNI9_PYRTR        APTNAFVLVDWASVLLLQLA-----KSAETWSKHGLDLATANALALETCVR----
S3CN37_OPHP1        SVASSFTLVEWCS-ILMQHL-----AGTELWDKVSKDVLTAQANALEKCT-----
G3ALT0_SPAPN        AITDYLSLLYWIN-TFNVDMMK-YGLEKQDGELLVRTQAIVLNKCIEYEGV----
Q7S5P0_NEUCR        APGSAFTLVEWCS-LLVQNL-----AGTPLWEKFGKDLTLSLAEVLEKCC-----
A0A165FVX9_9PEZI    APTHAFVLVEWCS-LLLQRF----STESSLWEKWGLDLLLADSQLLELCQ-----
A0A178DN93_9PLEO    AHVNAFVLVDWCSVLLLQFA-----KSPERWSKYGLEVALANARVLETCMS----
A0A177D7J2_ALTAL    AHANAFVLNDWCSVLLLQFA-----KSPERWAKHGLDVALANARVLETCVG----
A0A0C4DS68_MAGP6    APSSAFVLLEWCC-LLVENL-----AGTALWVKLGKELILASADCLERCL-----
A0A151N4J5_ALLMI    SGSAAALALSWTCLLARVVFPTWDKRQGEIW-KKLVEVQCLLLLEVLGGS-----
A0A146NKU7_FUNHE    TGSAAFIGLSWTCLLIPKVFSAPEKREGPIW-KKMVEVQSLLVAEVVGGA-----
A0A0F7Z917_CROAD    SGSAASLALSWTCLLVRIVFPTPDKRHGETW-EKLVEVQCFLLLEILGGS-----
A0A0W0DC37_CANGB    ALSDYLNLLDNIN-HFLQVILVDSNLT-SQYLPKVLQCHTITTASIETSLDISEK
A0A0L8RIK7_SACEU    AAADYLNLLDWIN-SLLVFVSRNSSLP-EEFIPKLLVAHSYATFGVETILDNQED
A0A178FRD2_TRIVO    APANAFVLVEWCS-TLLQYL----STRLDDYSPLVLETVFAISKALETCL-----
A0A175WD14_9PEZI    APGSAFVLVEWCS-LLMQNL-----AGTPLWDNFGKDIVLASADGLEKCL-----
A0A194VFU1_9PEZI    APTNAFVLVEWCS-LLMQNL-----AGTPLWDKFGKDIIVAAADALDKCC-----
A0A151TXV6_CAJCA    SHVGGYRLLSWSCLLLSKSQF--AAVSKNAL-CRVAAAQASLLSLVLPRS-----
A0A0M9ABE9_9HYME    TSLTAYLALKWSTLIILHGYKSNSDMNSEL--PKLIEAQANLSAAALASM-----
A0A072U5F0_MEDTR    SHVGCYRLLSWSCLLLSKSKF--STVSKNAL-CRVASGQASLLNLVWRRS-----
A0A0K8V1F4_BACLA    SAKYSLIALGWTDLIIRNGDYN-SNIFKTEY-SKLVECQSILYYNIYLSY-----
A0A0A1XE05_BACCU    SAKYSVIALGWTDLIIRNGDFH-SNIFKTEY-SKLVEYQSQLYHHIVLSY-----
A0A131YTA4_RHIAP    LARTCLTAFSWSCKVGHQVLVNRGDLKPDKI-RDLVEAQTNLLLSVSSAC-----
A0A0J6I2R9_COCPO    AAANAFVLIEWCT-LLLQHLGQREAENPEDAPQLAVNVLSANSKALETCL-----
A0A074XMM5_AURPU    APANAFVLIRWCA-LFLQQF----ALQRPLWDTWGLKIIHALVRATYTFE-----
A0A167FV98_9ASCO    ------TVLEWINGIISEKKTAKLALSTEFVAAHAILLEKAVY------------
A0A1A7XZM1_9TELE    SGSAAFIGLSWTCLLVPKVFSAPEKRDGPIW-KKMVEVQSLLVAEVVGGA-----
A0A0P7V1U6_9TELE    TAPAAAMALSWTCLLVRAVFPTPESREGPTW-KKLVEVQSLVLAEVVGGA-----
A0A1A7WWE1_9TELE    SGSAAFIGLSWTCLLVPKVFSAPEKRDGPIW-KKMVEVQSLLVAEVVGGA-----
```

```
GCN1_YEAST          G-KKSQDKQNQHRKRIRYCIFQTTVKAFLKCLKD-NDDSISFMKISIKTVLESYS 275
GCN1_SCHPO          ---------RDGKPSVADSAIKSTRRCYRSLFC----KKMDSLKKLVSFLLTETE
GCN1_DICDI          ----------------MKN-----NQPIFNQIL----NKNKILTFYQNIVTKDS-
GCN1_MOUSE          ----------------HKHAVDGAVKKLTKLWK----ENPGLVEQYFSAILSLEP
GCN1_HUMAN          ----------------HKHAVDGAVKKLTKLWK----ENPGLVEQYLSAILSLEP
M7ANV9_CHEMY        -------------------------------------NPGLVDQYLSVILSLEP
H8X4E7_CANO9        --------KTKHLDRVTKSAITATKTSIVNTLL--------EDDTYMDLIINQDC
W6A2P0_ICTPU        ----------------SKNAIKSVTKRLNKLWK----DSPGLVDQYMSTLLSLDQ
B6JZI6_SCHJY        ---------SSTKHTLANSALRSTRRCIRSLLL----TNSKNYESFVDKFFEALL
R4WJK2_RIPPE        ----------------NKKKISKANQMLTSLWN----SNDLILSLYEKGVLCLPD
W8CCK9_CERCA        ----------------NIRIVEMAEKIFFDSWL----NSYIF-DMCFSTMLTKEP
B0W357_CULQU        ----------------IQKISDKAYSFVREFFA----KKEDLEKVYFEKLIAAEP
E1ZX97_CAMFO        ----------------NKKLANKVYTLFAHEWA----AIKNIEVIYMENLTKLEL
W5JJ28_ANODA        ----------------IQKISDKAFSFLCQFFG----HQEGLERSYFDKLIALEP
F1KPR4_ASCSU        ----------------NASYARGKMKYLCTRLP----IKVVLDSAQRQFASSSTD
V9K7F0_CALMI        ----------------KKTAVRGVLKKVKRLWN----ENPGLAEQYLVTVMDLET
W0T7Q3_KLUMA        V-KHDENKQNQHRKRLRLCVFQQSTKSFARCLTLERDSNSNIVEDIIPFVLDNYV
S9R951_SCHOY        ---------RDAKPAIVRSALQNTRRCYRSIFL----AQLNDLSSFIPFLLNETK
S9W134_SCHCR        ---------RDGKPAIIRSALHSTRRCYRSLFL----AQLNDLSSFISFLLNETK
H0GUK0_SACCK        S-KKPKDKQNQHRKRIRQCIFQSTVKTMLKCLKD-NDDGMFCMQTVTKSVLEDHS
E5R2E4_ARTGP        --------GTSTKQSLKHSAIIVTRRALRAVFS----AEPHGNNLVRDVVPYLIK
J8Q888_SACAR        T-KKPQDKQNQHRKRIRHCIFQSTVKAFLKCLKD-NEADISYIETVTKTILEDHS
C5G992_AJEDR        --------RAKTKGSVKHSAIIVTRRALRAVIS----PEGNGEEILRRAIPLLTS
G4MR95_MAGO7        --------QPQCRNNLGSSAQVVTRRAFRKLVS----LEQTREKSITDAVQLLAT
C9SQ16_VERA1        --------QPAGRGTVARAAIVVTRRGLRKLFA----PTADLEKTVKEAVELLTA
B2WNI9_PYRTR        ------AGPHRRADRIAASALVSTRRALRAIFR----SETFGQNALSKLVTTFTA
S3CN37_OPHP1        --------RDSVRPSVTHSALVVARRGFRKLVS----IDSTREKAVNEAVQLLAA
G3ALT0_SPAPN        -------KKHQHRRRVHGSAIHTTKTSIVDALT-----ITEDKEAYIELIVKSIT
Q7S5P0_NEUCR        --------QPASRGTVSKSALVITRRGLRKLA-----AGDDREKVIEEAVKALAA
A0A165FVX9_9PEZI    --------TPRTRNSVKHSALVVTRRALRKVFT----TGALGESAIKDSVGRLTA
A0A178DN93_9PLEO    ------AGSNRRAGRISESALVSTRRALRAILR----SKAIGKDALSTLVKTLTT
A0A177D7J2_ALTAL    ------AGGDRRAGRISIAALVSTRRALRAIFR----SEAIGKDAHSKLITTLSA
A0A0C4DS68_MAGP6    --------QSPFNKHVGHSALVVTRRAFRKIVS----FESTREQAITEAVQLLAT
A0A151N4J5_ALLMI    ----------------HKHAVDGAVKKLKRLWK----ENLGLVDQYLSVILGLEP
A0A146NKU7_FUNHE    ----------------KTTAKKSSLKNLCHLWE----EKPGLVDHYISTLLTLDQ
A0A0F7Z917_CROAD    ----------------RKPVVNGAVKKLNRLWK----ENPGLVDQYLSIILHLEA
A0A0W0DC37_CANGB    SMELVSEKQNQHRRRMRKTIIQNSTKYLNKGLKN-TENIQDVLETIRHQLLEEYV
A0A0L8RIK7_SACEU    S-KKSQDKQNQHRKRIRQCIFQSTVKTFLKCLKD-NNDCLSYLQTVTNTLLQEHS
A0A178FRD2_TRIVO    --------GTSTKQSLKHSVIIITRRALRAVFS----AEFHGSNLVRDVVPSLIK
A0A175WD14_9PEZI    --------QPTSRATVGSSALVTMRRGIRKLAL-------ADTKAIDAAIQVLAA
A0A194VFU1_9PEZI    --------SPASRPGVAHSALVVTRRGFRKLVS----FTDSREKTITESVKALTT
A0A151TXV6_CAJCA    ----------------FRE-RRACRKKFFRLFS----QSPDIYKVYMEELRNGRI
A0A0M9ABE9_9HYME    ----------------DKKLTNKVYVLLAHQWS----SVKNIDIVYLETLTKLEA
A0A072U5F0_MEDTR    ----------------FRE-RRACKKKIFHLFK----ELPDIYKVYVQEVKNGSI
A0A0K8V1F4_BACLA    ----------------NMRIIDMAEKLLFDSWI----NSENY-ILCSSTIILKEP
A0A0A1XE05_BACCU    ----------------NIRIIEMAEKLLFDLWT----NIENY-KLCSSTILQKES
A0A131YTA4_RHIAP    ----------------DPTLNKKLKRKINYLWK----QVKT--DLYAEVLSSLEP
A0A0J6I2R9_COCPO    --------RSSKKEGLKHSAIIVSRRALRTVLS----KEETGDEVVRRLVRVLTS
A0A074XMM5_AURPU    --------SSNPRHSTHQTVLRVTRRAFRKLFS-----SDFGDEALNQSVSALTV
A0A167FV98_9ASCO    --------STEHRERLAHSALKTTRYAFSKYLI----NTSDTTNGYLQSLFDQKN
A0A1A7XZM1_9TELE    ----------------QATAKKSSLKSLAHLWQ----EKPGLVDHYIGTLLTLDQ
A0A0P7V1U6_9TELE    ----------------QRNTLKSALKSLSQLWK----ENPGLADQYMSTLLSLDQ
A0A1A7WWE1_9TELE    ----------------QATAKKSSLKSLAHLWQ----EKPGLVDHYIGTLLTLDQ
```

```
GCN1_YEAST           KLKITSVGVVMIMGALTQAALQLLSRQPALHSVLKENSAEKYCEYLGKEVF-LGK 330
GCN1_SCHPO           NAILPPKS-LPLYGVIISTCYYFHQSPNPRNEISQ--QAELFSKIMAQNVL-MAK
GCN1_DICDI           SNKSF----FIVYFLLRNFTDQSNKQKEQDNTF----KINDLLNIYNKTVIGSSQ
GCN1_MOUSE           SQNY-----AAMLGLLVQFCTNHK--EMDAVSQ----HKSTLLEFYVKNIL-MSK
GCN1_HUMAN           NQNY-----AGMLGLLVQFCTSHK--EMDVVSQ----HKSALLDFYMKNIL-MSK
M7ANV9_CHEMY         NQSY-----AAMLGFLVQFCTSQK--EMDVVNR----HKSALLDFYMKTIV-MSK
H8X4E7_CANO9         ---------LALFGVLNLAAIELIPKKPSMLEKFK-SHESKVLNTFINLVL-LGK
W6A2P0_ICTPU         SPSS-----LPLLAECVDFCTSQK--DMATINK----HKAAILDLYLKTVL-MSK
B6JZI6_SCHJY         PTLPMPNG-LAFVGEFLSCCYYFKLSKGPLEALGK--HKQTIATLLVKQVF-AAK
R4WJK2_RIPPE         STHS-----IVFGSFFIKYLCEKK--SAEILE-----YKTKILDLIVKVVI-MSK
W8CCK9_CERCA         AHSV-----IILIMLLIRFERRHSDNKEYDIK-----YKSKLLEYFVKGVI-TSK
B0W357_CULQU         SSAV-----ILFLSAILTYC-KTEFDNLTLLEQ----NKAKLLDHVVKGLI-TVK
E1ZX97_CAMFO         GNGV-----IILASLLMKYLVAAK--RHDLVEQ----LKVNMIDIFIKVTI-SCK
W5JJ28_ANODA         SSGV-----IVMLCAILRYCQQQEHDSVALLQQ----HKAKLLDHVVKGLV-TVK
F1KPR4_ASCSU         AERV-----LALLSLLSFEEDNTD-------------VVAFFLQIYLKAIL-MAK
V9K7F0_CALMI         NLLY-----LGMLGVLLQFCSEQK--DIATINK----HKDALLDIYVKAVL-MSK
W0T7Q3_KLUMA         KLKLSNTGIIVLLGSLVHSVLQCQSKKPIPYNSLE-ELTEKLVEYIGKEVI-LAK
S9R951_SCHOY         AALVPPKA-LPLYGVILGTCYYFQLSSTPRTLASE--NIDFLVKMIPQHVL-TAK
S9W134_SCHCR         PALVLPKA-LPLYGVILGTCYYFQLSSEPRALASE--NIDPFVKMIPQHIL-AAK
H0GUK0_SACCK         KLKMTSVGVVLIMGALTQTALQLLSRQPALQSALKESSVEKYCEYLGKEVL-LGK
E5R2E4_ARTGP         ETAS-GFKNAPILGVICGVCARLPNRKSDLDA-----MKGDIFQYYSKEIV-ASR
J8Q888_SACAR         KLKMTSVGVVVMMGAITQTTLQLLSRQPTLHSYLKENSVEKYCEYLGKEVF-LGK
C5G992_AJEDR         EAVS-GSRNAPFLGIVAGVSDRIPNRKPILED-----LKTAILQYYAKEII-GSR
G4MR95_MAGO7         KGAAPTAKYAPTLGVIAGVCARKDYGKPILEK-----LKPQFFGFYTREII-GSK
C9SQ16_VERA1         KAAQPTAKNAILLGVIAGVASRKPDAGAVLET-----LKASYFAFYTREVV-GSR
B2WNI9_PYRTR         KGSAPTAGNAVFLGVIAGVSSRLPTVKPVFEK-----HKADYYTFYTREII-GSR
S3CN37_OPHP1         KGTAPTARNAVFLGVIAGVSVRNAKAKPIFEA-----TKPQYFEFYTREIT-ASK
G3ALT0_SPAPN         KNTADEG--FSILGALNLAIIELVPTIPSLTEIFA-SKDKEILEYYSNQGL-LNK
Q7S5P0_NEUCR         KGTQPTAKYAVLLGVIAGVCSRKPEAVPIIEK-----LKSQYFTFYTREIV-GSR
A0A165FVX9_9PEZI     KAPAPVLKNAIFLGVIAGVCARIEKLKPVLYE-----LRKDFYSFYAREFL-GSR
A0A178DN93_9PLEO     KGPAPTAGNAVFLGVIAGVSSRLPEVKPLLEA-----HKQDYTAFFVREIV-GSR
A0A177D7J2_ALTAL     KGSAPTAGNAVFLGVIAGVSSRLETVKPLLEA-----HKQDYYTYYTREIV-GSR
A0A0C4DS68_MAGP6     KGAQPTAKYSVTLGVIAGVCSRKDYAKPILEK-----LKPQYFGFYTREII-GSR
A0A151N4J5_ALLMI     NQSY-----AAMLGLLVQFCTIQK--EMDVVNR----HKSALLDFYVKTIL-MSK
A0A146NKU7_FUNHE     SPTT-----LPMLGVCLDFCSAQK--DKATIEK----HKSALLDLYIKSVL-MSK
A0A0F7Z917_CROAD     NQGY-----AIMLGLLVQFCTNQK--DLGTINK----HKGALLDFYLKTIL-MSK
A0A0W0DC37_CANGB     KLKLPVAGILVFSAALTKAALQLHVQTPIVLEFLRTTYSKDFVVFFGKEVL-LGK
A0A0L8RIK7_SACEU     KLKITSVGIVVIMGALTQTTLQLLPRQPILYSDLKENSVEKFCEYLGREVF-LGK
A0A178FRD2_TRIVO     EATS-GFKNAPILGVICGVCARLPNRKNDLDA-----VKGEIFQFYSKEIV-ASR
A0A175WD14_9PEZI     KGSKPAATNAVLLGVVAGVCSRKPEAKPVLEG-----LKSHYFAFYSREIV-GSR
A0A194VFU1_9PEZI     KATTPSSRYAIMLGVIAGVCSRQADSKPILEK-----LKSEYFTFYTREIV-GSK
A0A151TXV6_CAJCA     PFKDN----PELLLLLLDFSSR----SPSLFAE----YKPAFLDLYVNAIL-SAK
A0A0M9ABE9_9HYME     GNGV-----IVLASLLTMYLVNTK--KSDLVIK----LKTNTIDAFIKLTI-SCK
A0A072U5F0_MEDTR     PYKDS----PELLLLLLEFSTR----SSSLFGE----FKSAFLDIYVNAIL-SAK
A0A0K8V1F4_BACLA     AFNI-----INFLMLLLRFEHFKGH-EEYILNR----HKSELLQHFIKSII-INK
A0A0A1XE05_BACCU     ASHI-----VVFLMLLLRFENSKED-IEYDQRM----HRAELLEHFIKVVI-TSK
A0A131YTA4_RHIAP     SYAV-----LLMWCYLIAYLDAQK--QRDVIHS----YKAKFLDVFKTAIL-LSK
A0A0J6I2R9_COCPO     ETGS-GFRNAPYLGVLSGVCARLPKRRAVLDD-----VKPAIFQFYIKDII-GSR
A0A074XMM5_AURPU     KAAAPTPQNALVLGIIAGVSARLPKRATTFET-----KKSDYNAFFLREFI-GSR
A0A167FV98_9ASCO     LSS------IILLSAMAGAASDLVSSLPSASELIK-DHKENIYNLYVQVVL-SSK
A0A1A7XZM1_9TELE     SSTT-----LVMLGVCLDFCTAQK--EKATIEK----HKSPLLDLYIKSVL-MSK
A0A0P7V1U6_9TELE     NPSA-----LALLCVCVDFCTAQK--DMSTVQK----HKAALLDLYLKTVL-MSK
A0A1A7WWE1_9TELE     SSTT-----LVMLGVCLDFCTAQK--EKATIEK----HKSPLLDLYIKSVL-MSK
                                                                          :
```

```
GCN1_YEAST            NPPSSFCLEIGLKPFLKEFVSQELFIKFFIPNIEKAVLRSPEVGFSILSELYAGV 385
GCN1_SCHPO            PALEKYLYH-EFCYSLGLLLSVDQLKLYLLPSIEKALLRSPEIIFSGILSSLAHG
GCN1_DICDI            QKIEEHK---FFKRLFNQ-LTNEDLQSIILPPLSRHIKRDQDQVFKILIFILENL
GCN1_MOUSE            AKPPKYLLD-NCAPLLRF-MSHSEFKDLILPTIQKSLLRSPENVIETISSLLASV
GCN1_HUMAN            VKPPKYLLD-SCAPLLRY-LSHSEFKDLILPTIQKSLLRSPENVIETISSLLASV
M7ANV9_CHEMY          TKPQKHLLD-NCAPLLRY-VTHSEFKDLVLPTLQKSLLRNPENVIETISCLLASM
H8X4E7_CANO9          KAPSKFEVE-GFGPCVETLVNEDNFGTLLLPAIEKAILRSSENSFGIVLPIFFKN
W6A2P0_ICTPU          TRPLQHILA-KSSSLLCH-VSHSEFKEQLLPTLQKALLRSPENSIQTISSMLASL
B6JZI6_SCHJY          GIMPVALYA-EFTRGYGLVSSLDDFSSLVLPSLEKTLLRSPEVVFAGVITHLTSG
R4WJK2_RIPPE          VQIDPYVVQ-ACRPVLLL-ASHVDFSDTLLPSLQKAMLRNPELALHVVGRILANL
W8CCK9_CERCA          IKPHNSVIN-ACRPLLID-LSQEDFKENLFPVLLKSILRSPEVAIYTMGNIIHHI
B0W357_CULQU          TKPHASDIR-GSAIVLAA-ITKDDFKATVLPALQRSMLRSPEVILRAVGAIVSEI
E1ZX97_CAMFO          KKPDLYVVD-VAVPLLRR-VTHEEFKTQLLPALQKAMLRNPEIIIESVGHILSGL
W5JJ28_ANODA          TKPHASDIA-GCAILLRA-ITKDEFRTIIVPALQRSMLRSAEVILRAVGAIVNEL
F1KPR4_ASCSU          QRATPHIVG-ACSRLIER-LDATQLKQEVLPAAKKAMLRSPEVAIFGLSDGLRFV
V9K7F0_CALMI          SKPPKHLLE-WCEPLLRH-VSHQQFKDLLLPTLNKSTLRSPENVIETISFLLSSV
W0T7Q3_KLUMA          QPPSAFCLSVFLSEFLHTFVTEEQVSKILIPSFEKANLRAPETSFAASSEFYSAF
S9R951_SCHOY          PLLPKLSVE-GFCYSVGLLLSLDKLSSNLLPSIEKALLRSPELVFAGVLSSLVVG
S9W134_SCHCR          PALHRFAGE-GFCYSIGLLLSLDKLSSDLLPSIEKALLRSPELVFAGILSSLAIG
H0GUK0_SACCK          NSPSPFCLEVSLKPFLKEFVSQSLFTKFFVPNIEKAILRSPEVGFSILSELYAGV
E5R2E4_ARTGP          TPVPRHIHE-GIHDLFMSFSTKEDLQKHVWPAVEKAILRSPEILLAGVLVSLVSS
J8Q888_SACAR          NTPSSFCLEVSLKPFLREFVQQDLFVKFFVPNIEKAILRSPEVGFSILSELYAGV
C5G992_AJEDR          TVLPSHIVG-GLHDFFISFVTAKDLQVNIWPSLEKAILRAPEIVLGGLIPSLASS
G4MR95_MAGO7          TPVPSHFAE-GLYDFFHSFVTLEDLGKEVTPALEKGLLRAPEIVLNDLITPLVDA
C9SQ16_VERA1          VAVPDHVAA-GLRDFFSDFVTLDELTKDVIPTIEKGLLRAPEIVLE-VLAPLVRS
B2WNI9_PYRTR          TQLPDYVSK-GLHDFFDSFPTLEELRKDVFPPIEKALLRAPEVVLNDVLSPMILA
S3CN37_OPHP1          TTLTSNIAG-GLHDFFASFVTVEELDTLVVPALKKGLLRAPEIILDDLITPLVSS
G3ALT0_SPAPN          IAPSVASLE-LFGTCIESLVTVENFTSILLPNIEKAVLRSSENSFGVVLPVLFKQ
Q7S5P0_NEUCR          TQVPPHQAA-ALSDFFSSFVTLEDLDKEVFPSLEKGLLRAPEVVLNDLITPLVGA
A0A165FVX9_9PEZI      GLVPEHIAD-GLHDFFAAFATKQDLKENLIPSIEKALLRAPEVVLNGLITPLIRS
A0A178DN93_9PLEO      SQLPDHVSH-ALHDFFDSFPTLDELRKDIIPPIEKALLRAPEVVLNDIVSPMVLA
A0A177D7J2_ALTAL      SQLPDHISN-ALHDFFDSFPTLEELRKDVIPPIEKALLRAPEVVLNDIVSPMFLA
A0A0C4DS68_MAGP6      TPVPNHLAE-GLYDFFHSFLTLEELGKDVIPSLEKGLLRAPEIVLNDLVTPLIGA
A0A151N4J5_ALLMI      TKPQKHLLE-NCSPLLRY-MSHTEFKDLVLPTLQKSLLRSPENVIETISCLLVSV
A0A146NKU7_FUNHE      TKPQRHILD-KSGSLLRH-VTHSEFKELLLPALQKTMLRSPENAMQTVSCLLSAV
A0A0F7Z917_CROAD      TKPHKHVLD-NCAPLLRY-VSHSEFKDLLLPALQKSLLRSPENAIETISCLLASV
A0A0W0DC37_CANGB      TPPSSLCVATSIDTISQELFTEDMIKNDIIPVLEKSNLRSPESSFQYARALFGSI
A0A0L8RIK7_SACEU      NPPSPFCLEVGLKPFLKEFVSQNLFTTYFVPNIEKAILRSPEVGFSTLSELYAGV
A0A178FRD2_TRIVO      TPVPRHVHE-GIRDLFTPFSTMEDLQKYVWPAVEKAILRSPEILLAGVLTSLVSS
A0A175WD14_9PEZI      TPIPAHLAD-GLRDFFQSFVSLEDLDKEVFPALEKGLLRAPEVVLNDLITPLVRS
A0A194VFU1_9PEZI      TPVPKYLGE-GLGDFFSAFVTLEELDKEVIASLKKGLLRAPEVVLNDLFSPLVKD
A0A151TXV6_CAJCA      EKPGKSLIE-AFHPLYLQ-MSHEDFQNIVIPSSVKMLKRNPEIVSESVGILLKSV
A0A0M9ABE9_9HYME      KKPDLYVVH-NAVPLLRR-ISHDEFKSQLLPALQKAMLRNPEIIIESVGHILSGL
A0A072U5F0_MEDTR      AKPGKSLIE-AFHPLYLQ-MSHEDFGTIVLPAAVKMLKRNPEIVLESVGILLKSV
A0A0K8V1F4_BACLA      TRPQSAIVM-ACEPLLTI-ITQKDFEELIHPVLLKSILRSPEMAIRVMGLIFHKL
A0A0A1XE05_BACCU      IMPQNSIVK-ACEPLLKI-VTQQEFEGVIYPVLLKSILRSPEMAIRVMGLIFHQL
A0A131YTA4_RHIAP      TAAPTHVLE-HSRCLLRH-ATHEDFKEQLVPALQKAMLRNPEIIMESVAHVLQGV
A0A0J6I2R9_COCPO      TVVPSHIAG-SLGDFFISFASAEDLQKEIWPSLEKAMLRSPEVVFTGIIPSFVAA
A0A074XMM5_AURPU      TVLPQFIAN-ALVDYFNSFVTLEDLKKDIIPAIEKALLRSPEIVLNDLVTPMINA
A0A167FV98_9ASCO      VPLNRAVVD-SFQPLFVDYTLTEDFDTIILPAVSKSLLRAPEIVLQFIIPGLLSA
A0A1A7XZM1_9TELE      TKPQQHILD-KSGSLLRH-VTHSDFKELLLPALQKTMLRSPENAMQTISCLLSAV
A0A0P7V1U6_9TELE      SRPQQHILE-RSSSVLRH-VSHTEFQEQLLPALQKAMLRSPENAIETISCLLASV
A0A1A7WWE1_9TELE      TKPQQHILD-KSGSLLRH-VTHSDFKELLLPALQKTMLRSPENAMQTISCLLSAV
                                             .   . .    :   *  :
```

```
GCN1_YEAST          SPEK--VNLLNAFASSKLINQYFSSFKS--SKEVVRSVSLQSMIILLRKISNTDT 440
GCN1_SCHPO          FADS-KVDASSL-ILSSVLTSFVNGLKS--SNAEVRRNCFQTFKDLSAN----AS
GCN1_DICDI          SSDFNVIDLSSL-LKSMLLPMLLPVIQSTISIEENRKLLKKTFTLIIER----SK
GCN1_MOUSE          T-----LDLSQ--YALDIVKGLANQLKS--NSPRLMDEAVLALRNLARQ----CS
GCN1_HUMAN          T-----LDLSQ--YAMDIVKGLAGHLKS--NSPRLMDEAVLALRNLARQ----CS
M7ANV9_CHEMY        T-----LDLSQ--YAVDIVKGLACQLKS--NSPQLMDEAVVALKNLARQ----CS
H8X4E7_CANO9        LQ----IQIT---PSPKLLSSIISGVKS--QKEHVRNGASQTLALLIEK----AK
W6A2P0_ICTPU        T-----LDLSQ--YALDIGKGLASQLKA--SNMELMEHAVQAMQNLAQQ----CS
B6JZI6_SCHJY        FATA-GKDLSSV-LLQKITPAFISGFKS--SNATTRQNACATFTACAKL----AK
R4WJK2_RIPPE        S-----LDLSQ--YAYTVGKIISASLYS--NDENTRNQAASACKFLSLQ----CS
W8CCK9_CERCA        N-----IDLSE--YTSSFGKVILQNLYC--KDDVTRKESLETLKELSQK----CT
B0W357_CULQU        P-----VDISD--FAYDLGKTLVANLAS--KDETTRQEAVESLKQIAMK----CA
E1ZX97_CAMFO        S-----LDLSQ--YSQEISKGLFANLHS--KEDLVRDEAVGACRKLALQ----CS
W5JJ28_ANODA        E-----LDISD--YALDLGKPLVQNLAS--KEETVRQEAVESLKQVALK----CS
F1KPR4_ASCSU        K-----VDMSA--FALDLYKTLSASLVS--ADDEVRARASNAVISLACK----VS
V9K7F0_CALMI        T-----LDLSQ--YAMDISKGLASQLKS--NNTQLMDEAALAVRNLTHQ----CS
W0T7Q3_KLUMA        NSSK--INFLGLFVSSKCITQTISSLKS--SKEIVRESSLKSLIALLRSLKTDSY
S9R951_SCHOY        LSDS-GKDASSL-LLSRLYDTFASNLKS--SNATVRQNCFHTFSVLCKH----AK
S9W134_SCHCR        FADS-GKDASSL-LLTSLYEAFASNLKS--SNATVRQNCFQTFSVLCKH----AK
H0GUK0_SACCK        SPGK--INLLNVFTSSKLLNQSFSALKS--SKDVVRAISLHSVKILFRKISKSDT
E5R2E4_ARTGP        IPVD--IDLSDV-FSSSICKQLLANIKS--TNAIIRKGAIEALEKFIPR----CS
J8Q888_SACAR        SPYK--VNLLNVFISSKLLNQTFSAIKS--SKEVVRAISLHSVITLLKKVSKNDT
C5G992_AJEDR        IRFE--VDISEI-FFTRFCKPLLTNIKI--TNPIIRNGAVKAFESLVSR----CK
G4MR95_MAGO7        LPSD--YDLSSV-LSKNLLKPLLANAKS--TNASIRSGALTAFRKVVTR----SA
C9SQ16_VERA1        LPPS--HDLSQI-LSERLLKPILANVKS--SNPAIRSSTVAAFDLIASR----SH
B2WNI9_PYRTR        LPQS--LDLSDI-LLISMLKPLLSNVKS--TNPAIRAGALRTFKALASR----SQ
S3CN37_OPHP1        LPKN--YDLSTI-LSEHLAKPLLSNVKS--SKESVRNGALSAFRALAAR----CT
G3ALT0_SPAPN        FPK---IDISEPFVSSKLLTSIISGFKS--TKENVREGAFKVTELILLNN-FTDS
Q7S5P0_NEUCR        LPQD--FDLSAA-LHGKFVKPLLNNVKS--SNAVIRSGAVTVFKGLAAR----SK
A0A165FVX9_9PEZI    LPAD--VDLSDI-LEGNLLKALLSNVKS--SNPNIRAGVLSAFELLVSR----CH
A0A178DN93_9PLEO    LPVS--VDMSQI-LLGNLVKPLLANVKS--SNPSIRAGALRTFAALASR----SR
A0A177D7J2_ALTAL    LPET--MDLSDI-LLGNMLKPLLSNVKS--SNAAIRAGALRTFKALASR----SK
A0A0C4DS68_MAGP6    LPAQ--YDLSSI-LSNNLLKPLLSNTKS--TNPTIRSGALAAFKTIIPR----AS
A0A151N4J5_ALLMI    K-----LDLSQ--YAVDIVKGLASHLKS--NSPQLMDEAVVALKNLARQ----CS
A0A146NKU7_FUNHE    T-----LDLSQ--YAMDIGKAIASQLKA--NNAQLMEEAVKAMENLAQQ----CS
A0A0F7Z917_CROAD    T-----LDLSQ--YALDIVKGLASQLKS--NSPHLMDKAVVALKNLALQ----CS
A0A0W0DC37_CANGB    NAKT--INMAELYASTKLMSQAFSSFKS--SREGTKTAAISFTVSVLRSLCVETT
A0A0L8RIK7_SACEU    STEK--VNLLSVFTSSKLLSQSFSAFKS--SKEIVRSISLHSVITLLRKVSKSGT
A0A178FRD2_TRIVO    IPVE--IDLSEV-FSSSIYKQLLANIKS--TNAVIRKGAIEALEAFIPR----CS
A0A175WD14_9PEZI    LPQ---LDLSQA-LNGRFVKPLLSNIKS--SNPVIRSGAVSAFREIASN----SR
A0A194VFU1_9PEZI    LPED--WDLSNI-LHGQLLKPLLSNIQS--SNAAIRTGAVNVFRVIVGR----CH
A0A151TXV6_CAJCA    K-----LDMSK--YAAEILSVVLAQARH--ADEGRRDGALAIVRSLSQK----SS
A0A0M9ABE9_9HYME    S-----LDLSQ--YSQDISKGLFANLYS--KEDLVRDEAVGACRRLALQ----CS
A0A072U5F0_MEDTR    K-----LDLSK--YAAEILSVVLVQARH--ADEGRRDVALDIVKNLSQK----SS
A0A0K8V1F4_BACLA    N-----IDLSE--YASSLGKVLLHHLYC--KDDIVRGESLETLKELSLK----CT
A0A0A1XE05_BACCU    N-----IDLSE--YASSLGKVLLQHLYC--KDDIVRGESLEALKELSLK----CT
A0A131YTA4_RHIAP    T-----LELSP--YLGELGKSLAQHLVA--KDESLRQGAVVALRNLAHQ----CG
A0A0J6I2R9_COCPO    IPRE--IELSEV-VSTRLSKPLLSSFKS--ASQTVRQGAVKAFEALIAK----CK
A0A074XMM5_AURPU    LPKD--LDLSDI-LANNLLKPLVSSAKS--SNATIRAGALRTFSATARR----SH
A0A167FV98_9ASCO    LSPL--VDISSA-VENSLVNSFTSAFAS--SNVIVRDSAAECLRIAIQH----CY
A0A1A7XZM1_9TELE    T-----LDLSQ--YAMDIGKAIASQLKA--NNAQLMEEAVQAMQNLAKQ----CS
A0A0P7V1U6_9TELE    T-----LDLSQ--YALDIGKGLASQLKA--NNPQLMEQAVQALQHLAQQ----CS
A0A1A7WWE1_9TELE    T-----LDLSQ--YAMDIGKAIASQLKA--NNAQLMEEAVQAMQNLAKQ----CS
                             :
```

```
GCN1_YEAST          TLEDLTKLI-DEIFKNIK----SNLNADYKSLISKILIEIPLT------------ 495
GCN1_SCHPO          DNESLSRVA-SELITSLR--TGKVTASDQRVLFVDALSSLSL-------------
GCN1_DICDI          DTKLISSMITDDLLKTLS----VAGNPSQKLIIISIISSIISTKNFIERLSLTTE
GCN1_MOUSE          DSSATEALT-KHLFAILGGSEGKLTIIAQKMSVLSGIGSLSHHV-----VSGPS-
GCN1_HUMAN          DSSAMESLT-KHLFAILGGSEGKLTVVAQKMSVLSGIGSVSHHV-----VSGPS-
M7ANV9_CHEMY        DPSAVELLG-RHLFAILGGSEGKLTVVAQKISVLSGIGSFSHHV-----VSGSS-
H8X4E7_CANO9        DDIS------DEIIKAVK----MTTNAESKSLLIKSLL-----------------
W6A2P0_ICTPU        DPTAIQDLV-THLFGILGGSEGKLTVVGQKMNVLSGIKSCSHHA-----VSGFS-
B6JZI6_SCHJY        NQEVADHAV-EELLKVLR--TGK--------------------------------
R4WJK2_RIPPE        DPEAIWSLL-EHLFQVFNGSQGKITVADHKISILQGIGNFSYNA-----VTGSS-
W8CCK9_CERCA        TIESVEYLL-RGIFDILNGVNGKVTVTEYRINLIQGAGYLSFNS-----ISIQE-
B0W357_CULQU        GVKAIEALL-KEVFAVFNGSGGKITVVELRINLLQGAGNLSNNR-----ISSED-
E1ZX97_CAMFO        DTTALETLL-SSVFAVFHGSEGKLTVATHKISVLQGAGNLSYNV-----ASGSS-
W5JJ28_ANODA        GATAIEALL-KEVFAVLNGSGGKITVAEFRINLLQGAGNLSYNK-----ISTEK-
F1KPR4_ASCSU        DTSAVESLI-NAIFATYSGSDGKITTSAQRLTVLETLKGMSSHN-----AYGRES
V9K7F0_CALMI        DPAAVESLV-RHLFAVLGGSEGKLTTAAQKMSVLSGIGNCSYHT-----VTGAS-
W0T7Q3_KLUMA        DEKALEKLM-DELFKSLK----SNMNTDYKITISSILVNTPTY------------
S9R951_SCHOY        DSKVLMEVA-LKLTSALK--TNKVTSNEHRNLYMECLSHLSV-------------
S9W134_SCHCR        DSKMLMDVA-LKLVSALK--TNKVTSNEHRNLYIECLSHLSV-------------
H0GUK0_SACCK        NSEDLMKVV-DEIFRNIK----SNLNADYKSMISKILIEIPLT------------
E5R2E4_ARTGP        DERSLLRVV-DEVVTPLK--TQKITNVEQRALQARVLQVIPS-------------
J8Q888_SACAR        QLEDLMKVI-DEIFKNIK----SNLNADYKSMVSIILIEIPLT------------
C5G992_AJEDR        AEQWLLKVV-EEVMAPLK--TQKITNAEQRGLQVQVLSAIPP-------------
G4MR95_MAGO7        DAETLGKAT-DEIVTPLK--GGKIASAEQRAIHAEMLTALST-------------
C9SQ16_VERA1        DDKALEKAC-DEVVTPLK--GGKLASADHRILHCEMLLALPQ-------------
B2WNI9_PYRTR        DGEKVDKVA-DELLTPLK--QGKVSGADQKILHAQMLAVLPQ-------------
S3CN37_OPHP1        DLDAVAKVI-EETLTPLQ--TSKLTTPEHRIIHAQILDALPK-------------
G3ALT0_SPAPN        SIETLGKFV-DEILKALK----STSNADSKALIVKTLSYL---------------
Q7S5P0_NEUCR        DVALLEKVA-DEVLNPLK--TGKLASADHRVLHSEMLVALVV-------------
A0A165FVX9_9PEZI    EEKCLVAVT-NEILGPLK--SSKLPSAEHRVLHAQMISTIPT-------------
A0A178DN93_9PLEO    DEGVISKIA-DEVLNPLK--QGKVSGVDQKVLHAQMLAALPS-------------
A0A177D7J2_ALTAL    DDAKIDKVA-DEVLTPLK--QGKVSGVDQKVLHAQMLSALPE-------------
A0A0C4DS68_MAGP6    DLDALGKAT-DEIAAPLK--GGKVASADQRAIHSEMLMELAK-------------
A0A151N4J5_ALLMI    DPSAVESLG-RHLFAILGGSEGKLTVVAQKMSVLSGIGSLSHHA-----VSGPS-
A0A146NKU7_FUNHE    DATAVQDIV-THLFKILGGSEGKLTVVAQKMNVLSGIGSCSRHA-----ASGTS-
A0A0F7Z917_CROAD    EPSTVESFG-KHLFAILGGAEGKLTVVAQKISILSGIGSCSHHA-----VSGAS-
A0A0W0DC37_CANGB    EESALLKIV-ENIFTNLK----TNMNADYKAIVSTLLQAIPTF------------
A0A0L8RIK7_SACEU    NSEDLMKVV-DEIFKNIK----SNLNADYKSMISKILIEIPLT------------
A0A178FRD2_TRIVO    DEKSLLKVV-DEVVTPLK--AQKIPNVEQRALQAQVLRVVPC-------------
A0A175WD14_9PEZI    DFALLEQVA-DEVLGPLK--SGKLASADHRVLHSEMLAALPT-------------
A0A194VFU1_9PEZI    EKAVLEKIA-DEILTPLK--AGKLVSADHRVLHCQMLLSMSL-------------
A0A151TXV6_CAJCA    NPDALDTMF-SAIKSVIKGSEGRLAFPYQRVGMVNVIQELSN-------APDGKY
A0A0M9ABE9_9HYME    DTVALENLL-SSVFAVFHGSEGKLTVATHKISVLQGAGNLSYNA-----ASGSS-
A0A072U5F0_MEDTR    NPDALDIMF-NAIKSVIKGSEGRLAFPYQRVGMVNAIQELSN-------APDGKY
A0A0K8V1F4_BACLA    TIDSVEYLL-KGIFDTLNGRNGKITVAEYRIHLIQGAGYLSCNG-----ISDKE-
A0A0A1XE05_BACCU    TVKSVEYLL-KGIFGSLNGDNGKITVAEYRINLIQGAGYLSFNA-----ISYKE-
A0A131YTA4_RHIAP    SQEALEALI-KHLVGVLNGSEGKLTTTEQRLSVLTAIGEVSCHV-----VTGASH
A0A0J6I2R9_COCPO    EEQWLLKII-DEVILPLK--TSRITNVEHRALQVQVLSIFHC-------------
A0A074XMM5_AURPU    DDKLIEKVL-GEILTPLK--TGKVPSADQKIIFAQMLAALNS-------------
A0A167FV98_9ASCO    DQEAINRIS-LTLANNLK----KATSPDHRILYGSVLADNI--------------
A0A1A7XZM1_9TELE    DPSAVQDIV-THLFKILSGSEGKLTVVAQKMSVLSGVASCSHHA-----VSGTS-
A0A0P7V1U6_9TELE    DPTAVQDII-THLFNILGGSEGKLTVVAQKMSVLSGIGSCSHHA-----LSGAS-
A0A1A7WWE1_9TELE    DPSAVQDIV-THLFKILSGSEGKLTVVAQKMSVLSGVASCSHHA-----VSGTS-
```

```
GCN1_YEAST            HYEVSEKICKGLSPYIGKEGNEAALTLMLNAFFVHY-FSL---GKPIEDLDKIIS 550
GCN1_SCHPO            KHIDASMLLNELLPLFTK-AKESDFNSLASLIVKTLKFLLMNGRNPGDKIYDFLS
GCN1_DICDI            KLQLSKQILQSISIYLEKELNKDNRNKGFKLLGK----VMKMVEELPEQTIKIIT
GCN1_MOUSE            GQVLNGCVAELFIPFLQQEVHEGTLVHAVSILAL---WCNRFTTEVPKKLTDWFK
GCN1_HUMAN            SQVLNGIVAELFIPFLQQEVHEGTLVHAVSVLAL---WCNRFTMEVPKKLTEWFK
M7ANV9_CHEMY          SQALSGTMAELFIPFLQQEG-----------------------------------
H8X4E7_CANO9          KSTKSEKVLDSLLPLASKEQNETSLGSLVDVFAYHA----FKANKFDDNIAKTFI
W6A2P0_ICTPU          SQSLSSSVALQFIPFLQQEVHEGTLVHAVSVMSL---WMARLNVEVPAALRDWLK
B6JZI6_SCHJY          -----------LIPLFQKEQAEP-LKVLGAVLMKNLETGLLNNVLPDETVLKSLR
R4WJK2_RIPPE          IQPLAKNAVEHFIKILETEVHEKTLVHCLEMMSL---WCSKFVTEVPTTVLSWLE
W8CCK9_CERCA          ESVILQLAVDFFSKALITEIQEKVICHTLDMFSL---WMVKFVGELPKSIVDIFK
B0W357_CULQU          IQSLMPLVTDLFTKALETEQQEKVLCHALEMFGF---WSVNFRGEIPAKIVQTFK
E1ZX97_CAMFO          VQKLAETACEHFVKVLETEVHEKTLIQALEMMAL---WSKKFSSTLPKIVVDAFK
W5JJ28_ANODA          IQTILPSVCDQFAKVIESEIQEKVVCHALEMFGL---WTENYRGEISPKIVQLFK
F1KPR4_ASCSU          CELLGNAVISRLAPLIPPEVHDATLAAMWDAMVV---WGERM-PSISAHLLPLFK
V9K7F0_CALMI          SQTLSGTVTELFIHFLQQEVHEGTLLHTISVLTL---WCNKFTTEVPKHLLEWFK
W0T7Q3_KLUMA          SPQVSNIILNGLKNYISKESNEVALEKMLSAFFTHF-FSS---ESADPATISTIK
S9R951_SCHOY          KEIDSTSLLKEVLPLFMK-VQESSFYHLAAVIIRNIKSILLSNSSLDKGIYDFLS
S9W134_SCHCR          KEVDSASLLNEVLPLFMK-VQESSFYHLAAVVTRNVKSLLSSNISLSRSIYDFLS
H0GUK0_SACCK          HREVSEKICKNLCPYIGKEGNEVALTSMLNAFFIHY-FSL---EGPVEDLNKIIS
E5R2E4_ARTGP          SPSASQAILARLSAALLRESSEIALEPEIKSFCHHLTFLVSSGADVSKEHCNTVI
J8Q888_SACAR          HREVSEKICKGLSPYIGKEGNEIALTYMLHAFFIHY-FSL---AEPTEDLNKIIS
C5G992_AJEDR          STHVSQLVLGGLVVVLSREASEIALEIEIKAFCCHFAYLIRIAAPIGKEILSAIT
G4MR95_MAGO7          NEANATKICGAVPAVAGKEGNEPALVAETKALLCSVKTLLNSGVEVPAAALDGYK
C9SQ16_VERA1          SDTVAGKIATALATVTAKEGNEAAVAAETAALARSTSHLLQSDLDVPKPVLDAFI
B2WNI9_PYRTR          STSLSAKIPAGIAPVALKEPSEPAVLAEVSALTTHLNFGLANGVSVDKTVSDAFI
S3CN37_OPHP1          SDASANKVASGVAIAAAKEGNEAALTAEVATLTKSVKILLQSSTDVPKPVSDAFV
G3ALT0_SPAPN          PSKVSGKIIKSLLPLVSKDINETSLSSLVKTFIQHA-FKVLDDNDLSSQITKELT
Q7S5P0_NEUCR          STGIANKIANGLPLVTGKEGNEAALSAETVALNASAVYLLNAGEEIPKPLADAYV
A0A165FVX9_9PEZI      CQALAETVPATLAPVATKEPNEAAISAEAAAISKHFRYALSEQLKVERSVTDVFI
A0A178DN93_9PLEO      LPSLSKTIPAAIAPVALKESNEPVVVAEVSAMAKHLTFGLENGVALDKTVTDAFL
A0A177D7J2_ALTAL      SISLSKKIPAGIAPVALKEPSEPAVVAEVSAMTTHLNFGLANGVSLDKTVSDAFI
A0A0C4DS68_MAGP6      TEVNATKICLALPAVAGKEGNEPALAAETSALTEAAKTLLKSGAEVPAAAIDAFK
A0A151N4J5_ALLMI      SQALSGTMAELFIPFLQQEVHEGTLVHAVSVLAL---WCNRFTTEVPRNLVEWLK
A0A146NKU7_FUNHE      SQTLSSAVTVMFIPYLQQEVHEGTLVHAVSVLSQ---WSSRLTVEVPAALLDWFK
A0A0F7Z917_CROAD      NQVLSGSMVELFIRFLQQEVHEGTLVHAISMLAL---WCGRFLTEVPQTLVEWFK
A0A0W0DC37_CANGB      HENVSLDIAEKLSPLLVKESNEQALVNLLNPFYQHY-FAT---GTSIDKFNTTII
A0A0L8RIK7_SACEU      HREVSEKICKGLFPYISKESNETALALMLNAFFIHY-FSL---AESIEEFNKIVS
A0A178FRD2_TRIVO      SSSASQAILTGLSAALLRESSEVALEPEIKSFCHHLTFLVSSRADVSMENCTTVI
A0A175WD14_9PEZI      SAGIAAKVASGLPPIVGKEANEAALSAETLALNSSALSLLASG-DAPKPLLDAYA
A0A194VFU1_9PEZI      SGGIAVKAAAALPTPIGKEGNEVALKAETLGLSKAVKSLLTEGTELPKAVTDAYA
A0A151TXV6_CAJCA      LISLSRTICDFLLSCYKDDGNEEVKIVILSAIAS---WAVKSTDIIQESLVSFLV
A0A0M9ABE9_9HYME      VERLAETACEHFIKVLETEVHEKTLIHALEMMAL---WSNKFANNVPKCVIDAFK
A0A072U5F0_MEDTR      LINLSQTICDFLLSCYKDDGNEEVKIATLSAIAS---WADKSTNIIQESLVSFFA
A0A0K8V1F4_BACLA      TPIVLHLAMELFSKALQTEIQERVICCTLDMFSL---WMLKFIGELPKSIIDIFK
A0A0A1XE05_BACCU      TPIVLLLAIELFSKALVTEIQERVISCTLDMFSL---WMLKLSGELPKSITDVFK
A0A131YTA4_RHIAP      IQRLSEIALQHLLAVLKAEVHEGTLLLTLKMMSK---WCSRFTQEPPSFLVTGFK
A0A0J6I2R9_COCPO      FPELSHRVLSGLSPALARESNEAALESEVQAFCHHLAYLIRSQSTISKEDFSVVA
A0A074XMM5_AURPU      SEALAASIPQGLAPVAAKEPNEAAASAEIAALSKHLKFGLEKGVAVNAAVTDAYV
A0A167FV98_9ASCO      KSNNGASLAAIILPVATKESNELALTQLTRALFYHF----IRGDNYDKSIIEGIK
A0A1A7XZM1_9TELE      SQTLSSAVAVMFIPYLQQEVHEGTLVHAVSVLSQ---WSSRLTVEVPTALLDWLK
A0A0P7V1U6_9TELE      SQALSSSVTVLFIPYLQQEVHEGTLVHAVGVLSQ---WSGKLTVEVPRPLLDWLK
A0A1A7WWE1_9TELE      SQTLSSAVAVMFIPYLQQEVHEGTLVHAVSVLSQ---WSSRLTVEVPTALLDWLK
                                  .
```

```
GCN1_YEAST          AGFADK--KPALKKCWFAAFL---------------NNSNAASEEVILNFIDGCL 605
GCN1_SCHPO          KSLQRP--VAHESMFWLTSLATMAWDLPASD-----DVQIEFINFFLNNLSILTE
GCN1_DICDI          NSLKND--DDIIKGQVILSLSKSLGPEANGTNKKVIQIINGFTETINTILKNVKN
GCN1_MOUSE          KVFSLKTSTSAVRHAYLQCMLASFRG-----------DTLLQALDFLPLLMQTVE
GCN1_HUMAN          KAFSLKTSTSAVRHAYLQCMLASYRG-----------DTLLQALDLLPLLIQTVE
M7ANV9_CHEMY        -------------------------------------DTLLQAMDLLPMLIQTVE
H8X4E7_CANO9        TGFKSK-----VKRVWFVEYGK--------------HALGKEQPDFTTVFNDTLS
W6A2P0_ICTPU        KAFTLKSSTSSVRHAYLQAMLRAFKG-----------DTLSQALDLVPLLIQTVE
B6JZI6_SCHJY        SFLKDA--RAPIREYWILSIARLLWNFETFS-----AAQTSVLELFLHELLDLCK
R4WJK2_RIPPE        KGFALKTSTCPVRTAYIGVMSACCQS-----------GTAGQITQFIPILLKSVE
W8CCK9_CERCA        RGLDLKTTTQPVRVAYLQWFLAGLQN-----------AKLYQGFNFSPLLLSMVE
B0W357_CULQU        KGLEAK--AQVLRTSYLQWFLACLEN-----------GKLPSGTDYTAALGKIVE
E1ZX97_CAMFO        KGMAAKTSTAAVRTAYIKLFFST--------------PTAAYSTIIAPILAQAIT
W5JJ28_ANODA        KGLEAK--AQPIRTSYLQWFLSCLHY-----------GKLPGGSDFTLALSKVVE
F1KPR4_ASCSU        ASHKSTASRSIMIRAMVHLFCKC------------------GFEKFDSDLITTME
V9K7F0_CALMI        KAFTLKTSTSAIRHAYLQCMLASFRG-----------DTLLQALDLLPLLLQAVD
W0T7Q3_KLUMA        AGFNEK--KLPLKKIWFNSFLIHSN-----------SIKLDIADSFEEELIQHLK
S9R951_SCHOY        KSLDKP--VAYERQHWILALADLVWDSQTNE-----SSQLELNNFYLEHLNKLSQ
S9W134_SCHCR        KSLDKP--VAYERQHWILALADLAWDLRADE-----PSRLEFLNFYLEHLNKSSK
H0GUK0_SACCK        AGFTDK--KPPLKKCWFATFL---------------NNSDSASEEVFLNFLDGCF
E5R2E4_ARTGP        KGCDDK--RTGFRKTWLVNIGEVFWNCDSSVLQSSQTFSKECLQPALIRLHKSFT
J8Q888_SACAR        AGFTDK--KPALKKCWFATFL---------------NNSNAASEEVILNFVDGCL
C5G992_AJEDR        KGCGEK--RIALRKLWLVNIGQLLWDMDVEQLFG-SSLTMDFLRPVVARLLESSD
G4MR95_MAGO7        KGLGDK--KPGSRKIWVLSAGDLLLH-SCSLPDS--KIAPKVQEVILPVLFDTYN
C9SQ16_VERA1        KGAADK--KPALRRIWILRAGEVIGDITKVQSTS--SSTLKFAEGILPKLVDAHN
B2WNI9_PYRTR        KGMADR--RIPVKRLWALRAGDIWWNLSQEQQAR--PDVLAFCQTTLPKLVEIWQ
S3CN37_OPHP1        KGLADK--KAPARRIWIISAGEALYDFH-NASEHLPSSVTKFAEAVLPPLLTVYS
G3ALT0_SPAPN        AGLSHA--KQQLRRIWFIEFGEQIFTNNN-------ESEVKLLNNLFPTLVKSLE
Q7S5P0_NEUCR        KGLGEK--KLPVRRTWILRAGDILYAFNKESQETLPANFIKFAEAVVTPLGTTFA
A0A165FVX9_9PEZI    KGIGDK--RGSVRRGWTLRVGEIIWSLREHDFGN--TETISFLQATLPKMLDIFK
A0A178DN93_9PLEO    KGMTDK--RVPVRRLWAINAVDIWWNLSDAQYTQ--PDILAFCHATLPKLVEIWQ
A0A177D7J2_ALTAL    KGMGDK--RVPVKRLWAIRAADIWWGLSDEQQSQ--PDVLAFCQTTLPKLIEIWQ
A0A0C4DS68_MAGP6    KGLADK--KPTSRKLWILAAGELLLDVSQTTPLS--ASALKVTEGIMPALFETYN
A0A151N4J5_ALLMI    KAVSLKTSTSPVRQAYLQCMLASFKG-----------DTLLQGMDLLPMLIQTVE
A0A146NKU7_FUNHE    KAFTLKTSTSAVRHAYLQTMLGAFKG-----------DTLAQSSDLVPLLLQTIE
A0A0F7Z917_CROAD    KAFSLKACTSAVRHAYLQCMLASFKG-----------NVLLQGAELLPLLVQTVE
A0A0W0DC37_CANGB    SGLQEK--KAPLKRCWFSSLF---------------MNIDTVTESIAKTIGKLCT
A0A0L8RIK7_SACEU    AGFTDK--KPALKKCWFASFL---------------NNSNTASEQVLLDFIDGCL
A0A178FRD2_TRIVO    KGCDDK--RAGFRKTWLMNMGELFWNCDNSALQSSQTFSKECLHPVLTRLHKSFT
A0A175WD14_9PEZI    KGLADK--KIPVRRIWMFRVGDILSAFAEDTQAAIPPGFGVFAEAVLPPLLSTFN
A0A194VFU1_9PEZI    KGLADK--KLSARRVWTLSVGESLDSLSSDDSPS--SLFVKFAESVFPPLFDTYS
A0A151TXV6_CAJCA    SGLKEK---ETLRKGFLRSLHTICKN----------EDAVIKMLPLFGPLVQLVK
A0A0M9ABE9_9HYME    KGMTAKTSTAAVRTAYIKLFFST--------------PVVSYSEVITPLLIQAIS
A0A072U5F0_MEDTR    SGLKEK---EILRRGFLRSLRAICKN----------ADAVLKMSPLLVPLVQLVK
A0A0K8V1F4_BACLA    RGLDLKTTTQTVRISYLQWFLAGLNN-----------AKLHHDLNLTPLLIKIVE
A0A0A1XE05_BACCU    RGLDLKTTTQTVRISYLQWFLAGLNN-----------ANLRHDLNITSILIKIVE
A0A131YTA4_RHIAP    EGMEQKTATSAVRYGYLQCMISAFHG----------MPGCKQVEPILGLVLKAVE
A0A0J6I2R9_COCPO    KGCAEK--RSAFRKIWISNVGEVIWNLDRDSLFSSSSVKTNFLKPVVEKISSSFD
A0A074XMM5_AURPU    KGVAEK--RLPFRKLWTLSIAEIVWSLEGEVLKQ--PEVAGLVDAVVGKMIDSFN
A0A167FV98_9ASCO    KGFTEK--RLTIRRVWITEFVKRVSNEVKS------DSLVNFISSFDIQLDAAFK
A0A1A7XZM1_9TELE    KAFTLKTSTSLVRHAYLQAMLGAFKG-----------DTLSHASDFIPLLLQTTE
A0A0P7V1U6_9TELE    KAFTLKVSTSPVRHAYLQAMLGAFKG-----------DTLHQAMELLPLLLQTVE
A0A1A7WWE1_9TELE    KAFTLKTSTSLVRHAYLQAMLGAFKG-----------DTLSHASDFIPLLLQTTE
                                                                 .
```

```
GCN1_YEAST          EFVKDSIIHYQTHGHA---CILASIEFT-------------------------- 660
GCN1_SCHPO          KALMSVSGATQNGTYLAPIIYLSFGVNKLSVWNSERISHTLELQ--DILVKLSTP
GCN1_DICDI          AKTCDPSTTTASLHYMLSLITTTGVPKNDIFTKYSTDKTTI-----------SNL
GCN1_MOUSE          KAASQG---TQVPTVTEGVAAALLLSKLSVADAQAEAKLSGFWQ--------LVV
GCN1_HUMAN          KAASQS---TQVPTITEGVAAALLLLKLSVADSQAEAKLSSFWQ--------LIV
M7ANV9_CHEMY        KSASQS---TQVSMVTEGVASALLICRMSLADAL---------------------
H8X4E7_CANO9        EIEKSPAANIKN--IAAAFVILALTH----------------------------
W6A2P0_ICTPU        KAAAQS---SQHALLSEALAASVLLCHLSILDSVPESKLTSFWN--------LIL
B6JZI6_SCHJY        EALKNLSNAAQSGSIMAPFVLLAFVFSHVPKTAPEVAESEQFKHIQAFLRNALNP
R4WJK2_RIPPE        KAAAQS---SQVPVVTEGLSAACLLLKM-STYTVVDSKLSFLYN--------IIL
W8CCK9_CERCA        KGLQSP---IQIPLLCEAVCAAGVIMQ--TESTLNNPILGNFWN--------TMF
B0W357_CULQU        KAAQNP---TQTPAVSEGLGAACIIL---LTNAAVSDALKDFWN--------VVL
E1ZX97_CAMFO        RTMQQC---TQPAAVTEGLVASYILLKFVLADQIENDKQNVLWN-----------
W5JJ28_ANODA        KAAQNP---TQTPLVSEGVGAACIIL---LTNRTVEDKLKDFWN--------VVL
F1KPR4_ASCSU        NVEKSN---SASAGEWVGASLLLLSCDNDEVRQRVRKAV----------------
V9K7F0_CALMI        KVAAQS---SQVAIVSEGVAAAVLICRLSVVEAQIEPKLATFWQ--------LVL
W0T7Q3_KLUMA        DIVSNPLRNGEV-------STLGCFVYI--------------------------
S9R951_SCHOY        ATLNNPTTALQNGSILAPVVYLCLMSKKESDGDISKLKFTGDLQ--TLLDGLVNS
S9W134_SCHCR        AALSNPTNALQNGSILAPVVYSCLMNKERSDRELTELKSVDDLQ--SLLDGLVSS
H0GUK0_SACCK        EFAKDSIMHYQTHGHT---CILASIQFI--------------------------
E5R2E4_ARTGP        ETVANPIPSLQGGTISIANVFTALSLQK------FRSEDRLPYPVEDIFPIAVSA
J8Q888_SACAR        EFVKDSIMHYQTHGHT---CILASIEFI--------------------------
C5G992_AJEDR        DIAANPLPSVQIGAITISFVLTALSSR---LENRKDNEGELLLAYDNIARQALSI
G4MR95_MAGO7        EAVANPLAASQSGLIVGAYVTCTVAPSI--FQTNKAAPIEALLKKFSVSKQCLVW
C9SQ16_VERA1        EVVANPQLAAQNNATIGAYVVVALLCSA--SGSKLFGAVKLPTPRASILKQAMTL
B2WNI9_PYRTR        EVNANPLPAAQSGLVTVGHYVTALLIGK--VQNANDEKLVSIFKKSDVISQSLAT
S3CN37_OPHP1        DVVANPSTASQNGLVTGGLVVCGTAPFL--LKDASGA-LADLAKTFSLQKQCLVL
G3ALT0_SPAPN        PAQQSPLPTLANKGIVCAFVTIAVSKYV----------------EVDAGKIYIPS
Q7S5P0_NEUCR        EVVANPATAAQNGTVTGALVLCAVESLI--RRAESST-FEAVYKKANIQKNSLAV
A0A165FVX9_9PEZI    EVVANPLSAAQNGLITIAYVVTALYASK--LRLIKDGALSDLFEKASIVKQSLAL
A0A178DN93_9PLEO    EVIANPVPATQSGLVTVGHYVSALLLDK--VRNLKDEKFAAIYKKSDVLSQSLAV
A0A177D7J2_ALTAL    EVNANPLPATQSGLVAVGHYATALLIDR--VQNMKDEKLGSIHKKSDVISQSLAT
A0A0C4DS68_MAGP6    DAVANPLTSSQSGLITGAYITCSIAPLV--FGGNTSGPLEALSKKFSIAKHCLVT
A0A151N4J5_ALLMI    KAASQS---TQVSLVTEGVAASLLICKMSVVEAQIDAKLSGFWQ--------LIL
A0A146NKU7_FUNHE    KAAAQI---SQHALLAEGVAASVLLSRLALLETQTEAKFSSFWN--------LIL
A0A0F7Z917_CROAD    KAGAQS---TQIPLVTEGVAAALLICRLSVADAQIENKLNSFWQ--------LIL
A0A0W0DC37_CANGB    TYIKENLVHAQRTEHSVAFSILSGFVFF--------------------------
A0A0L8RIK7_SACEU    EFVKDSITHYQTHGHT---SILGTIEFI--------------------------
A0A178FRD2_TRIVO    EISANPIPSLQGGTISTANVLTALSLEK------FWNEDNLPYPIEDIFTTALSI
A0A175WD14_9PEZI    EVVTNPVAAAQNGLVTGGLVVCGLGPL---FHRLESDSLQASLKKASIQSNSLVV
A0A194VFU1_9PEZI    DVLSNPVKASQDGTIISAYVVCAVAPFL--LKDESNQKLSALSKKFSVSKQCLEL
A0A151TXV6_CAJCA    TGF------TKAVQRLDGMYALLLVAKIAAVDIKAEDTLVKEKI-------WALI
A0A0M9ABE9_9HYME    RAMQQS---AQPVAVTEGLVASYLLLKFVLANQVENDKQTVLWH-----------
A0A072U5F0_MEDTR    TGF------TKAVQRLDGIYALLLVGKIAAVDIKAEEILVKEKI-------WATI
A0A0K8V1F4_BACLA    KGLQSP---LQISLVCESVCATCIIIQ--TITITNDPLFGSFWN--------TVF
A0A0A1XE05_BACCU    KGLQSP---LQISLVCESVCAACIIIQ--TNTISSDPILGSFWS--------TMF
A0A131YTA4_RHIAP    RALAQP---LQPGPVCEGLAAACLLFRLQPLGSPSENKIKSMVT--------QLL
A0A0J6I2R9_COCPO    EINANPLPAVQGGTISIAYVLLALSCQN--LQGGKLDDGSLLLNTDVIVGQSLSL
A0A074XMM5_AURPU    EVVSNPVVATQNGQVTIACAVTALSLAK--VPQLAELKNASVLKKASILDRAIAL
A0A167FV98_9ASCO    EVASNPTNAIQNKAVAIGFALLTLKPIS-------------------------YA
A0A1A7XZM1_9TELE    KAAAQI---SQHALLAEGVAAAVVLSRLALLETQTEAKFSSFWS--------LIL
A0A0P7V1U6_9TELE    RAAVQN---SQQALVSEGVAAAVLLCRLSLLDTQTDAKMTTFWN--------LIL
A0A1A7WWE1_9TELE    KAAAQI---SQHALLAEAFIFSV------------ATMYRNPW------------
```

```
GCN1_YEAST          -NKILALDNTELNDRVMQLIETL--------------------PENSSIGDAILT 715
GCN1_SCHPO          KNNDVFIFSSKITNKLNDDQSKLWYFQGLCDFAKVSD-NLLFSNFVERWFQSVIG
GCN1_DICDI          YASTSFLHTDGFIQRTSKKDHAIDLLLTLFLRVKSFPSIKLNDKSPLY--SSVLN
GCN1_MOUSE          DEKRQTFTSEKFLLLASEDALCTVLRLTERLFLDHPHRLT-NSKVQQYY-RVLVA
GCN1_HUMAN          DEKKQVFTSEKFLVMASEDALCTVLHLTERLFLDHPHRLT-GNKVQQYH-RALVA
M7ANV9_CHEMY        ------------------TAMCTVLQLTERLLLDHAHRLP-ENKVQQYH-RALVA
H8X4E7_CANO9        -RDSPLLDETKLFEKL-DTIDLLWFVKDLY-----------------NHPKGWLY
W6A2P0_ICTPU        DEKKPLFTTEKFLVQTSEEALCTVLQLCERLFLDHPQRLN-NNKAQMYH-RAMVA
B6JZI6_SCHJY        KSKEMLLFTQRNINRLLDAQQKQWAIIAACIYITEFA-ESLDNSAYADLFYCLVF
R4WJK2_RIPPE        DMEKQVFISERFLSQASCEALCQVMNLCEKILMEDTIGGSVSTVRGPVH-RALVT
W8CCK9_CERCA        DMNKQIFFSERFMVAAPPETLCYVSLIAERLLTKYSDNLK--GSFNIVL-KALVS
B0W357_CULQU        DMNKKVFLGDKFLANTAGETLCYVMLMCEKLLLNHLDNVK--GEVEALY-KAVVY
E1ZX97_CAMFO        AIDEQIFFSEKFLSTCGDDVLYHLMLLCERLITEFFDRLN-EKALTGIH-RAVVS
W5JJ28_ANODA        DMSRPVFLGEKFLATTNAETLCYVMVICEQLLLHHRSELKGGGSTDPLF-RAAIV
F1KPR4_ASCSU        -IADDIIYRDKFITSLRKPDAMLLPQLSVKLITDRPYSNTEESVYSSVALRLLFI
V9K7F0_CALMI        DEKKQFFTSEKFLSVASGEALCTILQLTERLLTDLP-----EIKGQMYH-RALVA
W0T7Q3_KLUMA        -QRLFELEAEKTLSSIQSIID----------------------ENSESYGLMWAY
S9R951_SCHOY        -TGESILFSNKYSNKLTTDQAKLWWIRGLCTFASVCD-KVLTDDLASSWFQSLIN
S9W134_SCHCR        VNGDTILFSNKIINKLTTDSAKLWWLRGLCNFASVYA-KDLTDSLASSWFQSIIN
H0GUK0_SACCK        -NKILTLNNTQLNGRAIQLIDAL--------------------PENTTIGDAILT
E5R2E4_ARTGP        SPKLSFLLNPKIYTKLSSAEDLEWTLRALFAVTVQPGFSASDEQAKEAWAQTFIY
J8Q888_SACAR        -NKILTLNNTELNDRVMQLIDNL--------------------PENTSIGDAILT
C5G992_AJEDR        SPKPSFLLNPKIYTKLTSTEDFIWIIRALSTVSRSKAFHAGDPEIRDSWGQAFIY
G4MR95_MAGO7        EPKPSFLLNPKLFSKLSTDEDFEWFFRALASVVAELP-EDGDSDVSIAWAQALIY
C9SQ16_VERA1        DPKPSFLLNPRVYGKIATEDELTWMSRALASAASGLT-VATPSDVKTAWSEAYIY
B2WNI9_PYRTR        QPKPSFLLNQKVYSKVSTEEDVSIAMRALAAIAPSVA-DTSSGDVANAWAQAVIF
S3CN37_OPHP1        EPKPSFLLSPRIYTKLTADDDIRWLYRALVAAFPGLN-GNAESAVSIAWANAYVY
G3ALT0_SPAPN        LKAPGLLTSSKIYTKLTEAAELSWYLRAIAQASGSI--TELEEQERVDYGLAWVY
Q7S5P0_NEUCR        NPKPSYLFNPRIYGKFA-DEDLKWLTSALTAVAPFLN-GVGLPE-RVAWAQAFIY
A0A165FVX9_9PEZI    EPKPSFLLYHRIYTKLASEEDTIWAIRSLAALYDDLPSTKRENSTLQSWALAFIY
A0A178DN93_9PLEO    QPKPSFLLNSKVFTKLSTEDDVVIALRALSSVAPFLA--QTSAEAEDAWAQSFIF
A0A177D7J2_ALTAL    QPKPSIHLNPKVYSKLSSEEDVSIALRALKALAPEVT--QASKEVADAWAQAFIF
A0A0C4DS68_MAGP6    EPKPSFLLTPRLYSKLASNDDLRWLTRALVTVVNDLP-DDGSSSVCLAWSQALIY
A0A151N4J5_ALLMI    DEKKQIFTSEKFLQSATEEAMHTVLQLTERLLLDHAQRLS-ENKVQLYY-RALVA
A0A146NKU7_FUNHE    DEKKPLFTTEKFLSQGNEETLLAVLQLCERLFLDHAHRLN-ASKSQMYH-YATVA
A0A0F7Z917_CROAD    DEKKQIFTSEKFLQSASEEVMCTVLQLTERLLLDHEYRLP-GAKIQQYY-KALTA
A0A0W0DC37_CANGB    --------SENVYAEIEHDIIDI--------------------QKDAMLGEAFLR
A0A0L8RIK7_SACEU    -NKILTLNNTELHDRIMQLIEAL--------------------PAETCIGDAILT
A0A178FRD2_TRIVO    SPKPSFLLNPKIYTKLSSADELEWALRALFAVTVQPGFATSDEQAQEAWAQAFIY
A0A175WD14_9PEZI    EPKPSFLLNQRIYSKYV-DDDLKWFCRALSAVVPAL--GASGEAARVAWSQAYIY
A0A194VFU1_9PEZI    EPKPSFLLNPRVYAKVSSDDDARWLYRALSAVAPSLP-ADDTSKVSSAWAHVFIY
A0A151TXV6_CAJCA    SQNEPSVVPISMASKLSLEDSMACVDLLEVLLSEHLQRTLSNFSVRLLL-QLMIF
A0A0M9ABE9_9HYME    AIDEQIFFSEKFLSTCGDDVLYHLMLLCKRLIIEFGDKLN-EKTLNGVH-RAIVT
A0A072U5F0_MEDTR    SQNEPSLIPISMASKLAVEDSIACIDLLEVLLLEHLQRTLSNFSVTSLL-QLVIF
A0A0K8V1F4_BACLA    DVNKLIFFSERFMAAAPPETLCYVSLMAEKLLTQYSDKLK--GSLSVLF-KAIVY
A0A0A1XE05_BACCU    DMNKQIFFSERFMLAAPPETLCYVSLMAEILLTQYSDKLK--GPLNVLF-KAIVC
A0A131YTA4_RHIAP    DQEKQPFFADKFLQTASEDTHLLVLQLITRMILDHPDSCF--ANLKPFA-VPLLR
A0A0J6I2R9_COCPO    SLKQSFLLNPRIFTKLASKDELAWNIRALSGVSSEAAFQAVDPTVRDTWAQAFIY
A0A074XMM5_AURPU    EPKPSFLLNTRVYTKLTSHEDLVWLTRALAATSSGI--ASSQPEIQDAWAQTFIY
A0A167FV98_9ASCO    DEGASLLTSPRVYQKLISDQEFEWAILA----------TKSTDQFTLELGRAWIF
A0A1A7XZM1_9TELE    DEKKPLFTTEKFLSQASEETLLTVLLLCERLFLDHAHRLN-TSKSQMYH-HAIVA
A0A0P7V1U6_9TELE    DEKKLLFTTEKFLSQANEESLLTVLQLCERLFLDHAHRLS-NSKSQMYH-HATVT
A0A1A7WWE1_9TELE    --ISNYLTHVKFCSQGPG---------------------------------SEAS
```

```
GCN1_YEAST          AALSTELSIENRIHAVNLLQELFYK-KPEF-------IGFSVIDAIERRMRV--- 770
GCN1_SCHPO          VFSFAS--RENSNRALKILKSAILY-RPH--------LRMSICSQLWNYH-----
GCN1_DICDI          CLLHSQ--WSVSKHSAIKIRSILSRNDSVDID--YPLLSNQLLIEFSTILF----
GCN1_MOUSE          VLLSRT--WHVRRQAQQTVRKLLSSLGGVK-------LANGLLDELKTVLNSHKV
GCN1_HUMAN          VLLSRT--WHVRRQAQQTVRKLLSSLGGFK-------LAHGLLEELKTVLSSHKV
M7ANV9_CHEMY        VLLSRN--WHVRRHAQKTVRKLLSSLGGYK-------LAYGILEELKTVLKSHKV
H8X4E7_CANO9        VLTGKKVPYSVKKVAQERFREEVEV-NLS--------LSDGVIAEAYTLDPE---
W6A2P0_ICTPU        VLLCRA--YQVRKRAHQSVKKLLSSLGSPS-------LAHGLLGELCVVIHKHKV
B6JZI6_SCHJY        TLQAKS---NDKESCFSALKELSRT-CPE--------ARVKLSEQMWDWRLLTE-
R4WJK2_RIPPE        CIANHH----VKTKCKPALLRMFSSLQGTQ-------IAAMLLDELHALLVGNKF
W8CCK9_CERCA        NLISNS--EKVRTYTMTLLPTIMHSKNGVT-------YIKIVISELENQLDNVKF
B0W357_CULQU        CVNSHH--KKVRKYCLPRLERIVNSSNGIT-------LAKSLFQELTNFVETTKI
E1ZX97_CAMFO        CATASN--STTRQRCFPLVKKIMTGLSTYA-------PAQALLTEFNKFLENVKI
W5JJ28_ANODA        CVMSAQ--QKVRRYCLPLIRRIVNSEDGVA-------LAKSLLAEMTRYAENVKI
F1KPR4_ASCSU        VLLWPA--FEVRKHALSSLKKLILA-EDTR-------FTSSFLDAFYEYLISGSA
V9K7F0_CALMI        VLLNKT--WRVRKQAQQMVRKLLSSLGGVK-------LAYGLLEELKVVLSSHKV
W0T7Q3_KLUMA        TLLSNGVSFEQRQIAFELFDKSFKR-QPSF-------VGEVVLTALESAVRN---
S9R951_SCHOY        LFNFTG--NQNYRIAFEALKIAVIS-NPS--------LRKTICSQLWNLH-----
S9W134_SCHCR        LFNFSG--NQTSKIAFEALKNAVLV-NPG--------LRKIICSQFWNLH-----
H0GUK0_SACCK        STLSTELSIENRIHAVVLLQELFHK-KPEI-------IGFSVIDAIERRMRA---
E5R2E4_ARTGP        MVCSSVSPPKIRQLATDLLRQCYLK-QIVL-------IGTTVINSIWKWLYH---
J8Q888_SACAR        ATLSTELSVENRIRALNLLQELFYK-KPKT-------IGFCVINAIERRMRV---
C5G992_AJEDR        VICSSSTPPKVREHGVSALKQCYCG-NPAL-------VGTTMITALWGWLHA---
G4MR95_MAGO7        LISSPATGAQVRVDLCKRLSELYLS-NPER-------ISTAVVGGMWSWLES---
C9SQ16_VERA1        LICASAVTPKIRQLALDSLSSLYAA-QPTL-------ISTAIVGAIWHWLEA---
B2WNI9_PYRTR        FTISRTLSSQASSAAKQVLTKSYLRTSPEK-------ISSIIINGLWSWYRS---
S3CN37_OPHP1        AATSASSSAYLRKEAIAALSDLYLR-NPSQ-------VSGAVVSGLWNWLET---
G3ALT0_SPAPN        MLSSNNVMANIRLTGNKLVKQVLET-VPE--------LSDAIIKACYKVLKD---
Q7S5P0_NEUCR        VISSAATGPDVRRDAIDALSTLYAQ-SSAKAEGPSALLAETIVEGLWHWIEA---
A0A165FVX9_9PEZI    FVCAASIPPAIRREATHALTLVYVK-RPEV-------VGPAIISGLWLWNRQ---
A0A178DN93_9PLEO    FIVAQTVSPKAKAAAKQALTKVYLRTSPSK-------VGDILVRGLWSWYRS---
A0A177D7J2_ALTAL    LIVAPSVSAKAKSAAKQALTQSYLQTSPDK-------ISNIIVEGLWSWYRS---
A0A0C4DS68_MAGP6    LVSSATTSSQVRKELCQQISDLYQR-NPKK-------ISSVLTSGIWHWLGA---
A0A151N4J5_ALLMI    VLLSRT--WHVRKHAQQAVRKLLSSLGGFK-------LAYGLLEELKAVLRSHKV
A0A146NKU7_FUNHE    VLLSRS--WRVRKRAQQSVRKLLSSLGGSN-------LARGLLGELRVVINKHKV
A0A0F7Z917_CROAD    VLLSRS--WSVRRLAQQTVRKLLSLPRGFK-------LACGLLEELKAVLISHKV
A0A0W0DC37_CANGB    VCTSIELDAVTRQRSVTLLEKAVSL-QGET-------ISENVINSIANLMKQ---
A0A0L8RIK7_SACEU    ATLSTELSIENRIHAVNLLQELFFK-KPEI-------IGFGVINAIERRMCV---
A0A178FRD2_TRIVO    LVCSLASLPKIRQLATGLLRQCYLK-QPVL-------IGSIIINSIWKWLYY---
A0A175WD14_9PEZI    LASSTATPPAVRRQALESLSNLCV--QPAGSD--RVSVAAEIVDGMWHWIEA---
A0A194VFU1_9PEZI    LICSTTVSQKVRKEAADILSEVYIS-NPGK-------VARVLTNGLWTWIQA---
A0A151TXV6_CAJCA    FICHPR--WDIRRTACNVARKIIAS-APH--------------------------
A0A0M9ABE9_9HYME    CATAPK--YKIRKRCFPLIKKVLTGLSTYD-------PAQELLMEYNKFLENVKI
A0A072U5F0_MEDTR    FICHPR--WDIRRIACNVAKRIITS-VPQ--------LSEDILSEFSKYLNLVE-
A0A0K8V1F4_BACLA    NLISNS--EKLRTYTLSLLPRLMNAIEGVD-------YINNIMVELENQLDNIKF
A0A0A1XE05_BACCU    NLISNS--QKLRTYTLNLLPKLKNTNNGVD-------YIHKIMMELENQIDNIKF
A0A131YTA4_RHIAP    GLSHSS--HPVRQSAQSTVRKLVSVLSGTS-------LACFLLREFSSFVDS---
A0A0J6I2R9_COCPO    VISSAATPENLRESAISHLRNCYLK-NPQV-------VGMAIISAMWKWLLA---
A0A074XMM5_AURPU    LIVASGLPATARSETIASLRTAYLA-QPEA-------VGKTVTDALSHWAAD---
A0A167FV98_9ASCO    YVLSHQVPSSCRRSALEALRNVYMDNSPS--------VGPLLIEAL---------
A0A1A7XZM1_9TELE    VLLSRS--WRVRKRAQQGVRKLLSSLGGSS-------LAHGLLGELRVVINKHKV
A0A0P7V1U6_9TELE    VLLARS--WRVRKRAQQTVRKLLSSLGGSN-------LAHGLLEELKTVANMHKV
A0A1A7WWE1_9TELE    VKLSNP--RLLQKRALSFQKEFYSEERDSS---------------------ARSI
```

```
GCN1_YEAST          -------------QELIPQQNTSFKYVTSVLLAITS-------------ELPDKE 825
GCN1_SCHPO          ----------ADFEKSKSVGKFDSAKYDEISSLFQSLILSSMSADTSNFSNQELV
GCN1_DICDI          --------------DDSLIITPQIINSNVESTTTTTTTISNKKNYLIAFRSILSK
GCN1_MOUSE          -----LPLEA-LVTDAGEVTEMGKTYVPPRVLQEALCVISGVPGLKGDIPSTEQ-
GCN1_HUMAN          -----LPLEA-LVTDAGEVTEAGKAYVPPRVLQEALCVISGVPGLKGDVTDTEQ-
M7ANV9_CHEMY        -----LPIEA-LVTETGELSELGKAYIPPRVLQETLCVISGVAGQEVDRTETEN-
H8X4E7_CANO9        --------------------VHNPRDLSLVFAMLTQ--------------LKEVD
W6A2P0_ICTPU        -----LPPEV-LQTETGEWTELGRLYIPPWILLEALRVLCTTAGQWNDSSEAEK-
B6JZI6_SCHJY        ----------KLREQKKAEVVFYENSVSYFFTLLSAFTL-----DYEAMQPSDKQ
R4WJK2_RIPPE        -----------QSAKENRDNQENHHEFKPRHLVDAIVLICSGTNLSKEDKNTLA-
W8CCK9_CERCA        --------------NNEIDSNQEESVANANILVDVIESICNYEHLSIVDAKTVA-
B0W357_CULQU        ---------------LNEGE-QEEGVVPAQAIVETIVAATTIANI--GSADAQT-
E1ZX97_CAMFO        ----------KSESDKESKDESSMGEITGRCLADGLFAICSGSFLFEVPTYQMT-
W5JJ28_ANODA        ---------------LNEGDPHEDGVAPAQALVDAIWTICDVELL--ASANAQS-
F1KPR4_ASCSU        DYTYHKTCSATTAMSVAEGEKSESEHVEGKWILDALRVVLIPF----DAAEVQSD
V9K7F0_CALMI        -----MSPDL-LVTETGEPTETGKSYIPPRMLLEALSVITSIPGLETDVREAER-
W0T7Q3_KLUMA        --------------SSELLSRISFRRSAPLFTVLSQ-------------PLSDKE
S9R951_SCHOY        ----------YQAESAKKDGKFDEEYNKKLVQLLKSILL-AISADVSTVDSTRFV
S9W134_SCHCR        ----------YQSEASKKDDKFDENSYKKLVQLLKSILL-TISSNISDVNPKEIA
H0GUK0_SACCK        -------------QELIPQQNTSFKYITTVLLTITS-------------ELPDKE
E5R2E4_ARTGP        ----------LDIADKEAAAVLSGSGKSYLSHVVRAITPAVSEMEGHC--SINKK
J8Q888_SACAR        -------------QELIPQQNISFKYITSVLLAITT-------------ELPDKK
C5G992_AJEDR        ----------LDTANKDSAALSAGTGNEKIHLVIKAINLAPSEQNPDD--NISKE
G4MR95_MAGO7        ----------VEEGEKDSAAALAKAENKHLHLILRAICPSAQELPSKFTEEAGKE
C9SQ16_VERA1        ----------LDAGEKDTAAVLARTERNSLHLVVRAICLSPKDWQERE-IVPEEQ
B2WNI9_PYRTR        ----------SEQGDKESAAVLSKAQTGDLGAVLASICLPPDSLKKHD-ASIDTA
S3CN37_OPHP1        ----------QGNGDKESASILSKSQNAQLLPVLGAICRRSSEFVKATGKEPERN
G3ALT0_SPAPN        ----------FEIEQHMEVFNFALKNFSVIFASLTQ--------------LNNQE
Q7S5P0_NEUCR        ----------TEAADKESAAALAKTGTSNLHLVLKAICLKPEEYQKRAGAEPDKT
A0A165FVX9_9PEZI    ----------VSLDDKDSPATSAKTGISQLLLVIRSICPSKQEVEKLS-GKVEKN
A0A178DN93_9PLEO    ----------SEQEDKDSAAVASKTGTSDLNAVIGCLCLSSELLKKLD-ANVDQE
A0A177D7J2_ALTAL    ----------SEKEDKDSAAFASKASSVELGAVLGCICLSPETVKNHN-ASVDDA
A0A0C4DS68_MAGP6    ----------GEEGDKDSAATLAKADTTHLNLILRAICLPARELPEGAAEVTERS
A0A151N4J5_ALLMI    -----LPIEA-LVMESGELSELGKAYVPPRVLHETLCVISSVAGLEVDPTETEK-
A0A146NKU7_FUNHE    -----LPQDV-LVSESGELTELGRSYIPPRVLLDALCVVCSAASQWGDPAEAEN-
A0A0F7Z917_CROAD    -----LPPEA-LVTESGELSEQGKTYIPPRILQEALCVIACGPGMEGELEEKEK-
A0A0W0DC37_CANGB    -------------DSFELDEKCSIVYFIPIFNALVT-------------SNEDKT
A0A0L8RIK7_SACEU    -------------QELVPQQNISCKHITSVLLAITT-------------ELPDKE
A0A178FRD2_TRIVO    ----------LGIADKESAAVLSGAGYSNLSHVVRAITPSVSERDGLD--SINKK
A0A175WD14_9PEZI    ----------MEAADKETAAALAKSGNSNLHLVLKSICLGPKEYEIQA-VDADKA
A0A194VFU1_9PEZI    ----------LEAGDKDSASILAKTDANNMHSVLRAICLTEAEFTKGG-KSTSPE
A0A151TXV6_CAJCA    --------------DNDISLDPQVPFIPSVEVLVKALLIISPAALKQA-------
A0A0M9ABE9_9HYME    ----------KSENDSENKEDSSSCEITGRCIADGLFAICSGSFLFELPAMQMT-
A0A072U5F0_MEDTR    -----EKVSALRISDTDISLDPQVPFIPSVEVLVKALLIMSPAAMKVA-------
A0A0K8V1F4_BACLA    --------------STDYDGYHEENLATSNILVEIMQSICNFQQIP-GEAKKLA-
A0A0A1XE05_BACCU    --------------NTDCD-YQEENVAAKNILGELMQSICNFDQIP-DDAKKLS-
A0A131YTA4_RHIAP    -----------HLKDKAEECGPGEAKI----LAEALVTICSGSNL--DTKDTED-
A0A0J6I2R9_COCPO    ----------VNTGDKDSAALSAGTGGDKLLLVVRAITPSPN--------VVDSL
A0A074XMM5_AURPU    ----------VELETKESAAVLAKTGRQELYKVVRCICLTKEEVAAGQ-ATIEQA
A0A167FV98_9ASCO    ---------------SEKETLTTYTNLQQIINIFST-------------DVDPVQ
A0A1A7XZM1_9TELE    -----LPPDV-LVSESGELTETGRSYIPPRVLLDALSVVCSAASQWGDPAEAEN-
A0A0P7V1U6_9TELE    -----LSHDV-LLSETGELSEIGRSYTPPRVLQEALCTICSVAALQNDPTEAER-
A0A1A7WWE1_9TELE    -----KDIDVGLTANPSKVSELRKRF----------EGITTSSSDW-EMNRKER-
```

```
GCN1_YEAST          ASIKVLINAL-VIAQWNIFNIK----NGWAGLVL---RARLDPAEVVKEHASVIM 880
GCN1_SCHPO          DFDKYLVELL-FLSFAFKDK------FDWIRFCQ---VSKRDPATLVSERIHSII
GCN1_DICDI          NIKSELYPMLSLICYHPFIN------YNWKRVSS---LIQNDVNTTLSSNAIEIS
GCN1_MOUSE          ----LAQEML-IISHHPSLVAVQS--GLWPALLT---RMKIDPDAFITRHLDQII
GCN1_HUMAN          ----LAQEML-IISHHPSLVAVQS--GLWPALLA---RMKIDPEAFITRHLDQII
M7ANV9_CHEMY        ----LALEML-------------------------------------------
H8X4E7_CANO9        QVKSNAVKLI-IAANDPSIRIN----GGWIGLVQR-SGHKVDIGKLVKDNHQQIF
W6A2P0_ICTPU        ----LALDII-IVAHHPSIAAAKS--ELWPLLLS---SMKMDAAVFIDQHLQTIL
B6JZI6_SCHJY        AYDEYLLALL-FLSYYYKSK------IDWIRLCQ---NAKRDPSALVSSNLEKLF
R4WJK2_RIPPE        ------FHAL-LPCHHPLILSSQG--NVWLNITK---HLKLDPPKFVKHYKDKFI
W8CCK9_CERCA        ------LQSL-LICHHPAAVCNDC--FLWEKILK--EKFKLDPEIFVENNTKEII
B0W357_CULQU        ----IALNAL-LPAHHPATVSVQS--DLWESILR---RFELDGKYFISMNAAHIK
E1ZX97_CAMFO        ------RDAL-LPSHHPALLKAVP--NLWFKIAK---NYNLVPKDFLRSYSNEVR
W5JJ28_ANODA        ----LALSAL-LCSHHPAALSVRS--DLWESILA---RYKLDGKHFIALNTAQIG
F1KPR4_ASCSU        VALSTLTNAL-LVCSIPTVVEKGG--LEWMRWFH-----SIENQSTLFEGGTSVL
V9K7F0_CALMI        ----LAREIL-FMAHHPSIEVAHV--GLWSTILL---RLKLDPREFIKKYLEEIL
W0T7Q3_KLUMA        VSADILKKML-VLSQLSTVNLK----NGWAGLVL---NSRLDPAEVVRSSGEKML
S9R951_SCHOY        DYDFYLIELL-FLSFAYVDQ------FNWIQLCQ---LSHRDPATLVTERIHEIV
S9W134_SCHCR        DYDFYLIELL-FLSFAYVDQ------FDWIQLCQ---LSHRDPATLVTERIHDIV
H0GUK0_SACCK        ALINVLIDAL-VIAQWDIFNVK----NGWAGLVL---RAKLDPAEVVKKHADAIM
E5R2E4_ARTGP        DLEIQCIELL-VLCR-PELVPG----TSWINVSL---KMGVDPGKLAIEYPGQCL
J8Q888_SACAR        ASIDILIDAL-IVAQWDVFNVK----NGWAGLVL---RAKLDPAEVVKEHANTIM
C5G992_AJEDR        VLKTQLVELL-VICR-PQLIPG----TQWIDVVL---KTGIDPGELVREKYEECM
G4MR95_MAGO7        VLEAQLCSLL-VLAR-SDIVPR----SSWIDLCL---RSGLDPGQLATKHEKTLL
C9SQ16_VERA1        HLERQLCALF-VLAR-PELLPR----SNWIDLCL---RVEKDPGDLARRYEADLL
B2WNI9_PYRTR        VLQKQAMSLV-VLAR-KEIMPR----VSWIDLCL---RMGVDPGELVRNHLEAFM
S3CN37_OPHP1        LLEAQMCSLV-VLAR-SVLIPA----SSWIDLCL---RVEVDPGSLAQHHEKLLL
G3ALT0_SPAPN        LVRDNIQKLA-IIANHAQATVN----NGWIGLAQR-SNMKLDLGDLIKENHSQIL
Q7S5P0_NEUCR        KLESQMCSLL-VLAK-SQLIPR----ASWIDLCL---KVETDPGELARKYEQRLL
A0A165FVX9_9PEZI    ILEDQLLALQ-VLCR-PELIPS----ASWIEVCL---RTGMDPGTLVNERPLEYL
A0A178DN93_9PLEO    DLRQQALNLV-VLAR-PEIMPR----VSWIDLCL---RMNIDPGLLVREKLQGFM
A0A177D7J2_ALTAL    SLQKQAMNLI-VLAR-KEIIPR----VSWIDQCL---RMGVDPGQLVRDNLQEFV
A0A0C4DS68_MAGP6    ALEAQMCSLL-VLAR-PELVPR----SSWIDLCL---RVGLDPGELAKNNEKVLL
A0A151N4J5_ALLMI    ----LALEML-LVSHHPSLVVVQS--GLWPALLI---RMKIDPKDFITKHLDEIL
A0A146NKU7_FUNHE    ----VAMETL-IVAHHPSIVNARP--GLWPVLLA---SMNIRAEELIEKNLEAIL
A0A0F7Z917_CROAD    ----LVLETL-LVSSHPSLVAGQP--GLWPALLI---KMKLDPVDFITRHLEKIF
A0A0W0DC37_CANGB    TQGNILRFLL-VISQYDKFRIK----NGWAGLAL---KCGIDPSQIIAENTSTII
A0A0L8RIK7_SACEU    ALIKVLVNAL-IVAQWNIFNVK----NGWAGLVL---RAKLDPAEIVKQNANVII
A0A178FRD2_TRIVO    DLESQCIELL-VLCR-PELVPG----TSWINLSL---KMGIDPGKLAIEYPDQCL
A0A175WD14_9PEZI    QQELLMCSLL-VLAK-PQLVPR----ASWIDLCL---KVNLDPGELARKHEAALI
A0A194VFU1_9PEZI    QLEEQMSSLL-VLAR-PRLIPR----VAWIELCL---RVGVDPGKLAKKHDKVLL
A0A151TXV6_CAJCA    --PESFVRII-LCSHHPSVVGSAKRDAVWKRLSKCLQTHGFVVIDIISTNVGNFL
A0A0M9ABE9_9HYME    ------RDAL-IPSHHPAIYKAMP--NLWFKIAK---NFNLVPKKFLCTFNHEIK
A0A072U5F0_MEDTR    --PDSFVRII-LCSHHPCVVGSAKRDAVWKRLCKCLQTHGFDVIDIVAANVINFV
A0A0K8V1F4_BACLA    ------LQAL-LICHHPGVFCNNS--VLWETILK--SKLNFDPEIFVSVNAKDIT
A0A0A1XE05_BACCU    ------LQSL-LICHHPGVFCNNS--VLWETILK--NKFNFDPEIFVSLNAKEIT
A0A131YTA4_RHIAP    ----LALESL-LPAHHPVFAAHHP--NLWLRIVD-----LLKLERKAPMDPQQAL
A0A0J6I2R9_COCPO    VLDRQLIELL-VLCR-PELIPR----ANWIEITL---RAGADPGKIAQSYPKECI
A0A074XMM5_AURPU    VLDHQLVNLL-ILCR-PELTPR----SSWIETCL---KTGTDPGELVKRNTSKCM
A0A167FV98_9ASCO    AKESNLAKLI-FVCHYPGALGK----ASWATLCL---RAGVDPGELVAHLHDSML
A0A1A7XZM1_9TELE    ----LAMETL-IVTHHPSIIQARS--GLWPVLLS---SMNITAEEFIERNLESIL
A0A0P7V1U6_9TELE    ----LAIEIL-VIAHHPSIEAARP--GLWPVLLS---HMNINPTEFIDKHLDSIL
A0A1A7WWE1_9TELE    --IARRLEGI-DGEAHPA---------LQP---------------------SSVA
```

```
GCN1_YEAST          EKILEITGSCEWIDT-IYGACGLQAAAYAAFIQPNE--FTPILCKTIEADLTADD 935
GCN1_SCHPO          EEIELLLSSAIKDSK---ETAAIASISMIVFVAPEE--SIPLFVNVFRNQLLHLN
GCN1_DICDI          KYI--FEKGLNQKKNKSYQQAFQQAINGLMNYNVPL--LNEELVKLMVKALSYEP
GCN1_MOUSE          PRI---------TTQSPLNQSSMNAMGSLSVLSPDR--VLPQLISTITASVQNPA
GCN1_HUMAN          PRM---------TTQSPLNQSSMNAMGSLSVLSPDR--VLPQLISTITASVQNPA
M7ANV9_CHEMY        ------------------LSSMNAVGSLSALSPRK--VLPQLISTISASMENPA
H8X4E7_CANO9        DECVA-----------NPTPNSIKALATIAFVQPES---VGVITKFIDESLSVEH
W6A2P0_ICTPU        PLL---------LEGNTDNQAVRNAIGELSVLSPGK--LLPCIMDRVIQRLSNPA
B6JZI6_SCHJY        RNYELVLAESGNELK---TNAVISSLEMVAFVAPDD--AIPHIVKLFRSQLENIR
R4WJK2_RIPPE        KLL---------IEDFQCHPSYERALKTIAEINSTL--ILPPLLNTVNEVFSDAT
W8CCK9_CERCA        NIL---------IDNFQLKNPYENAISTVVRLSPGK--VLPTLIKKIITDLKNIE
B0W357_CULQU        EVF---------FNKYKATPMYENTLATLSAISPEV--ILPVLVKNVTDHLNNAR
E1ZX97_CAMFO        KML---------IQNYKPAPNYENALVRIVSLAPDA--FLPALVSNVTSKLDDSE
W5JJ28_ANODA        DVF---------FAVYKAKPMYESTLATLSRISPEQ--ILPVLVKNVCDQLTNSR
F1KPR4_ASCSU        NGC---VQKVFTINDDCVRHNAIRLLMSAGDISMHFRGIVWQKVREMVYGIDVQQ
V9K7F0_CALMI        PSV---------GSQSARDQATLNAMGSMAVLASGY--VLPRLSNLVTSMLGKQA
W0T7Q3_KLUMA        KDMLADLSDSKVVKS-ELRDAVVKAIAHISFINPEV--ISPLLAVAIKTNLNTSK
S9R951_SCHOY        YYIATTLSNNVRSEK---EKAMISSISMILFVAPEV--ATPLFVNLFRGQLVNLK
S9W134_SCHCR        KYIEVALSSDVRSEK---EKAMISSISMIVFVAPEA--STPLFVNLFRGQLVNLK
H0GUK0_SACCK        DKILEITNNNEWIDT-IYGSCGLQAAAYAAFIQPSE--FTPILCKTIEADLSTND
E5R2E4_ARTGP        NQILLSTNDPIRNNIADARQASWDAAADLGFVAPEV--MIPKLVAQFCDDLKPER
J8Q888_SACAR        DKIVGITSNDLWIDT-IYGTCGLHAAAYAAFIQPAE--FTPILCKSIEADLTTDD
C5G992_AJEDR        SQILRAIDDPIRSQITKGRPAAWDAAADLAFVAPDT--IIPRLVKQLHEDLNPKR
G4MR95_MAGO7        QEV--ALRSSFDQKSDAIRTASYKAAVELVFVAPEV--MTPRIIQLIQHDLDTSE
C9SQ16_VERA1        DEI--RNKSGAEQSSALVKKAACNAAAELAFVAPET--MTPRIVELIEQDLAVEE
B2WNI9_PYRTR        STVNSVTENKENDQFPAVSLAAYSAYTDMAFVAPDT--ALPAVVKQFSQDLDPKQ
S3CN37_OPHP1        DEI--AARTQLTQTSTAIKTAAYEAAAELVFVAPEK--MAPRIVELIQNDLNTDE
G3ALT0_SPAPN        KECALVTSDAPASGK--LYQAAIKAIGNIAFIQPAI--VSPILSNIIKENLDVEK
Q7S5P0_NEUCR        DEI--VNRTLYEQKSEAVRLAAYNAAAELVFVAPET--MTPRIVDLIQQNLKVSE
A0A165FVX9_9PEZI    DAIERVYQDHFDSYGGTINHAGYKASAELAFVAPDV--ITPLLKDRVASYVDPQL
A0A178DN93_9PLEO    DLITGTTEDKENDRFPAISLAAYNAYTDIAFVAPEV--GLPAVVRQLSQDLDPTQ
A0A177D7J2_ALTAL    NTINEATENKDNEPLPAINAAAYSAYTDMAFVAPEA--ALPAVIKQFSQDLDPKQ
A0A0C4DS68_MAGP6    QEV--AIRSTLDQKSAAVRNAAYKAAVELVFVAPDV--MTPRIIQLIEQDLDVAA
A0A151N4J5_ALLMI    PRI---------TTQTPMNQSSMNAVGSLSLLSPGR--VLPQLISTISATMENPA
A0A146NKU7_FUNHE    PQL---------LEVNADSQAVKNAVGALSVLSPNK--LLPRVMSHVIEGLSQPT
A0A0F7Z917_CROAD    DRI---------MTQSPMNQSSLNAVGLLSVLLPAK--VLPQLISTISASLENPA
A0A0W0DC37_CANGB    NTIISESSKPELLNT-IYGSCVIKAASYIAFINPVA--VAGPIAKLFSEDLSVAD
A0A0L8RIK7_SACEU    DKIDRITTDNEWIDT-IYGTCALQAAAYAAFIQPAE--FTPLLCRTIATDLEIGD
A0A178FRD2_TRIVO    NQILLSIGDPIRHNIAEARQAAWNAAADLGFVAPEV--MIPKLVAQFCEDLKPER
A0A175WD14_9PEZI    AEI--VGRTGFGQKSDAVKTAAYNAAAELVFVAPET--MTPRVITLIQQDLDAAA
A0A194VFU1_9PEZI    EEV--VQRSEFWQPT-SIKEASYDAAAELAFVAPAT--MTPQIEELIRSDLNVEK
A0A151TXV6_CAJCA    QVL--LGPMGLKSANPLEQQAAILSLSNLMSIIPGD--TYAEFEKHLLDLPERFA
A0A0M9ABE9_9HYME    KIL---------VQNYKPVASYENALTKVISIIPDI--ILPGIVFNITSKLDDPE
A0A072U5F0_MEDTR    QVL--LGPMGLRSANPLEQEAAISSLSNLMSIIPGD--TYTEFEKHLLNLPERFS
A0A0K8V1F4_BACLA    NII---------IDKFQLKKTYENSISTIIRLSPGK--LLPILMQKIITDLLSVE
A0A0A1XE05_BACCU    NII---------VDNFQPKKTYENSISTIVRLSPGK--FLPILVQNIITDLIYVG
A0A131YTA4_RHIAP    PLL---------LSRDTIDPDVKGAITTLTAVLPEF--VMNSVISKVLDALSQTE
A0A0J6I2R9_COCPO    AQVLLATEDPIRSKLSKGRTAAWNAAADLAFVAPDV--MMPILVKQIQEDLNIDR
A0A074XMM5_AURPU    DQIIRVSEDKLLATIPAFQLAAFQATADLAFVAPED--LTSVILDQICSDLDPKL
A0A167FV98_9ASCO    RDIFSVVDD-------SLYPTAFAALATLCFIRSDL--IVPSVVQFINSSLNSAL
A0A1A7XZM1_9TELE    PQL---------LEVNADSQAVKNAVGALCVLSPNK--LLPRVISHVTEGLSQPA
A0A0P7V1U6_9TELE    PCL-----------LDTDSQAAQNAVGSLCVLSPDK--LMPSVMERITYRLSDRA
A0A1A7WWE1_9TELE    KRL---------LEVDA-----------------------PRYTR--ASDLCEPR
```

```
GCN1_YEAST          FSRLSEEDFEI-FAGEEGVLV-VDVLEESMNKK-----LSNKNSKEYETLMW--- 990
GCN1_SCHPO          ISSVSSTDLEI-WKTPEGVLW-DNVLEKKSSKK------LDKNTKDYETKRW---
GCN1_DICDI          VLAITQQQWSI-YHTLPTELF-------VEKQEQLVESRNDRKVKPKTAEEQ---
GCN1_MOUSE          LCLVTREEFSI-MQTPAGELFDKSIIQSAQQ-DSIKKANMKRENKAYSFKEQ---
GCN1_HUMAN          LRLVTREEFAI-MQTPAGELYDKSIIQSAQQ-DSIKKANMKRENKAYSFKEQ---
M7ANV9_CHEMY        LRHVTREEFAI-MQTPEGELFDKSIIQSAQQ-DSMKKANMKRENKAYSFKEQ---
H8X4E7_CANO9        IDEVSK---SI-YYGKEGEMV-IDVTAKKPKAL-------DKNAKDYEIRAW---
W6A2P0_ICTPU        LKQVTRREYAI-MKTPEGELYDKSIIMSAQQ-ESTKKGNMKRENKAYSFKEQ---
B6JZI6_SCHJY        FDNITDTDIAI-WKTPEGTMY-HNVLEKQTKLQ--------KNTKDYETKKW---
R4WJK2_RIPPE        LLNVSPMEYAI-FKSPEGELFDKSILQSKENETTMNIKNMKRESKVYSYKEQ---
W8CCK9_CERCA        TSTISDEEYSI-FVTLKDNLLDKEGNES-----IYASGGQMRDIKGQYDKSQ---
B0W357_CULQU        MGNVTDEEYFT-YLTPDGELYDKSVIPNSDE--QTNTAHLKRENKAYSYKEQ---
E1ZX97_CAMFO        ILRVTKDEYFT-YLTPEGELYDKSVLPVNDENDILNSMNMKRESKVYSFKEQ---
W5JJ28_ANODA        MSDVTDEEYFT-YLTPDGELYDKSVLPSSDE--PIQTAHLKRENKAYSYKEQ---
F1KPR4_ASCSU        YLAITEKDVAI-YKVPDGKLYNTEVIDQNSE-EAIEAKNVKRESKAYKFKEQ---
V9K7F0_CALMI        LHLVTREEFEI-LQTPEGELHDKSLILSTQQ-ETAKKSNMKRENKAYSFKEQ---
W0T7Q3_KLUMA        LSAFATEDIQI-WKGEEGELV-IDVLSAKSSAA-----LTDKNSKDYEILKW---
S9R951_SCHOY        LTNVSDMEYEV-WKTPDGVLW-DNVLEKKSNKI-------DKNTKDYETKLW---
S9W134_SCHCR        LADVSDLEYEI-WKTPDGILW-ENVLEKKTQKM-------DKNTKDYETKRW---
H0GUK0_SACCK        LSQLSEEDFNI-FAGEEGILV-VDVLEESMNKK-----LSNKNSREYETLIW---
E5R2E4_ARTGP        ISQLSAIDIAI-ARHTGDTPF-VDVLDTK-AKN-----LPNKGARDYDTLKW---
J8Q888_SACAR        LSQLSEEDFDI-FAGEEGVLV-VDVLEESMSKK-----LSNKNSKDYETLMW---
C5G992_AJEDR        VLKFESTDLAI-ARTPEGTMF-VDVLSTK-SKP-----VIGKG-KDSDTLKW---
G4MR95_MAGO7        LKDIGPLEAAI-FRTPEGTTF-VDVLAKK-GTT----VS-TKG-KDADTLKW---
C9SQ16_VERA1        LKKIGPLEAAI-YRTPEGTAF-VDVLAKKAQDT-----VPDKNNKDYDILKW---
B2WNI9_PYRTR        LESVGPTEAAI-FRTPEGTAY-IDVLSKK------APVVIDKNTKDYDTLKW---
S3CN37_OPHP1        LKTVGPVEAAI-FRTPEGTTF-VDVLAKK-TSS-----LPSKSSKDYDVLKW---
G3ALT0_SPAPN        FSSIDNETLKI-YNGKEGELV-IDVLNKDTKKQ-----VEDKNSKDYEIKKW---
Q7S5P0_NEUCR        VQSVGPLEAAI-FRTPEGTAF-VDVLAKK-QNV-----VPNKNDPNYHTLKW---
A0A165FVX9_9PEZI    LQDIGPTEAAI-FRTPEGTTF-IDPLAKKGQSI-----VPDKNVKDYDIMKW---
A0A178DN93_9PLEO    LESVGPTEVAM-FRTPEGTAY-IDVLSKK------APVVIDKNIKDYDTLKW---
A0A177D7J2_ALTAL    MESVGPTEAAI-FRTPEGTPY-IDVLSKK------APVVIDKNTKDYDTLKW---
A0A0C4DS68_MAGP6    LEGIGPLEAAI-FRTPEGTAF-VDVLAKK-SSN-----VPTKG-KEADTLKW---
A0A151N4J5_ALLMI    LHHVTREEYAI-MKTPEGELYDKSIIQSAQQ-DSMKKANMKRENKAYSFKEQ---
A0A146NKU7_FUNHE    LLQVTREEYAI-MQTPDGELYDNSIIQSAQK-ENTKKVNMKRENKAYSYKEQ---
A0A0F7Z917_CROAD    LCLVTQEEFAI-MKVPEGELYDKSILHSAQQ-DSLKKANMKRENKAYSFKEQ---
A0A0W0DC37_CANGB    LDKLTEEDVSI-WKGNEGEMV-INVLEKRNDRK-----LADKNSKDYETLKW---
A0A0L8RIK7_SACEU    LSQLSEEDIDI-FNGDEGVLV-INVLEESINKK-----LSNKNSKEYETLMW---
A0A178FRD2_TRIVO    ISQFSATDIAI-ARHTGDTPF-IDVLDTK-AKN-----LPNKSAKDYDTLKW---
A0A175WD14_9PEZI    VRTVGPLEAAI-FRTPEGTAF-VDVLAKK-QNV-----VPNKNTKDYDTMKW---
A0A194VFU1_9PEZI    LQDIGPMEAAI-FRTPEGTAF-VDVLAKK-ANE-----VPNKNQKDYDTLKW---
A0A151TXV6_CAJCA    HDTLSENDIQI-FRTPEGMLSTEQGVYVAEAVAAKNTKQAKGRFRMYDDEDG---
A0A0M9ABE9_9HYME    ILKVTKDEYFT-YLTPEGELYDKSVLPTNDENDILNSMNMRRESKVYSFKEQ---
A0A072U5F0_MEDTR    HNALSENDIQI-FHTPEGMLSTEQGIYVAESVAFKNTKQAKGRFRMYGEEDG---
A0A0K8V1F4_BACLA    SSNITEEEYYI-YLTSKQEMPDDKAVKN---------EGNGRENKHQNEKTI---
A0A0A1XE05_BACCU    SSDISEEEYYI-FVSPKGETFDEKAIKN----DIYATTGHTRDNKHQIEKII---
A0A131YTA4_RHIAP    LRLVTAEEYSI-YSTPEGVLHNKSVLSTVVE-ATTDSKNIKRESKVYSFKEQ---
A0A0J6I2R9_COCPO    ITRFGPTDIAI-ARHSGETPF-IDVLSTK-TKR-----LPGKGDKDYDTLKW---
A0A074XMM5_AURPU    LQDVGPTEAAI-FRTPEGTAF-IDVLAKQTQAQ-----APSKNSKDYDTYKW---
A0A167FV98_9ASCO    ISEFSADDITI-WNSPEGELVDLDARNSSTKRKVYVE---NKNSKDFETRKW---
A0A1A7XZM1_9TELE    LLQVTREEYNI-MLTPEGELYDNSIMQSAQK-ENTKKVNMKRENKAYSYKEQ---
A0A0P7V1U6_9TELE    LRQVTREEYAI-MLTPEGELYDKSIIQSAQQ-ENTKKGNLKRENKAYSFKEQ---
A0A1A7WWE1_9TELE    SVRV--QQYSMEVDTP---------LKSS---DPGSRAGSRPEPKSSNHTEPAHG
                        .
```

```
GCN1_YEAST          --E---QKIRKEQAKKN------------VKKLSKE---------EQELVNEQLA 1045
GCN1_SCHPO          --E---AEVRAKQSAKK------------PAKLSKD---------QQALVDAQLD
GCN1_DICDI          --R--DEESRKRIEEKKKIQ---------SGELEK----------QEKERQKQLA
GCN1_MOUSE          IIE---MELKEEI---KKKK-----GIKEEVQLTSK---------QKEMLQAQMD
GCN1_HUMAN          IIE---LELKEEI---KKKK-----GIKEEVQLTSK---------QKEMLQAQLD
M7ANV9_CHEMY        IIE---LELKEEI---KKKK-----GIKEEVQLTTK---------QKELLHAQLE
H8X4E7_CANO9        --E---ESIGKELKQ--------------AKKFTSE---------EKKLVAEQIA
W6A2P0_ICTPU        IIE---LELQEEI---KKKK-----GIKDEVQLTSK---------QREMMQNQLE
B6JZI6_SCHJY        --E---AEMRANLAKKK------------PVSLTKE---------QKQAVEEQLR
R4WJK2_RIPPE        QEE---IALRKELEEKKRKE-----GKLSQPELTPK---------QKEAVKIQLE
W8CCK9_CERCA        QEK---NMLITESSDKKRQK--------GKPELSQK---------QQESVKNQLE
B0W357_CULQU        VEE---LALRREIEEKRRKE-----GKSKPPQLTPK---------QKEAIEKQTE
E1ZX97_CAMFO        QEE---LQLRRELYEKRKRE-----GKIKEPKLTPK---------QEETLKAQIA
W5JJ28_ANODA        LEE---LQLRRELEEKRRKE-----GKWKPPQLTPK---------QKELIDKQRE
F1KPR4_ASCSU        VAE---VELRKELAEKRRK----------EGKLTER---------QKKAVEAELQ
V9K7F0_CALMI        IID---LELTEEL---KKKK-----GLKEEVQLSKK---------QKEMLQTQLE
W0T7Q3_KLUMA        --Q---ESIKKDQAKKG------------IKKLTKE---------EQKLVSDQLK
S9R951_SCHOY        --E---AEVRAKQAQKK------------PMKLSKD---------QQVLVDLQLA
S9W134_SCHCR        --E---AEVRAKQAERK------------PMKLNKD---------QQALVDAQLE
H0GUK0_SACCK        --E---QKIRKEQAKKS------------VKRLSKE---------EQELVDRQLA
E5R2E4_ARTGP        --E---EELRAEVAKKHGQK---------QKKLTAD---------EQAKIKAQLE
J8Q888_SACAR        --E---QKIRKEQAKKN------------VKKLSKE---------EQELVDKQLT
C5G992_AJEDR        --E---EELRAQVAQKRGQQ---------AKKLTAD---------EQAKVNSQLT
G4MR95_MAGO7        --E---AELRAQLAEKKGQ----------PKKLSAD---------EKAKVDAQLK
C9SQ16_VERA1        --E---AELRSSLAQKKGQ----------QKKLTAE---------ETARVNAQLK
B2WNI9_PYRTR        --E---EELRAQLAQKKGQ----------TKKLTPD---------EQAKVNAQLA
S3CN37_OPHP1        --E---EELRAQLAAKKGS----------QKKLTAD---------ETAKVNAQLK
G3ALT0_SPAPN        --E---ESIKKEIASKKAP----------AKKLTKE---------EILLVNQQIA
Q7S5P0_NEUCR        --E---EELREQIAQKKGV----------QKKLTAE---------ENAKVNAQLK
A0A165FVX9_9PEZI    --E---EELRTQLAQKAGA----------QKKLTAE---------ERAKVNAQLD
A0A178DN93_9PLEO    --E---EELRAQLAQKKGQ----------AKKLTAD---------EQAKVNAQLS
A0A177D7J2_ALTAL    --E---EELRAQLAQKKGQ----------AKKLSAD---------DQAKVNAQLA
A0A0C4DS68_MAGP6    --E---AELRSQLAQKKGQ----------QQKLSAD---------DKAKVDAQLK
A0A151N4J5_ALLMI    IIE---LELKEEI---KKKK-----GIKDEVQLTSK---------QKEMVHAQLE
A0A146NKU7_FUNHE    IIE---LELQEEM---KRKK-----GIKDEVQLTSK---------QKEMIQNQLE
A0A0F7Z917_CROAD    IIE---LELKEEI---KKKK-----GIKEEIQLTSK---------QKEMLTAQLE
A0A0W0DC37_CANGB    --E---ESIRKEQAKKA------------NLKLSKE---------DQMLVKEQLE
A0A0L8RIK7_SACEU    --E---QKIRKEQAKKS------------IKKLSKD---------EQELVNKQLA
A0A178FRD2_TRIVO    --E---EELRAEVAKKHGQT---------QKKLTAD---------EQAKVKAQLE
A0A175WD14_9PEZI    --E---EELRAQLAQKKGT----------QKKLTAD---------ETAKVNVQLK
A0A194VFU1_9PEZI    --E---EELRAQLAAKKGM----------QKKLTAE---------EHAKVNAQLK
A0A151TXV6_CAJCA    VDH---TRPNHSVKRDQPSREAAGAGKKDIGKAAKKAGKYKGKTAKEEARELLLK
A0A0M9ABE9_9HYME    QEE---LQLRRELYEKRKKE-----GKIQEPKLTPK---------QEEILKAQMT
A0A072U5F0_MEDTR    LDH---TQSNHSMKRDQPSREAAGAGKKDSGKTTKKADK--GKTAKEEARESLLK
A0A0K8V1F4_BACLA    SSE----------SEKKRYK-----GKANLSELSQK---------QQEAIKNQIE
A0A0A1XE05_BACCU    ISE----------SDKKRYK-----GKASLSEPSQK----------QEAIKNQIE
A0A131YTA4_RHIAP    MEE---IELKKELEAKKKSK-------VQEPELTKK---------QKEVMDAQLQ
A0A0J6I2R9_COCPO    --E---AELRAEMAQKRGQH---------QKKLTPE---------EQVKVKAQLL
A0A074XMM5_AURPU    --E---QELRDSLAQKKGT----------TKKLTAD---------EQTKVNAQLA
A0A167FV98_9ASCO    --E---ESVRNEISKKKANA----PDTLKTKKLSKE---------EQTI----LD
A0A1A7XZM1_9TELE    IIE---LELQEEL---KKKK-----GIKEEVQLTSK---------QKELIQIQLE
A0A0P7V1U6_9TELE    IIE---LELQEEI---KKKK-----GIKEEVQLTSK---------QKEMVQNQLE
A0A1A7WWE1_9TELE    LISTGGLELESKA---ERIA-----RYKAERRRQLA---------ERYGISLDHV
```

```
GCN1_YEAST          KESA-----VRSHVSEISTRLKRGIRLVSELSKAACLVQNGIATWFPLAVTKLLY 1100
GCN1_SCHPO          AEAK-----IRSRVNLIALSLERGLGIIRSLGEAV---QLAPALWVEDAIDVLLF
GCN1_DICDI          AQAV-----IRKDVQDVIDRLHLAMDTCQTMAKSSS-NPQFVGEFMSPIIVALLQ
GCN1_MOUSE          KEAQ-----IRRRLQELDGELEAALGLLDAIMARN---PCGLIQYIPVLVDAFLP
GCN1_HUMAN          REAQ-----VRRRLQELDGELEAALGLLDIILAKN---PSGLTQYIPVLVDSFLP
M7ANV9_CHEMY        KESQ-----IRKQLKEAST------------------------------------
H8X4E7_CANO9        KESK-----IRSGIKSTVAKVNFAIGLINELAAQAKVVNNGADSWFPTSVKKLLD
W6A2P0_ICTPU        KESS-----IRKKLQGLDMELQCTVGLLEAIMARH---PPQISHHIPGVLHVLLP
B6JZI6_SCHJY        VEGD-----IRKKVTNVVSSFTHSMFIIRSLAASV---QLRPDLWIEDAINCLLF
R4WJK2_RIPPE        KERA-----IRERLKKDEERLNKAVQLLSCIIAGA---PLELALNLPTVIPPVVH
W8CCK9_CERCA        KEIC-----IRDRLNLLNKKLKIVIAWLESACLGN---PKAVSLYFNELLSQVLS
B0W357_CULQU        KERA-----IKARLRELADIITTLISQIEGAIKGT---PRQLSLFFPTLLPAILS
E1ZX97_CAMFO        KENG-----IRKRLTELKAKIDNTVSLVTCSIRGN---QQELSLYLKDLLPPILK
W5JJ28_ANODA        KENA-----IKLRLRALNDTITTLVSQIEGAAKGT---PKQLSLFFPTLLPAILR
F1KPR4_ASCSU        TERA-----IRERLRSLYEDCERRLGALSAAINGN---PSGSTAHIDIIIDVIIP
V9K7F0_CALMI        REGA-----IRKKLQQLDEELQSALNVLNTVIKRN---PTDLSKHIPALIRSFLP
W0T7Q3_KLUMA        KESE-----IRARVDNFVVMIKSTIAIISQLTKDATLLDNGLANWFPASVNALLS
S9R951_SCHOY        KEAK-----IRQSVNSMVASLERGLEIIQSMGKAV---QLFPGLWIEEAIEVLIF
S9W134_SCHCR        KESK-----IRQSVRRLVVSLERGLGIIQSMGNAV---QLCPGLWVEEAIDVLVF
H0GUK0_SACCK        KESK-----IRLHVSEIFTRLKRGIKLIFVLSKAACLVQNGITIWFPLAVTKFLH
E5R2E4_ARTGP        KESE-----IRDAVNSTEVVIKRGAGIIKSLANSP---PTQADGWINPACSSLCK
J8Q888_SACAR        KESE-----VRLHVSRIFTRLKRGIRLVSELSKSACLVQNGIATWFPLAVKNFLH
C5G992_AJEDR        REAE-----IRKNVQAEEEIIKRGVGIVESLARGP---PTDVEAWINPAVGCVTD
G4MR95_MAGO7        KESA-----IRERVRGVAAKVMRGVGVIKSLATGP---PTDATLWMGPAVNALLD
C9SQ16_VERA1        KESA-----IRHSIRLLEARLFRGIGIIQSLASGP---PTEAALWMGPVIMALLG
B2WNI9_PYRTR        KESA-----IRKEITATEQRMRRGVGIIQSLATGP---PTEAEQWMGSAVGLLIQ
S3CN37_OPHP1        KEAG-----IRERIRLLEARLLRGFGVIRSLATGP---PTDATQWMGPAIKALLD
G3ALT0_SPAPN        EESK-----VRNEVKAIVGDAHYAVSIITELTNSAKLVENGASLWFPVAVIKLLE
Q7S5P0_NEUCR        KEAE-----IRESVRHVAANLLRGFGIVKALATGP---PTDASRWMGPAVKATLS
A0A165FVX9_9PEZI    KEAE-----IRKSVAHAQATLRRGAGLVEALATGP---PTDAEAWMGEAVKDLLG
A0A178DN93_9PLEO    KEAA-----IRSSVAAIERKMRRGVGIVQSLATGP---PTEAEQWMGPAVNLLIQ
A0A177D7J2_ALTAL    KEAA-----IRKEVAATEHRMRRGVGIIRSLATGP---PTEAEQWMGPAVDLLIQ
A0A0C4DS68_MAGP6    KESA-----IRERVRGVAAKVMRGIGVIHSLATGP---PTDAGLWMSPAVKAILG
A0A151N4J5_ALLMI    KESQ-----IRKRLKELDSELETALGLLNTVMKRN---PPGLTQYIPSLVGSFLP
A0A146NKU7_FUNHE    KESA-----VRKRLQGMDAELQSVVGLLEATLKAG---PAQITRELPGVLQVLIP
A0A0F7Z917_CROAD    KESQ-----MRKQLKELDTELESTLGLFDAVLKRN---PPSLSQYIPALVSCFLP
A0A0W0DC37_CANGB    KESK-----IRTHVNGIYLRLYRSLSLIKQLSKEAKLVDNGILVWYPTAVNSLMS
A0A0L8RIK7_SACEU    KESE-----IRLRVSGIFTRLKRGVRLVSELSKAACLVENGIATWFPLAVTKFLR
A0A178FRD2_TRIVO    KESE-----IRDAVNSAEVMIKRGAGIIKSLANSP---PTEADGWINPACSSLCK
A0A175WD14_9PEZI    KEAG-----IRDSVRQIAARLLRGFGIIKALATGP---PTDASRWMGAAIKTTLA
A0A194VFU1_9PEZI    KESE-----IRHRVQGVAAQLLRGIGIVRALATGP---PTEASLWMGTAVESLLN
A0A151TXV6_CAJCA    EEAS-----VRDRVCGIQQNLSLMLRTLGDMAISN---SVFAHSRLPSMVKFVEP
A0A0M9ABE9_9HYME    KETA-----IRKRLTELKFRIDIVVSLTMCSIRGN---SQELSIYLKDFLPLILK
A0A072U5F0_MEDTR    EEAS-----IRDRVREIQKNLSLMLRTLGNMAIAN---SIFAHSRLPSMVKFVEP
A0A0K8V1F4_BACLA    KENY-----TKERVGLLNKKLKSAISMLEAAFAGN---PKAVSLHFHKLLMQLLK
A0A0A1XE05_BACCU    KGNY-----TRDRLGLLNKKLKNAISMLEAAFAGN---PKAVSLHFHELLMQLLQ
A0A131YTA4_RHIAP    KEHA-----IRLRIRKLAGSVERAMLLLDAVMAAP---ASTVCQYGAPLFPQLVQ
A0A0J6I2R9_COCPO    KEAE-----IRKSIQIQEQIIRRGAGIVESLARGP---ATEAEEWINQAVNCLCR
A0A074XMM5_AURPU    KEAE-----IRSHVTSVVAKLRRGIGMVTGLVTGP---PTEAANWFTVTTARLFE
A0A167FV98_9ASCO    KQSA-----IRASVNQIVAKYKRAISLVNTLASQTL-VENGRELWFPASVLKLVE
A0A1A7XZM1_9TELE    KESD-----IRKRLYGMDVELQSVVGLLESTMRAR---PAQITKELPAVLQVLMP
A0A0P7V1U6_9TELE    KESA-----IRKRLQGLHMELQSTAKLLVTLLSRR---PPRLSWYLPGVLQALLP
A0A1A7WWE1_9TELE    PDCDYPSGHAQKKPDGLMCLLEEVAQKLH---RAF---PAQITKELPAVLQVLMP
                              :
```

```
GCN1_YEAST          LCSEPNISKLTED-VNNVFLQL--SQNVS-ERLG--NIRLFLGLATLRVHNAN-- 1155
GCN1_SCHPO          HNVLKYSEPFLKNLAYDTFLLTLKASGFS-ERLGDRSYSSSLASILAHTFSVN--
GCN1_DICDI          LMK----HEITNHQFTQVFEKLICCVPSR-FKLD-RSFARHYIYIINNIY-----
GCN1_MOUSE          LLK----SPLAAPRVKGPFLSLAACVMPP----RLKTLGTLVSHVTLRLLKPE-C
GCN1_HUMAN          LLK----SPLAAPRIKNPFLSLAACVMPS----RLKALGTLVSHVTLRLLKPE-C
M7ANV9_CHEMY        ----------------------------------------LVSHVTLRLMKPA-C
H8X4E7_CANO9        LAISE--GDFFKS--AESFINL--SNLIT-SRFE--VLKTFVGVAVLRLYGVK--
W6A2P0_ICTPU        LLH----SPLAAPHVHQTFLDIGTCVLQK----QLHFLALLVGHVTLRLLKPE-C
B6JZI6_SCHJY        GNIYQESLRFSGTLASETLLCCIKASSLE-ERIG----EANFALKLLKSLSQV--
R4WJK2_RIPPE        GLS----SALCADPLSDLFIALSQPAFTG-VSLQ-PVIVERVAYTALRLLHPK-C
W8CCK9_CERCA        TLK----CPLSEP-LAKLYYKMRETCFED----S-FDLGKDLAIMTIRLEKPV-F
B0W357_CULQU        VFS----SPLAAPSMVKLYLRLMDTCFGV-ESGL-GEIGRDVAIATIRLSKPH-C
E1ZX97_CAMFO        NLG----SPLAAPEMSELYISLRQTVTMD----NSIILGDLIAHVTLRQLQPQ-C
W5JJ28_ANODA        VFS----SPLAAPAMVKLYIRLRDCCFTS----EQVELGRDISIATVRMSKPH-C
F1KPR4_ASCSU        LLK----SSLVSQLAVDAFRAYRDAAFEP-SDD---YLHELILHATIRALHSA--
V9K7F0_CALMI        LFS----SPLVAPRLIEPFLAMGACVMPA----DLQYLGSLIGHVTLRVLKPE-C
W0T7Q3_KLUMA        ILQQDNFYSLFENIGFDLFLQL--SFLLE-DRLG--MFSKTVGYATLLVYKVP--
S9R951_SCHOY        DKVLQLSTPFTGNLALEVFKLVVNASGLP-SRLGESAYSDSLAITLLDSVSGA--
S9W134_SCHCR        GKVLHLSTPFTGDLALVVFKLVVAASGLP-SRLGESAYSDSLAITILNALNAN--
H0GUK0_SACCK        LCSENNILKLTED-VNKVFLQL--SKNVS-ERLG--NIRLFLGLATLRVHNAK--
E5R2E4_ARTGP        LAQLGG-GVLVGDSISSALMAC--GNKVS-SRIG--EMRPFVAIATLRAVGKT--
J8Q888_SACAR        LCSEKNISKVTED-VNKVFLQL--SQNVS-ERLG--NIRLFLGLATLRVHNAK--
C5G992_AJEDR        LAKAGA-GALVGDAVASAYVAC--SNRIS-SRLG--LMRPFVGIATLRALGRT--
G4MR95_MAGO7        AVDAGA-CLITGDAAPTAYLAC--ADRVS-SRLG--SFRQFIGVATLRAHGVE--
C9SQ16_VERA1        VIDAGA-SMLTGEAASTAYLAC--SEKIS-SRLG--PFRPFVGVATLRARGVT--
B2WNI9_PYRTR        AIRAGA-GLLLGDIPATALIAC--SERIS-NRLG--VLRPFVGVAVLRTIGAI--
S3CN37_OPHP1        AIEAGA-CSVTGDAAPLALITC--AERVS-SRLG--ALRPFIGAAILRAHDVI--
G3ALT0_SPAPN        FCKLELVVELFHSSPVDAYLNL--SSLIS-SRLA--LLKQFVGVATLRSYSVQ--
Q7S5P0_NEUCR        VIDAGA-TLITGEAGPLAFISC--SECVT-SRVG--PIRPFIGVATLRAHNVS--
A0A165FVX9_9PEZI    VIEAGA-GLILGDTAANAYVAC--ASLVS-SRLG--VLRPFFGIATLRALGSS--
A0A178DN93_9PLEO    AIRAGA-GLLLGDIPATAFIAC--SERIS-NRLG--VLRPFVGVATLRTIGTI--
A0A177D7J2_ALTAL    AIRAGA-GLLLGDIPATALISC--SERIS-SRLG--TMRPFIGVAVLRTIGSI--
A0A0C4DS68_MAGP6    AIDAGA-CLITGDAAPLAYLAC--ADRVS-TRLG--SFREFIGVATLRAHDVE--
A0A151N4J5_ALLMI    LFK----SPLAAPRIKIPFLSLAYCVMPA----RLRTFGILVSHVTLRLMKPE-C
A0A146NKU7_FUNHE    LLQ----SPLAAPCIQQIFLDIGVCLMPK----HLHHLAVLVGHVTLRLLKPE-C
A0A0F7Z917_CROAD    LLK----SPLAAPRIKAPFLSLVSCVMPD----HLKTFGTVASHVTLRMMKPE-C
A0A0W0DC37_CANGB    LIRTENSYNLLESLLVESFLSL--SENTA-EKLE--HYRLFCGLAILRVYKAK--
A0A0L8RIK7_SACEU    LCSEKNISKLTDD-VNEIFLQL--SQNVS-DRLG--NIRLFLGLATLRVHNAK--
A0A178FRD2_TRIVO    LAQLGG-GVIVGDSISSALMAC--GDKVS-SRIG--EMRPFVAIATIRAVGKT--
A0A175WD14_9PEZI    AIDAGA-CLITGDTGPMAFISC--ADQVA-SRLG--SIRPFIGAATLRAHDVS--
A0A194VFU1_9PEZI    ALDSGA-SLVTGDAAPLAYIAL--AGRIS-DRLA--SMRPFVGVATLRAHEVT--
A0A151TXV6_CAJCA    LMR----SPIVSDEAFETMVKLARCTAPPLCDWA-IDISTALRLIVTDEVHLL-L
A0A0M9ABE9_9HYME    NLG----SPLAAPAMSDLYIHLKEIVKIN-----NPVLSDLVAHVTLRQLQPQ-C
A0A072U5F0_MEDTR    LLR----SPIVSDEAFETLVMLSRCTASPLCDWA-LDISTALRLVVTDEVHLL-L
A0A0K8V1F4_BACLA    LLS----CPIAGESLAKLYFKLREACFED----S-FELGRDIAIMTLRLENPR-L
A0A0A1XE05_BACCU    LLA----CPIAAESVAKLYFKLREACFED----C-FDLGKDIAIITLRLENPR-L
A0A131YTA4_RHIAP    ALTALFNSRLAAPIVVPAYLRLKDVLFTP----DLQHFAASVGYLMLRLAKPC-C
A0A0J6I2R9_COCPO    LVEVDA-GALVGSSISEAYVAC--TSRIS-SRLE--EIRPFIGIATLRSLGKT--
A0A074XMM5_AURPU    VINAGA-GLVLGDAAVLAYLEC--AQKTS-SRLG--AMRSFVGVAALRAAGLT--
A0A167FV98_9ASCO    LLNCP--NQVLGQEIGESFLNL--SLNLS-DRID-PGMKKIIGVAILRSIGVSET
A0A1A7XZM1_9TELE    LLH----SPLAAPRVQQVFLDIGVCLIPK----HLHNLAVLVGHVTLRLLKPE-C
A0A0P7V1U6_9TELE    LLH----SPLAAPVLRQHFLNIGVSLIPK----ELHYLAVLVGQVTLRLLKPE-C
A0A1A7WWE1_9TELE    LLH----SPLAAPRVQQVFLDIGVCLIPK----HLHNLAVLVGHVTLRLLKPE-C
```

```
GCN1_YEAST          GISQDYLQEPLVELLTRVLFR-----IKFVSNQAA-------------------- 1210
GCN1_SCHPO          ------SSENIKELTKSILYK-----LRFAIEQNY--------------------
GCN1_DICDI          -YRPTLSEIQILGFIQKILTH-----IRESIAKEA--------------------
GCN1_MOUSE          ALDKSWCQEELPVAVRRAVSL-----LHTHTIPSRVGKG------------EPDA
GCN1_HUMAN          VLDKSWCQEELSVAVKRAVML-----LHTHTITSRVGKG------------EPGA
M7ANV9_CHEMY        ELDESWCQEELSTATNRVVCL-----LHAHTIPSRTGKG------------EPGT
H8X4E7_CANO9        -VEPKYEEEPLESLLGRILYR-----VKILSDQNP--------------------
W6A2P0_ICTPU        ELDPAWSQEDLNTATHRTIQL-----LHTHTVPHREGKT--------------DA
B6JZI6_SCHJY        -LGYEKSLDNSADLVTNVLHK-----LRFCIEVHG--------------------
R4WJK2_RIPPE        AISSAWAEQDLGKAMRWMIQE-----MDKNVKD----------------------
W8CCK9_CERCA        ALPKEWDANDIDECVIKVLLN-----LQKYILNHRTDMNK-----------DESH
B0W357_CULQU        DLEESWCTANLVELISDILVSVYDETIDKYNVHIEEDGS--------------KN
E1ZX97_CAMFO        DLDQAWEEENLDTAVKRTLNL-----IHTITIKRK--------------------
W5JJ28_ANODA        DLEEGWCTANIVELVSDILVSLYDETIDMYNVHREEPGS--------------KN
F1KPR4_ASCSU        YVDRCWSEEPLASQLSRTVAML---SDRCVVVPILLDGEECAVTEDELLMELGDD
V9K7F0_CALMI        CLDEAWGQEDVLTSTARAVCM-----LRSRTVPSRSGKV------------ESDV
W0T7Q3_KLUMA        HLLSELTADAKLQLISTALFK-----IKHGCRQVP--------------------
S9R951_SCHOY        -------HSVNENVVDSFLYK-----VRFATEQAY--------------------
S9W134_SCHCR        -------PSVDESIIDSFLYK-----VRFAIEQAY--------------------
H0GUK0_SACCK        NISENYLQEPLVELLTRVLFR-----IKFVSDQAE--------------------
E5R2E4_ARTGP        FLRGELETEPLGSLITRILYR-----LRILSEQRP--------------------
J8Q888_SACAR        NISENYSQEPLLELLSRVLFR-----VKFVSNQAA--------------------
C5G992_AJEDR        YLDPALEDEPLGELVARILYR-----LRLGSEQRP--------------------
G4MR95_MAGO7        LLPENLKAEPFEDLVTRVMYR-----LRFAGEQRP--------------------
C9SQ16_VERA1        SLPESQMQEPVEELVTRVLYR-----LRFSGEQRP--------------------
B2WNI9_PYRTR        QLAKEYEDEDLGDLVTRVLYR-----LRFLSEQRP--------------------
S3CN37_OPHP1        SIPDSYQEEPLSDLITRVLYR-----LRFAGEQRP--------------------
G3ALT0_SPAPN        GLSENLTQEPLLALVGRILYR-----IKILADQKP--------------------
Q7S5P0_NEUCR        ALPENLTEEPFDDLITRALYR-----LRFAGEQRP--------------------
A0A165FVX9_9PEZI    TSKPELEQEPLGDLITRLLYR-----LRFAGEQRP--------------------
A0A178DN93_9PLEO    QLSDKYEDEDLGDLVTRVLYR-----LRFLSEQRP--------------------
A0A177D7J2_ALTAL    QLASEYEDEDLGDLVTRVLYR-----LRFLSEQRP--------------------
A0A0C4DS68_MAGP6    LIPENLKQEPLHELVTRVLYR-----LRFAGEQRP--------------------
A0A151N4J5_ALLMI    ELDESWCQEELPTAINRAVTL-----LHAHTIPSKTGKG-------------EPG
A0A146NKU7_FUNHE    DLDQAWDEEDLDTAANRTVLL-----LYDHTVPQREGKT-------------ADV
A0A0F7Z917_CROAD    DLDESWCQEDLPTAVNRVISL-----LHKHTVPTRIAKG------------EEGP
A0A0W0DC37_CANGB    FIPSNYLAENLEELLSRVLFR-----LKIVTSNTE--------------------
A0A0L8RIK7_SACEU    NISENYLQEPLVEALSRVLFR-----IKFVSNQAA--------------------
A0A178FRD2_TRIVO    FLRSELETEPLGSLITRILYR-----LRILSEQRP--------------------
A0A175WD14_9PEZI    ALPENLTQEPFEDLVTRILYR-----LRFAGEQRP--------------------
A0A194VFU1_9PEZI    ALPENLQEENFEDLVTRVLFR-----LRFAGEQRP--------------------
A0A151TXV6_CAJCA    DLVPSVAEEEVNERPFRGLFERILDGLSISCKSGA--------------------
A0A0M9ABE9_9HYME    DLNQAWEEENLDTAVKRTLNL-----LHTTTIKHK--------------------
A0A072U5F0_MEDTR    DLVPSVAEEQVNQKPSHGLFERIIDGLSTSCKSGA--------------------
A0A0K8V1F4_BACLA    DLPNEWITNDMNEIIKSIFLN-----INKILLPRHTDEGE-----------KGSN
A0A0A1XE05_BACCU    ELPNEWITNDVNEVIKSILLN-----IHKLIIYRQTDGNE-----------NAST
A0A131YTA4_RHIAP    EVDPRWTEEDIDSCMRRVVAC-----FHDLCCAPT------------------GA
A0A0J6I2R9_COCPO    YMPSDLEVEPLGTLVTRILYR-----IRLASEQRP--------------------
A0A074XMM5_AURPU    TLPESMQEEPLGDLITRVLYR-----LRFLGEQRP--------------------
A0A167FV98_9ASCO    YIAEEYLAEPLKDLVTRVLYR-----IKFLCVQRP--------------------
A0A1A7XZM1_9TELE    DLDPAWGQEDLDTAVHRTILL-----LHTHTVPQREGKP-------------GDV
A0A0P7V1U6_9TELE    PLDEAWIQEDLSVATQRTVQL-----LHNHTVPHREGRAVLKTDTSPSILSDADT
A0A1A7WWE1_9TELE    DLDPAWGQEDLDTAVHRTILL-----LHTHTVPQREGKP-------------GDV
                                      .
```

```
GCN1_YEAST          --IDSISLTYILPLLINVLEKGKAIALKNADKPVVKAEFVEEDEEEEHLLLAMEI 1265
GCN1_SCHPO          --FEPQMFACIFPLLYDLTFNITNSD---------------EEDEAELQLLVTEI
GCN1_DICDI          --LSGFAFNYFWPIIKNGLE---------------------TTISFTIQEISMEI
GCN1_MOUSE          APLSAPAFSLVFPMLKMVLTEMPYH----------------SEEEEEQMAQILQI
GCN1_HUMAN          APLSAPAFSLVFPFLKMVLTEMPHH----------------SEEEEEWMAQILQI
M7ANV9_CHEMY        ASLSAPAFSLVFPLLKTVLTETPND----------------SEEKEALMVKILQI
H8X4E7_CANO9        --LDAVSLAYALPLLIKVLQIGQSAAFKNSKKIAVTSEFVENDPEEEHLLLSVEI
W6A2P0_ICTPU        RPLSAPGFSFCFPLLQAILSRNSGT----------------SEEVELMLTHVLQI
B6JZI6_SCHJY        --FSTPMFACVFPLLYRLVQKEFNAKT--------------EDERDEQILLVTET
R4WJK2_RIPPE        --ITSPVISYLLPSLKAALSS--------------------EPPNDPIISTAIHL
W8CCK9_CERCA        YILNAPSFAYIFEFLKRALVLNS------------------ITKNEDYILIGIEI
B0W357_CULQU        YLLNAPAFSYTFEFLKRALVLNE------------------AEKDESMLINGIQI
E1ZX97_CAMFO        ELFTAPAFCYVFPFIRKTLL---------------------SYKDDGMIVQGLQL
W5JJ28_ANODA        YLLNAPTFSYTFEFLKRALTLSE------------------ADRDESLLINGIQL
F1KPR4_ASCSU        DTINVGKLCIALPLLNAILSD--------------------RTHTYALRLNTIQF
V9K7F0_CALMI        KPLAAPAFAYCFPLLKVVLTDTVND----------------TEEKEGMLIQALQV
W0T7Q3_KLUMA        --FQSMALTYILPLLVKVMEIGKKVAIKNANKPANRSEFVEEEPEEEQLLLALDI
S9R951_SCHOY        --FEPETFSCIFPLINKICYENATKD---------------DEKSTERLLLAIEM
S9W134_SCHCR        --FEPEAFSCIFPLVNKLCYENATKD---------------DEKSTEHLLLAIEM
H0GUK0_SACCK        --LDPISLTYILPLLINVLEKGKAAALKNADKPVVKAEFVEEDEEEEHLLLAMEI
E5R2E4_ARTGP        --LDGVSLGYILPLIFIVLESN-GIEES-------------KDDSGEQVLLALEF
J8Q888_SACAR        --LDSISLTYILPLLINVLEKGKNAALKNADKPVVKTEFVEEDEEEEHLLLAMEI
C5G992_AJEDR        --FDVATLSYILPLIFIILERD-GIEES-------------KESKGEQVLLALEF
G4MR95_MAGO7        --FDVVSVNYMLPLALLILNKG-GFGAT-------------AEDRDTHLVLATEL
C9SQ16_VERA1        --FDPISLTYTLPLIFFILESG-GLGST-------------PDDRDTQLVLAIEF
B2WNI9_PYRTR        --LDAVSLAYCFPLLFLVLEKG-GIGKTS------------PEESDEQLILAIEV
S3CN37_OPHP1        --FDTVSLIYFLPLLLLVLNKG-GFSDS-------------AEDRDTQVVLAIEI
G3ALT0_SPAPN        --LDSLSLSYILPLLTKVLHDGKSVAIKNASKTAVTSEFVEEDPEEEQLLLAIEI
Q7S5P0_NEUCR        --FDVISLIYMLPLILLVLEKG-GFGSN-------------ADDKDATLVLAIEF
A0A165FVX9_9PEZI    --FDTISLIYLLPLIFVVIKHG-GVGRTN------------AEDGDEQVVLALEF
A0A178DN93_9PLEO    --LDSVSLAYCFPLLFLVLEKG-GIGKAS------------AEEADEQLILVIDI
A0A177D7J2_ALTAL    --LDAVSLAYCFPLLFLVLEKG-GIGKSA------------AEEADEQLILAIEV
A0A0C4DS68_MAGP6    --FDSVSAIYMLPLVLLVLQKG-GFGGT-------------AEDKDTQIVLATEF
A0A151N4J5_ALLMI    A-LSAPAFSLVFPLLKTVLTETPND----------------SEEKEELMVRILQI
A0A146NKU7_FUNHE    APLAAPAFSFCFPLLNAMLRESSGS----------------TEEAESLMSRALQV
A0A0F7Z917_CROAD    VPLSAPAFALVFPLLKMVLLETPND----------------SEEKEELMVKVLQI
A0A0W0DC37_CANGB    --LDSITLTYLLPLITYVLEEGKKAALKNADKPVNRTEFVEEDSEEENLLLAMDI
A0A0L8RIK7_SACEU    --LDSISLTYILPLLINVLEKGKSVALKNADKPVVKAEFIEEDEEEEHLLLAMEI
A0A178FRD2_TRIVO    --LDGVSLGYILPLIFIVLENN-GIEES-------------KDDSGEQVLLALEF
A0A175WD14_9PEZI    --FDTVSLIYFLRLVLLVLEKG-GFGTN-------------ADDRDAQLVLAIEI
A0A194VFU1_9PEZI    --FDSVSLVYILPLILFVIRKG-GFGAT-------------VDDRDAQLVLAIEF
A0A151TXV6_CAJCA    --LPVDSFSFVFPIIERILL---------------------CSKKTKFHDDVLQI
A0A0M9ABE9_9HYME    KLFTAPTFCYIFPFIKKTLL---------------------SYRDDNMIVQGLQI
A0A072U5F0_MEDTR    --LPVDSFTFVFPIMERILL---------------------CSKKTKFHDDVLRL
A0A0K8V1F4_BACLA    TLFSAPSFTYTFEFLIRAFATDF------------------VANSEELLLIGIQL
A0A0A1XE05_BACCU    TLFNAPSFTYIFEFLKRAFVTNF------------------VANSEELLFIGIQI
A0A131YTA4_RHIAP    RRLPSPAFCFAFPLLRLLLSS--------------------PDTNDTLLTQCLQV
A0A0J6I2R9_COCPO    --FDVVSFGIMLRLIIVVLEKD-GVKEA-------------AESRGEQILLALEF
A0A074XMM5_AURPU    --LDAVTLSYCLPLAFLVLEQG-GVGKSE------------EEEAQEQIILAAEF
A0A167FV98_9ASCO    --LDAITLVYVIPLLLRTLENKGGIGTSS------------DDEIEEQVILALDI
A0A1A7XZM1_9TELE    APLSAPAFSFCFPFLNVTLGESSGS----------------TEETENIMSRALQV
A0A0P7V1U6_9TELE    KPLSAPAFSFCFPLLHAVLSQTPST----------------SEEAESMLVRVLQI
A0A1A7WWE1_9TELE    APLSAPAFSFCFPFLNVTLGESSGS----------------TEETENIMSRALQV
                      :
```

```
GCN1_YEAST          ISVHAEAF--------------EDPSIPRISIVEVLLSLL-SLPSKAK-IAKDCF 1320
GCN1_SCHPO          LEFQALYS--------------ASLRRMRSKLIKSLLHLLEIAPTQYQ-ENKNSL
GCN1_DICDI          IQKHTAQG----------------QAYPRGSMISSLIIVV-STNSRLEAQARNTI
GCN1_MOUSE          LTVHAQLR-ASPDTPPERVDENGPELLPRVAMLRLLTWVIGTGSPRLQVLASDTL
GCN1_HUMAN          LTVQAQLR-ASPNTPPGRVDENGPELLPRVAMLRLLTWVIGTGSPRLQVLASDTL
M7ANV9_CHEMY        LTVHAQLR--SASGQTLLVDENGPELLPRTDMLLLLTRVIGTGSPRLQVLASNTL
H8X4E7_CANO9        ISAHAELF--------------ADAGIPRTSILKILVSLM-AVPSKSK-LVKECF
W6A2P0_ICTPU        IHTHSQLR-SSSDATDELIDENGPELLPRVNMLLLLTKVIGTSTPQLQVLASGCL
B6JZI6_SCHJY        LIMQAPTA--------------HELYAMRCKYLESLLHLVAAVPSQYH-EVRDAM
R4WJK2_RIPPE        LSTRVRLR----GSSNRNPDLHSPVLLPRRQMMTLCIDIISSSGGRVQQQASACL
W8CCK9_CERCA        IETYAEIR--GYTVLGDLTDCKHPKYMPRCEMLKLLLYFIKNSHGRVQTNAAAVL
B0W357_CULQU        IAYHAQLK-GDTVDGQDFEDLYHPRYMPRLEMIKLLLRLIQNHRGRVQTQAVAAL
E1ZX97_CAMFO        IQEHAKQR---GGSTTDLKDIRHPRLLPRKQMFDLLIELMETTTGRVQSHAVATL
W5JJ28_ANODA        IAYHAQLK-GDTVDGKDFDDLYHPVFMPRLEMIRLLLRLIQKHRGRVQTQAVAAL
F1KPR4_ASCSU        IAAAFSKR---------FIKNGEVCMLPLSRLCSMLLAVLEDESVELSQLSCSAL
V9K7F0_CALMI        ISEHSQLR-SAGDDVDELIDENGPELLPRTEMLLLLTRVMGTSSPRLQVLASNTL
W0T7Q3_KLUMA        ISSHGEAF--------------QDTSIPRSSIISVLLSLL-ALPSKAK-LAKEYF
S9R951_SCHOY        LGFQAPYS--------------SSLRGMRALFLKVLLHLLETVPAHYQ-DVKNSL
S9W134_SCHCR        LGFQAPYS--------------FCLRGMRAMFLKALLHLLETVPAHYQ-DIKNSL
H0GUK0_SACCK        ISVHASAF--------------EDPSIPRISIIEVLLSLL-SLPSKAK-IAKECF
E5R2E4_ARTGP        ISFHTNSF--------------SDARLPRLETLRHLINSMRKHTAHYK-LVRDAL
J8Q888_SACAR        ISVHAEAF--------------EDPSIPRTSIIEVLLSLL-SLPSKAK-IAKDCF
C5G992_AJEDR        LSFHTNSF--------------SDNRLPRIRTLQSLISSMQIYTQHYK-IIRDTL
G4MR95_MAGO7        LSFHTDTA--------------SSEALPRAQMLETLISSMQVYNQHYK-IIRDCF
C9SQ16_VERA1        LSFHTNVC--------------EDQTTPRGQVLSVLVTAMQQYTQHFK-IIKDCF
B2WNI9_PYRTR        LAFHTDSC--------------TDPRLPRKSLLEILVWSMQRYQQHYK-MIKDCI
S3CN37_OPHP1        LSFHTDVC--------------ADEAIPRSDLLSSLISSMETYSQHYK-IIKDCF
G3ALT0_SPAPN        ISAHADSF--------------EDEGIPRSSILEVLISLM-KLPSKAK-LSKDCF
Q7S5P0_NEUCR        LSFHTDVY--------------ADEATPRAEILSTLITSMQNYNQHYK-IIKDCF
A0A165FVX9_9PEZI    ISFHTDAF--------------SRQSLQRNELLSSLIFAMQKYTQHYR-LVKDCL
A0A178DN93_9PLEO    LTFHTNSC--------------TDTRLPRKELLETLIWSMQRYQQHYK-LIKDCL
A0A177D7J2_ALTAL    LTFHTNSC--------------TDPRLPRKALLEILVWSLQKYQQHYK-IIKDCI
A0A0C4DS68_MAGP6    LGFHTDTS--------------ADEALPRAEMLETLISSMRQYNQHYK-IIRDCF
A0A151N4J5_ALLMI    LMVHAQMRSSLANGQTLLVDENGPELLPRTDMLLLLTRVIGTGSPRLQVLASNAL
A0A146NKU7_FUNHE    INVHSQLR-ASTDSDDMAIDENGPELLPRVNMLLLLTRVISTATPRLQVLASQCL
A0A0F7Z917_CROAD    ITVHAQLR-SAANNEDWLVDENGPELLPRTDMLLLLTKVIGTGSPRVQVLGSVAL
A0A0W0DC37_CANGB    ISAQAPLF--------------ENSSIPRASILQVLLSLL-SLPSKAK-IAKDCL
A0A0L8RIK7_SACEU    ISVHAEAF--------------VDPSIPRISIIEVLLSLL-SLPSKAK-IAKDCF
A0A178FRD2_TRIVO    ISFHTNSF--------------SDARLPRIETLRHLINAMRKHTAHYK-LVRDAL
A0A175WD14_9PEZI    LSFHTDAS--------------ADEAIPRAEILSVLISSMQQYGQHYK-IIKDCF
A0A194VFU1_9PEZI    IQYHTDVC--------------ADETIPRSDILSVLIFSMQTYAQHYK-ITRDCF
A0A151TXV6_CAJCA    FYLHLDPH----------------LPLPRIRMLSVLYHVLGVVPA-YQASIGPAL
A0A0M9ABE9_9HYME    IQEHAKQR---GSSS-DFRDMKHPQLLPRKHMFDLLIELMEITSGRVQSHAVATL
A0A072U5F0_MEDTR    IYLHMDAH----------------LPLPRVRMLSVLYHALSVVPA-YKASIGPAL
A0A0K8V1F4_BACLA    IESHAQIR--GFTVFGEVTDYNHPKYMPRLEMSKLLLNLINIYQGRVQTQAVGAM
A0A0A1XE05_BACCU    IESHAQIR--GFTVIGEVTDYNHPKYMPCLEMSKLLLNIINTYQGRVETQAAGAI
A0A131YTA4_RHIAP    LSAHSVMR------CTEPFSVENPKNLPVEKMLETVVGLLGRTTGRIQRLAQKVA
A0A0J6I2R9_COCPO    LTIHANLF--------------SDSRLPRTEVLRTLISAMRIHAEHYK-LMRDLL
A0A074XMM5_AURPU    LSFHADTC--------------SEPRLARKEVLSVLVSSLQAYQQHFR-LFKDCF
A0A167FV98_9ASCO    LSAHAEQL----------------NNIPRGNLLQNLIDLMTSRMNSAK-HIKDCL
A0A1A7XZM1_9TELE    INVHSQLR-AATDMDDSIIDENGSELLPRVNMLQLLIRIISTATPRLQVLASQCL
A0A0P7V1U6_9TELE    IHEHAQLR-SHTDSLDLLFDEYGPEMLPRVNMLVLLTKVISTGAPRLQVLASHCL
A0A1A7WWE1_9TELE    INVHSQLR-AATDMDDSIIDENGSELLPRVNMLQLLIRIISTATPRLQVLASQCL
                    :                                      :
```

```
GCN1_YEAST           NALCQSISVA-----PNQEDLDMILSNLLSPNQFVRSTILETLDN---------- 1375
GCN1_SCHPO           LSLCEGLHST-----YTDEELNLLLSNLFHPESSIRSAVLQALQA----------
GCN1_DICDI           FQLIEG---------VETSDIGELMEGIISKHVQVRSICLQAIEKIPSIYSPSFV
GCN1_MOUSE           TALCASSSGEDGCAFAEQEEVDVLLAALQSPCASVRETALRGLMELRL-VLPSPD
GCN1_HUMAN           TTLCASSSGDDGCAFAEQEEVDVLLCALQSPCASVRETVLRGLMELHM-VLPAPD
M7ANV9_CHEMY         TALCTSSSGEDGCAYAEQEEIDVLLQALQSPYMNVRDAALRGLMELQM-VLPTPD
H8X4E7_CANO9         LSLCQYIAVN-----ISDEDLQLLLENVVSPHVFVRSTILEGLDS----------
W6A2P0_ICTPU         TALCLSAGGQDSCALAEQAEIDVLLEALLSPCFSVRDAALRGLLELEL-ALPT-D
B6JZI6_SCHJY         ISFAQSISSE-----YTEEELQLLLSKVCASDSSLRTAVLQTLQC----------
R4WJK2_RIPPE         LDLAQASSGAEGCAVASSEEIDTLLTALQQSSSHVRDAALRALLAITQ-VFPKPK
W8CCK9_CERCA         LEVANLSSGEDYCGFASEDEIKLILNFLQCDKQVVREACLRALQIIKRSILLENV
B0W357_CULQU         LDVAESSSGSEYTAKAEHREIEVLLVALQDELGAVRDVALRALAIMIK-VLPSIA
E1ZX97_CAMFO         LDVAQSGSGQPGTAIATSEDIDSLIGALQNSLSTVRDAALRGLTVIRQ-AFPSQK
W5JJ28_ANODA         LDVADSSSGREYRARADEREIECLLVALQDDLEAVRDVALRALAIMID-VLPSIA
F1KPR4_ASCSU         RSLCQLVDDAPRREVRMVQMLEELLSKLTLTKTNVRENVL-SILSVPHQLYQYAL
V9K7F0_CALMI         TSLCASSSGEEGCAYAEQEEIDVLLQALLSPCSSVRDAALRGLLEMNM-VLPTPD
W0T7Q3_KLUMA         MTLCQNISMT-----PTEEDLNLLLSSLLTPNQFVQATILEALDD----------
S9R951_SCHOY         LSLVQGIGAS-----YTDNELKLLLEHTCHNETSVRSAVLQSLQV----------
S9W134_SCHCR         LSLVQGIRTS-----YTDNELNILLQHACHNESSVRSAVLQSLQV----------
H0GUK0_SACCK         NALCQSISVA-----PNQEDLDIILSNLLSPNQFVRSTILEILDN----------
E5R2E4_ARTGP         SDLCRAMAAN-----IQPEELEILLQGSISREIPVRTAVLQSVLS----------
J8Q888_SACAR         NALCQSISVS-----PNQEDLDMILSNLLSPNQFVRSTILETLDN----------
C5G992_AJEDR         FDLCRCIAQN-----IEQEELEVILKASIVPEISVRTSVLQAILS----------
G4MR95_MAGO7         SDMVRCVAPN-----ISEKEIGVVARGAIVPQTSVRTTVLQSISA----------
C9SQ16_VERA1         ADACRCIAPN-----ITSEEIGVLARGSLVPQVSVRSTVLQSISA----------
B2WNI9_PYRTR         TDLASGLAPN-----ISNEELGALLRGTIVPETGVRTATLQAIDA----------
S3CN37_OPHP1         SDMVRCVAPN-----INQDETAILAQGIIVPQASVRSTVLQSISA----------
G3ALT0_SPAPN         LSLCQHVSIN-----ISESDLQLLLSNIVTPEVFVRSTILEGIDA----------
Q7S5P0_NEUCR         SDMVRCIAPN-----ISAEEIAVLSRGAIVPQVAVRTAALQAISS----------
A0A165FVX9_9PEZI     TDSCRFLAPN-----IAEPELQIVLRGVILPEIPVRTALLQAIHS----------
A0A178DN93_9PLEO     TNLASGLAPN-----ITNEELASLLRGTIVPEPAVRTATLQAIDT----------
A0A177D7J2_ALTAL     TNLASGLAPN-----IDNDELGALLRGTIAPETGVRTAILQAIDA----------
A0A0C4DS68_MAGP6     SDMVRCLAPN-----INEKEIGVVARGAIVPQTSVRTTVLQSISA----------
A0A151N4J5_ALLMI     TALCTSSSGEDGCAYAEQEEIDVLLQALQSPCMNVRDAALRGLMEMQM-VLPTPD
A0A146NKU7_FUNHE     TALCASAGGGDGCTVAEQQEIDVLLNALLSSCFSVRDAALRGLVEMEF-ALPT-D
A0A0F7Z917_CROAD     TALCASSSGEDGCAYAEQEEINVLLQALQSPCMNVRDAALRGLMELQM-VLPTPD
A0A0W0DC37_CANGB     NTLSQSISVH-----PTENDLKIIISSLLSGHSFVRTTILEILDN----------
A0A0L8RIK7_SACEU     NALCQSISVA-----PNQEDLTIMLSNLLSPNQFVRSTILETIDN----------
A0A178FRD2_TRIVO     SDLCRAMAAN-----IQPDELEVLLRGSISREIAVRTAVLQSILS----------
A0A175WD14_9PEZI     ADMVRCVAPN-----LLPEEIYVLARGTIVPQASVRTAVLQAISA----------
A0A194VFU1_9PEZI     ADLVRCIAPN-----ITHDELAVLARGVIVPQASVRTTVLQSISA----------
A0A151TXV6_CAJCA     NELSLG---------LQPAEVASALYGVYAKDVHVRMACLNAVKCIPAVANRSLP
A0A0M9ABE9_9HYME     LDVAQSGSGQSGTAIATNEDIDSLIGALQNSLSTIRDAALRALIVVRQ-AFPSQK
A0A072U5F0_MEDTR     NELSLG---------FQPDEVASALYGVYAKDVHVRMACLNAVKCIPAVSSRSLP
A0A0K8V1F4_BACLA     LEVANLSSGEDYCAYATDDEIELFLNSLENSKESVREVALRVLKIIKKVILINNE
A0A0A1XE05_BACCU     LEVANLSSGEDYCTYATDDEIALFLNSLESGKESVREVSLRALKIIKKVILFETE
A0A131YTA4_RHIAP     VDVCFSISGLENCSRADVAEVKVLLGALYSPELVVRETALQCLLMLQL-VVPTHE
A0A0J6I2R9_COCPO     IDICRSVTDN-----VQPDELTILLQGVIVPETSVRTAVLQAIEA----------
A0A074XMM5_AURPU     TDVCRSVAPN-----ATEAETDVVVKATIVPESSVREVALQAISA----------
A0A167FV98_9ASCO     VAIIQNVSGT-----ISETEINVLLSNVLSGDVFVRTTLLEVIDG----------
A0A1A7XZM1_9TELE     TALCVSAGGGDGCAVAEQPEIDVLLDALLSPCFSVRDAALRGLLEMEF-ALPT-D
A0A0P7V1U6_9TELE     TSLCASAGGGEGCALAEQEEIDVLLEALLSPCFSVRDAALRGLLELEL-ALPT-D
A0A1A7WWE1_9TELE     TALCVSAGGGDGCAVAEQPEIDVLLDALLSPCFSVRDAALRGLLEMEF-ALPT-D
                                                       ::   *  :
```

```
GCN1_YEAST           -EFE-LEPFMKYSPEVFICRFDSDPSN-REIADFIW--EFNKFVVNDEL-LKSLF 1430
GCN1_SCHPO           --FD--LSRFEFIKEIFLELYDDNETN-ASIAHQIS--TQNGLDATETS-FFELQ
GCN1_DICDI           WEDK-------YIGSLWFARFDNHDANTSALAEKIWLATNQPTQLPEDF-MKLLS
GCN1_MOUSE           TDEK---SGLSLLRRLWVIKFDKEDEI-RKLAERLW--STMGLDLQSDL-CSLLI
GCN1_HUMAN           TDEK---NGLNLLRRLWVVKFDKEEEI-RKLAERLW--SMMGLDLQPDL-CSLLI
M7ANV9_CHEMY         SDEK---NGLNLLRRLWVVKFDVEDEI-RKLAETLW--ESMGLELQPDL-CSLLI
H8X4E7_CANO9         -EFE----LSAYSKEVYVATYDTDNNC-RELAQTIW--EDNSLSVPDTT---NLL
W6A2P0_ICTPU         SAEP---NGLNVLRRLWVAKFDVEEEG-KALADKLW--QTLRLELVPEL-CPLLI
B6JZI6_SCHJY         --LD--LHRFDFIKEIFLELYDDTDAN-ASLAHDIS--KSNTFEADESS-LKELL
R4WJK2_RIPPE         QNAE---QAFRLTKRIWVAKFDVVTEN-RDVADKVW--ANANLSHDTKGLSEGLL
W8CCK9_CERCA         IHRN---LISDIKLRLWVAKHDISEEN-CILATDLW--ESLEFSF-PNI--EDIL
B0W357_CULQU         DDYE---LGLRLTRRLWVAKHDVCEET-KLLAEHVW--TQGEFEVPIVM-ADELM
E1ZX97_CAMFO         EDRD---QLDRLTRRVWIARFDVNDEN-KILASELW--NAADFTAHAEVLCEELI
W5JJ28_ANODA         DDYE---LGLRLTRRIWVAKHDVAEDT-KQLADTVW--VQGNYELPIVM-ADELM
F1KPR4_ASCSU         SNAECAHLVDLLIRRIYIARHDTNEDC-AKLASVIW--HNEGLQTNPEL-CTEVL
V9K7F0_CALMI         SDEK---NGLNLLRRLWVSQFDVDEES-RLQAKKLW--DTMGLELQPDL-CSLLI
W0T7Q3_KLUMA         -EYD-LQPYMSYSPEIYITCFSEDVNN-RDVANFIW--DSNQFKVTDEL-AQSLL
S9R951_SCHOY         --LD--LTQFSFIKEIFLELYDDNESN-AAVAHQIS--TQNGLDANEDS-YSELL
S9W134_SCHCR         --LD--LTQYSFIKEIFLELYDDNESN-AAVAHQIS--TQNGLDANEDS-YSELL
H0GUK0_SACCK         -EFE-LEPFMKFSPEIFICRFDSDPSN-RDVADFIW--EFNKFEINDEL-LKSLF
E5R2E4_ARTGP         -EID--LTDIDFSVYIWIAYHDSVAEN-AEVAKEIW--EENALDVDEQS-PDLII
J8Q888_SACAR         -EFE-LQPFMEYSPEVFICRFDSDSSN-REVADFIW--EFNKFSINDGL-LKSLF
C5G992_AJEDR         -ELD--LTDLDFSEYIWLGCHDNLAEN-RETAEVIW--EQNALDVDENS-ANLLV
G4MR95_MAGO7         -DVD--MSELEFSEEIWIAYHEDSEEN-VELAKEIW--EESGFQTSKDV-PVKML
C9SQ16_VERA1         -EVD--MSEAVFSTEIWLACHDDVEEN-SELGREIW---TEGFEVSEKL-AFEML
B2WNI9_PYRTR         -ELD--MNDLTFSEEIFIACHDDVPEN-AELARTIW--DENDLELKPDA-GVRML
S3CN37_OPHP1         -EVD--MSELPTSEEVWLACHDDIEEN-VDVGHEIW--DESEFKVTEDL-PERTL
G3ALT0_SPAPN         -EFD-LQGEIEYSNELWVATHDNDSNC-KELANTIW--EDNNLHIIPET-PKKLL
Q7S5P0_NEUCR         -DVD--MSELSTSEEIWLACHDDVPEN-ADLGRDIW--EESEFQVTEEL-AFKML
A0A165FVX9_9PEZI     -ELD--LTDFEFSEEIWLAYHDDVSEN-RELAQAIW--AENGLEVDQRS-GIKMI
A0A178DN93_9PLEO     -ELD--MNDLDFSEEIWIACHDDVPEN-VELARTIW--EENELQLTPDA-GIKML
A0A177D7J2_ALTAL     -ELD--MNDLKFSEEIFIACHDDEPEN-AELARTIW--EENDLELKADA-GVQLL
A0A0C4DS68_MAGP6     -DVD--MSELEFSEEIWLACHEDNVEN-VDLGREIW--EESGFEISEDV-PTKML
A0A151N4J5_ALLMI     SDEK---NGLNLLRRLWVVKFDVEDEI-RKLAERLW--ESMGLELQPDL-CSLLI
A0A146NKU7_FUNHE     STES---SGMSLLRRLWVARFDVEEEG-RLLAEKLW--ESLGLELVPEL-CSMLI
A0A0F7Z917_CROAD     SDER---NGMNLLRRLWVVKFDREEEI-RLLAERLW--TSMALELQSDI-CSILI
A0A0W0DC37_CANGB     -EYE-LQEFMKFSPEIFMCRFDSDDNN-RELANFIW--EFNKFEIVPEL-LDGLL
A0A0L8RIK7_SACEU     -EFE-LEPFLKYSPEVFICRFDSDPSN-RETADFIW--EFNKFCINDKL-MKSLL
A0A178FRD2_TRIVO     -EID--LTDIDFSVHLWIAYHDNVAEN-AEIAREIW--EENALDVDEQS-PDLII
A0A175WD14_9PEZI     -DVD--MSEVGVSEEIWLACHDDEEEN-VDLGREIW--EESEFQTSEEL-AHKML
A0A194VFU1_9PEZI     -EVD--MSDLSFSEEIWLACHDDVDEN-VELGKEIW--QESEFETSNEA-ALKMI
A0A151TXV6_CAJCA     ENSE-------VATSIWIALHDPEKSV-AQVAEDIW--DHYGFDFGTDF--SGLF
A0A0M9ABE9_9HYME     EDSV---QLSHLVRRIWIAKFDICDEN-KILANELW--TAADLVMHADKLADELI
A0A072U5F0_MEDTR     QNTE-------VATSIWIALHDPEKSV-AEVAEDIW--DHYGFDFGTDF--SGIF
A0A0K8V1F4_BACLA     MHQN---LISRLKLLLWIAIYDTSDEN-RILASDLW--EAAKFSP-PEL--DDVL
A0A0A1XE05_BACCU     AHRN---IISRLKLRLWIAKFDISVEN-RILASDLW--ESVKFSP-PDL--DDVL
A0A131YTA4_RHIAP     TDPE---NSLELAARLWVARFDSKEEI-KALAEKLW--RELKLEPEPSL-CARLL
A0A0J6I2R9_COCPO     -EID--LTDLDFSEYIWLGCHDHVSEN-AEISKAIW--EENGLEVDANS-PDFIM
A0A074XMM5_AURPU     -EFE--LSGKDFYDEIFLATHDDVQEN-VELAHEIW--VENELKTSPES-ATSIL
A0A167FV98_9ASCO     -ELDNCLVDRGFSSEIWIERFDDELVI-ANLATEIW--QYNEFKTDEQT-PRKLL
A0A1A7XZM1_9TELE     STEA---SGMSLLRRLWVARFDVEEEG-RALAEKLW--ESLDLELVSDL-CSMLI
A0A0P7V1U6_9TELE     SSEG---SGLSVLRRLWVARFDVEEEG-RMLADKLW--QSLCLELVPEL-CALLI
A0A1A7WWE1_9TELE     STEA---SGMSLLRRLWVARFDVEEEG-RALAEKLW--ESLDLELVSDL-CSMLI
                                    ::.  ..         .  :
```

```
GCN1_YEAST          PLFNQDDSGLRLFAANAYAFGAVSLFTSE--ENSSK-DYLNDLLNFYKEKAKPLE 1485
GCN1_SCHPO          IFFTQDSDYLQQIIGKSLI-DLLDEFEEL--GQFI----PKELMRTYRENALPSA
GCN1_DICDI          DSTFNVNSETRKINALAIK-EAATCHTHM--IPEI----VDNLFEIYEQNYP---
GCN1_MOUSE          DDVIYHEAAVRQAGAEALS-QAVARYQRQ--AAEV----MGRLMEIYQEKLYRPP
GCN1_HUMAN          DDVIYHEAAVRQAGAEALS-QAVARYQRQ--AAEV----MGRLMEIYQEKLYRPP
M7ANV9_CHEMY        KDIIYYEEAVRQAGAEALS-KAVEQYRNQ--AAEV----MSKLMEIYQEKLYRPP
H8X4E7_CANO9        ELFGLQDPGLRMSVAQAYRDASIQTHIN----------LEELFNFYIEKKNPPA
W6A2P0_ICTPU        KDVTHHEETVRVAVAEALS-SAVSQYKEE--SATV----LSQLTELYQQKLYRPP
B6JZI6_SCHJY        PFLDNESAYVHEILGKALC-DLIDDYEEF--STSI----PRELMSNYRVKALPTP
R4WJK2_RIPPE        DDVVHPVAEIQQAAASALA-ALLQTGSSQTTVDVV----LQLLLNIYNDKNNMVP
W8CCK9_CERCA        IDVTHNEVCIQRAASAAMV-PLLFKDYSQ--VKSI----VKSLLGIYKEKLIMIP
B0W357_CULQU        KDIIHPEPCIQKAASFALV-SILAEDSSI--IDSI----VEQLLEIYQEKLTMIP
E1ZX97_CAMFO        QDIAHPVEPVQQAAAHALA-ESLASVPHL--TPSV----LDNLLQLYQEKLAMIP
W5JJ28_ANODA        KDIVHPELCIQKAAAGALV-SILMEDAST--VDGV----QEQLLEIYREKLTMIP
F1KPR4_ASCSU        DDVTSVEEFMRKSASHALE-SLIVAYPEK--LDVV----LLRLDALYSELSEMRG
V9K7F0_CALMI        KDVIYHEEAIRHAGAEALS-NAVAQYQSQ--AAQV----MTKLIEIYQEKLFRPP
W0T7Q3_KLUMA        KFFEQTDSGLRLFTAKAYA-SAVAQLEKE--NPGSFTRYFHTLLDFYVQKAEPPK
S9R951_SCHOY        PLLNVKSNYLHTLVGKSFV-DLIDEFEDF--STIL----PQKLMEMYNKNALPSP
S9W134_SCHCR        PLLDVKSNYLHTLIGKSFV-DLIDEFEDF--SSVL----PKKLMEIYSKNALPSP
H0GUK0_SACCK        SLFNQDDSGLRLFAANAYAFGSVSLFTSE--GSSSN-TYLDALMSFYKQKAKPLE
E5R2E4_ARTGP        KHLANDDLPLRSAAALALA-HACQLCPSI--FPDT----LKKLESMYREQVHSKP
J8Q888_SACAR        LLFNQDDSGLRLFAANAYAFGAVSLFTSE--GSSSPNTYLNELMEYYKEKAKLLE
C5G992_AJEDR        KYLDSKDSQLRGAAARALA-HACEVSPAV--FTDI----LEKLQSKYREEVRPKA
G4MR95_MAGO7        PYLESKDGQLRKAASRALA-EACSNHPET--VNLI----LEKLRLAYVEFAKPRV
C9SQ16_VERA1        PYLSSKDGQLRRAAARSLA-EASSVHPQV--IEPL----IEKLEETYAELAKPRQ
B2WNI9_PYRTR        PYLDSLDKQLRRAAARSIG-EIITKFPDT--FQDL----LQRLRESYTEKAKPRV
S3CN37_OPHP1        PYLESKDAQLRRAAARALA-ESISTLKSTKIFDAI----TEQLRSSYTEQAKPRV
G3ALT0_SPAPN        ELFGNHDSGLRVSIATAYV-DAVNQLRQQ--EESVLGSCLDDLIELYHEKKNPPA
Q7S5P0_NEUCR        PYLESKDGQLRRAAAKGLA-EALGQNLSA--VNPI----LEKLRESYTELAKPRL
A0A165FVX9_9PEZI    PYLRNVDKQLRRAASRALA-HAVELNPSS--CAEI----LARLEDEYREAAKPRV
A0A178DN93_9PLEO    PYLDSLDKQLRRAAARAIG-EIITQFPDT--FERL----LQNLRESYVEKAKPKL
A0A177D7J2_ALTAL    PYLDSLDKPLRRATARAIG-EVITKFPDT--FDDI----LRRLRETYAEKAKPRV
A0A0C4DS68_MAGP6    PYLESKDNQLRRAASRALA-EACKRHRKT--VNPI----LEKLRLSYVEFAKPRV
A0A151N4J5_ALLMI    KDVIYYEEAVRQAGAEALS-KAVAQYRDQ--AADV----MNKLTEIYEEKLFRPP
A0A146NKU7_FUNHE    GDITHHEEAIRTAAAEALS-SAVSQYREQ--AATV----LGQLTELYHQKLYRPP
A0A0F7Z917_CROAD    NDVIHHEQAVRQAGAEALS-KGVEQYRSQ--TRDV----MAKLMEIYQEKLYRPP
A0A0W0DC37_CANGB    SLFYQQDSGLRLFLARGFAYATYNVKGEE--YLEQ---SLKMLMNFYNEKAQPLT
A0A0L8RIK7_SACEU    PLFNQDDSGLRLFVANAYAFGAITISTSE--HRSPD-VYLNNLMDFYKENARPLE
A0A178FRD2_TRIVO    KHLANDDLSLRSAAAIALA-HACELCPSI--FSDT----LKKLESMYREQVHTKP
A0A175WD14_9PEZI    PYLESKDAQLRRSAAKSLA-EIVNHHPTV--IDPV----LDKLRASYTELAKPRV
A0A194VFU1_9PEZI    PYVQSKDVQLRRAAARSLA-EAVQLHSST--MQEV----LEQLRSSYIELAKPRK
A0A151TXV6_CAJCA    KALSHLNYNVRVAAAEALA-AALDEHPES--IQET----LSTLFSLYIRDMGVGD
A0A0M9ABE9_9HYME    QDIAHPVEPVQQAAACALA-QCLTEVLPL--VPII----LDKLLQLYQEKLTMIP
A0A072U5F0_MEDTR    KALSHVNYNVRLAAAEALA-AALDEHPDL--IQES----LSTLFSLYIRDMGIGN
A0A0K8V1F4_BACLA    LLVTHKELCIQKAASASLV-QLLSTNHQQ--VKYT----VKSLLEIYREKLTMIP
A0A0A1XE05_BACCU    LVVTHKELCIQKAASASLL-ELLSTNHQE--IKYT----IKSLLEIYREKLTMIP
A0A131YTA4_RHIAP    SDVMHPQAETRSTAAQALK-SLLQAFPDC--LHDV----LLQLMDAYRQHLQRPE
A0A0J6I2R9_COCPO    EYLGTADSQLRGAAAVALA-HACKFSPSV--FTTT----LEKLETKYQDEIKPRA
A0A074XMM5_AURPU    KYLASHDIQLRRAAARSLA-ALVQEHSSI--SETV----ISNLQDTYKEDAKPRA
A0A167FV98_9ASCO    EFLGSEHASLRHACARSIA-ASVGANKTL--ASAT----YQSLINLFIEKSKPPP
A0A1A7XZM1_9TELE    GDITHHEEAIRSAAADALS-SAVSQYREQ--SASV----LGQLTDLYHQKLYRPP
A0A0P7V1U6_9TELE    DDVTHHEGAVRAAGAEALS-NAVSCYREQ--APAV----LARLTELYHQKLYRPP
A0A1A7WWE1_9TELE    GDITHHEEAIRSAAADALS-SAVSQYREQ--SASV----LGQLTDLYHQKLYRPP
                              :   . .                         *   :
```

```
GCN1_YEAST           PILDQFGLVLVSASEQKDPWQGRSTVAITLKIMAKAFSAEDDTVVNIIKFLVDDG 1540
GCN1_SCHPO           PEYDEYGIIKKETIGRDLGRIARESVAVSFFHISKYLSSNL--LLPFLEFLLTAS
GCN1_DICDI           ---DEI-------RETPITSKFRISVATALSGLGNAIVEPE-VLKSLFTKIIERG
GCN1_MOUSE           PVLDALGRVIS--ESPPDQWEARCGLALALNKLSQYLDSSQ--VKPLFQFFVPDA
GCN1_HUMAN           PVLDALGRVIS--ESPPDQWEARCGLALALNKLSQYLDSSQ--VKPLFQFFVPDA
M7ANV9_CHEMY         PVLDALGRMIS--ESPPDQWEARCGIALALNKLSQYLDSSQ--VKPLFQFFVPDA
H8X4E7_CANO9         PKLDEFGLVIKSTIDQRDRWEERSTIALALKFLVPHLSETD--VEKLFKFLVEEA
W6A2P0_ICTPU         PVLDALGRVIS--EAPPDQWEARCGIALALNKLSQWLREDQ--VTPIFQFFVPDA
B6JZI6_SCHJY         PEYDEYGIIVKDTIGRDLGRSSREAIATCFAHVVKVMASNI--LIEFLEFLLTAT
R4WJK2_RIPPE         ARVDSLGRIV---EKPIDTWEPRSGVALALAQLAPLLTPDK--VADLAKFYVEKG
W8CCK9_CERCA         PKLDQFDREV---FPAIDQWEPRRGIAICISQISKFFDVED--VNEIMQFMVSQG
B0W357_CULQU         AKLDQFDREI---EPAIDPWGPRRGVAVALSQCASFLTADL--VNTLTQFMVATG
E1ZX97_CAMFO         PKLNDFGRVV---EQPIDTWGPRRGVALALAQIAPLLTADT--VHRLIQFFVLTG
W5JJ28_ANODA         ARLDQFDREI---EPAIDPWGPRRGVAIAFCHIAPFLTPEL--VKSLIEFMVRHG
F1KPR4_ASCSU         AVIDDVGRMV---KEPVDQWERRAGVGEALILLAAHIPESA--AVTFVKIVVPRG
V9K7F0_CALMI         PVLDALGRVIS--ESPPDQWEARCGIALALNKLSEYLDGSQ--VKLLFQFFVPEA
W0T7Q3_KLUMA         DILDEYGLVKVSAMDQKDPWEARSTTAIALKELCSSFEAYDGSVVEFIHFLIDSG
S9R951_SCHOY         PEYDEYGIIKKETVGRDLGIVPREGVSIIFVHISQYLSSNL--LVPFLEFLLSSV
S9W134_SCHCR         PEYDEYGIIKKETIGRDLGIVTREGVSIIFVHISQYISSSL--LVPFLEFLLSSV
H0GUK0_SACCK         AILDQFGLVLVSASEQKDPWQGRSTVAITLKIMAKALSAEENTVVSVIKFLVDDG
E5R2E4_ARTGP         VQTDAYGMPRK--AEQADAWEIRSGIALSFGAMASGFSGDD--IVSFFRFLIDDG
J8Q888_SACAR         PILDKFGLVLVSASEQKDPWQGRSTVAITLKIMAKALCAEDNTVVNVIKFLVDDG
C5G992_AJEDR         PEKDAYGMPKK--IDGQDKWEPRSGIALAFGAMAKGYQKDE--IVTLLRFLIDEG
G4MR95_MAGO7         PELDEFGMPKK--MDLSDPWEARHGIASSFKELAPYLKREH--LDSFFAFLIEQG
C9SQ16_VERA1         QELDQYGMPKK--LDLADPWEGRHGIGSAFKELGPHMKKQQ--LDPFFDFLIQKG
B2WNI9_PYRTR         PERDEYGMPRK--IDLRDPWESRDGIALTFKEMTPGFKPDD--LVDFLNFLIFEG
S3CN37_OPHP1         AKLDEFGMPRK--MDLSDPWEARHGIAAAFRELVPSLDKNQ--VEPFLHFLIEQG
G3ALT0_SPAPN         PKLDKFGLVIKSTIDQRDRWEERSTIALALKLLAPVFDKEC--IEKLFNFLVHDQ
Q7S5P0_NEUCR         PELDEFGMPKK--KDLSDPWEARQGLALAFQGIAPLLQKNQ--LEPFFAFLIDNG
A0A165FVX9_9PEZI     PELDRYGMPKR--MDLSDPWEARHGIALAFKELATSFDTAQ--LVSFVQFLIENG
A0A178DN93_9PLEO     PERDEYGMPRK--VDLRDPWESRDGIALAFKEMTPGFSPET--LVDFMTFLVHGG
A0A177D7J2_ALTAL     PERDEYGMPRK--VDLRDPWESRDGIALTFKEMTPGFKPDD--LVDFLNFLIYEG
A0A0C4DS68_MAGP6     PELDEFGMPKK--MDLSDPWEARHGIASAFKELSPYLERQH--LDAFFALLIEQG
A0A151N4J5_ALLMI     PVLDALGRVIS--ESPPDQWEARCGIALALNKLSQHLDSSQ--VKPLFLFFVPDA
A0A146NKU7_FUNHE     PVLDALGRVIS--ESPPDQWEARCGIALALNQMSQYLDEAQ--VTPLFLFFVPDA
A0A0F7Z917_CROAD     PVLDALGRVIL--DSPPDQWEARCGIALALNKLSQHLESSQ--VKPLFQFFVPDA
A0A0W0DC37_CANGB     AILDEFGLVVVPASQRKDLWEERSTAAIALKELSPGLPSDGDTVVSVIEFLIAGP
A0A0L8RIK7_SACEU     PILDQFGLILVPANEQKDPWQGRSTTAITLKIMAKALSADENTVVNIIKFLVDDG
A0A178FRD2_TRIVO     VQTDSYGMPRK--AEQADAWEIRSGIALSFGAMASGFSGDD--IVSFFRFLIDEG
A0A175WD14_9PEZI     QLLDEFGMPKK--MDLSDPWGARHGIALAFKHLASHLPKLQ--LDPFFNFLIEQG
A0A194VFU1_9PEZI     QELDAFGMPKK--MDLSDPWEARHGIACAFKELAPHLEISQ--LDGFFNYLIEQG
A0A151TXV6_CAJCA     DNVDA-------------GWLGRQGIALALHSAADVLRTKD--LPVVMTFLISRA
A0A0M9ABE9_9HYME     PKLNDFGRVV---EQPIDTWGPRRGVALALAQMAPLLSADT--ILKLVQFFVSTG
A0A072U5F0_MEDTR     DNVDA-------------GWLGRQGVALALHSAADVLRTKD--LPVVMTFLISRA
A0A0K8V1F4_BACLA     PKLDQFDREI---FPAIDQWEPRRGISISISQVSKFFDVDD--VNEIMQFMVAQG
A0A0A1XE05_BACCU     PKLDQFDREI---FPAIDQWEPRRGIAISISQISKFFDVDD--VNEIMQFMVSQG
A0A131YTA4_RHIAP     PVRDSFGRVIM--ERPPDIWEPRSGVGLALGQLAPLVPSPA--VPELMEFYVHEA
A0A0J6I2R9_COCPO     PETDAYGMPKK--VDTPDNWQLRSGIALALKSMAQGFHGDQ--IVGFLQFLISDG
A0A074XMM5_AURPU     PQRDKYGMPIK--MDLSDPWQSRQGIALAFNQLTPTYGSEQ--LVPLMQFMVAEG
A0A167FV98_9ASCO     PVYDKFGILVK--SSNEDPWYTRSGIAKALQEFAPLLQETS-LVIEMFEFLINTG
A0A1A7XZM1_9TELE     PVLDALGRVIS--EAPPDQWEARCGIALALNKLSQYLNEPQ--VTPLFLFFVPDA
A0A0P7V1U6_9TELE     PVLDALGRVIS--ESPPDQWEARCGIALALNKLAQYLDESQ--VTPLFLFFVPDA
A0A1A7WWE1_9TELE     PVLDALGRVIS--EAPPDQWEARCGIALALNKLSQYLNEPQ--VTPLFLFFVPDA
                        :                  *   .  :                 .    :
```

```
GCN1_YEAST          ------GLVDREPIVRQEMKEAGVELITLHGSQNSKDLIP--IFEEALSSSTDSA 1595
GCN1_SCHPO          EAEAQIPVTDASQKVSSKMLEAGKLAIFQSGAHQVEALME--LFEQKLNVDSLPT
GCN1_DICDI          -------LFDPKEEVVQEFVSTGMSIINQQGVQFSGELLA--TFEAFLARPDNGT
GCN1_MOUSE          -------LNDRNPDVRKCMLDAALATLNAHGKENVNSLLP--VFEEFLKDAPN-D
GCN1_HUMAN          -------LNDRHPDVRKCMLDAALATLNTHGKENVNSLLP--VFEEFLKNAPN-D
M7ANV9_CHEMY        -------LNDRNTEVRKCMLDAALSTLNTHGKDNVNSLLP--VFEEFLKNAPN-D
H8X4E7_CANO9        -------LGDKDQNVRQQYQDSGVVAIEAHGAENVEALIR--IFEESLQAKN---
W6A2P0_ICTPU        -------LNDRHSEVRRCMLDAALSVLNTHGKDNVNSLLP--VFEEFLKNAPQ-D
B6JZI6_SCHJY        EVDSQIPVTDVSVTVASTMLEAGKVAIELHGKHQVESLMS--FFEESLQRVDSSS
R4WJK2_RIPPE        -------LGDRNETVRHNMLTAALAAVDLHGKDTVNTLLP--IFEPVLDKGPD-C
W8CCK9_CERCA        -------FRDRNEIVQKEMLAAALCIVDCHGKQTILNLLP--VFEGFLDKAPR-S
B0W357_CULQU        -------LRDREEIVHKEMLAASLAI----------RLVPPSTFEEFLDKAPN-N
E1ZX97_CAMFO        -------LGDRNQFVRTEMLTAAVAAVDLHGSANITSLLP--VFENFMDKAPK-I
W5JJ28_ANODA        -------LRDRSEIVQKEMLAASLGIVEHHGKDSVAYLLP--TFEQFLDKAPS-H
F1KPR4_ASCSU        -------LSDRNAECRDLMRNTAIEAIKKYGEARMSELLP--FLEGLLHSTPD-G
V9K7F0_CALMI        -------LNDRHAEVRKCMLDAALSALNTHGKDNVNSLLP--VFEEFLRNAPN-D
W0T7Q3_KLUMA        ------ALGDKEELVRQEMKEAGIEVVDYYGSKYLKELMP--IFESFLSTSNDVV
S9R951_SCHOY        QPDAKIPVTDSSVIVASNMLEAGKQSIVAFGSYQVEALME--LFEAKLNVDTLPT
S9W134_SCHCR        QSDAQIPVTDSSVTVASNMLVAGKQAITAFGSYQVEALME--LFETKLNVDTLPT
H0GUK0_SACCK        ------GLVDREPIVRQEMKEAGVELITLHGSQNSEELIP--IFEEALSSSRDSA
E5R2E4_ARTGP        ------PLIDRSAFVRRQMAESGSSVITLRGREKVEELMS--IFETTLETSDKET
J8Q888_SACAR        ------ALVDREPIVRQEMKEAGIELITLHGSQNSEELIP--IFEEALNSSTDSA
C5G992_AJEDR        ------PLIDKSAFVRRQMAESGSTVITLRGGEKVEQLMQ--LFEKTLETSDKAS
G4MR95_MAGO7        ------PLGDQNGSVRAEMLEAANKAIEIHGKGMVDKLMK--TFETTLEAPDKGS
C9SQ16_VERA1        ------PLGDQNAAVRSEMLEAAISAIDYHGKSMIDRLMV--AFENNLEGPDKST
B2WNI9_PYRTR        ------PLGDRSPAVRDELIEAATSVITVKAQTKVEPLME--LFENALEAPDRKS
S3CN37_OPHP1        ------PLGDQNGTVRQEMLEAAIKAIEVHGKPIVAKLMQ--TFEKTLSAPDKNS
G3ALT0_SPAPN        ------ALGDKEGLVRQELQEAGIETINLHGSKFVESLIP--IFEENLAAKNERS
Q7S5P0_NEUCR        ------PLGDQNGNVRAQMLEAANTAIEIHGKSILDKLMK--TFEKTLEAPDKGT
A0A165FVX9_9PEZI    ------PLGDRSSIVRQAMIDAAIAIIGAHGHTKLEELMK--IFETTLAAPDKGS
A0A178DN93_9PLEO    ------PLGDRSSAVRDEMIEAATSVITTKAKTEVEPLME--LFENALEGPDRKS
A0A177D7J2_ALTAL    ------PLGDRSPAVREELIEAATSVITVKAQTKVEPLME--LFENALEAPDRKS
A0A0C4DS68_MAGP6    ------PLGDQNSSVRAEMLEAANKAIEVHGKSIVDKLMK--VFEKTLEAPDKGS
A0A151N4J5_ALLMI    -------LNDRSPDVRKCMLDAALSTLNTHGKENVNSLLP--VFEEFLKNAPN-D
A0A146NKU7_FUNHE    -------LNDRHSEVRRCMLDAALSALNSHGKDNVSSLLP--VFEEFLKNAPQ-D
A0A0F7Z917_CROAD    -------LNDRHPEVRKCMLDAALSALNTHGKESVNSLLP--VFEEFLKNAPN-D
A0A0W0DC37_CANGB    -------LGDREPIVRQEMKEAGIEIINQHGAQKSSQLVP--LFESSLETIKDVN
A0A0L8RIK7_SACEU    ------GLVDREPIVRQEMKEAGVELITLHGSQKSEELIP--FFEEALSSSTNSA
A0A178FRD2_TRIVO    ------PLIDRNASVRRQMAESGSAVITSRGREKVEELMS--IFETTLETSDKET
A0A175WD14_9PEZI    ------PLGDQNASVRAEMLEAANTAIEIHGKTILDRLMK--TFEKTLEAPDRNS
A0A194VFU1_9PEZI    ------PLGDRNATVRSEMLEAANNAITIHGKAILDELMQ--TFERTLEVPDKGS
A0A151TXV6_CAJCA    -------LADPNADVRGRMINSGILIIDKNGKDNVSLLFP--IFENYLNKTAPDE
A0A0M9ABE9_9HYME    -------LGDRNQAVRTEMLTAAVAVVDLHGKANITSLLP--VFENFMDKAPK-I
A0A072U5F0_MEDTR    -------LADLNADVRGRMINSGILIIDKNGKDNVSLLFP--IFENYLNKTAPDE
A0A0K8V1F4_BACLA    -------FRDRNETVHKEMLASALCIVDCHGKETIVNLLP--VFEDFLDKAPK-S
A0A0A1XE05_BACCU    -------FRDRNETVHKEMLASALCIVDHHGKETIVNLLP--VFEDFLDKAPK-S
A0A131YTA4_RHIAP    -------LSDPHPVVHKTLLDAATALVDFHGNSKVNILLP--LFERFLDDAPR-D
A0A0J6I2R9_COCPO    ------PLVDQNVSVRRQMAESGSAVIALHGQDNVEELMH--LFEKTLETSDKAT
A0A074XMM5_AURPU    ------PLGDKSASVRDSMLDATTAIIAAKGNLQVETLMK--LFESTLEGGS-GS
A0A167FV98_9ASCO    -------LGDQNSTVHSELLEAGLAVINTRGLNNVESLIP--IFESYLAKSDK--
A0A1A7XZM1_9TELE    -------LNDRHAEVRRCMLDAALSALNTHGKENVSSLLP--VFEEFLKNAPQ-D
A0A0P7V1U6_9TELE    -------LNDRHAEVRRCMLDAALSGLNAHGKDNVSSLLP--VFEEFLKSAPQ-D
A0A1A7WWE1_9TELE    -------LNDRHAEVRRCMLDAALSALNTHGKENVSSLLP--VFEEFLKNAPQ-D
                           . *           :               *.    :*  :
```

```
GCN1_YEAST            -----LKENVIILYGTLARHLQQSDARIHTIIERLLSTLDTPSADIQQAVSACIA 1650
GCN1_SCHPO            DANDRLREATVVLFGTVAQHLPSNDPRLAVVMDSLLSVLSTPSESVQLAVAVCLP
GCN1_DICDI            GEEDSIRANVVVYMGALAKHMDASNPKVSIVIDKLVDALSIPSESVQVGISKCIA
GCN1_MOUSE            ASYDAVRQSVVVLMGSLAKHLDKSDPKVKPIVAKLIAALSTPSQQVQESVASCLP
GCN1_HUMAN            ASYDAVRQSVVVLMGSLAKHLDKSDPKVKPIVAKLIAALSTPSQQVQESVASCLP
M7ANV9_CHEMY          ANYDAVRQSVVILMGSLAKHLDKSDPKVKPIVAKLIAALSTPSQQVQESVASCLP
H8X4E7_CANO9          -----IKESIVVLYGTLARHLKPTDERLKVIFDRLMKSLDTPN--VQFAVAECIA
W6A2P0_ICTPU          ASYDSVRQSVVILMGSLAKHLDKSDPKVKPIVAKLITALSTPSQQVQESVAGCLP
B6JZI6_SCHJY          SFDDRLREAMIVLFGTVAKHLSSSDTRLVVVIDSLIATLSTPSESVQLAVANCLP
R4WJK2_RIPPE          SSYDAVRQSVVILMGSLARHLDKDDKKIKPIVAKLIDALSTPSQQVQEAVANCLP
W8CCK9_CERCA          QSYDNVRQAVVILMGSLARHLEPDDIRIEPIVRRLISALSTPSQQVQEAVANCLP
B0W357_CULQU          SDYDNIRQAVVILMGSLARHLDRDDARIQPIVNRLLTALSTPSQQVQESVANCIP
E1ZX97_CAMFO          GSFDSIKQSVVILMGSLARHLDKNDPRIKPIVMRLIAALSTPSQQVQEAVANCLP
W5JJ28_ANODA          SSYDNIRQAVVILMGSLARHLDREDPRIKPIVDRLLTALSTPSQQVQEAVANCIP
F1KPR4_ASCSU          PQHDNLRQGLVLMLGTLAQHLDPSNEKVRTITARLIETLSTPSQQVQEAVSKCLP
V9K7F0_CALMI          ASYDAVRQSVVILMGSLAKHLDKSDPKVKPIVAKLITALSTPSQQVQESVASCLP
W0T7Q3_KLUMA          -----MKENVVILYGSLARHLEPNDPRIRTIAERLLTSLQTPSEDLQKSISKCLA
S9R951_SCHOY          EANDRLREATVVLFGTVAQHLQENDQRLVVVLENLISALSTPSESVQLAVASCIS
S9W134_SCHCR          EANDRLREATVVLFGTVAQHLRENDQRLVVVIENLISALSTPSESVQLAVASCIS
H0GUK0_SACCK          -----LKENVIILYGTLARHLQERDPRIHTIIERLLSTLDTPSADIQQAVSACIA
E5R2E4_ARTGP          EQSDWLNEAVIILYGSLAQHLIAGDKRIQKVTRKLMDALSTPSETVQLAVAECLI
J8Q888_SACAR          -----LKENVIILYGTLARHLQESDARIHTIIERLLSTLDTPSVDIQQAVSACIA
C5G992_AJEDR          EQSDWLNEAVIVLYGSLARHLRSGDKRVDIVIGKLLAALSTPSETVQFAVAECLP
G4MR95_MAGO7          AAADRVNEAVIIMYGALARHLKAGDAKIPVVIERLIATLSTPSEAVQYAIAECLP
C9SQ16_VERA1          EAADRVSEAVIIMYGALAKHLKKGDAKLPIVVERLLATLSTPSETVQYAIADCLP
B2WNI9_PYRTR          EMYDQVNEAVIILYGALGRHLAAGDQRVPKVVQRLLATLSTPSETVQYAVAQCLP
S3CN37_OPHP1          DAADRVNEAVIIMYGALARHLKQGDAKIPVVIERLLATLSTPSETVQYAIAECLP
G3ALT0_SPAPN          KVQDSIKESVIILYGSLARHLETSDPRLKIIVDRLIKTLDTPSEKVQFAVSECIA
Q7S5P0_NEUCR          AFADRVNEAVIIMYGALARHLKHGDAKIPVVIDRLIATLSTPSETVQYAIAECLP
A0A165FVX9_9PEZI      EM-DTVNEAVIILYGALAGHLKRGDERIPKVTHRLLATLSTPSETVQYAVAECLP
A0A178DN93_9PLEO      EMYDQVNEAVVILYGALGRHLVSGDKRVPKVVQRLLSTLSTPSETVQYAVAQCLP
A0A177D7J2_ALTAL      EMYDQVNEAVIILYGALGRHLAAGDERVPKVVQRLLATLSTPSETVQYAVAQCLP
A0A0C4DS68_MAGP6      AASDRVNEAVIIMYGALARHLKAGDAKVPVVIERLLATLSTPSEAVQYAIAECLP
A0A151N4J5_ALLMI      ASYDAVRQSVVILMGSLAKHLDKSDPKVKPIVAKLITALSTPSQQVQESVASCLP
A0A146NKU7_FUNHE      ASYDSVRQSVVILMGSLAKHLDKNDPKVKPIVAKLITALSTPSQQVQESVAGCLP
A0A0F7Z917_CROAD      ASYDAVRQSVVILMGSLAKHLDKNDPKVKPIVAKLIAALSTPSQQVQESVASCLP
A0A0W0DC37_CANGB      -----TKENVIILYGTLAQHLDKSDKRIGSIIDQLLQTLDTPSLDVQQAVSSCIA
A0A0L8RIK7_SACEU      -----LKENVIILYGTLARHLQESDARIHTIIERLLSTLDTPSADIQQAVSACIA
A0A178FRD2_TRIVO      EQSDWLNEAVIILYGSLAQHLVVGDDRIQKVTRKLMDALSTPSETVQLAVAQCLI
A0A175WD14_9PEZI      EAADRVNEAVIIMYGALARHLKPGDKKIPVVIERLLATLSTPSETVQYAIAECLP
A0A194VFU1_9PEZI      EAGDRVNEAVIIMYGTLARHLEPGDAKIAIVIERLLATLNTPSETVQYAVAECLP
A0A151TXV6_CAJCA      EKYDLVREGVVIFTGALAKHLAKDDPKVHAVVDKLLDVLNTPSEAVQRAVSACLS
A0A0M9ABE9_9HYME      GSFDSIKQSVVILMGSLARHLDKDDPRIKPIVMRLIAALSTPSQQVQEAVANCLP
A0A072U5F0_MEDTR      EQYDLVREGVVIFTGALAKHLAKDDPKVHAVVDKLLDVLNTPSESVQRAVSACLS
A0A0K8V1F4_BACLA      QSYDNVRQAVVILMGSLARHLEADDTRIEPIVRRLISALSTPSQQVQEAVANCLP
A0A0A1XE05_BACCU      QTYDNVRQAVVILMGSLARHLDADDTRIEPIVRRLISALSTPSQQVQEAVANCLP
A0A131YTA4_RHIAP      QSYDQVRQSVVILMGTLARHLDKDDQKVKPIVNRLIDTLATPSQQVQEAVATCLP
A0A0J6I2R9_COCPO      EQSDWLNEAVIILYGSLARHLKSGDKRLQTVIKKLLAALSTPSESVQYAVSECLT
A0A074XMM5_AURPU      QTQDAVNEAVVILYGALARHLKAGDSRVPKVVQRLLTTLNTPSESVQYAVAQCLP
A0A167FV98_9ASCO      EKNDQISESVIILYGALARHLQSDDARLIKVVDRLLATLDTPSEDVQYAVSQCLP
A0A1A7XZM1_9TELE      ASYDSVRQSVVILMGSLAKHLDKSDPKVKPIVAKLITALSTPSQQVQESVASCLP
A0A0P7V1U6_9TELE      ASYDSVRQSVVILMGSLAKHLDKSDPRVKPIVAKLITALSTPSQQVQESVASCLP
A0A1A7WWE1_9TELE      ASYDSVRQSVVILMGSLAKHLDKSDPKVKPIVAKLITALSTPSQQVQESVASCLP
                                ::  *::. *:   : ::  :   *:  *  *.  :* .:: *:
```

```
GCN1_YEAST          PLVFQFKQKVGDYLGILMEKLLNPTVASSMRKGAAWGIAGLVKGYGISALSEFDI 1705
GCN1_SCHPO          PLVKKSLGKSKEYYELLSNKLMNST-SLADQKGAAYGLAGLVKGYGIKAFQDFNI
GCN1_DICDI          QLIPSFKKQGDRLIPMLLEKLKNSSGNYADRRGAAFGLAGSVKGLGIGSLKNYSI
GCN1_MOUSE          PLVPAVKEDAGGMIQRLMQQLLESD-KYAERKGAAYGLAGLVKGLGILSLKQQEM
GCN1_HUMAN          PLVPAIKEDAGGMIQRLMQQLLESD-KYAERKGAAYGLAGLVKGLGILSLKQQEM
M7ANV9_CHEMY        PLVPAIKDDAGGMIQKLMQLLLESD-KYAERKGAAYGLAGLVKGLGILSLKQQKM
H8X4E7_CANO9        PLVPSFKDELSSYFDTLFTTLFTGK-TLAKRRGAAFGIAGLVKGDGVRALSDYDV
W6A2P0_ICTPU        PLVPAIKEDAGDMVRKLLQLLLESD-KYAERKGAAYGLAGLVKGLGILSLKQQDI
B6JZI6_SCHJY        PLIKKYSDKNEDYMKKLTETLLSSS-SFAEKKGAAYGLAGLTKGVGIKAFKDFGI
R4WJK2_RIPPE        ALVPSIKDEAPILVNNLLERLLSSE-SYGERKGAAYGLAGIVKGMGILVLKQLNI
W8CCK9_CERCA        HLVPSVKEKAPEIIKKLLQLLVKSD-KYGERRGAAYGIAGIVKGLGILSLKQLDI
B0W357_CULQU        HLIPSVKEQAPQMVNKLMQQLIKSE-KYGVRRGAAYGIAGIVKGLGILSLKQLDI
E1ZX97_CAMFO        HLTDSIKEDAPKIVDNLMDQLLKSD-KYGERKGAAYGLAGLIKGMGILALKQLDI
W5JJ28_ANODA        HLIPSVKEEAPAMVKKLMQQLVKSE-KYGVRRGSAYGIAGIVKGLGILSLKQLDI
F1KPR4_ASCSU        PLVPAIRDSAKELVRTLSCLLVEAD-SYGERRGAAYGLAGLVKGLGMSAVRELEL
V9K7F0_CALMI        PLVPAIKDDAAGMVRKLLQLLLESD-KYAERKGAAYGLAGLVKGLGILSLKQQEI
W0T7Q3_KLUMA        ALVPLFKDSIQEYIDSSFQVLYDSKAPVQIRRGAAWGIAGLVKGYGIAALSEFDV
S9R951_SCHOY        PLIKKLLPKSKQYYDHLVSSLLNAS-SFAERKGTAYGLAGLIKGLGIKAFKDFNI
S9W134_SCHCR        PLIKKLLPKAKEYYDRLTTGLINAS-SFAERKGTAYGLAGLVKGLGIKAFKDFDI
H0GUK0_SACCK        PLVFQFKPKVGEYLNLLMEKLLNPTVAVSMRKGAAWGIAGLVKGYGISALSEFDI
E5R2E4_ARTGP        PLIRLDGTDASHFVQELLNQLFTSK-KYAARRGAAYGLAGIVRGKGILALRDFGI
J8Q888_SACAR        PLVFQFKPKVGEYLNLLMKKLLDPTIPAPIRKGAAWGIAGLVKGYGISALSEFDI
C5G992_AJEDR        PVVRLSSTETGTYIRDMLDQLLHSK-QYAARRGAAYGLAGIVAGKGISAFREYRI
G4MR95_MAGO7        PLVKACGNKSSKYFDEIMETLLTSK-NYAVQRGAAYGLAGLVLGRGIASLREFRI
C9SQ16_VERA1        PLVQACRDQTPKYFQQVLGVLLTSK-KYSEQRGAAYGLAGLVQGCGISTLKDHRI
B2WNI9_PYRTR        PLVRTSEQELPNYINQMMEQLLQSK-KYASRRGAAYGLAGIVRGKGLGVLKEYRI
S3CN37_OPHP1        PLVQTCGDKSSKYFTQMLDVLLSSK-SYAAQRGAAYGLAGLILGRGISVLREYRI
G3ALT0_SPAPN        PLVPSFESRLQEFFDLLTEKLFTGK-SLPIRKGAAYGIAGLVKGSGIKTLSSFDI
Q7S5P0_NEUCR        PLVRTCGDKSSKYIEQVIETLMTSK-DYAVQRGAAYGLAGLILGRGVKSLKEHRI
A0A165FVX9_9PEZI    PLIRASTSDIPNYIQQMLDDLFHSK-KYATRRGAAYGLAGIVRGRGISALREFRI
A0A178DN93_9PLEO    PLIRTSEQELPKYIDQMMEQLLQSK-KYASRRGAAYGLAGIVRGKGLGVLKEYRI
A0A177D7J2_ALTAL    PLVRTSEQEVSNYINQMMDQLLTSK-KYASRRGAAYGLAGIVRGKGLGLLKEHRI
A0A0C4DS68_MAGP6    PLVRSCGNKASKYFEEIMETLLTSK-KYAVQRGAAYGLAGLVLGRGIIALREHRI
A0A151N4J5_ALLMI    PLVPAIKEDASGMIQKLMQLLLESD-KYAERRGAAYGLAGLVKGLGILSLKQQEM
A0A146NKU7_FUNHE    PLVPAIKEDAAGIVQNLLQLLLESD-KYAERKGAAYGLAGLVKGLGILALKQQDI
A0A0F7Z917_CROAD    PLVPAIKDDAGGMIQKLLQLLLESD-KYAERKGAAYGLAGLVKGLGILSLKQQEM
A0A0W0DC37_CANGB    ALVFQFKENCGSYITQLLAKLLDSTIAKPVRKGAAWGIAGIVKGYGISALSEFDI
A0A0L8RIK7_SACEU    PLVFQFKPRVGEYLNLLMDKLLNPTVAASIRKGAAWGIAGLVKGYGISALSEFDI
A0A178FRD2_TRIVO    PLIRLDGSDASHFVQELLDQLFTSK-KYAARRGAAYGLAGIVRGKGVLALRDFGI
A0A175WD14_9PEZI    PLVRTCGDKSSKYFDEVLETLMTSK-KYPEQRGAAYGLAGLVLGRGINVLREYRI
A0A194VFU1_9PEZI    PLVQTCSDKSSKYFDEVLETLLTSK-KYAEQRGAAYGLAGLVLGRGINSLRNYRI
A0A151TXV6_CAJCA    PLVQSKKDDAAALVNRLLDQLMKSE-KYGERRGAAFGLAGLVKGFGIPCLKKYRI
A0A0M9ABE9_9HYME    HLVPSIKEDAPKIVDKLMDQLLKSD-KYGERKGAAYGLAGIIKGMGILALKQLDI
A0A072U5F0_MEDTR    PLMQSKQDEADTLVTRLLDQMMKSE-KYGERRGAAFGLAGVVKGFGLSCLKKYKI
A0A0K8V1F4_BACLA    HLVSSVKEKAPEIIKKLLQQLIKSD-KYGERRGAAYGIAGIVKGLGILSLKQFDI
A0A0A1XE05_BACCU    HLVSSVKDRAPEILKKLLQQLIKSD-KYGERRGAAYGIAGIVKGLGILSLKQFDI
A0A131YTA4_RHIAP    PLIPAIKDEAPVLVQKLLNQLLNSE-QYGERRGAAYGLAGLVRGLGILSLKQLDI
A0A0J6I2R9_COCPO    PLIRLSPIETPVYIDELVDQLLHSK-RYATRRGAAYGLAGIVHGRGISALREFRI
A0A074XMM5_AURPU    PLVQASNKEAGQYVQQMMDTVLTAK-KYAAKRGAAYGLAGIVKGRGVSALREFRI
A0A167FV98_9ASCO    PLVKLITPQLGSYFEKILEKLFTSA-KFAERRGAAYGLAGLVKGVGISSLADYDI
A0A1A7XZM1_9TELE    PLVPAIKEDAAGIVRNLLQLLLESD-KYAERKGAAYGLAGLVKGLGILALKQQDI
A0A0P7V1U6_9TELE    PLVPAIKEDAGDMVRKLLQLLLESD-KYAERKGAAYGLAGLVKGLGILALKQQDI
A0A1A7WWE1_9TELE    PLVPAIKEDAAGIVRNLLQLLLESD-KYAERKGAAYGLAGLVKGLGILALKQQDI
                     :                  :         ::*:*:*:**   * *:  . .  :
```

```
GCN1_YEAST            IRNLIEAAEDKKEPKRRESVGFCFQYLSESLGKFFEPYVIEILPNILKNLGDAVP 1760
GCN1_SCHPO            LDSLSELISNRQNATHRQVALFAVEAFSRILGIYFEPYLPDLLPLLLTSFGDNAN
GCN1_DICDI            LDTLQSYIEDKKHPTSRQGALFAFECLCNTIGRVFEPYIIHILPKLLVCFGDNVS
GCN1_MOUSE            MAALTDAIQDKKNFRRREGALFAFEMLCTMLGKLFEPYVVHVLPHLLLCFGDGNQ
GCN1_HUMAN            MAALTDAIQDKKNFRRREGALFAFEMLCTMLGKLFEPYVVHVLPHLLLCFGDGNQ
M7ANV9_CHEMY          MTTLTDAIQDKKNFRRREGALFAFEMLCTMLGKLFEPYVVHVLPHLLLCFGDGNQ
H8X4E7_CANO9          IRTLTDASDDKKDAVKRESVSLVFESLSKSLGKFFEPFVFEILPIILKNLGDMSS
W6A2P0_ICTPU          MTTLTDAIQDKKNARRREGALFAFEMLCNMLGKLFEPYVVHVLPHLLLCFGDGNQ
B6JZI6_SCHJY          MDTLKEALEDKKNKDRRQGALFAIESFSHILGVFFEPYVPEIIPLLISTFGDSST
R4WJK2_RIPPE          MSTLTNAIQDKKNYKHREGALFAFEMLCSVLGRLFEPYIVHVLPHLLLCFGDGNS
W8CCK9_CERCA          MSKLTIFIQEKKNYKSREGALFAFEVLCTTLGRLFEPYIVHVLPHLLQCFGDSSQ
B0W357_CULQU          MTKLTKHIQDKKNFKCREGALFAFEMLCSTLGRLFEPYIVHVLPHLLQCFGDSSS
E1ZX97_CAMFO          MSKLTNAIQDKKNYRHREGALFAFEMLCTMLGRLFEPYIVHVLPHLLLCFGDSSQ
W5JJ28_ANODA          MSKLTAHIQDKKNFKCREGALFAFEMLCSTLGRLFEPYIVHVLPHLLQCFGDSSS
F1KPR4_ASCSU          IKMIQNSLANKKDPKHREGALLALEMLCSTMGKLFEPYIVQVLPSLLICFGDSDE
V9K7F0_CALMI          MGTLTEAIQDKKNFRRREGALFAFEMLCNMLGKLFEPYVVHVLPHLLLCFGDGNQ
W0T7Q3_KLUMA          IRYLIEGSEDKKDPRRRESVAYGFECLSISLGKFFEPYVIEVLPNILKNLGDSVP
S9R951_SCHOY          MDQLHESMTNRQSSSIRQGALFCVEAFSRTLGIYFEPYLPDLLPLLLSSFGDSAS
S9W134_SCHCR          MAQLCESMSNRQSSSIRQGALFCVEAFSRTLGIYFEPYLPDLLPLLLSSFGDSAT
H0GUK0_SACCK          IRNLIEAAEDKKEPKRRESVGFSFQYLSQSLEKFFEPYVIEILPNILKNLGDAVP
E5R2E4_ARTGP          MSRLTEASENKKESNQRQGAVLAYELLAFVLGRVFEPYVIRIVPQLLTSFGDPSI
J8Q888_SACAR          IRNLIEAAEDKKEPKRRESVGFCFQYLSQSLEKFFEPYVIEILPNILKNLGDAVP
C5G992_AJEDR          MAHLTDALENKKDPNQRQGAIMAFELFSLILGAIFEPYVIQIVPQLLGSFGDPSI
G4MR95_MAGO7          MSNLHSALENKKEANQRESALLAYELLATILGRLFEPYVILIVPQLLAGFGDSNA
C9SQ16_VERA1          MTTLKGAIENKKEASQREAGLMALELLSSLLGRIFEPYVIQIVPELLTGFGDSNA
B2WNI9_PYRTR          MSTLKGASDNKKDPNQRQGVYLAYELLSLILGRLFEPYVIQLVPQLLAGFGDSST
S3CN37_OPHP1          LSTLTGAMENKKEANQRESTLLAYELMSTILGRLFEPYVIQIVPQLLAGFGDSNP
G3ALT0_SPAPN          IRELTDAADDKKNPTRREGVSIAFECLSRSLGKFFEPYVIEVLPIILKSLGDSVP
Q7S5P0_NEUCR          LITLRSALENKKEVNQRESAMIALELLSTILGRLFEPYVIQIVPQLLAGFGDSNP
A0A165FVX9_9PEZI      MATLKSATENKKDPNYRQGALFAYELLSLVLGRMFEPYTIQIVPNLLSGFGDSTA
A0A178DN93_9PLEO      MSTLKGASENKKDVNQRQGVYLAYELLSLILGRLFEPYLIQLVPQLLAGFGDTSA
A0A177D7J2_ALTAL      MSTLKGASENKKDPNQRQGVYLAYELFSLILGRLFEPYVIQLVPQLLVGFGDTST
A0A0C4DS68_MAGP6      MSTLNGALENKKDASQRESALLAYELLATMLGRLFEPYVIQIVPQLLTGFGDSNA
A0A151N4J5_ALLMI      MTTLTDAIQDKKNFRRREGALFAFEMLCSMLGKLFEPYVVHVLPHLLLCFGDGNQ
A0A146NKU7_FUNHE      MTTLTDAIQDKKNFRRREGALFAFEMLCNMLGKLFEPYVVHVLPHLLLCFGDGNQ
A0A0F7Z917_CROAD      MAKLTDAIQDKKNFRRREGALFAFEMLCIMLGKLFEPYVVHVLPHLLLCFGDGNQ
A0A0W0DC37_CANGB      IRSLMEASEDKKDAIKRESVAYAFEYLSQSLGKLFEPYVIEVLPNILKNLGDSVP
A0A0L8RIK7_SACEU      IRNLIEAAEDKKEPKRRESVGFCFQYLSQSLGKFFEPYVIEILPNILKNLGDAVP
A0A178FRD2_TRIVO      MSRLAEASENKKESNQRQGAVLAYELLAFVLGRVFEPYVIRIVPQLLTSFGDPSI
A0A175WD14_9PEZI      MITLNSCLENKKEIRQRESAMLAYELLSTILGRLFEPYVIQIVPQLLAGFGDGNA
A0A194VFU1_9PEZI      MNTLRGACENKKEVNQREAALLAFELLSTILGRLFEPYVIQIVPQLLTGFGDGNA
A0A151TXV6_CAJCA      VIILQECLAERNSAKSREGALLGFECLCETLGRLFEPYVIQMLPLLLVSFSDQVV
A0A0M9ABE9_9HYME      MTTLTNAIQDKKNYRHREGALFAFEMLCTMLGRLFEPYIVHVLPHLLLCFGDSSQ
A0A072U5F0_MEDTR      VIILQECLAERNSAKSREGALLGFECLCETLGKLFEPYVIQMLPLLLVSFSDQVA
A0A0K8V1F4_BACLA      MSKLTTFIQEKKNYKSREGALFAFEVLCTTLGRLFEPYIVHVLPHLLQCFGDSSQ
A0A0A1XE05_BACCU      MSKLTTYIQEKKNYKSREGALFAFEVLCTTLGRLFEPYIVHVLPHLLQCFGDSSQ
A0A131YTA4_RHIAP      MNTLTEAVQDKKNARRKEGALLAFEMLCSVLGRLFEPYVVHVLPHLLLCFGDSNQ
A0A0J6I2R9_COCPO      MPRLKEASENKKDPNERQGSLLAFELLSLVLGRMFEPYIIQILPQLLTAFGDPSV
A0A074XMM5_AURPU      MSNLVSAIEDKKDPYKRQGAFLAYELMSLILGRVFEPYVIQVVPQLLSGFGDAIV
A0A167FV98_9ASCO      IRSLTDAIEDKKDPKKRQGGQFAFECLSMALGKYFEPYVIELVPIILSCLGDSNI
A0A1A7XZM1_9TELE      MTTLTEAIQDKKNFRRREGSLFAFEMLCNMLGKLFEPYVVHVLPHLLLCFGDGNQ
A0A0P7V1U6_9TELE      MTTLTDAIQDKKNFRRREGALFAFEMLCNMLGKLFEPYVVHVLPHLLLCFGDGNQ
A0A1A7WWE1_9TELE      MTTLTEAIQDKKNFRRREGSLFAFEMLCNMLGKLFEPYVVHVLPHLLLCFGDGNQ
                      :  :     :::    ::      : :.  :   ***:   ::* ::  :.*
```

```
GCN1_YEAST          EVRDATARATKAIMAHTTGYGVKKLIPVAVSNLDEIAWRTKRGSVQLLGNMAYLD 1815
GCN1_SCHPO          EVREATMDAVKQIMSQLSAFGVKLLLPTLLDGLNEYNWRSKKASVEILGLMSYMA
GCN1_DICDI          EVRDATADTAKAIMSQLSGHGVKIVLPALLKALDDRSWRTKEGSIELLGAMAFCA
GCN1_MOUSE          YVREAADDCAKAVMSNLSAHGVKLVLPSLLAALEEESWRTKAGSVELLGAMAYCA
GCN1_HUMAN          YVREAADDCAKAVMSNLSAHGVKLVLPSLLAALEEESWRTKAGSVELLGAMAYCA
M7ANV9_CHEMY        YVREAADDCAKAVMSNLSAHGVKLVLPSLLAALEEESWRTKAGSVELLGAMAYCA
H8X4E7_CANO9        EVRVATDNAAKEIMKNTTSFGVKKLIPLAISNLDEIAWRSKKGSVELLGAMAYLD
W6A2P0_ICTPU        YVREAADDCAKAVMRNLSAHGVKLVLPSLLVALEEESWRTKAGSVELLGAMAFCA
B6JZI6_SCHJY        EVRDATSDAAKAIMSHLSGYGVKLILPSLLDGLNEYNWRSKFASVEMLGLMSYMA
R4WJK2_RIPPE        YVRDATDDCAKVVMSKLSAHGVKLVLPSLLAALEKDSWRTKTGSVELLGAMAYCA
W8CCK9_CERCA        YVRQAADDTAKVVMGKLSAHGVKLVLPSLLNALDEDSWRTKMASVELLGAMAFCA
B0W357_CULQU        YVREAADECAKTVMAKLSAHGVKLVLPSLLNALDEDSWRTKTASVELLGAMAFCA
E1ZX97_CAMFO        YVRTATDDTARVVMSKLSAHGVKLVLPSLLAALEEDSWRTKTGSVELLGAMAYCA
W5JJ28_ANODA        YVRQAADECAKTVMAKLSAHGVKLVLPSLLNALDEDSWRTKTASVELLGAMAFCA
F1KPR4_ASCSU        NVRRAADDAARAMMAMLSAHGVKLVLPSLLAALDEESWRTKCASVELLGAMAFCA
V9K7F0_CALMI        YVREASDDTAKAVMRNLSAHGVKLVLPSLLVALEEESWRTKAGSVELLGAMAYCA
W0T7Q3_KLUMA        EVREATAQAAKAIMSSTTSFGVKKLIPVAVSNLDDISWRTKRGSVELLGNMAYLD
S9R951_SCHOY        EVREATLEAVKHIMSQLSAYGVKLLLPSLLDGLNDYNWRSKRASVEILGLMSYMA
S9W134_SCHCR        EVREATLESVKHIMSQLSAYGVKLLLPSLLDGLNDYNWRSKRASVEILGLMSFMA
H0GUK0_SACCK        EVREATAHATKAIMAHTTGYGVKKLIPVAVSNLDEIAWRTKRGSVQLLGNMAYLD
E5R2E4_ARTGP        DVRDACLDAAKACFASLSSFGVKQILPTLLDGLDDTQWRSKKGACDLLGAMAYLD
J8Q888_SACAR        EVRDATARATKAIMTHTTGYGVKKLIPVAVSNLDEIAWRTKRGSVQLLGNMAYLD
C5G992_AJEDR        DVRNACLDAAKTCFSNLSSYGVKQILPTLLDGLDDQQWRSKKGACDLLGAMAYLD
G4MR95_MAGO7        NVRDAALASAKACFARLSSYGVKQILPTLLRGLDDDQWRSKKGACDLLGAMAYLD
C9SQ16_VERA1        DVRDACLAAAKACFAKLSSYGVKQILPTLLRGLDDQQWRSKKGACDLLGAMAYLD
B2WNI9_PYRTR        DVREACLDAAKTCFSTLSSFGVKQVLPILLEGLDEDQWRSKKGACDSLGAMAYLD
S3CN37_OPHP1        NVRDAALAAAKACFAKLSSFGVKQILPTLLDGLDDTQWRSKKGACDLLGAMAYLD
G3ALT0_SPAPN        EVRDSTDRAAKQIMKNTTSFGVKKLIPLAISNLDEIAWRSKKGSVELLGSMAYLD
Q7S5P0_NEUCR        NVREAALHAAKSCFAKLSSFGVKKILPTLLDGLDEDQWRSKKGACDLLGAMAYLD
A0A165FVX9_9PEZI    DVREACLDAAKTCFASLSSYGVKQILPTLLDGLDDQQWRSKKGACDLLGAMAYLD
A0A178DN93_9PLEO    DVREACLDAAKTCFSTLSSFGVKQVLPILLEGLDDQQWRSKKGACDSLGAMAYLD
A0A177D7J2_ALTAL    DVREACLDAAKTCFSSLSSFGVRQVLPILLEGLDEDQWRSKKGACDSLGAMAYLD
A0A0C4DS68_MAGP6    NVRDAALASAKACFARLSSYGVKNILPTLLNGLDDDQWRSKKGACDLLGAMAYLD
A0A151N4J5_ALLMI    YVREAADDCAKAVMSNLSAHGVKLVLPSLLAALEEESWRTKAGSVELLGAMAYCA
A0A146NKU7_FUNHE    YVREAADDCAKAVMRNLSAHGVKLVLPSLLVALEEESWRTKAGSVELLGAMAYCA
A0A0F7Z917_CROAD    YVREAADDCAKAVMSNLSAHGVKLVLPSLLAALEEDSWRTKAGSVELLGAMAYCA
A0A0W0DC37_CANGB    EVRDATAAATKSIMANTTGYGVKKMIPVTVANLDEIAWRTKRGSVQLLGNMAYLD
A0A0L8RIK7_SACEU    EVRDATARATKAIMAHTTGYGVKKLIPVAVSNLDEIAWRTKRGSVQLLGNMAYLD
A0A178FRD2_TRIVO    DVRDACLDAAKACFASLSSFGVKQILPTLLDGLDDTQWRSKKGACDLLGAMAYLD
A0A175WD14_9PEZI    DVREAALAAAKACFAKLSSYGVKQILPTLLNGLDDDQWRSKKGACDLLGAMAYLD
A0A194VFU1_9PEZI    NVREASLAAAKACFAKLSSYGVKKILPTLLDGLDEDQWRSKKGACDLLGAMAYLD
A0A151TXV6_CAJCA    AVREAAECAARAMMSQLSAQGVKLVLPSLLKGLEDKAWRTKQSSVQLLGAMAYCA
A0A0M9ABE9_9HYME    YVRTATDDTARVVMSKLSAHGVKLVLPSLLAALEEDSWRTKTGSVELLGAMAYCA
A0A072U5F0_MEDTR    AVREAAECAARAMMSQLSAQGVKLVLPSLLKGLEDKAWRTKQSSVQLLGAMAYCA
A0A0K8V1F4_BACLA    YVRQAADDTAKVVMGKLSAHGVKLVLPSLLNALDEDSWRTKTASVELLGAMAFCA
A0A0A1XE05_BACCU    YVRQAADDTAKVVMGKLSAHGVKLVLPSLLNALDEDSWRTKTASVELLGAMAFCA
A0A131YTA4_RHIAP    YVREATDNTAKAVMSKLTAHGVKLTLPSLLAGLENDLWRTKSGSVELLGAMAYCA
A0A0J6I2R9_COCPO    DVRDACLDTAKACFASLSSYGVKQILPTLLEGLDDPQWRSKKGACDLLGAMAYLD
A0A074XMM5_AURPU    DVREACLDAAKTCFASLSSYGVKQVLPTLLEGLDEQQWRSKKGACDSLGAMAYLD
A0A167FV98_9ASCO    EVREATTYASRSIMKNTTGYGISKLIPLALENLDQTAWRAKKGAVELLGNMAYLD
A0A1A7XZM1_9TELE    YVREAADDCAKAVMRNLSAHGVKLVLPSLLVALEEESWRTKAGSVELLGAMAFCA
A0A0P7V1U6_9TELE    YVREAADDCAKAVMRNLSAHGVKLVLPSLLVALEEESWRTKAGSVELLGAMAYCA
A0A1A7WWE1_9TELE    YVREAADDCAKAVMRNLSAHGVKLVLPSLLVALEEESWRTKAGSVELLGAMAFCA
                     ** :     :  :   :. *:   :*  :  *:.  **:* .: : ** *::
```

```
GCN1_YEAST           PTQLSASLSTIVPEIVGVLNDSHKEVRKAADESLKRFGEVIRNPEIQKLVPVLLQ 1870
GCN1_SCHPO           PKQLSVFLPTIIPKLSEVLTDSHSQVRNTANKSLLRFGDVISNPEIQTLVPTLLK
GCN1_DICDI           PKQLSSCLPTIVPKLTYVLNDTHTKVQEAAKEALSHIGSVIRNPEIQIHVPLLLQ
GCN1_MOUSE           PKQLSSCLPNIVPKLTEVLTDSHVKVQKAGQQALRQIGSVIRNPEILAIAPVLLD
GCN1_HUMAN           PKQLSSCLPNIVPKLTEVLTDSHVKVQKAGQQALRQIGSVIRNPEILAIAPVLLD
M7ANV9_CHEMY         PKQLSSCLPNIVPKLTEVLTDSHVKVQKAGQQALRQIGSVIRNPEILAIAPVLLD
H8X4E7_CANO9         PEQLSASLSIIIPEIVGVLNDTHKEVRKAAEQSLKRFGEVIRNPEIQQIVPYLIN
W6A2P0_ICTPU         PKQLSSCLPSIVPKLTEVLTDSHVKVQKAGQQALRQIGSVIRNPEIQAITPILLD
B6JZI6_SCHJY         PKQLSYSLPTIIPRLTDVLTDSHNQVRNAANKSLTRFGDVISNPEIQTLVPVLLK
R4WJK2_RIPPE         PKQLSSCLPSIVPKLIEVLSDSHVKVQKAGTEALKQIGSVIRNPEIQSIVPVLLE
W8CCK9_CERCA         PKQLSSCLPNIVPKLIEVLGDSHTKVQEAGAEALKVIGSVIKNPEIQAIVPILLT
B0W357_CULQU         PKQLSSCLPSIVPKLMEVLGDSHIKVQEAGADALKVIGSVIKNPEIQAIVPVLLK
E1ZX97_CAMFO         PKQLSSCLPSIVPKLIEVLSDSHTKVQEAGAEALKVIGSVIRNPEIQAIVPVLLK
W5JJ28_ANODA         PKQLSSCLPSIVPKLMEVLGDSHIKVQEAGADALRVIGSVIKNPEIQAIVPVLLK
F1KPR4_ASCSU         PKQLSACLPSIVPKLIEVLADSHSKVQKSGEKALKQIAKVIRNPEILGISSHLLA
V9K7F0_CALMI         PKQLSSCLPNIVPKLTEVLTDSHVKVQKAGQQALRQIGSVIRNPEILAITPVLLD
W0T7Q3_KLUMA         PTQLSASLSTIVPEIVGVLNDSHKEVRKAADESLNRFGEVIRNPEIQKLVPILIN
S9R951_SCHOY         PKQLSVSLPTIVPKLTEVLTDSHSQVRNTANNSLLRFGEVISNPEIQNLVPVLLK
S9W134_SCHCR         PKQLSVSLPTIVPKLTEVLTDSHSQVRNTANKSLLRFGEVISNPEIQNLVPILLK
H0GUK0_SACCK         PTQLSASLSTIVPEIVGVLNDSHKEVRKAADESLKRFGEVIRNPEIQKLVPILLQ
E5R2E4_ARTGP         PQQLALNLPDIIPPLTEVLNDSHKEVRNSANRSLQRFGDVISNPEVKSLVSILLK
J8Q888_SACAR         PTQLSASLSTIVPQIVGVLNDSHKEVRKAADESLKRFGEVIRNPEIQKLVPTLLQ
C5G992_AJEDR         PQQLAISLPDIIPPLTIVLNDSHKEVRNSANRSLQRFGEVISNPEVKSLVGVLLK
G4MR95_MAGO7         PQQLALSLPEIIPPLTAVLNDSHKEVRSGANKSLKRFGEVISNPEVKGLVDILLK
C9SQ16_VERA1         PQQLALSLPDIIPPLTAVLNDSHKEVRSGANKSLKRFGEVISNPEVKGLVDILLK
B2WNI9_PYRTR         PNQLALSLPDIIPPLTVVLTDSHKEVRASANRSLQRFGEVISNPEIKSVVNIILK
S3CN37_OPHP1         PAQLALSLPEIIPPLTAVLNDSHKEVRSAANKSLKRFGEVINNPEVRSLVDILLK
G3ALT0_SPAPN         PTQLSASLSTIVPEIVGVLNDTHKEVRKAAEQSLKRFGEVIRNPEIQAIVPDLIN
Q7S5P0_NEUCR         PQQLAQSLPEIIPPLTAVLNDSHKEVRLAANKSLKRFGEVIENPEIHSLVDILLK
A0A165FVX9_9PEZI     PQQLAQSLPDIIPPLTDVLNDSHKEVRASANRSLQRFGEVITSPEIKGVVNILLK
A0A178DN93_9PLEO     PNQLALSLPEIIPPLTTVLTDSHKEVRSSANRSLQRFGEVISNPEVKSVVNILLK
A0A177D7J2_ALTAL     PSQLALSLPEIIPPLTVVLTDSHKEVRASANRSLQRFGEVISNPEIKSVVNIILK
A0A0C4DS68_MAGP6     PQQLALSLPEIIPPLTAVLNDSHKEVRSAANKSLKRFGEVINNPEVKGLVDILLK
A0A151N4J5_ALLMI     PKQLSSCLPNIVPKLTEVLTDSHVKVQKAGQQALRQIGSVIRNPEILAIAPVLLD
A0A146NKU7_FUNHE     PKQLSSCLPSIVPKLTEVLTDSHVKVQKAGQQALRQIGSVIRNPEILAITPILLD
A0A0F7Z917_CROAD     PKQLSSCLPNIVPKLTEVLTDSHVKVQKAGQQALRQIGSVIRNPEILAIAPVLLD
A0A0W0DC37_CANGB     PTQLSNSLSTIVPQIVGVLNDSHKEVRKAADESLKRFGEVIRNPEIQRLVPILLK
A0A0L8RIK7_SACEU     PTQLSASLSTIVPEIVGVLNDSHKEVRKAADESLKRFGEVIRNPEIQKLVPILLQ
A0A178FRD2_TRIVO     PQQLALNLPDIIPPLTEVLNDSHKEVRNSANRSLQRFGDVISNPEVKSLVSILLK
A0A175WD14_9PEZI     PQQLAQSLPEIIPPLTAVLNDSHKEVRLAANKSLKRFGEVINNPEIHSLVDILLK
A0A194VFU1_9PEZI     PQQLANSLPDIIPPLTAVLNDSHKEVRTAAKQSLKRFGEVINNPEVKSLVDILLK
A0A151TXV6_CAJCA     PQQLSQCLPKIVPKLTEVLTDTHPKVQSAGQMALQQVGSVIKNPEISALVPTLLK
A0A0M9ABE9_9HYME     PKQLSSCLPSIVPKLIEVLSDSHTKVQEAGAEALKVIGSVIRNPEIQAIVPVLLK
A0A072U5F0_MEDTR     PQQLSQCLPKIVPKLTEVLTDSHPKVQSAGQTALQQVGSVIKNPEIAALVPTLLK
A0A0K8V1F4_BACLA     PKQLSSCLPNIVPKLIEVLGDSHTKVQEAGADALKVIGSVIKNPEIQAIVPILLT
A0A0A1XE05_BACCU     PKQLSSCLPNIVPKLIEVLGDSHTKVQEAGADALKVIGSVIKNPEIQAIVPILLT
A0A131YTA4_RHIAP     PKQLSSCLPSIVPKLIEVLSDSHVKVQRAGAQALKQIGSVIKNPEIQAIVPVLLD
A0A0J6I2R9_COCPO     PQQLAVSLPDIIPPLTVVLNDSHKEVRNSANRSLQRFGDVISNPEVKGLVNILLK
A0A074XMM5_AURPU     PEQLAVSLPEIIPPLTDVLNDSHKEVRASANRSLQRFGDVISNPEIKSQVDIILK
A0A167FV98_9ASCO     PQQLSSSLSTIIPEIVGVLNDTHKEVRNAANQSLQRFGEVIRNPEIQTLVPILIK
A0A1A7XZM1_9TELE     PKQLSSCLPSIVPKLTEVLTDSHVKVQKAGQQALRQIGSVIRNPEILAITPILLD
A0A0P7V1U6_9TELE     PKQLSSCLPSIVPKLTEVLTDSHVKVQKAGQQALRQIGSVIRNPEILAITPILLD
A0A1A7WWE1_9TELE     PKQLSSCLPSIVPKLTEVLTDSHVKVQKAGQQALRQIGSVIRNPEILAITPILLD
                     * **:  *. *:* :  ** *:* :*:  .  :*  ...** .**:      ::
```

```
GCN1_YEAST               AIGDPTKYTEEALDSLIQTQFVHYIDGPSLALIIHIIHRGMHDRSANIKRKACKI 1925
GCN1_SCHPO               ALSDCTRYTDDALEALLKTSFVHYLDPPSLALVIPILKYGLRERNAGTKRQSAKI
GCN1_DICDI               TYDDPEIHSKELLENLLSTNYVHTIDPASLSLLLPILERTLKERSSELKKMSCQI
GCN1_MOUSE               ALTDPSRKTQKCLQTLLDTKFVHFIDAPSLALIMPIVQRAFQDRSTDTRKMAAQI
GCN1_HUMAN               ALTDPSRKTQKCLQTLLDTKFVHFIDAPSLALIMPIVQRAFQDRSTDTRKMAAQI
M7ANV9_CHEMY             ALTDPSRKTQECLQTLLDTKFVHFIDAPSLALIMPIVQRAFQDRSTDTRKMAAQI
H8X4E7_CANO9             AIGDPTKHLDEALDKLTKTQFVHYIDSSSLALIIHVIHRAMKDRSASTKKKACQI
W6A2P0_ICTPU             ALTDPSHKTQHCLHTLLETKFVHFIDAPSLALIMPIVQRAFQDRSTDTRKMAAQI
B6JZI6_SCHJY             ALSDPTIHTEEALSALVKTPFVHYIDPPSLALVVPIVYYGLNERVAAVKKQSAKI
R4WJK2_RIPPE             ALQDPANKTATCLQTLLDTQFVHFIDAPSLALIMPVVQRAFMDRSTETRKMAAQI
W8CCK9_CERCA             ALEDPSTNTFICLHSLLQTKFVHFIDAPSLALIMPVVERAFMDRSTETRKMAAQI
B0W357_CULQU             ALENPSNKTSHCLQSLLETKFVHFIDAPSLALIMPVVQRAFMDRSTETRKMAAQI
E1ZX97_CAMFO             ALQDPSHKTATCLQTLLDTQFVHFIDAPSLALIMPVVQRAFLDRSTETRKMAAQI
W5JJ28_ANODA             ALEDPSGKTSACLQSLLETKFVHFIDAPSLALIMPVVQRAFMDRSTETRKMAAQI
F1KPR4_ASCSU             GLVDPASKTTSCLQTIVNTRFIHYIDAASLALIMPIVRRAFSDRNTETRRMAAQI
V9K7F0_CALMI             ALTDPSRRTQLCLQTLLDTKFVHFIDAPSLALIMPIVQRAFQDRSTDTRKMAAQI
W0T7Q3_KLUMA             AIGDPTKHTEEALDALIQTQFVHYIDGPSLALIIHVIHRGMRDRSANTKRKACKI
S9R951_SCHOY             ALSDCTRYTDDALSAILKTAFVHYLDSPSLALVIPIIQYGMRERNASTKRQSAKI
S9W134_SCHCR             ALSDCTRFTDDALSAILKTAFVHYLDSPSLALVIPIIQYGMRERNASTKRQSAKI
H0GUK0_SACCK             AIGDPTRYTEEALDSLIQTQFVHYIDGPSLALIIHIIHRGMHDRSANVKRKACKI
E5R2E4_ARTGP             ALSDPTKYTDEALDALIKISFVHYLDAPSLALVVRILERGLSDRST-TKRKAAQI
J8Q888_SACAR             AIGDPTKYTEEALDSLIQTQFVHYIDGPSLALIIHIIHRGMHDRSANIKRKACKI
C5G992_AJEDR             ALSDPTKYTDEALDALIRVSFIHYLDAPSLALVVRILERGLGDRST-TKKKAAQI
G4MR95_MAGO7             ALSDPTKYTDEALDSLIKVQFVHYLDAPSLALVSRILQRGLADRSN-TKRKASQV
C9SQ16_VERA1             ALSDPTKYTDDALDALIKVQFVHYLDAPSLALVTRILQRGLGERSN-TKRKAAQV
B2WNI9_PYRTR             ALSDPTKYTDDALDALIKIQFAHFLDAPSLALVVRILERGLGDRSG-TKRKSSQI
S3CN37_OPHP1             ALSDPTKYTDDALDALIKIKFVHYLDAPSLALVSRILQRGLGDRSN-TKRKASQV
G3ALT0_SPAPN             AIGDPTKYTDDALDKLIKTQFVHYIDGPSLALIIHVIHRGMKDRSASTKKKACQI
Q7S5P0_NEUCR             ALSDPTKYTDEALDALIKVQFVHYLDAPSLALVSRILQRGLGDRSN-TKRKAAQV
A0A165FVX9_9PEZI         ALSDPTKFTDDALDALIKVSFVHYIDAPSLALVVRILERGLGDRSA-TKRKSSQI
A0A178DN93_9PLEO         ALSDPTKHTDDALDALIKIQFAHFLDAPSLALVSRILERGLGDRSA-TKRKSAQI
A0A177D7J2_ALTAL         ALSDPTKYTDDALDALIKIQFAHFLDAPSLALVVRILERGLGDRSG-TKRKSSQI
A0A0C4DS68_MAGP6         ALSDPTKYTDEALDSLIKVQFVHYLDAPSLALVSRILQRGLGDRSN-TKRKASQV
A0A151N4J5_ALLMI         ALTDPSRKTQKCLQTLLDTKFVHFIDAPSLALIMPIVQRAFQDRSTDTRKMAAQI
A0A146NKU7_FUNHE         ALTDPSRKTQTCLQTLLDTKFVHFIDAPSLALIMPIVQRAFQDRSTDTRKMAAQI
A0A0F7Z917_CROAD         ALTDPSRKTQKCLQTLLDTKFVHFIDAPSLALIMPIVQRAFQDRSTDTRKMAAQI
A0A0W0DC37_CANGB         AIGDPTKYTEEALDALIQTQFVHYIDGPSLALIIHIIHRGMHDRSANTKRKACKI
A0A0L8RIK7_SACEU         AIGDPTKYTEEALDSLIQTQFVHYIDGPSLALIIHIIHRGMHDRSANIKRKACKI
A0A178FRD2_TRIVO         ALSDPTKYTDEALDALIKISFVHYLDAPSLALVVRILERGLSDRST-TKRKAAQI
A0A175WD14_9PEZI         ALSDPTKYTDEALESLIKIQFVHYLDAPSLALVSRILQRGLGDRSS-TKRKAAQV
A0A194VFU1_9PEZI         ALSDPTKYTDEALDSLIKVQFVHYLDAPSLALVSRILQRGLGDRSN-TKRKASQV
A0A151TXV6_CAJCA         GLSDPNEHTKYSLDILLQTTFVNSIDAPSLALLVPIVHRGLRERSADTKKRAAQI
A0A0M9ABE9_9HYME         ALQDPSHKTATCLQTLLDTQFVHFIDAPSLALIMPVVQRAFLDRSTETRKMAAQI
A0A072U5F0_MEDTR         GLSDPNEHTKYSLDILLQTTFVNSIDAPSLALLVPIVHRGLRVRSADTKKRASQI
A0A0K8V1F4_BACLA         ALEDPSKNTSNCLHSLLQTKFVHFIDAPSLALIMPVVERAFMDRSTETRKMAAQI
A0A0A1XE05_BACCU         ALEDPSKNTSNCLHSLLQTKFVHFIDAPSLALIMPVVERAFMDRSTETRKMAAQI
A0A131YTA4_RHIAP         ALQDPAEKTSGCLATLLNTKFVHFIDAPSLALIMPVVQRAFQDRSTETKKMAAQI
A0A0J6I2R9_COCPO         ALSDPTKYTDEALDALIKISFIHYLDAPSLALIVRILERGLGDRST-TKRKAAQI
A0A074XMM5_AURPU         ALSDPTKYTDDALDALIKVNFIHYLDAPSLALVVRVLERGLGDRSA-TKKKASQI
A0A167FV98_9ASCO         AISDSTRYTEDALDSLLKTKFVHYIDAPSLSLIVHVLHRGLKDRSAAIKRKACQI
A0A1A7XZM1_9TELE         ALTDPSRRTQTCLQTLLDTKFVHFIDAPSLALIMPIVQRAFQDRSTDTRKMAAQI
A0A0P7V1U6_9TELE         ALTDPSRKTQHCLQTLLDTKFVHFIDAPSLALIMPIVQRAFLDRSTDTRKMAAQI
A0A1A7WWE1_9TELE         ALTDPSRRTQTCLQTLLDTKFVHFIDAPSLALIMPIVQRAFQDRSTDTRKMAAQI
                            :        *  :    : : :* .**:*:  ::   :  *    :: :.::
```

```
GCN1_YEAST          VGNMAIL-VDTKDLIPYLQQLIDEVEIAMVDPVPNTRATAARALGALVE-RLGEE 1980
GCN1_SCHPO          FGLMASL-TEPENLAVYLESLMPRLREVLIDPVPDTRATAAKALGSLIE-KLGEK
GCN1_DICDI          VGNLCSL-TEPKDLVPYLNILMPVMKTVLLDPIPEVRAICARALGLLVR-GMGEE
GCN1_MOUSE          IGNMYSL-TDQKDLAPYLPSVTPGLKASLLDPVPEVRTVSAKALGAMVK-GMGES
GCN1_HUMAN          IGNMYSL-TDQKDLAPYLPSVTPGLKASLLDPVPEVRTVSAKALGAMVK-GMGES
M7ANV9_CHEMY        IGNMYSL-TDQKDLSPYLPSVTPGLKTSLLDPVPEVRTVSAKALGAMVK-GMGES
H8X4E7_CANO9        VGNMAIL-VDSKDLQPYLNELVEELETAMVDPVPATRSTAARALGSLVE-KLGEE
W6A2P0_ICTPU        IGNMYSL-TDQKDLSPYLPSVIPGLKTSLLDPVPEVRTVSAKALGAMVK-GMGES
B6JZI6_SCHJY        FGLMASL-TDPSDLSVHLEKLVPRLREVLIDPVPDTRATAAKALGSLVE-KLGET
R4WJK2_RIPPE        IGNMYSL-TDQKDLTPYLPTIIPGLKTSLLDPVPEVRSVSARALGAMVR-GMGEA
W8CCK9_CERCA        IGNMYSL-TDQKDLVPYLPNIIPGLKMSLLDPVPEVRAISARALGAMVR-GIGES
B0W357_CULQU        IGNMYSL-TDQKDLTPYLPNIIPGLKTSLLDPVPEVRAVSSRALGAMVK-GMGES
E1ZX97_CAMFO        IGNMYSL-TDQKDLTPYLPTIIPGLKTSLLDPVPEVRSVSARALGAMVR-GMGES
W5JJ28_ANODA        IGNMYSL-TDQKDLTPYLPNIIPGLKTSLLDPVPEVRAVSARALGAMVR-GMGES
F1KPR4_ASCSU        IASIYSL-TDNKDMEPYLCELVPGLQKSLLDPVPEIRTVAAKAFGAIVACSSGDT
V9K7F0_CALMI        IGNMYSL-TDQKDLAPYLPSVMPGLKLSLLDPVPEVRTVSAKALGAMVK-GMGES
W0T7Q3_KLUMA        VGNMAIL-VDTKDLVPYLQQLIDEVEIAMVDPVPNTRATAARALGALVE-RLGEE
S9R951_SCHOY        FGLMASL-TEPENLAIYLDPLMPRLREVLIDPVPDTRATAAKALGSLME-KLGET
S9W134_SCHCR        FGLMASL-TEPENLAIYLDALMPKLREVLIDPVPDTRATAAKALGSLME-KLGET
H0GUK0_SACCK        VGNMAIL-VDTKDLVPYLQQLIDEVEIAMVDPVPNTRATAARALGALVE-RLGEE
E5R2E4_ARTGP        IGSLAHL-TERKDLISHLPILVAGLKTAVVDPVPTTRATASKALGSLIE-KLGED
J8Q888_SACAR        VGNMAIL-VDTKDLVPYLQQLIDEVEIAMVDPVPNTRATAARALGALVE-RLGED
C5G992_AJEDR        IGSLAHL-TERKDLISHLPILVAGLKLAIVDPVPTTRATASKALGSLIE-KLGED
G4MR95_MAGO7        IGSLAHL-TERKDLVSHLPVLVAGLKIAVVDPVPTTRATASRALGSLME-KLGEE
C9SQ16_VERA1        IGSLAHL-TERKDLISHLPILVAGLKLAIVDPVPTTRATASRALGSLME-KLGEE
B2WNI9_PYRTR        IGSLAYL-SERKDLTSHLPILVAGLRVAIVDPVPATRATASKALGSLVE-KLGED
S3CN37_OPHP1        IGSLAHL-TEKKDLVAHLPVLVAGLKVAVVDPVPTTRATASRALGSLVE-KLGED
G3ALT0_SPAPN        VGNMAIL-VDAKDLRPYLNELVGELEIAMVDPVPATRSTAARALGSLVE-KLGEE
Q7S5P0_NEUCR        IGSLAHL-TERKDLVAHLPVLVAGLKIAIVDPVPTTRATASRALGSLVE-KLGED
A0A165FVX9_9PEZI    IGSLAHL-TERKDLVAHLPILVAGLKIAVIDPVPTTRATASKAFGSLME-KLGED
A0A178DN93_9PLEO    IGSLAYL-TERKDLVTHLPVLVAGLRVAIVDPVPTTRATASKALGSLME-KLGED
A0A177D7J2_ALTAL    IGSLAYL-SERKDLTSHLPILVAGLRVAIVDPVPATRATASKALGSLVE-KLGED
A0A0C4DS68_MAGP6    IGSLAHL-TERKDLVSHLPVLVAGLKVAVVDPVPTTRATASRALGSLVE-KLGEE
A0A151N4J5_ALLMI    IGNMYSL-TDQKDLAPYLPSVTPGLKASLLDPVPEVRTVSAKALGAMVK-GMGES
A0A146NKU7_FUNHE    IGNMYSL-TDQKDLSPYLPSVIPGLKASLLDPVPDVRTVSAKALGAMVK-GMGES
A0A0F7Z917_CROAD    IGNMYSL-TDQKDLAPYLPSVTPGLKTSLLDPVPEVRTVSAKALGAMVK-GMGES
A0A0W0DC37_CANGB    VGNMAIL-VETKDLIPYLQQLIDEVEIAMVDPVPNTRATAARALGALVE-RLGED
A0A0L8RIK7_SACEU    VGNMAIL-VDTKDLVPYLQQLIDEVEIAMVDPVPNTRATAARALGALVE-RLGEE
A0A178FRD2_TRIVO    IGSLAHL-TERKDLISHLPILVAGLKTAVVDPVPTTRATASKALGSLIE-KLGED
A0A175WD14_9PEZI    IGSLAHL-TERKDLVAHLPVLVAGLKVAVVDPVPTTRATASRALGSLVE-KLGED
A0A194VFU1_9PEZI    IGSLAHL-TEKKDLVAHLPVLVAGLKAAVVDPVPTTRATASRALGSLVE-KLGED
A0A151TXV6_CAJCA    VGNMCSLVTEPKDMIPYIGLLLPEVKKVLVDPIPEVRSVAARAIGSLIG-GMGEE
A0A0M9ABE9_9HYME    IGNMYSL-TDQKDLTPYLPTIIPGLKTSLLDPVPEVRSVSARALGAMVR-GMGES
A0A072U5F0_MEDTR    VGNMCSLVTEPKDMIPYIGLLLPEVKKVLVDPIPEVRSVAARAIGSLIG-GMGED
A0A0K8V1F4_BACLA    IGNMYSL-TDQKDLVPYLPNIIPGLKMSLLDPVPEVRAISARALGAMVR-GIGES
A0A0A1XE05_BACCU    IGNMYSL-TDQKDLVPYLPNIIPGLKMSLLDPVPEVRAISARALGAMVR-GIGES
A0A131YTA4_RHIAP    IGNMYSL-TDHKDLAPYLPAIIPGLKQALLDPVPEVRSVSSRALGAMIK-GMGET
A0A0J6I2R9_COCPO    IGSLAHL-TERKDLTSHLPILVAGLKIAIVDPVPTTRATASKALGSLIE-KLGEE
A0A074XMM5_AURPU    IGSLAHL-TERKDLITHLPILVAGLRVAIVDPVPTTRATASKALGSTIE-KLGED
A0A167FV98_9ASCO    VGNMSIL-TDSRDLQPYLPSMVSELEVAMVDPVPETRATASKALGSLVE-KLGEE
A0A1A7XZM1_9TELE    IGNMYSL-TDQKDLSPYLPSVIPGLKASLLDPVPEVRTVSAKALGAMVK-GMGES
A0A0P7V1U6_9TELE    IGNMYSL-TDQKDLSPYLPSVIPGLKASLLDPVPEVRTVSAKALGAMVK-GMGES
A0A1A7WWE1_9TELE    IGNMYSL-TDQKDLSPYLPSVIPGLKASLLDPVPEVRTVSAKALGAMVK-GMGES
                    .. :  *  :  ::  ::  :   :.  ::**:*  *: .::*:*  :    *:
```

```
GCN1_YEAST              QF---PDLIPRLLDTLSDESKSGDRLGSAQALAEVISGLGLTKLDEMLPTILAGV 2035
GCN1_SCHPO              KF---PTLIPELFNVLRSECSEVDRQGAAQGLSEILAGLGLARLEDVLPEILKNT
GCN1_DICDI              NF---STLIPWLLETVKSDQGAVERSGAAQGLSEVLASLDISRFNSLINELLAMT
GCN1_MOUSE              CF---EDLLPWLMETLTYEQSSVDRSGAAQGLAEVMAGLGVEKLEKLMPEIVATA
GCN1_HUMAN              CF---EDLLPWLMETLTYEQSSVDRSGAAQGLAEVMAGLGVEKLEKLMPEIVATA
M7ANV9_CHEMY            CF---EDLLPWLMETLTYEQSSVDRSGAAQGLAEVMAGLGVEKLEKLMPEIVATA
H8X4E7_CANO9            QF---PGLIPKLIATLQDESKAGDRLGSAQALSEVICGLGTNKLEELLPSIISSA
W6A2P0_ICTPU            CF---DNLLPWLMETLASEQSSVDRSGAAQGLAEVMAGLGVEKLDKLMPDVVQTA
B6JZI6_SCHJY            NF---PSIIPELLSILKSDASEVDRQGAAQGLSEILAGLGLARLDDVFPDILANT
R4WJK2_RIPPE            SF---EDLLPWLMKTLTSETSSVDRSGAAQGLSEVVGGLGVEKLHKLMPEIISSA
W8CCK9_CERCA            SF---EDLLPWLMQTLTSETSSVDRSGAAQGLSEVVGGLGVEKLHQLMPDIIATA
B0W357_CULQU            SF---EDLLPWLMQTLTSESSSVDRSGAAQGLSEVVGGLGVEKLHKLMPEIIATA
E1ZX97_CAMFO            SF---EDLLPWLMQTLTSETSSVDRSGAAQGLSEVVRGLGVEKLHKLMPEIISTA
W5JJ28_ANODA            SF---EDLLPWLMQTLTSESSSVDRSGAAQGLSEVVGGLGVEKLHKLMPEIIATA
F1KPR4_ASCSU            SVRLREQIVPWLKEKLVSDASPVDRSGAAQGLAEVLKALGDDQLAYVMPDIIKTT
V9K7F0_CALMI            CF---DDLLPWLMEMLASEQSSVDRSGAAQGMAEVMAGLGVEKLEKLMPDIVATA
W0T7Q3_KLUMA            QF---PDLIPRLLATLSDNTKSGDRMGSAQALAEVISGLGLSKLDELLPTILSGV
S9R951_SCHOY            KF---PTLIPELFNVLRSECSEVDRQGAAQGLSEVLSGLGLTRLEDVLPEILQNT
S9W134_SCHCR            KF---PTLIPELFNILRSECSEVDRQGAAQGLSEVLSGLGLTRLEDVLPEILQNT
H0GUK0_SACCK            QF---PDLIPRLLDTLSDEFKSGDRLGSAQALAEVISGLGLTKLDEMLPTILAGV
E5R2E4_ARTGP            TL---PDLIPSLMATLKSEAGAGDRMGSAQALAEVLAGLGTSRLEDTLPSLLQNV
J8Q888_SACAR            KF---PDLISRLLDTLSDESKSGDRLGSAQALAEVISGLGLTKLDEMLPTILAGV
C5G992_AJEDR            AL---PDLIPSLMTTLKSDTGAGDRLGSAQALSEVLAGLGTSRLEETLPTILQNV
G4MR95_MAGO7            AL---PDLIPGLMQTLKSDTGAGDRLGSAQALSEVLAGLGTTRLEETLPTILQNV
C9SQ16_VERA1            AL---PDLIPGLMQTLKADTGAGDRLGSAQALSEVLAGLGTTRLEETLPTILQNV
B2WNI9_PYRTR            AL---PDLIPSLMATLKSDTGAGDRLGSAQALSEVLAGLGTGRLEETLPSILQNV
S3CN37_OPHP1            AL---PDLIPGLMQTLKSDTGAGDRLGSAQALSEVLAGLGTTRLEETLPTILQNV
G3ALT0_SPAPN            QF---PTLIPNLLATLQDERKAGDRLGSAQALAEVICGLGINKLEELLPNILASA
Q7S5P0_NEUCR            AL---PDLIPGLMQTLKSDTGAGDRLGSAQALSEVLAGLGTSRLEETLPTILQNV
A0A165FVX9_9PEZI        AL---PDLIPDLMTTLKSDTGAGDRLGSAQALSEVLAGLGTSRLEETLPTILQNV
A0A178DN93_9PLEO        AL---PDLIPSLMSTLKSDTGAGDRLGSAQALSEVLAGLGTGRLEETLPSILQNV
A0A177D7J2_ALTAL        AL---PDLIPSLMSTLKSDTGAGDRLGSAQALSEVLAGLGTGRLEETLPSILQNV
A0A0C4DS68_MAGP6        AL---PDLIPGLIQTLKSDTGAGDRLGSAQALSEVLAGLGTTRLEETLPTILQNV
A0A151N4J5_ALLMI        CF---EDLLPWLMETLTYEQSSVDRSGAAQGLAEVMAGLGVEKLEKLMPEIVATA
A0A146NKU7_FUNHE        CF---DDLLPWLMETLASEQSSVDRSGAAQGLAEVMAGLGVEKLDKLMPDVVQTA
A0A0F7Z917_CROAD        CF---EDLLPWLMETLTYEQSSVDRSGAAQGLAEVMAGLGVEKLEKLMPDIVATA
A0A0W0DC37_CANGB        QF---PDLIPRLLDTLNDETKSGDRLGSAQALAEVISGLGLSKLDELLPTVLAGA
A0A0L8RIK7_SACEU        QF---PDLIPRLLDTLSDESKSGDRLGSAQALAEVISGLGLTKLDEMLPTILAGV
A0A178FRD2_TRIVO        TL---PDLIPSLMATLKSEAGAGDRMGSAQALAEVLAGLGTSRLEDTLPSLLQNV
A0A175WD14_9PEZI        AL---PDLIPGLMQTLKSDTGAGDRLGSAQALSEVLAGLGTSRLEETLPTILQNV
A0A194VFU1_9PEZI        AL---PELIPQLMQTLKSDTGAGDRLGSAQALSEVLAGLGTTRLEETLPTILQNV
A0A151TXV6_CAJCA        NF---PDLVPWLFETLKSDNSNVERSGAAQGLSEVLAALGIVFFEHVLPDIIRNC
A0A0M9ABE9_9HYME        SF---EDLLPWLMQTLTSETSSVDRSGAAQGLSEVVRGLGVEKLHKLMPEIISTA
A0A072U5F0_MEDTR        NF---PDLVPWLFETLKSDNSNVERSGAAQGLSEVLAALGVEFFEHVFPDIIRNC
A0A0K8V1F4_BACLA        SF---EDLLPWLMQTLTSESSSVDRSGAAQGLAEVVGGLGVQKLHQLMPDIITTA
A0A0A1XE05_BACCU        SF---EDLLPWLMQTLTSESSSVDRSGAAQGLAEVVGGLGVEKLHQLMPDIITTA
A0A131YTA4_RHIAP        CF---EDLIPWLMQTLTSESSPVDRSGAAQGLSEVLGGLGVEKLQTLMPEIISTA
A0A0J6I2R9_COCPO        AL---PDLIPSLMTTLKSDTGAGDRLGSAQALSEVLAGLGTSRLEETLPSILQNV
A0A074XMM5_AURPU        AM---PDLIPSLMATLKSDTGAGDRLGSAQALSEVLAGLGTSRLEETLPTILQNI
A0A167FV98_9ASCO        QF---PDLIPRLMDTLKDESRSGDRLGSAQALSEVIYGLGIGKLEELLPVILKNC
A0A1A7XZM1_9TELE        CF---DDLLPWLMETLASEQSSVDRSGAAQGLAEVMAGLGVEKLDKLMPDVVQTA
A0A0P7V1U6_9TELE        CF---EDLLPWLMETLASEQSSVDRSGAAQGLAEVMAGLGVEKLEKLMPDVVQTA
A0A1A7WWE1_9TELE        CF---DDLLPWLMETLASEQSSVDRSGAAQGLAEVMAGLGVEKLDKLMPDVVQTA
                         .     ::. *   :  :    :* *:**.::*:: .*.   :   :  ::
```

```
GCN1_YEAST            TNF--RAYIREGFMPLLLFLPVCFGSQFAPYINQIIQPILSGLADNDENIRDTAL 2090
GCN1_SCHPO            SSP--VPHIRESFISLLIYLPATFGSRFQPYLARAIPPILSGLADDSELVQTASL
GCN1_DICDI            NSP--RPHVREGILSIFIFTPISLGDLFLPYLPKVLPQVLKGLADDSDPVREVCM
GCN1_MOUSE            SKVDIAPHVRDGYIMMFNYLPITFGDKFTPYVGPIIPCILKALADENEFVRDTAL
GCN1_HUMAN            SKVDIAPHVRDGYIMMFNYLPITFGDKFTPYVGPIIPCILKALADENEFVRDTAL
M7ANV9_CHEMY          SKVDIAPHVRDGYIMMFNYLPLTFGDKFTPYVGPIIPCILKALADENEFVRDTAL
H8X4E7_CANO9          SSP--RAAVRAGFMPLLLFLPVCFGSQFAPYLNRIIPPILNGLADQDEEIRETAL
W6A2P0_ICTPU          SKVDIASHVRDGYIMMFIYLPLTFGERFTPYVGPIIPCILKALADENEYVRDTAL
B6JZI6_SCHJY          SNG--NPSIRESFISLLIYLPATFGARFQPYLARAIPPILNGLADESDFVQSASL
R4WJK2_RIPPE          ERSDIAPHVKDGYIMMFIYMPVVFTNEFTPYIGEIIVPILKALADENEYVRDTAL
W8CCK9_CERCA          ERNDIAPHVKDGYIMMFIYMPGPFPKEFTPYIGQIINPILKALADENEYVRDTAL
B0W357_CULQU          ERTDIAPHVKDGYIMMFIYMPSAFPNDFTPYIGQIINPILKALADENEYVRDTAL
E1ZX97_CAMFO          ERTDIAPHVKDGYIMMFIYMPSAFTTEFTPYIGQIINPILKALADENEYVRETAL
W5JJ28_ANODA          ERNDIAPHVKDGYIMMFIYMPSAFPNDFTPYIGQIINPILKALADENEYVRDTAL
F1KPR4_ASCSU          ESEMVAPEVRDGYILMYIYLPMLFGDQFVPFLPQVVPSVLKALADENEYVRDSAL
V9K7F0_CALMI          SKVEIAPHVRDGYMMMFIYLPVTFGDKFTAYVGAIIPCILKALADENEFVRDTAL
W0T7Q3_KLUMA          TNY--RAYVREGFMPLMLFLPVCFGQQFAPYINQIIQPILSGLADTDENIRDTAL
S9R951_SCHOY          SSP--TAYIRESFISLLIYLPATFGARFQPYLARAIPPILAGLADESELVQTASL
S9W134_SCHCR          SSP--TPYIRESFISLLIYLPATFGARFQPYLARAIPPILSGLADESELVQTASL
H0GUK0_SACCK          TNF--RAYIREGFMPLLLFLPVCFGSQFASYINQIIQPILSGLADNDENIRDTAL
E5R2E4_ARTGP          SSS--KPTVREGFMTLFIFLPACFGNSFATYLNRIIPPILVGLADEVESIRETSL
J8Q888_SACAR          TNF--RAYVREGFMPLLLFLPVCFGSQFASYINQIIQPILSGLADNDENIRDTAL
C5G992_AJEDR          SSA--KASVREGFMSLFVFLPACFGNSFASYLNKIIPPILAGLADDVEAIRETSL
G4MR95_MAGO7          ESS--KPAVREGFMSLFIFLPVCFGNSFANYLGRIIPPILSGLADDIEAIRETAL
C9SQ16_VERA1          ESS--KPAVREGFMSLFIFLPVCFGNSFASYLGRIIPPILAGLADDVESIRETAL
B2WNI9_PYRTR          SSN--RASVREGFMSLFIFLPVCFGNSFANYLSKIIPPILGGLADDVESIRETAL
S3CN37_OPHP1          ESS--KPSVREGFMSLFIFLPVCFGNSFAAYLGKIIPPILAGLADDVDSIRETAL
G3ALT0_SPAPN          SSP--RSHIRAGFMPLLLFLPVCFGSQFSPYLNKIIPPILNGLADQDEEIRDTAL
Q7S5P0_NEUCR          ESS--KPAVREGFMSLFIFLPVCFGNSFANYLAKIIPPILSGLADDAESIRETAL
A0A165FVX9_9PEZI      SSS--KPAVREGFMSLFIFLPACFGNSFSAYLNRIIPPILAGLADEVDSIRETSL
A0A178DN93_9PLEO      ASN--KASVREGFMSLFIFLPACFGNSFANYLSKIIPPILGGLADDVESIRETAL
A0A177D7J2_ALTAL      ASN--RASVREGFMSLFIFLPVCFGNSFANYLSKIIPPILGGLADDVESIRETAL
A0A0C4DS68_MAGP6      ESS--KPAVREGFMSLFIFLPVCFGNSFANYLGRIIPPILSGLADDIEAIRETAL
A0A151N4J5_ALLMI      SKVDIAPHVRDGYIMMFNYLPITFGNKFTPYVGPIIPCILKALADENEFVRDTAL
A0A146NKU7_FUNHE      SKVDIASHVRDGYIMMFIYLPLTFGDKFTPYVGPIIPCILKALADENEYVRDTAL
A0A0F7Z917_CROAD      SKVDIAPHVRDGYIMMFNYLPITFGEKFTPYVGPIIPCILKALADENEFVRDTAL
A0A0W0DC37_CANGB      TNY--RSFVREGFMPLLLFLPICFGAQFAPYINQIIQPILAGLADIDENIRDTSL
A0A0L8RIK7_SACEU      TNF--RTYIREGFMPLLLFLPVCFGSQFASYINQIIQPILSGLADNDENIRDTAL
A0A178FRD2_TRIVO      SSS--KATVREGFMTLFIFLPACFGNSFAAYLNRIIPPILVGLADEVESIRETSL
A0A175WD14_9PEZI      ESA--KASVREGFMSLFIFLPVCFGNSFANYLGKIIPPILSGLADDVESIRDTAL
A0A194VFU1_9PEZI      ESS--KPAIREGFMSLFIFLPVCFGNSFANYLAKIIPAILSGLADDVESIRETAL
A0A151TXV6_CAJCA      SHL--KASVRDGYLTLFKYLPRSLGVQFQNYLPQVLPAILDGLADENESVRDAAL
A0A0M9ABE9_9HYME      ERTDIAPHVKDGYIMMFIYMPSAFTTEFTPYIGQIINPILKALADENEYVRETAL
A0A072U5F0_MEDTR      SHQ--KASVRDGYLTLFKYLPRSLGVQFQKYLPQVLPAILDGLADENESVRDAAL
A0A0K8V1F4_BACLA      ERTDIAPHVKDGYIMMFIYMPGPFPNDFTPYIGQIINPILKALADENEYVRDTAL
A0A0A1XE05_BACCU      ERTDIAPHVKDGYIMMFIYMPGPFPKDFTPYIGQIINPILKALADENEYVRETAL
A0A131YTA4_RHIAP      ERTDIAPHVKDGYVMMFIYLPGVFQKEFTPYISQIINPILKALADENEYVRETAL
A0A0J6I2R9_COCPO      SSA--KSAVREGFMSLFIFLPACFGNSFASYLNRIIPPILSGLADDVEAIRETSL
A0A074XMM5_AURPU      SSS--KPAVREGFMSLFIYLPACFGNSFSNYLSKIIPPILSGLADDIDTIRETAL
A0A167FV98_9ASCO      TSS--KSWIRQGYMPMLLYLPACFGASLSPYLNQIIPPILSGLADNVEDVRDTAL
A0A1A7XZM1_9TELE      SKVDIASHVRDGYIMMFIYLPLTFGDKFTPYVGPIIPCILKALADENEYVRDTAL
A0A0P7V1U6_9TELE      SKVDIASHVRDGYIMMFIYLPLTFGDKFTPYVGPIIPCILKALADENEYVRDTAL
A0A1A7WWE1_9TELE      SKVDIASHVRDGYIMMFIYLPLTFGDKFTPYVGPIIPCILKALADENEYVRDTAL
                            . :: . : :  : *  :   :  ::   :  :* .***  : ::  .:
```

```
GCN1_YEAST            KAGKLIVKNYATKAVDLLLPELERGMFDENDRIRLSSVQLTGELLFQVTGISSR- 2145
GCN1_SCHPO            RAAKMIVNNYATKSVDLLLPELEKGLFDNAWRIRLSSVQLVGDLVFKLAGINRK-
GCN1_DICDI            RCGQSIVLQFAVTGIEVIVPALEKVLFHENWRIRLSCVQLFGDLLFKLAGTTAQE
GCN1_MOUSE            RAGQRVISMYAETAIALLLPQLEQGLFDDLWRIRFSSVQLLGDLLFHISGVTGKM
GCN1_HUMAN            RAGQRVISMYAETAIALLLPQLEQGLFDDLWRIRFSSVQLLGDLLFHISGVTGKM
M7ANV9_CHEMY          RAGQRIISMYAETAIALLLPQLEQGLFDDLWRIRFSSVQLLGDLLFHISGVTGKM
H8X4E7_CANO9          RSGRLIVKNYAKKAVDLLLPELEAGLSNESYRIRLSSLELTGDLLFQITGLSGK-
W6A2P0_ICTPU          RAGQRIISMYAETAIALLLPELEQGLFDDLWRIRFSSVQLLGDLLFHISGVSGKM
B6JZI6_SCHJY          RAARMIINNYASKSVDLLLPELEKGLFDNYWRIRVSSVQLVGDLIFKLAGINKKS
R4WJK2_RIPPE          KAGQRIVNLYAESAITLLLPELEKGLFDDNWRIRYSSVQLLGDLLYRISGVSGKM
W8CCK9_CERCA          KAGQRIVNLYAESAVTLLLPELEKGLFDDNWRIRFSSVQLLGDLLYRISGVSGKM
B0W357_CULQU          KAGQRIVNLYAESAIALLLPELEKGLFDDNWRIRYSSVQLLGDLLYKISGVSGKM
E1ZX97_CAMFO          RAGQRIVTLYADSAIMLLLPELEKGLFDDNWRIRYSSVQLLGDLLYRISGVSGKM
W5JJ28_ANODA          KAGQRIVNLYAESAIALLLPELEKGLFDDNWRIRYSSVQLLGDLLYKISGVSGKM
F1KPR4_ASCSU          KAGQRLISTYCSHARRLLLPQLQAAMFDDNWRIRYASVTLIGDFLFNISGVSGKM
V9K7F0_CALMI          RAGQRIISMYAETAIALLLPELEQGLFDDNWRIRFSSVQLLGDLLFHVSGVTGKM
W0T7Q3_KLUMA          KAGKLIVKNYANKAIDLLLPELEHGMFDENERIRLSSVQLAGELLFQVTGISSK-
S9R951_SCHOY          RAAKMIVNNYATRAVDLLLPELEKGLFDDSWRIRLSSVQLVGDLIYKLAGINKKT
S9W134_SCHCR          RAAKMIVNNYATRAVDLLLPELEKGLFDDNWRIRLSSVQLVGDLIYKLAGINKKT
H0GUK0_SACCK          KAGKLIVKNYATKAVDLLLPELERGMFDENERIRLSSVQLTGELLFQVTGISSK-
E5R2E4_ARTGP          RAGRLLVKNFSTRSIDLLLPELERGLANDNYRIRLSSVELIGDLLFNLTG-ATA-
J8Q888_SACAR          KAGKLIVKNYATKAVDLLLPELERGMFDENDRIRLSSVQLTGELLFQVTGISSR-
C5G992_AJEDR          RAGRLLVKNFATKSIDLLLPELERGLADDNYRIRLSSVELVGDLLFNLTGIQNK-
G4MR95_MAGO7          KAGRLLVKNFAVRAVDLLLPELERGLADDSYRIRLSSVELVGDLLFNLTGISGK-
C9SQ16_VERA1          RAGRLLVKNFAMRAVDLLLPELERGLADDSYRIRLSSVELVGDLLFNLTGITGN-
B2WNI9_PYRTR          RAGRLLVKNFATKAIDLLLPELERGLADDSYRIRLSSVELVGDLLFNLTGISGK-
S3CN37_OPHP1          RAGRLLVKNFATRAVDLLLPELERGLADNSYRIRLSSVELVGDLLFNLSGISSK-
G3ALT0_SPAPN          RAGRLIVKNYAKKAVDLLLPELELGLSDPNYRIRLSSVELTGDLLFQITGISGK-
Q7S5P0_NEUCR          RAGRLLVKNFAVRAVDLLLPELERGLADDSYRIRLSSVELVGDLLFNLTGVKVT-
A0A165FVX9_9PEZI      RAGRLLVKNFATRSIDLLLPELERGLADDSYRIRLSSVELVGDLLFSLTGITAT-
A0A178DN93_9PLEO      RAGRLLVKNFATKAIDLLLPELERGLADDSHRIRLSSVELVGDLLFNLTGITGK-
A0A177D7J2_ALTAL      RAGRLLVKNFATKAIDLLLPELERGLADDSYRIRLSSVELVGDLLFNLTGISGK-
A0A0C4DS68_MAGP6      KAGRLLVKNFAVRAVDLLLPELERGLADDSYRIRLSSVELVGDLLFNLTGVSAN-
A0A151N4J5_ALLMI      RAGQRIISKYAETAIALLLPQLEQGLFDDLWRIRFSSVQLLGDLLFHISGVTGKM
A0A146NKU7_FUNHE      RAGQRIISMYAETAIALLLPELEQGLFDDLWRIRFSSVQLLGDLLFHISGVTGKM
A0A0F7Z917_CROAD      RAGQRIISMYAETAIALLLPQLEQGLFDDLWRIRYSSVQLLGDLLFHISGVSGKM
A0A0W0DC37_CANGB      KAGKLIVKNYATKAIDLLLPELERGVFDENERIRLSSVQLSGELLFQVTGISSR-
A0A0L8RIK7_SACEU      KAGKLIVKNYATKAVDLLLPELERGMFDENDRIRLSSVQLTGELLFQVTGISSK-
A0A178FRD2_TRIVO      RAGRLLVKNFSTRSIDLLLPELERGLANDNYRIRLSSVELIGDLLFNLTG-ATA-
A0A175WD14_9PEZI      RAGRLLVKNFAVRAVDLLLPELERGLADDSYRIRLSSVELVGDLLFNLAGIKVN-
A0A194VFU1_9PEZI      RAGRLLVKNFSVRAVDLLLPELERGLADDSYRIRLSSVELVGDLLFNLTGITGK-
A0A151TXV6_CAJCA      GAGHVLVEHYATTSLPLLLPAVEDGIFNDSWRIRQSSVELLGDLLFKVAGTSGKA
A0A0M9ABE9_9HYME      RAGQRIVNLYADSAIMLLLPELEKSLFDDNWRIRYSSVQLLGDLLYRISGVSGKM
A0A072U5F0_MEDTR      GAGHVLVEHYATTSLPLLLPAVEDGIINDSWRIRQSSVELLGDLLFKVAGTSGKA
A0A0K8V1F4_BACLA      KAGQRIVNLYAESAVMLLLPELERGLFDNNWRIRFSSVQLLGDLLYRISGVSGKM
A0A0A1XE05_BACCU      KAGQRIVNLYAESAVMLLLPELEKGLFDDNWRIRFSSVQLLGDLLYRISGVSGKM
A0A131YTA4_RHIAP      KAGQRMVNMYADTAMTLLLPQLEKGLFDDNWRIRYSSVQLLGDLLYKISGVTGKM
A0A0J6I2R9_COCPO      RAGRLLVKNFATKSIDLLLPELERGLADDSHRIRLSSVELVGDLLFNLTGINTK-
A0A074XMM5_AURPU      RAGRLLVKNFATRAIDLLLPELERGLADDSYRIRLSSVELVGDLLFNLSGISGT-
A0A167FV98_9ASCO      KAGRRLVRNYATKAVDLLLPELESGLSDRNHRIRLSSVELTGDLLFQVTGINSK-
A0A1A7XZM1_9TELE      RAGQRIISMYAETAIALLLPELEQGLFDDLWRIRFSSVQLLGDLLFHISGVTGKM
A0A0P7V1U6_9TELE      RAGQRIISMYAETAIALLLPELEQGLFDDLWRIRFSSVQLLGDLLFHISGVTGKM
A0A1A7WWE1_9TELE      RAGQRIISMYAETAIALLLPELEQGLFDDLWRIRFSSVQLLGDLLFHISGVTGKM
                       ..: ::  :.  .  :::* ::  : .   *** :.: * *:::: ::*
```

```
GCN1_YEAST            -----NEFS--EEDGDHNGEFSGKLVDVLGQDRRDRILAALFVCRNDTSGIVRAT 2200
GCN1_SCHPO            -----ALQEDEEEEGTHSDVSRKALLDIIGQERHDRILSTLYIVRQDIAAVVRTP
GCN1_DICDI            VQSNNSSYNAKDDDDDEPGSSGNDIQKILGKERLGRILSSLYMMRFDNNSSVRQK
GCN1_MOUSE            -----TTETASEDDNFGTAQSNKAIITALGVDRRNRVLAGLYMGRSDTQLVVRQA
GCN1_HUMAN            -----TTETASEDDNFGTAQSNKAIITALGVERRNRVLAGLYMGRSDTQLVVRQA
M7ANV9_CHEMY          -----TTETASEDDNFGTAQSNKAIISALGVERRNRVLAGLYMGRSDAQLVVRQA
H8X4E7_CANO9          -----NELI--EDQ-----TVSKTLAQVLGQERRDRILAALFVCRSDVAGMVRNA
W6A2P0_ICTPU          -----TTETASEDDNFGTAQSNKAIIGALGVDRRNRVLSGLYMGRSDTQLVVRQA
B6JZI6_SCHJY          V---EEEQQEEEENVTASDVKRKALIEAIGNDRHDRIMSALFIVRQDVSALVRAP
R4WJK2_RIPPE          -----STETANEDDNFGTEQSHKAIIGALGAERRNRVLAGLYMGRSDVSLMVRQA
W8CCK9_CERCA          -----TTETASEDDNFGTEQSHTAIIRFLGEERRNRVLSGLYMGRSDVSLMVRQA
B0W357_CULQU          -----TTQTASEDDNFGTEQSHKAIIRSLGAERRNRVLAGLYMGRSDVSLMVRQA
E1ZX97_CAMFO          -----STETASEDDNFGTEQSHYAIINALGAERRNRVLAGLYMGRSDVALMVRQA
W5JJ28_ANODA          -----TTQTASEDDNFGTEQSHKAIIRSLGGDRRNRVLAGLYMGRSDVSLMVRQA
F1KPR4_ASCSU          -----TSATSNEDDTMGMEAAGKAIVRQLGQACRDRVLAGIYLARSDVALTVRQV
V9K7F0_CALMI          -----TTETASEDDNFGTAQSNKAIINALGEDRRNRVLAGLYMGRSDTQLVVRQA
W0T7Q3_KLUMA          -----NEFD--EEDGDFNSEIGKQMVEVLGEERRARILSALFVCRSDVSGIVRAT
S9R951_SCHOY          ----LQEETDENEEGARTDVNRKALLETIGSERHDRVLASLYILRQDIAAAVRTN
S9W134_SCHCR          ----LQEETDESEEGTRTDVNRKALLETIGSERHDRVLSSLYILRQDIAAVVRTH
H0GUK0_SACCK          -----NEFS--EEDGDHNGEFSGKLVDVLGQDRRDRILAALFVCRNDISGIVRAT
E5R2E4_ARTGP          -----TDG---EEEIDSAIQAGQSLLEVLGEERRNKVLSSLYICRCDTSGLVRSA
J8Q888_SACAR          -----NEFS--EEDGDHNGEFSGKLVDVLGQDRRDRILAALFVCRNDTSGIVRAT
C5G992_AJEDR          -----GEE---DEEDDKAVQAGQSLLEVLGEDKRNKVLSSLYICRCDTSGLVRSA
G4MR95_MAGO7          -----TEDGD-EDEEEKVKEAGNSLREALGDEKRNKILSALYICRCDTATSVRAA
C9SQ16_VERA1          -----AEPG--EEEEEMAREAGASLREVLGEEKRNKILSALYVCRCDTANAVRSA
B2WNI9_PYRTR          -----VEE---DEVEEGAKEAGQSLLEVLGEEKRNKVLSALYICRCDSSGLVRTA
S3CN37_OPHP1          -----QEMEEGEMEEDAVREAGATLREVLGEDKRDKILSALYICRCDTSVAVRAA
G3ALT0_SPAPN          -----NELS--EDQFEVSGEVNKTLVEVLGQDRRDRVLASLFVSRADVAGIVRNA
Q7S5P0_NEUCR          -----DGEDE-EEDIETVKEAGASLREILGEEKRNKVLSSLYICRCDTSGAVRSA
A0A165FVX9_9PEZI      -----GEQ---EEDEEKALEAGHSLLEVLGEEKRNKVLSSLYICRCDTSGLVRTA
A0A178DN93_9PLEO      -----VEE---EEVEEGAKEAGQSLLEVLGEEKRNKVLSALYICRCDTSGLVRTA
A0A177D7J2_ALTAL      -----VEE---EEVEEGAKEAGQSLLEVLGEEKRNKVLSALYICRCDTSGLVRTA
A0A0C4DS68_MAGP6      -----QEPGD-EEEEERVKEAGASLREALGDEKRNKILSALYICRCDTAGAVRTA
A0A151N4J5_ALLMI      -----TTETASEDDNFGTAQSNKAIINALGVERRNRVLAGLYMGRSDTQLVVRQA
A0A146NKU7_FUNHE      -----TTETASEDDNFGTAASTKAIISALGAERRNRVLSGLYMGRSDTQLVVRQA
A0A0F7Z917_CROAD      -----TTETASEDDNFGTAQSNKAIINALGVGRRNRVLAGLYMGRSDTQLAVRQA
A0A0W0DC37_CANGB      -----NEF---SEEGEIGSEFSGKMVDVLGQERRDRVLSSLFVCRNDTSGIVRAS
A0A0L8RIK7_SACEU      -----NEFS--EEDGDHNGEFSGKLVDVLGQDRRDRILAALFVCRNDTSGIVRAT
A0A178FRD2_TRIVO      -----ADG---EEEIDSAIQAGQSLLEVLGEERRNKVLSSIYICRCDTSGLVRSA
A0A175WD14_9PEZI      -----TELGE-EEDQEMTKEAGTSLREILGEEKRNKILSALYVCRCDTAGAVRSA
A0A194VFU1_9PEZI      -----EDVD--EMEEEAIREHGTSLRETLGEEKRNKVLSALYICRNDTAVSVRSA
A0A151TXV6_CAJCA      -----LLEGGSDDEGSSTEAHGRAIIEVLGRDKRNEVLAALYMVRADVSLSVRQA
A0A0M9ABE9_9HYME      -----STETASEDDNFGTEQSHYAIINALGAERRNRVLAGLYMGRSDVALMVRQA
A0A072U5F0_MEDTR      -----LLEGGSDDEGSSTEAHGRAIIEVLGREKRNEILAALYMVRADVSLSVRQA
A0A0K8V1F4_BACLA      -----TTETASEDDNFGTEQSHTAIIRFLGEERRNRVLSGLYMGRSDVSLMVRQA
A0A0A1XE05_BACCU      -----TTETASEDDNFGTEQSHTAIIRFLGEERRNRVLSGLYMGRSDVSLMVRQA
A0A131YTA4_RHIAP      -----TTETAHEDDNFGTEQSHKAIMIALGEERRNRVLAGLYMGRLDTSLMVRQA
A0A0J6I2R9_COCPO      -----ADI---EEEDDTAAQAGQSLLEVLGEDKRNKVLSALYICRCDTSGLVRSA
A0A074XMM5_AURPU      -----AEQ---EEAEEGANEAGQSLLEVLGEEKRNKVLSALYICRCDTSGLVRTA
A0A167FV98_9ASCO      ---------NIDEEQVASSEINKTLIDVLGSQRRDRILSSLFICRSDTSAMVRNS
A0A1A7XZM1_9TELE      -----TTETASEDDNFGTAASNKAIIGALGAERRNRVLSGLYMGRSDTQLVVRQA
A0A0P7V1U6_9TELE      -----TTETASEDDNFGTAQSNKAIIGALGVERRNRVLSGLYMGRSDTQLVVRQA
A0A1A7WWE1_9TELE      -----TTETASEDDNFGTAASNKAIIGALGAERRNRVLSGLYMGRSDTQLVVRQA
                                 .            :   :*     .::: ::: * *    **
```

```
GCN1_YEAST            TVDIWKALVPNTPRAVKEILPTLTGMIVTHLASSSNVLRNIAAQTLGDLVRRVGG 2255
GCN1_SCHPO            AIQIWKAIVVNTPRTVREILPTLTSIIVSNLNSSSNDRRTMCVKSLGDLLKKAGF
GCN1_DICDI            VLLIWKYIVSNTPKTLREILPTLIEMIISSIGSNNVEKRQISAKTLGDIVSKLSD
GCN1_MOUSE            SLHVWKIVVSNTPRTLREILPTLFGLLLGFLASTCADKRTIAARTLGDLVRKLGE
GCN1_HUMAN            SLHVWKIVVSNTPRTLREILPTLFGLLLGFLASTCADKRTIAARTLGDLVRKLGE
M7ANV9_CHEMY          SLHVWKIVVSNTPRTLREILPTLFSLLLGFLASTCADKRTVAARTLGDLVRKLGE
H8X4E7_CANO9          SADIWKALVANTPRTVKEILPTLTSLIVQKLASDDETQRTIAAQTLGDMVRRVGA
W6A2P0_ICTPU          SLHVWKIVVSNTPRTLREILPTLFTLLLGFLASTFPDKRTIAARTLGDLVRKLGE
B6JZI6_SCHJY          ASQIWKAVVVNTPRTVKEIMPTLTSMIISNLNSSGNDRRVMCVKTLGELIRKIGF
R4WJK2_RIPPE          ALHVWKVVVTNTPRTLREILPTLFSLLLGCLASTSHDKRQVAAKTLGDLVRKLGE
W8CCK9_CERCA          ALHVWKVVVTNTPRTLREILPTLFGLLLGCLASTSYDKRQVAARTLGDLVRKLGE
B0W357_CULQU          ALHVWKVVVTNTPRTLREILPTLFSLLLGCLASTSYDKRQVAARTLGDLVRKLGE
E1ZX97_CAMFO          ALHVWKVVVTNTPRTLREILPTLFTLLLGCLASTSYDKRQVAARTLGDLVRKLGE
W5JJ28_ANODA          ALHVWKVVVTNTPRTLREILPTLFSLLLGCLASTSYDKRQVAARTLGDLVRKLGE
F1KPR4_ASCSU          ASHVWKIVVANTPRMLKEIMKTLFEMLLGCLASNSEDRQQMAARCLGELVKKMGE
V9K7F0_CALMI          SLHVWKIVVSNTPRTLREVLPTLFGLLLGFLASTCADKRTIAARTLGDLVRKLGE
W0T7Q3_KLUMA          TVDIWKALVPNTPRTIKEILPELTSTIVVHLASSSRTLRLIAAQTLGDLVRRVGG
S9R951_SCHOY          AQQIWKNIVVNTPRTVRDIITTLTSLIIGNLNSAGNDRRAMCVKSLGDLSKKAGF
S9W134_SCHCR          AQQIWKNIVVNTPRTVRDIMSTLTALIVGNLNSTGNDRRAMCVKSLGDLLKKAGF
H0GUK0_SACCK          TVDIWKALVPNTPRAVKEILPTLTGMIVTYLASSSSVLRNIAAQTLGDLVRRVGG
E5R2E4_ARTGP          AINVWKALVA-TPRTLKELVPTLSQVIIRRLGSSNMEQKIIAGNALGELIKKAGE
J8Q888_SACAR          TVDIWKALVPNTPRAVKEILPTLTGMIVTHLASSSKTLRNIAAQTLGDLVRRVGG
C5G992_AJEDR          AIAVWKALVA-TPRTLRELVPTLSQLIIRRLASPNMEQKVIAGNALGELIKKAGE
G4MR95_MAGO7          AVAVWKALVS-SPKTLKELVPTLTQLIIRRLGSTNMEHKVIASNALGELIRKAGD
C9SQ16_VERA1          AIGVWKALVS-SPRTLKELVPTLTQLIIRRLGSSNMEHKVIASNALGELIRKAGD
B2WNI9_PYRTR          SINVWKALVA-SPRTLRELIPTLTQLIIRRLASSSMEQKHIASSALGELIRKAGD
S3CN37_OPHP1          AISVWKALVS-SPRTLKELVPTLTQLIIRRLGSANMEHKVIASNALGELIRKAGD
G3ALT0_SPAPN          AVDIWKALVANTPRTVKEILPSLTAIIVRKLASGDDVERTIAAQSLGEMVRRVGS
Q7S5P0_NEUCR          AVSVWKALVH-SPRILKELVPTLTQLIIRRLGSSNMEHKIIASNALGELIRKAGD
A0A165FVX9_9PEZI      AVNVWKALVA-SPRILKELIPTLTQLIIRRLASSNMEHKVIAGNALGELIRKAGE
A0A178DN93_9PLEO      AINVWKALVA-TPRTLRELVPTLTQLIIRRLASSNMEQKVIAGNALGELIRKAGD
A0A177D7J2_ALTAL      SINVWKALVA-SPRTLRELIPTLTQLLIRRLASSNMEQKVIAGNALGELIRKAGD
A0A0C4DS68_MAGP6      AIAVWKALVS-SPRTLKELVPTLTQLIIRRLGSFNMEHKVIASNALGELIRKAGD
A0A151N4J5_ALLMI      SLHVWKIVVSNTPRTLREILPTLFGLLLGFLASTCADKRSIAARTLGDLVRKLGE
A0A146NKU7_FUNHE      SLHVWKIVVSNTPRTLREILPTLFSLLLGFLASTCPDKRTIAARTLGDLVRKLGE
A0A0F7Z917_CROAD      SLHVWKIVVSNTPRTLREILPTLFGLLLGFLASTCADKRTIAARTLGDLVRKLGE
A0A0W0DC37_CANGB      TVDIWKALVPNTPRTVKEILPVLTDMIVTNLASSSNTLRNIAAQTLGDVVRRVGG
A0A0L8RIK7_SACEU      TVDIWKALVPNTPRAAKEILPTLTGMIVTHLASPSHTLRNIAAQTLGDLVRRVGS
A0A178FRD2_TRIVO      AINVWKALVA-TPRTLKELVPTLSQVIIRRLGSSNMEQKVIAGNALGELIKKAGE
A0A175WD14_9PEZI      AIGVWKALVH-SPRTLKELVPTLTQLIIRRLGSSNMEHKVIASNALGELIRKAGD
A0A194VFU1_9PEZI      AVGVWKALVS-SPKTLKELVPTLSQLIIRRLGSSNMEHRVIASNALGELIRRAGD
A0A151TXV6_CAJCA      ALHVWKTIVANTPKTLREIMPVLMDTLITSLASSSSERRQVAGRSLGELVRKLGE
A0A0M9ABE9_9HYME      ALHVWKVVVTNTPRTLREILPTLFTLLLGCLASTS--------------------
A0A072U5F0_MEDTR      ALHVWKTIVANTPKTLREIMPVLMDTLIASLASASSERRQVAGRSLGELVGKLGE
A0A0K8V1F4_BACLA      SLHVWKVVVTNTPRTLREILPTLFSLLLGCLASTSYDKRQVAARTLGDLVRKLGE
A0A0A1XE05_BACCU      SLHVWKVVVTNTPRTLREILPTLFSLLLGCLASTSYDKRQVAARTLGDLVRKLGE
A0A131YTA4_RHIAP      SLHVWKVVVTNTPRTLREILPTLFSLLLGFLASSSYDKQQVAARTLGDLVRKLGE
A0A0J6I2R9_COCPO      AINVWKALVA-SPRTLKELVPTLTQLIIRRLGSANMEQKVIAGNALGELIKKAGE
A0A074XMM5_AURPU      SINVWKALVA-SPRTLRELIPTLTQLLIHRLASSNMEQKEIAGNALGELIRKAGE
A0A167FV98_9ASCO      ALEVWKCLVANTPRTVKEILPSLTQMIIKRLASSDDEQRTIAAQTLGDLVRRVGG
A0A1A7XZM1_9TELE      SLHVWKIVVSNTPRTLREILPTLFSLLLGFLASTCPDKRTIAARTLGDLVRKLGE
A0A0P7V1U6_9TELE      SLHVWKIVVSNTPRTLREILPTLFSLLLGFLASTCPDKRTIAARTLGDLVRKLGE
A0A1A7WWE1_9TELE      SLHVWKIVVSNTPRTLREILPTLFSLLLGFLASTCPDKRTIAARTLGDLVRKLGE
                         :** :*  :*:  ::::  *   ::  : *
```

```
GCN1_YEAST          NALSQLLPSLEESLIETSNSDSRQGVCIALYELIESASTETISQFQSTIVNIIRT 2310
GCN1_SCHPO          DVLPQLLPVLKQGL-ESANSGDRIGVCIALEELINSATPEQLEIYSDDFVYAVRR
GCN1_DICDI          RILPEILPILERGL-RSELEETRQGVCIGLSEVISSA-KTQLLPYLSSVVTCITK
GCN1_MOUSE          KILPEIIPILEEGL-RSQKSDERQGVCIGLSEIMKSTSRDAVLFFSESLVPTARK
GCN1_HUMAN          KILPEIIPILEEGL-RSQKSDERQGVCIGLSEIMKSTSRDAVLYFSESLVPTARK
M7ANV9_CHEMY        KILPEIIPILEEGL-RSEKSDERQGVCIGLSEIMKSTSRDAVLFFSESLVPTVRK
H8X4E7_CANO9        NALPQLLPSLKEAK-------DQEGACIALTELIKSTSFDGLTTYKDTFISIIYN
W6A2P0_ICTPU        KILPEIIPILEEGL-RSEKSDERQGVCIGLSEIMKSTSKDAVLVFSESLVPTVRK
B6JZI6_SCHJY        DVMEQLLPSLENGR-LSTNPQDRIGVCIAITELINSCAPEQLENYASTITNAIRG
R4WJK2_RIPPE        RVLPEIIPILEQGL-ESDQPDQRQGVCIGLSEMMASTSRDMVLTFVNSLVPTVRK
W8CCK9_CERCA        RILPEIIPILEKGL-NSEHADQRQGVCIGLSEIMASTSREMVLSFVNSLVSTVRK
B0W357_CULQU        RVLPEIIPILERGL-NSDQADQRQGVCIGLSEIMASTSRDMVLTFVNSLVPTVRK
E1ZX97_CAMFO        RVLPEIIPILEKGL-QSDQADQRQGVCIGLSEIMASTNKDMVLTFVISLVPTVRK
W5JJ28_ANODA        RVLPEIIPILERGL-NSDQADQRQGVCIGLSEIMASTSRDMVLTFVNSLVPTVRK
F1KPR4_ASCSU        RILIDVLPVLELGL-ESPSVEQRQGVAIALAEIIENTTRDVVIMYTPQLVEPIKK
V9K7F0_CALMI        KILPDIIPILEEGL-RSEKSDERQGVCIGLSEIMKSTSRDAVLVFSESLVPTVRK
W0T7Q3_KLUMA        NALSQLLPTLKHSLETSSDPNSKQGVCIALHELIQSSNSDSLDEFQDFIVNIICS
S9R951_SCHOY        DVLSQLLPSLKDGL-ESSDRENRIGVCIALQELISSTSQDQLEIYSDQFVYSVRE
S9W134_SCHCR        DVLSQLLPSLKAGL-ESSDRQDRIGVCIALQELISSASHDQLEIYSDQFVYSVRE
H0GUK0_SACCK        NALSQLLPSLEESLIETSNPDSRQGVCIALYELIESASAETISQFQSIIVNIIRT
E5R2E4_ARTGP        GVLSTLLPELEEGLITSTDIDGRQGICLAVRELVVSSSDESLETYEKPLISIVKT
J8Q888_SACAR        NALSQLLPSLEESLEETSNPDSRQGVCIALYELIESASAETISQYQSIIVNIIRA
C5G992_AJEDR        GVLSTLLPSLEAGLVASTDVDSRQGICIALRELVVSATAESLEDYEKILISIVRT
G4MR95_MAGO7        NVLSSLLPTLEEGLQTSTDVDARQGICLALKELISSASEEALEEHEKILISVVRT
C9SQ16_VERA1        GVLSTLLPTLEEGLQTSTDSDSKQGICLALKELISSASEEALEDHEKTLISVVRT
B2WNI9_PYRTR        GVLATLLPTLEDGL-HTTDTDAKQGICIALRELIDAASPEQLEDYEKTLIKVVRT
S3CN37_OPHP1        NILQSLLPTLAEGLKTSTDSDAKQGICLALKELIASAAPESLEDHEATLISIVQS
G3ALT0_SPAPN        NALEQLLPTLEESF-DTTDKSAKQGICIALTELIKSTPTEGLYNYQETFISIIKQ
Q7S5P0_NEUCR        GVLATLLPTLEEGLQTSSDVDARQGICLALKELIASASPEALEDHEKTLISVVRT
A0A165FVX9_9PEZI    GVLATLLPYLEEGLQTSTDADARQGICIALRELIASASPEAIEDYEKTLISIVRT
A0A178DN93_9PLEO    GVLATLLPTLEEGL-HTTDTDAKQGICIALRELIASASPEQLEDYEKTLIQVVRT
A0A177D7J2_ALTAL    GVLATLLPTLEEGL-HTTDTDAKQGICIALRELIAAASPEQLEDYEKTLIQVVRT
A0A0C4DS68_MAGP6    NVLATLLPTLEEGLQTSTDVDARQGICLALKELISSASEEALEDHEKTLISVVRT
A0A151N4J5_ALLMI    KILPEIIPILEEGL-RSEKSDEREGVCIGLSEIMKSTSRDAVLFFSVSLVPTVRK
A0A146NKU7_FUNHE    KILPEIIPILEDGL-RSDKSDERQGVCIGLSEIMKSTSKDAVLVFSESLVPTVRK
A0A0F7Z917_CROAD    KILPEIIPILEEGL-RSEKSDKRQGVCIGLSEIMKSTSRDAVLCFSESLLPTVRK
A0A0W0DC37_CANGB    NAMAQLLEALEVTLEKTSNPDSREGVCIALNELVMSASMDTLTQYQDTVANILRR
A0A0L8RIK7_SACEU    NALSQLLPSLEESLVETPNPDSRQGVCIALHELIESASAETISQFQSIIVNIIRT
A0A178FRD2_TRIVO    GVLSTLLPELEEGLITSTDIDGRQGICLAVRELVVSSSEESLETYEKALISIVKT
A0A175WD14_9PEZI    GVLATLLPTLEEGLQTSRDVDAKQGICLALKELISSASQDALEDHEKTLISVVRT
A0A194VFU1_9PEZI    GVLASLLPTLEEGLQSSTDTDAKQGICLALKELISSASPEALEDHEKILISVVRT
A0A151TXV6_CAJCA    RVLPLIIPILSQGL-NDPDSSRRQGVCVGLSEVMASAGKSQLLSFMNELIPTIRT
A0A0M9ABE9_9HYME    -------------L-QSDQADQRQGVCIGLSEIMTSTNKDMVITFVISLVPTVRK
A0A072U5F0_MEDTR    RVLPLIIPILSQGL-SDPDSSRRQGVCSGLSEVMASAGKSQLMTFMTDLIPTIRT
A0A0K8V1F4_BACLA    RVLPEIIPILEKGL-NSEQPDQRQGVCIGLSEIMSSTSKEMVLSFVNSLVPTVRR
A0A0A1XE05_BACCU    RVLPEIIPILEKGL-NSEYPDQRQGVCIGLSEIMASTSKEMVLSFVNSLVPTVRK
A0A131YTA4_RHIAP    RVLPEIVPILEQGL-DSDLPDQRQGVCVGLSEILASTSRDMVLTFLDSLVPTVRR
A0A0J6I2R9_COCPO    GVLSTLLPSLEEGLLASTEVDARQGICIALRELVISSSGESLEVYEKILISTVRT
A0A074XMM5_AURPU    GVLSTLLPTLEQGL-QTTDVDAKQGICIALRELIASASPDSLEDHEKTLVSVVRI
A0A167FV98_9ASCO    NALGSLLPTLEEEM-LSSDGDAKQGICLALIELVKSTSPEALIEYQQTIVGIVRN
A0A1A7XZM1_9TELE    KILPEIIPILEEGL-RSDKSDERQGVCIGLSEIMKSTSKDAVLVFSESLVPTVRK
A0A0P7V1U6_9TELE    KILPEIIPILEEGL-RSDKSDERQGVCIGLSEIMKSTSKDAVLVFSESLVPTVRK
A0A1A7WWE1_9TELE    KILPEIIPILEEGL-RSDKSDERQGVCIGLSEIMKSTSKDAVLVFSESLVPTVRK
                                          : * . .: *::       :  .   .
```

```
GCN1_YEAST          ALIDESATVREAAALSFDVFQDVVGKTAVDEVLPYLLHMLESSDN---SDFALLG 2365
GCN1_SCHPO          ALMDGDLEVRETAAEAFDSLQSILGDRAVDDVLPQLLKLLESENQ---SEQALSA
GCN1_DICDI          ALCDPLIDVREAAAKAFDHLYHTFGSKASNEILPQLIQLLDNSNNKDLAGYALDG
GCN1_MOUSE          ALCDPLEEVREAAAKTFEQLHSTIGHQALEDILPFLLKQLDDEEV---SEFALDG
GCN1_HUMAN          ALCDPLEEVREAAAKTFEQLHSTIGHQALEDILPFLLKQLDDEEV---SEFALDG
M7ANV9_CHEMY        ALCDPLEEVREAAAKTFEQLHSTIGYQALEDILPFLLKQLDTEET---SDFALDG
H8X4E7_CANO9        GLITQDKPTRNAAAQAFEQLYEQIGKVVIDGIIPQLLSELSN------SSASLLA
W6A2P0_ICTPU        ALCDPLEEVREAAAKTFEQLHATIGHQALEDILPALLKQLEDEET---AEFALDG
B6JZI6_SCHJY        ALVDSDASVRSVAAEAFDSLQNAIGNKAIDEVLPELLILLQSDEK---SEFALSA
R4WJK2_RIPPE        ALCDPLPEVRQAAAKTFDSLHSTVGVRALDDILPPMLQQLNNGDSG-VSEWTLDG
W8CCK9_CERCA        ALSDPLPEVREAAAKTFESLHSTVGSRALDDILPSMLDGLNDPDPA-IAEHTLDG
B0W357_CULQU        ALADPLPEVRQAAAKTFDSLHTTVGSRALDDILPSMLESLSDPDPD-VAEWTLDG
E1ZX97_CAMFO        ALCDPLPEVRQAAAKTFDGLHSTVGVRALDDILPAMLTQLNSPDPA-EAENTLDG
W5JJ28_ANODA        ALADPLPEVRQAAAKTFDSLHTTVGSRALEDILPSMLESLSDPDPD-VAEWTLDG
F1KPR4_ASCSU        AISDPEMEVRKAAAATFTSFCQSVGSSAFDDIVAPLLDSLGEQDD-----CLLDG
V9K7F0_CALMI        ALCDPLEEVREAASKTFEQLHSTIGYQALEDILPSLLQQLDDEEM---SEFALDG
W0T7Q3_KLUMA        TVIDEDESVREAAASCFDVYQEVVGKVAIDEIIPFLLNKLKEPES---SEYALSA
S9R951_SCHOY        ALMDSDLEVREAAAEAFDTLQTVIGDRAVDEVLPELLKLLESDDK---SEHALSA
S9W134_SCHCR        ALMDSDLEVRETAAEAFDILQTVIGDRAVDEVLPELLKLLESDDK---SEYALSA
H0GUK0_SACCK        ALIDESATVRQAAALSFDVFQDVVGKTAVDEVLPYLLHMLESSDN---SGFALLG
E5R2E4_ARTGP        ALVDTNDQVREAAAAEAFDALQQALGKRIVDKVLPDLLHLLHNEND---AEQALAA
J8Q888_SACAR        ALVDESATVRQAAALSFDVFQDVVGKTAVDEVLPYLLHMLESSDN---SGFALLG
C5G992_AJEDR        ALVDHDEAVREAAAAEAFDALQQVLDKRAVDQVLPDLLHLLRSEAD---AQQALSA
G4MR95_MAGO7        ALTDSDTEVREAAAAEAFDSLQQILGKRAVDQVLPFLLNLLRSEDE---AENALSA
C9SQ16_VERA1        ALTDSDPEVREAAAAEAFDSLQQILGKKAVDQVLPYLLNLLRSENE---ADNALSA
B2WNI9_PYRTR        ALVDPNVDVREAAAAEAFDALQQIFGKTAVDEVLPYLLNLLRSDND---AQNALSA
S3CN37_OPHP1        ALTDSDEDVREAAAAEAFDSLQQVIGKRAVDQVMPSLLHLLQNEEE---ADNALAG
G3ALT0_SPAPN        ALVDSDSEVREAAAQAFEGLQTELGKVVIDEVLPDLLKMLEGDD----SQHALLA
Q7S5P0_NEUCR        ALTDRDEDVREAAAAEAFDSLQQILGKRAVEEVMPFLLTLLANEEE---AENALAA
A0A165FVX9_9PEZI    ALVDTDEDVREAAAAEAFDSLQKIIGKKAVDQVLPHLLSLLRTEGE---ADNALSA
A0A178DN93_9PLEO    ALVDPNADVREAAAAEAFDALQQILGKRAVDQVLPYLLNLLRSEDD---AQHALSA
A0A177D7J2_ALTAL    ALVDPNADVREAAAAEAFDALQQIFGKRAVDQVLPYLLNLLRSDDD---AQNALSA
A0A0C4DS68_MAGP6    ALTDSDDDVREAAAAEAFDSLQQILGKRAVDQVLPYLLSLLRSEDE---AENALSA
A0A151N4J5_ALLMI    ALCDPLEEVREAAAKTFEQLHSTIGHQALEDILPFLLKQLDVEET---SEFALDG
A0A146NKU7_FUNHE    ALCDPLEEVREAAAKTFEQLHATIGHQALDDILPTLLKQLDDEDT---AEFALDG
A0A0F7Z917_CROAD    ALCDPLEEVREAAAKTFEQLHSTIGYQALEDILPFLLKQLDCEET---SEFALDG
A0A0W0DC37_CANGB    TLIDSNESVRQAAALSFDSYQEAVGKVAVDEVIPYLLNALKSNEN---SEYALLG
A0A0L8RIK7_SACEU    ALIDESATVRQAAAQSFDVFQDVVGKTAVDEVLPYLLHMLESSDN---SGFALLG
A0A178FRD2_TRIVO    ALVDTNDQVREAAAAEAFDALQQALGKRIVDRVLPDLLHLLHNEND---AEQALAA
A0A175WD14_9PEZI    ALTDSDADVREAAAAEAFDSLQQILGKRAVDQVLPYLLNLLRSEEE---ANNALAA
A0A194VFU1_9PEZI    ALTDSDEEVREAAAAEAFDSLQQILGKHAVDQVLPFLLNLLRSDEN---ADNALQA
A0A151TXV6_CAJCA    ALCDSVSEVRESAGLAFSTLYKSAGMLAIDEIVPTLLHALEDDET---SDTALDG
A0A0M9ABE9_9HYME    ALCDPLPEVRQAAAKTFDGLHSTVGVRALDDILPAMLTQLNSPDSA-EAENTLDG
A0A072U5F0_MEDTR    ALCDSEPAVRESAGLAFSTLYKSAGMQAIDEIVPTLLHALEDDKT---SDTALDG
A0A0K8V1F4_BACLA    ALSDPLPEVRGAAAKTFESLHSTVGSRALDDILPSMLEGLNDPDPT-VAEYTLDG
A0A0A1XE05_BACCU    ALSDPLPEVRGAAAKTFESLHSTVGSRALDDILPSMLEGLNDPDPN-VSEYTLDG
A0A131YTA4_RHIAP    ALCDPLKEVRVAAAKTFDNLHSTVGSRALDDILSPLLMQLGHGDSV-LAENTLDG
A0A0J6I2R9_COCPO    ALLDSNQDVREAAAAEAFDALQQALGKRIVDRVLPDLLNLLHTDAE---ADRALAA
A0A074XMM5_AURPU    ALVDGDEDVREAAAAEAFDSLQKVLGKRAIDQVLPYLLNLLRSDED---ADNALSA
A0A167FV98_9ASCO    ALTDSDSNVREAAGHAFDALQESLDEAA-DDILPDLIEQMQNGDE-----SALSA
A0A1A7XZM1_9TELE    ALCDPLEEVREAAAKTFEQLHATIGHQALDDILPTLLKQLDDEET---AEFALDG
A0A0P7V1U6_9TELE    ALCDPLEEVREAAAKTFEQLHATIGHQALDDILPALLRQLDDEES---AEFALDG
A0A1A7WWE1_9TELE    ALCDPLEEVREAAAKTFEQLHATIGHQALDDILPTLLKQLDDEET---AEFALDG
                     :      .*  *.  *       .    : ::. ::  :            * .
```

```
GCN1_YEAST               LQEIMS--KKSDVIFPILIPTLLAPPIDAFRASALGSLAEVAGSALYKRLSIIIN 2420
GCN1_SCHPO               LREIIS--RRSSTIFPVLIPTLIKKPVSAFNARALSSLAQVAGVTLNKRLPSILN
GCN1_DICDI               LRQVIL--VRSSIVLPVLIPKLLSRPISTSNVTALSSLAADAGEGLYVHLSTIIP
GCN1_MOUSE               LKQVMA--VKSRVVLPYLVPKLTTPPV---NTRVLAFLSSVAGDALTRHLGVILP
GCN1_HUMAN               LKQVMA--IKSRVVLPYLVPKLTTPPV---NTRVLAFLSSVAGDALTRHLGVILP
M7ANV9_CHEMY             LKQVMA--VKSRVVLPYLVPKLISPPV---NTRVLAFLSSVAGEALTRHLGVILP
H8X4E7_CANO9             LKELMA--SKSDIIFPITLPSLLEPPVD---AVALASLASVAGTALYKRLSTILN
W6A2P0_ICTPU             LKQVMA--VKSRSVLPYLVPKLTAPPV---NTRVLAFLSAVTGDALTRHLGVILP
B6JZI6_SCHJY             LQEIIT--RRSTSIFPVLIPTLIKQPISAFNARALASLATAAGATLLRRLPSILT
R4WJK2_RIPPE             LRQVMA--IKSRVVLPYLVPQLTATPI---NTKALSILASVAGEALTKYLNKILP
W8CCK9_CERCA             LRQMMT--IKSRVVLPFLIPQLTAAPV---NTKALSILVSVAGDALTKYLPKILD
B0W357_CULQU             LRQVMA--IKSRVVLPYLIPQLTANPV---NTKALSILASVAGEALTKYLPKILP
E1ZX97_CAMFO             LRQVMA--IKSRVVLPYLVPQLASPPV---NTKALSILASVAGEALTRFLHRILP
W5JJ28_ANODA             LRQVMA--IKSRVVLPYLIPQLTATPV---NTKALSILASVAGEALTKYLPKILP
F1KPR4_ASCSU             LSQIMR--MNSRQMLSYVLPKLTRPPI---NARALCALSAVAGDSLTRNLGRILE
V9K7F0_CALMI             LKQVMA--VKSRVVLPYLVPKLTAPPV---NTRVLAFLSSVAGDALTRHLSVILP
W0T7Q3_KLUMA             LQEIMA--TKSEVIFPILIPTLLKPPIDSFKANALGSLAEVAGPALYKRISTIIN
S9R951_SCHOY             LKEIIA--RRSTSIFPVLIPTLIKQPITAFNARALASLAQAAGSTLNRRLPSILN
S9W134_SCHCR             LKEIIA--RRSASIFPVLIPTLIKQPISAFNARALASLAQAAGPTLNRRLPSILN
H0GUK0_SACCK             LQEIMS--KKSDVIFPILIPTLLAPPIDAFRASALGSLAEVAGSALYRRLSIIIN
E5R2E4_ARTGP             LLTLLTEATRANIILPNLIPTLLTKPVTGFNAKALASLSKVAGGGMNRRLPTILN
J8Q888_SACAR             LQEIMS--KKSDVIFPILIPTLLAPPMDAFRSSALGSLAEVAGSALYKRLSVIIN
C5G992_AJEDR             LLTLLTETTRANIILPNLIPTLLTLPISGFNARALASLAEVASSSMTRRLPAILN
G4MR95_MAGO7             LLTLLTETTRSNIILPNLIPTLIAPPISAFNAKALASLSRVAGAAMNRRLPNIVN
C9SQ16_VERA1             LLTLLTETTRSNIILPNLIPTLITPPITAFDAKALASLSRVAGAAMNRRLPNIIN
B2WNI9_PYRTR             LLTLLTDQARSNIILPNLLPTLLTSPMSAFNARAIASLAEVASSAMTRRLPNILN
S3CN37_OPHP1             LLTLLTETSRSNIILPSLIPTLIAPPISAFNAKALASLSRVAAGAMNRRLPNIVN
G3ALT0_SPAPN             LKDIMA--TKADVIFPILIPTLLAPPMDAFKASAISSLASVAGSALYRRLSLIIN
Q7S5P0_NEUCR             LLTLLTETTRSNMILPNLIPSLIKPPISSFNAKALASLSKVAGAAMNRRLPSIIN
A0A165FVX9_9PEZI         LLTLLTENTRANIILPILIPTLLTSPISAFNAKAVASLSQVASGALTRRLPNIIN
A0A178DN93_9PLEO         LLTLLTDQARSNIILPNLLPTLLTSPMSAFNARAIASLAEVASSAMTRRLPNILN
A0A177D7J2_ALTAL         LLTLLTDQARSNIILPNLLPTLLTSPMSAFNARAIASLAEVASSAMTRRLPNILN
A0A0C4DS68_MAGP6         LLTLLTETTRSNIILPNLIPTLIAPPISAFNAKALASLSKVAGAAMNRRLPNIIN
A0A151N4J5_ALLMI         LKQVMA--VKSRVVLPYLVPKLIAPPV---NTRVLAFLSSVAGDALTRHLTVILP
A0A146NKU7_FUNHE         LKQVMA--VKSRSVLPYLVPKLTAPPV---NTRVLAFLSAVAGDALTRHLGVILP
A0A0F7Z917_CROAD         LKQVMA--VKSRVVLPYLVPKLISPPV---NTRVLAFLSTVAGDALTRHLGVILP
A0A0W0DC37_CANGB         LQDIMA--TKSDVIFPILMPTLLASPIDSFRASALGSLAEVAGPALYKRLSVIIN
A0A0L8RIK7_SACEU         LQEIMS--KKSDVIFPILIPTLLSPPIDAFRASALGSLAEVAGSALYKRLSIIVN
A0A178FRD2_TRIVO         LLTLLTEATRANIILPNLIPTLLTKPITGFNAKALASLSKVAGGGLNRRLPTILN
A0A175WD14_9PEZI         LLTLLTESTRSNIILPNLIPTLITPPISAFNAKALASLSKVAGAAMNRRLPNIIN
A0A194VFU1_9PEZI         LLTLLTETTRSNIILPNLIPTLIAPPISAFNAKALASLSKVAGAAMNRRLPGIIN
A0A151TXV6_CAJCA         LKQILS--VRTSAVLPHILPKLVHPPLSAFNAHALGALAEVAGPGLNFHLATVLP
A0A0M9ABE9_9HYME         LRQVMA--IKSRVVLPYLVPQLTTPPV---NTKALSILASVAGEALTRFLHKILP
A0A072U5F0_MEDTR         LKQILS--VRTSAVLPHILPKLVHPPLSAFNAHALGALAEVAGPGLDFHLGTVLP
A0A0K8V1F4_BACLA         LRQVMT--IKSRVVLPYLVPQLTAPPV---NTKALSILVSVAGDALTKYLPKILD
A0A0A1XE05_BACCU         LRQVMT--IKSRVVLPYLVPQLTAVPV---NTKALSILVSVAGDALTKYLPKILD
A0A131YTA4_RHIAP         LRQVMA--IKSRVVLPYLIPQLTTPPV---NTKALSHLSAVAGESLSRHLPKILP
A0A0J6I2R9_COCPO         LLTLLTETTRANIILPNLIPSLLASPMTSFNAKALASLAEVTGGALTRRLPNILN
A0A074XMM5_AURPU         LLTLLTEQTRSNVILPNLLPTLLTPPITAFNAKALASLAQVASSAMTRRLPNILN
A0A167FV98_9ASCO         LKEMMS--TKSDVVFPVLIPTLLTPPMTPFKARSLASVVAVSGVALYKRLTSILN
A0A1A7XZM1_9TELE         MKQVMA--VKSRSVLPYLVPKLTAPPV---NTRVLAFLSAVAGDALTRHLGVILP
A0A0P7V1U6_9TELE         LKQVMA--VKSRSVLPYLVPKLTAPPV---NTRVLAFLSAVAGDALTRHLGVILP
A0A1A7WWE1_9TELE         MKQVMA--VKSRSVLPYLVPKLTAPPV---NTRVLAFLSAVAGDALTRHLGVILP
                         :  ::     .:  ::.   :* *    *:       :   :   :.  :   :   ::
```

```
GCN1_YEAST          ALVDAII-GTSEDESTKGALELALDRVFLSVNDDEGLHPLLQQIMSLLKSDNIEK 2475
GCN1_SCHPO          ALMESSL--ASTGDDL-VALNGAIDKVNLSVKDQEGLQILMAHFYSFSESEDFRK
GCN1_DICDI          SLIESFT--NPNTISNAKEIKEAAVSICKSI-DEQGWDTLIGLLIEQTEIRLPNI
GCN1_MOUSE          AVMLALK--EKLGTPDEQLEMANCQAVILSVEDDTGHRIIIEDLLEATRSPEVGM
GCN1_HUMAN          AVMLALK--EKLGTPDEQLEMANCQAVILSVEDDTGHRIIIEYLLEATRSPEVGM
M7ANV9_CHEMY        AMMSALK--EKLGSADEQLEMANCQAVVLSVEDDVGQRIIIEDLLEATRSPEVGM
H8X4E7_CANO9        TLIASII--------AGEEISEEFNQVLLSVED-DGAHLLMQQLLALMKHEDPQI
W6A2P0_ICTPU        ALLSSLK--EKLGTEEGPQELSSCQTVILSVEDEVGQRIIIEDLLEATRGADAGL
B6JZI6_SCHJY        ALMESTF--SASEGDL-EGLTSATDSIMVSVQDPEGITQMMAYFTNLATNEDYRK
R4WJK2_RIPPE        ALLTALA--ASDGSP---QELEYCQAVVLSVNDEAGIRVIVDELLDASKSDKPEC
W8CCK9_CERCA        ALLHALS--EAQGTTHEYRELDYCQAVILSVTDEVGIRTIVDTLMVSAKSDHINT
B0W357_CULQU        ALMTALA--TAQGTPEEAQELEYCQAVILSVSDEVGVRTIMDTVMESTKSTNPET
E1ZX97_CAMFO        ALLTALS--SAQGTANELQELEYCQAVVLSVTDEVGVRTVMDQLMEATRAEDLSK
W5JJ28_ANODA        ALMSALA--AAQGTPEEVLELEYCQAVILSVSDEVGIRTIMDTVMESTKSDKAET
F1KPR4_ASCSU        SLLANCN---------DDEQVGQCLQVLLSVTDPEGVSTIVSTLLQKALTQD---
V9K7F0_CALMI        ALMTALK--DKMGTSEGQEELANCHTVILSVVDEVGQRIILEDLLEATKNPDVGM
W0T7Q3_KLUMA        SVVNTLI--ETDDEDTKHSLEGTLDKILLSVTDNEGLHPLLQQIMALLKHEDSSK
S9R951_SCHOY        ALMESIL--VAKDDVL-QSLEESVDTIVLSIKDQEGLAVLMAHLYSFTENEDYKK
S9W134_SCHCR        ALMESIL--VAKDDNL-QSLEEAVDTIVLSVKDQDGLTVLMAHLYSFTENEDYKK
H0GUK0_SACCK        TLVDAIT-TASNDESTKTALEVALNRIFLSVADDEGLHPLLQQIMSLLKNDNVEK
E5R2E4_ARTGP        TLMDEMI--SAEDSSLESEISEAFDTVLGSVDEFDGLNVAMNVMLTLIKHDDHRR
J8Q888_SACAR        ALVDAII-TTSNDESTKTALEVALDRVFLSVTDDEGLHPLLQQIMSLLKSDNKEK
C5G992_AJEDR        AFMDTIV--NTSDDELKKEVEEAFDTILESVDEYDGLNASMSVMLTLVKHEDHRK
G4MR95_MAGO7        SLMDNLV--NCKDDSLREDLDASFHTVISSIDEYDGLNTVMNVLLQLTKHEDHRK
C9SQ16_VERA1        SLMDNIV--NCKEDDLRVDLETSFDTVILSIDEHDGLNTVMNVLLQLTKHEDHRR
B2WNI9_PYRTR        TIMDNVI--ATKDEDLRAELETSFDKVLLSVDEYDGLNTAMSVMLALSKHDDERR
S3CN37_OPHP1        SLMDNII--NCDEDELREDLDTSFDTVILSIDEHDGLNTVMNVLLQLLRHEDHRK
G3ALT0_SPAPN        TLVNAVVDSNSEPEETQKEITNAFDKILLAIDDDEGVHPLMQQLMALVKHEDSRK
Q7S5P0_NEUCR        SLMDNII--NCTDDTLREELDESFDTVILSIDEYDGLNMVMQTLLQLLKHDDHRR
A0A165FVX9_9PEZI    SLLDNIV--DCQNEELRTELDGSFDTVLLSVDEYEGLNTTMSVMLGLVKHDDHRK
A0A178DN93_9PLEO    TIMDNVI--ASKDDELKSELEASFDKVLLSVDEYDGLNTAMSVMLALAKHDDERR
A0A177D7J2_ALTAL    TMMDNVI--ASKDEELKSELETAFDTVLLSVDEFDGLNTAMSVMLALSKHDDERR
A0A0C4DS68_MAGP6    SLMENLV--NCTDDDLRADLDASFHTVILSIDEYDGLNTIMNVLLQLTKHEDHRK
A0A151N4J5_ALLMI    AMMSALK--EKLGTSDEQLEMANCQVVILSVEDDAGQRIIIEDLLEATRSPEVGM
A0A146NKU7_FUNHE    ALLSSLK--GKLGTEEEAQELCSCQTVILSVEDEAGQRIIIEDLLEATRGADPGL
A0A0F7Z917_CROAD    AMMSALK--EKLGTNDEQQEMLNCQAVILSVEDEAGQRIIIEDLLDTTRSAEVGM
A0A0W0DC37_CANGB    SLVDTLI-SDDIDDTTKEGVKQALDRVFLSVKDDEGLHPLLQQILALVKNDDMKK
A0A0L8RIK7_SACEU    ALVDAII-TISGDESTKAALEVALDRVFLSVTDDEGLHPLLQQIMSLLKNDNIEK
A0A178FRD2_TRIVO    TLMDEII--STEDSGLKSEVSDAFDTVLDSVDEFDGLNVAMNVMLALMKHDDHRR
A0A175WD14_9PEZI    SLMDNIV--SCTEEELREDLDNSFDTVILSIDEYDGLNVVMNVLLQLIKHEDHRK
A0A194VFU1_9PEZI    SLMDNII--NCTDDELREDLDKSFDTVILSIDEYDGLNTTMQTLLQLMKHEDHRK
A0A151TXV6_CAJCA    PLLSAM---GDEDKEVQTLAKEAAETVVLVI-DEEGVEPLISELVKGINDSQAVV
A0A0M9ABE9_9HYME    GLLTALS--SAQGTPNEVQELEYCQTVILSVTDEVGIRTIMDQLMEATRADDLSR
A0A072U5F0_MEDTR    PLLSAM---SDVDQEVQTSAKKAAETVVLVI-DEEGVEPLISELLKGVSDSQAAI
A0A0K8V1F4_BACLA    ALLHALS--DAQGTAYEYQELDYCQAVILSVTDEIGVRTIVDTLMMSAKSDSINI
A0A0A1XE05_BACCU    ALLQALS--DAQGTAYEYQELDYCQAVILSVTDEIGVRTIVDTLMMSAKSDSINI
A0A131YTA4_RHIAP    ALLTAFS--ASLDTPKQQEELEHCQAVVLSVVDEAGVQTVVEQLLAGAR--QPGQ
A0A0J6I2R9_COCPO    TLIDNSL--STKNEKLRPEINSAFDTVLNSVDECDGLNAAMNVMITLMKHEDHHK
A0A074XMM5_AURPU    ALMDSVL--GTKDEELRADLENSFDAVLLSIDEFDGLNTAMSAMLALTKHEDHHR
A0A167FV98_9ASCO    ALVDALT------RDRDESISEALDTVVLSVVHEEGVHPLMQHLLSLAKNEQISK
A0A1A7XZM1_9TELE    ALLSSLK--GKLGTEEAAQELCSCQTVILSVEDEVGQRIIIEDLLEASRGADPGL
A0A0P7V1U6_9TELE    ALMSSLK--EKLGTEEGQQELSSCQTVILSVEDETGQRIIIEDLLEATRGADAGM
A0A1A7WWE1_9TELE    ALLSSLK--GKLGTEEAAQELCSCQTVILSVEDEVGQRIIIEDLLEASRGADPGL
                     .:                       :   : .  *    :  .
```

```
GCN1_YEAST            RIAVLERLPNFFDKTVLDFDVYIPNFVSHAILSLDDEDQRVVNGNFNALSTLL-K 2530
GCN1_SCHPO            RLFAAEHMLVFFQNCKLDYYRYVGDWVRHFITLFEDKSQDVVVAAVAAQNTLV-S
GCN1_DICDI            RLGACELIGEFYNGNTM-VTEYPEELLLSLLSLFNDPDALVQQAANNALGFIT-K
GCN1_MOUSE            RQAAAIILNMYCSRSKADYSSHLRSLVSGLIRLFNDSSPVVLEESWDALNAIT-K
GCN1_HUMAN            RQAAAIILNIYCSRSKADYTSHLRSLVSGLIRLFNDSSPVVLEESWDALNAIT-K
M7ANV9_CHEMY          RQAAAVILNIYCSKSKADCTAHLRNLVSGLIRLFNDTNPVVLNESWDALNSIT-K
H8X4E7_CANO9          REVIFAQTKDFFEHATLDYSMYLEDIVYQMILSLADPSPNVVKASMESLTVLI-K
W6A2P0_ICTPU          REASVTILNAYFSRTRLDYSTHTRTLLSGLVRLLNDPNPEVLHQSWDTINSIT-K
B6JZI6_SCHJY          RAFACSRMAAYFKDSKVELGKFYAEWVRVFIGLYEDRSEDVVKAALAAQTALV-G
R4WJK2_RIPPE          RRASATLLCAFCTHTRADLSQHVAQLLRGLILNFTDSDPVVLQMSWDALSAVT-K
W8CCK9_CERCA          RKSASSLLFVFCTHSPGDYSEYVPQLLRCLLCLMADSDRDILQRSWDALNSVV-K
B0W357_CULQU          RKAAATLLCAFCTHSPGDYSQYVPQLLRGLLRLLADSDRDVLQRSWDALNAVI-K
E1ZX97_CAMFO          RRSAATLLCAFCRDTRADYSQYVPQLLRGLIHLFTDEDRDVLQMSWEALTAVT-K
W5JJ28_ANODA          RKAAATLLCAFCTHSPGDYSQYVPQLFRGLLRLLADSDRDVLQRSWDALNAVT-K
F1KPR4_ASCSU          HVASSALIHLFAKNTKVDLSELVEEILPGALLLYNSSNNAIVENAIETLVCVT-K
V9K7F0_CALMI          RKAAATIINTYCSKSKADYFPHVRNLMAGLIRLFNDADETVLNESWDALSAVT-K
W0T7Q3_KLUMA          RIVMLERLPNFFDNTVLDYSIYTTDIVTNAILSLNESDSRIVEANFHALTSLV-K
S9R951_SCHOY          RLFAAKHMLTFFQNSKLDYFRFVPDWISHLVRLFEDRSSEVIAAAVAAQNALT-S
S9W134_SCHCR          RLFAAKHMLTFFQNSKLDYFRFVPDWISHLVRLFEDRSTDVIAAAVAAQHALT-S
H0GUK0_SACCK          RIAVLERLPNFFDKTTLDFDVYINDFVSHAILSLDDEDSRVVNGNFNALSTLL-K
E5R2E4_ARTGP          RSSAAMHLATFFTNTELDISRFYPELIRVLLISFDDRDKGVVKAAWEGLNQLT-K
J8Q888_SACAR          RIAVLERLPNFFDKTTLDFDIYIPDFVSHAILSLDDEDSRVVSGNFNALSTLL-K
C5G992_AJEDR          RANAATRLGRFFSHADVDISRYHPDLIRVLLISFDDHDRDVVKAAWEALTQLT-T
G4MR95_MAGO7          RAATGKQLARFFAATDVDYSRYNQDIIRSLLISFDDSDMEVVKSAWSALSEFT-K
C9SQ16_VERA1          RQATANHLAKFFAAGEVDYSRYNQDIIRSLLVSFDDGDIDVVKASWTALNEFT-R
B2WNI9_PYRTR          RARADMHLAKFFAECDVDFSRYYPELIRALLISFGDSDAEVVKAAWTALSTLTSK
S3CN37_OPHP1          RAATARHMATFFSQASVDYSRYYQDLIRALLISFDDRDTDVVKSSWAALTEFT-K
G3ALT0_SPAPN          RAAVFQRLGNFFTHTNLDYSAYLEDMVSQFILSLGDPASEVVEGAFESLSALV-K
Q7S5P0_NEUCR          RASTAFHLSKFFASADVDYSRYNQDIIRSLLLSFDDRDMDVVKSAWSALSEFT-K
A0A165FVX9_9PEZI      RASADLHLAKFFTETQVDYSRYYPDLIRVLLLSFDDHDMEVVKAAWTALSALT-K
A0A178DN93_9PLEO      RARADMHLATFFAESDVDFSRYYPDLIRALLISFDDGDKEVVKAAWTALSTLTTK
A0A177D7J2_ALTAL      RARADMHLAKFFADADVDFSRYYPDLIRALLISFGDSDTEVVKAAWTALSTLTSK
A0A0C4DS68_MAGP6      RAATDLQLAKFFAATDVDYSRYNQDIIRSLLVSFDDQDMGVVKAAWSALSEFT-K
A0A151N4J5_ALLMI      RQAAAVILNIYCSKTKADYTGHLRNLVSGLLRLFNDVNPVVLNESWDALSSIT-K
A0A146NKU7_FUNHE      RQAAITVLNAYFARTRLDYSAHIRTLLSGLIRLLNDSNPDVLSQSWDTINSIT-K
A0A0F7Z917_CROAD      RQAAAVILNIYCSKTKADYTAHLRNLVSGLIRLFNDTNSVVLNESWDALNAIS-K
A0A0W0DC37_CANGB      RIVTLQCLPNFFEQTSLDLDVYVPDFVSNSIMSLDDENAVFVKATFEALSALV-K
A0A0L8RIK7_SACEU      RIVILERLPNFFDKTTLDFGIYIPDFVSHAILSLDDEDPRVVKGNFDALSTLL-K
A0A178FRD2_TRIVO      RSSAAMHLATFFSNTEMDISRFYPELIRVLLISFDDRDKEVVKAAWEGLNQLT-K
A0A175WD14_9PEZI      RAATGQHLAKFFAAADVDYSRYNQDIIRALLISFDDRDPAVVKAAWSALSEFT-K
A0A194VFU1_9PEZI      RAATGRHLANFFAEADVDYSRYNQDIIRSLLISFDDRDTAVVKAAWSALSEFT-K
A0A151TXV6_CAJCA      RRSSSYLIGYFFKNSKLYLVDEASNMISTLIILLSDPDSSTVTLAWEALSRVI-T
A0A0M9ABE9_9HYME      RRSAATLLCAFCRDTRADYSQYVPQLLRGLIHLFTDNDKDVLQMSWEALTAVT-K
A0A072U5F0_MEDTR      RRSSSYLIGYFFKNSKLYLVDEAPNMISTLIVLLSDPDSSTVTVAWEALSRVI-M
A0A0K8V1F4_BACLA      RKSASSLLCVFCTHTPGDYSQYVPQLLRCLLRLMADSNRDILQNSWDALNSVV-K
A0A0A1XE05_BACCU      RKSASSLLCVFCTHSPADYSQYVPQLLRCLLRLMADSNREILQNSWDALSSVV-K
A0A131YTA4_RHIAP      RRAAVALLCAFCAHTRASLTPHVPQLLRELLRLFTDTDRHVLQLAGEALAAVT-K
A0A0J6I2R9_COCPO      RAAAANRLSSFFGKTTLDISRYYPELVRVCLISFDDYDTNVVAAAWEALSQLT-S
A0A074XMM5_AURPU      RARADMHLAKFFKTTDLDISRYYPDLIRTLLISFDDSDAEVVKAAWTALSALT-G
A0A167FV98_9ASCO      REITFRHMVLFFEQSTLDYSMYIQDWLSLLINALDERDENVVKAAWAALSVLV-K
A0A1A7XZM1_9TELE      RQAAVTILNAYFARTRLDYSAHTRTLLSGLIRLLNDSNPEVLSQSWDTINSIT-K
A0A0P7V1U6_9TELE      RQASVTILNAYFARTRLDYSAHTRALLSGLIRLLNDSNLEVLHQSWDTINSMT-K
A0A1A7WWE1_9TELE      RQAAVTILNAYFARTRLDYSAHTRTLLSGLIRLLNDSNPEVLSQSWDTINSIT-K
                      :         :               .   :    .               .
```

```
GCN1_YEAST          KVDKPTLEKLVKPAKQSLALTGRQGQD--------VAAFKLPR-GPNCVLPIFLH 2585
GCN1_SCHPO          ALRKDQLDSLVSIAYHSLRDVGSQGVN--------LPAFEVAQ-GVNSILPIFLY
GCN1_DICDI          SLKKDNL-TYLPVFQKGIQLLVNETYE---EVST-IPGFCLPK-GLASVLPVLIS
GCN1_MOUSE          KLDAGNQLALIEELHKEIRFIGNECK-----GEH-VPGFCLPKRGVTSILPVLRE
GCN1_HUMAN          KLDAGNQLALIEELHKEIRLIGNESK-----GEH-VPGFCLPKKGVTSILPVLRE
M7ANV9_CHEMY        KLDAGNQLALIEDLHRDIRMVGNEAK-----GEH-MPGFCIPKKGVTSILPVLRE
H8X4E7_CANO9        KQPKELLEKLVKPSYQALRLTGIS-----------VPAFALPK-GPNSILPVFLH
W6A2P0_ICTPU        KLDAGSQLALIDDLHRDIRSAAAEVR-----GEH-LPGFCLPKKGVTCILPVLRE
B6JZI6_SCHJY        SLRKDQMEPLVLPLCKALSDVGVPDTA--------LPAFQLPR-AINSVLPILLQ
R4WJK2_RIPPE        TLDSSQQISYVGDIRQAIKFAASDLKP----GEL-LPGLCLPK-GITPILPIFRE
W8CCK9_CERCA        SLDSAQQISHVSDVRQAVRFASSELK-----TSE-LPGFCLPK-GITPLLPVFRE
B0W357_CULQU        TLDSAQQIAHVTDVRQAVKFASSDLK-----GAE-LPGFCLPK-GITPLLPVFRE
E1ZX97_CAMFO        TLSSEQQIAHVQDIRQAVRFAVSDLK-----GQELLPGFCLPK-GITPILPIFRE
W5JJ28_ANODA        TLDSAQQIAHVTDVRQAVKFASSDLPK----GSE-LPGFCLPK-GITPLLPVFRE
F1KPR4_ASCSU        SLDQRQQIAAIGTVKQALASLQAHAN-----GGI-IAGMSHPK-GLQPLLPILRE
V9K7F0_CALMI        KLDSGGQLSLIDDLHKDIRAAAADAK-----GEH-LPGFCIPKKGVTSILPVLRE
W0T7Q3_KLUMA        NQDKPMLEKLIKPAKQALLMTGKQGED--------LAAFKLTK-GPSCVLPIFLH
S9R951_SCHOY        SMRKDRIESVISVTYKSLHDVGINDTT--------LPAFEIPQ-GINSVLPIFLH
S9W134_SCHCR        SMRKDRIESVINITYKSLHDVGVHDTT--------LPAFELPQ-GISSILPIFLH
H0GUK0_SACCK        KVDKPTLEKLVKPAKQSLALTGKQGED--------LAAFRLPK-GPNCVLPIFLH
E5R2E4_ARTGP        SMKKEEMEVLVNPTRQVLRQVGVPGSN--------LAGFSLPK-GIGAILPIFLQ
J8Q888_SACAR        KVDKSTLEKLVKPSKQSLALTGKQGED--------LAAFKLPR-GPNCILPVFLH
C5G992_AJEDR        HIRKEEMEVLVIPTRQVLRQVGVPGSD--------LPGFCLPK-GIGAIFPIFLQ
G4MR95_MAGO7        KLRKEEMEALVVSTRQTLLQVGVAGNN--------LKGFELPK-GVSAILPIFLQ
C9SQ16_VERA1        KLKKEEMEALVFSTRQALQHVGVAGAS--------LKGFELPK-GINAILPIFLQ
B2WNI9_PYRTR        RLRKEEMESLVISTRQTLNQVGVAGAD--------LPGFSLPK-GINAVLPIFLQ
S3CN37_OPHP1        KLKKEEMEALVASTRQTLLMVGVAGTN--------LPGFELPK-GINAILPIFLQ
G3ALT0_SPAPN        RQPKESLEHLVKPARQALDFTGVRGED--------LAGFKLPK-GPNCILPIFLH
Q7S5P0_NEUCR        KLRKEDMENLVISTRQTLLQVGVAGVN--------LRGFELPK-GINAILPIFLQ
A0A165FVX9_9PEZI    KLRKEEMESLVFSSRQTLQQVGVPGAN--------LTGFSLPK-GINAILPIFLQ
A0A178DN93_9PLEO    RLRKEEMESLVISTRQTLNQVGVAGTD--------LPGFSLPK-GINAILPIFLQ
A0A177D7J2_ALTAL    RLRKEEMESLVISTRQTLNQVGVAGAD--------LPGFSLPK-GINAILPIFLQ
A0A0C4DS68_MAGP6    KLKKEEMEALVSSTRQTLLLVGVAGSN--------LRGFELPK-GINAILPIFLQ
A0A151N4J5_ALLMI    KLDAGNQLALIEDLHRDIRVVGNEAK-----GEH-VPGFCIPKKGVTSILPVLRE
A0A146NKU7_FUNHE    KLDASSQLALIDDLHRDIKAIAADVK-----GQH-LPGFCLPKKGVTCILSVLRE
A0A0F7Z917_CROAD    KLDAGSQLALIEDLHRDIRIVGNDAA-----GEH-VPGFCIPKKGVTSILPMLRE
A0A0W0DC37_CANGB    KQPKDMLEKLVQPAKQALQRTGKQGED--------LSAFALPR-GPNCVLPIFLH
A0A0L8RIK7_SACEU    KVDKPTLEKLVKPSKQSLALTGKQGED--------LAAFVLPR-GPNCILPIFLH
A0A178FRD2_TRIVO    SMKKEEMEVLVNPARQVLRQVGVPGSN--------LAGFSLPK-GIGAILPIFLQ
A0A175WD14_9PEZI    RLKKEEMEALVQSTRQTLLQVGVAGHN--------LAGFELPK-GINAILPIFLQ
A0A194VFU1_9PEZI    HLKKEEMEALVFSTRQNLLQVGVAGAN--------LPGFELPK-GINAILPIFLQ
A0A151TXV6_CAJCA    SVPKELLPSYIKLVRDAVSTSRDKERRKKKGGPILIPGFCLPK-ALQPLLPIFLQ
A0A0M9ABE9_9HYME    TLASDQQIAHVQDIRQAVRFAVSDLK-----GQELLPGFCLPK-GITPILPIFRE
A0A072U5F0_MEDTR    SVPKEVLPSYIKLVRDAVSSSRDKERRKKKGGPVLIPGFCLPK-SLQPILPIFLQ
A0A0K8V1F4_BACLA    TLDSTQQISLVCDVRQAVRFASSEIR-----GAE-MPGFCLPK-GITPLLPVFRE
A0A0A1XE05_BACCU    ALDSTQQIALVSDVRQAVRFASSEMK-----GSE-LPGFCLPK-GITPLLPVFRE
A0A131YTA4_RHIAP    TLDTNQQIEYVTDVRQAIRFAASDLK-----GQEYLPGFCQEK-GISPILPIFRE
A0A0J6I2R9_COCPO    HMRKEEMEVLVIPTRQVLRQVGVAGAN--------LPGFCRPK-GISAVFPIFLQ
A0A074XMM5_AURPU    RLRKEEMESLVFSTRQTLNQVGVAGHS--------LPGFSLPK-GINAILPIFLQ
A0A167FV98_9ASCO    KLSKEDMEKLVRPTRQALVLTGSPGSD--------LPGFALPK-GPSCILPIFIQ
A0A1A7XZM1_9TELE    KLDASSQLALIDDLHRDIRSVAADVK-----GQH-LPGFCLPKKGVTCILSVLRE
A0A0P7V1U6_9TELE    KLDAGSQLALIDDLHRDIRAAGAEAK-----GQH-LPGFCLPKKGVTCILPVLRE
A0A1A7WWE1_9TELE    KLDASSQLALIDDLHRDIRSVAADVK-----GQH-LPGFCLPKKGVTCILSVLRE
                              :      :                 : .:   : .   ::.::
```

```
GCN1_YEAST          GLMYGSNDEREESALAIADVVSKTPAANLKPFVSVITGPLIRVVGERFSSDIKAA 2640
GCN1_SCHPO          GLMHGTMDQREQSALGIADIVLKTEPSKLRPFVTQITGPLIRIIGERFPVEVKCA
GCN1_DICDI          GLMYGTSDQREQATNTLRTVINHTSADALKPFVMQITGPLILVIGDKFPWQVKSA
GCN1_MOUSE          GVLTGSPEQKEEAAKGLGLVIRLTSADALRPSVVSITGPLIRILGDRFNWTVKAA
GCN1_HUMAN          GVLTGSPEQKEEAAKALGLVIRLTSADALRPSVVSITGPLIRILGDRFSWNVKAA
M7ANV9_CHEMY        GVLTGNPEQKEEAAKALGLVIKLTSAEALKPSVVSITGPLIRILGDRFSWNVKVA
H8X4E7_CANO9        GLMYGSSDQRELSALAIADIIEKTPAENLRTLATSLTGPLIRVIGEKVASNIKSA
W6A2P0_ICTPU        GVLTGTPEQKEEAARALGAVIKLTSAEALRPSVINITGPLIRILGDRFAWTVKTA
B6JZI6_SCHJY        GLMYGSTEQREKSALGIADIVRRTEPTALRPSVTQITGPLIRIIGERFPTDVKSA
R4WJK2_RIPPE        SILNGSPETKEAAAQGLGEVIALTTAQSLQPSVIHITGPLIRILGDRFPPSVKAA
W8CCK9_CERCA        AILNGMPDVKENAAQGLGEIISLTSAQSLQSSVVQITGPLIRILGDRFNAGVKAA
B0W357_CULQU        AILNGLPEEKENAAQGLGEVIQLTSPASLQPSVVHITGPLIRILGDRFNAGVKAA
E1ZX97_CAMFO        AILNGLPEAKEHAAQGLGEVIRLSSADALQPSVVHITGPLIRILGDRFNWSVKAA
W5JJ28_ANODA        AILNGLPEEKENAAQGLGEVIKLTSPTSLQPSVVHITGPLIRILGDRFNAGVKAS
F1KPR4_ASCSU        GILSGGVEMKEVAGETLGTIVSMSSAAALKAHVVNVTGPLIRVLGDRYPPPVKLS
V9K7F0_CALMI        GVLTGSPEQKEEAAKALGLVIKLTSAEALKPSVINITGPLIRILGDRFSWNVKVA
W0T7Q3_KLUMA        GLMYGSGDEREASALAIADIVSKTPAAGLKSYVTVITGPLIRVVGERFNSDIKAA
S9R951_SCHOY        GIMHGTNEQREQSALGVADIVTRAEADKLRPYVTQITGPLIRIIGERFPVEVKSA
S9W134_SCHCR        GLMHGTNEQREQSALGVADIVMRAEPDKLRPYVTQITGPLIRISGERFPVEVKSA
H0GUK0_SACCK        GLMYGSNDEREESALAIADVVSKTPAANLKPFVSVITGPLIRVIGERFSSDIKAA
E5R2E4_ARTGP        GLLNGNIEQRTQSALAIGDIIDRTSPESLKAFVTQITGPLIRVVSER-SVDIKCA
J8Q888_SACAR        GLMYGSNDEREESALAIADVVSKTPAVNLKPFVSVITGPLIRVVGERFSSDIKAA
C5G992_AJEDR        GLLNGNVDQRVQSALAIADIIDRTSAEALRPYVTQITGPLIRVVSER-SVEIKCA
G4MR95_MAGO7        GLMNGTPDQKIQSALAISDIVARTSEASLKPFVVQITGPLIRVVSER-STEVKSA
C9SQ16_VERA1        GLMNGTAEQRTQAALAISDIVDRASETALKPFVTQITGPLIRVVSER-STEVKAA
B2WNI9_PYRTR        GLMNGTIDQRTQAALAISDVIDRTSAKSLQPFVTQITGPLIRVVTER-SVEVKAA
S3CN37_OPHP1        GLMNGTADQRTIAALAISDIVDRTSEAALKPFVTQITGPLIRVVTER-STDVKCA
G3ALT0_SPAPN        GLMYGNSDQKEASAFAIADIINKTPDVNLKPFATTMTGPLIRVIGEKVNSDIKAA
Q7S5P0_NEUCR        GLMNGSADQRVASALAISDIVDRTSEASLKPFVTQITGPLIRVVSER-STEVKSA
A0A165FVX9_9PEZI    GLMNGTTEQRVQSALAISDLVARTSEDSLKPFVTQITGPLIRVVSER-SVDVKSA
A0A178DN93_9PLEO    GLMNGSVEQRTQAALAISDVIDRTSAKTLQPFVTQITGPLIRVVTER-STEVKAA
A0A177D7J2_ALTAL    GLMNGSVDQRVQGALAISDVIDRTSAKSLQPFVTQITGPLIRVVTER-SVEVKAA
A0A0C4DS68_MAGP6    GLMNGSPDQKTQAALAISDIVDRTSEASLKPFVTQITGPLIRVVSER-STEVKSA
A0A151N4J5_ALLMI    GVLTGNPDQKEEAAKALGLVIKLTSAEALKPSVVSITGPLIRILGDRFSWNVKVA
A0A146NKU7_FUNHE    GVLTGSPEQKEEAAKALGGVIKLTSPEALRPSVVNITGPLIRILGDRFAWTVKTA
A0A0F7Z917_CROAD    GVLTGNSDQKEEAAKALGLVIKLTSAEALKPSVVSITGPLIRILGDRFSWNVKVA
A0A0W0DC37_CANGB    GLMYGSAEEREVSALAIADVVSKTPADNLKPFVSAITGPLIRVVGERFKSDVKAA
A0A0L8RIK7_SACEU    GLMYGSNDEREESALAIADVVSKTPAANLKPFVSVITGPLIRVVGERFSSDIKAA
A0A178FRD2_TRIVO    GLLNGNIEQRTQSALAIGDIIDRTSPESLKTFVTQITGPLIRVVSER-SVDIKCA
A0A175WD14_9PEZI    GLMNGTPDQRVSAALAISDIVDRTSENSLKPFVTQITGPLIRVVSER-STEVKSA
A0A194VFU1_9PEZI    GLMNGTPEQRTTSALAISDIVDRTSEASLKPFVTQITGPLIRVVSER-STDVKSA
A0A151TXV6_CAJCA    GLISGSAELREQAALGLGELIEVTSEQSLKEFVIPITGPLIRIIGDRFPWQVKSA
A0A0M9ABE9_9HYME    AILNGLPEAKEQAAQGLGEVIKLTNASALQPSVVHITGPLIRILGDRFNWSVKAA
A0A072U5F0_MEDTR    GLISGSAELREQAALGLGELIEVAGEQSLKEVVIPITGPLIRIIGDRFPWQVKSA
A0A0K8V1F4_BACLA    AILNGMPDVKENAAEGLGEIISLTSAQSLQPSVVQITGPLIRILGDRFNAGVKAA
A0A0A1XE05_BACCU    AILNGMPDVKENAAEGLGEVISLTSAQSLQPSVVQITGPLIRILGDRFNAGVKAA
A0A131YTA4_RHIAP    AILIGVPELKEQAAQGLGEVIRLTDAASLRQSVISITGPLIRILGDRFSFSVKVA
A0A0J6I2R9_COCPO    GLLNGTVEQRVQSALAIGDIIDRTSTEALKPFVTQITGPLIRVVSER-SVEIKCA
A0A074XMM5_AURPU    GLMNGSAEQRTAAALAISDIIDRTSADALKPFVTQITGPLIRVVSER-SVEVKAA
A0A167FV98_9ASCO    GLMYGTSDQREQAALGIADIVERTSPDSLKPFVTQITGPLIRTIGERFPSDVKAA
A0A1A7XZM1_9TELE    GVLTGSPEQKEEAAKALGGVIRLTSSEALRPSVINITGPLIRILGDRFAWTVKTA
A0A0P7V1U6_9TELE    GVLTGSPEQKEEAAKALGVVIKLTSPEALKPSVVNITGPLIRILGDRFSWTVKTA
A0A1A7WWE1_9TELE    GVLTGSPEQKEEAAKALGGVIRLTSSEALRPSVINITGPLIRILGDRFAWTVKTA
                    .:: *  : :   .    :  ::  :     *:  .  :*****    ::    :* :
```

```
GCN1_YEAST              ILFALNVLFIKIPMFLRPFIPQLQRTFVKSLSDATNETLRLRAAKALGALIEHQP 2695
GCN1_SCHPO              ILYTLNIILSKISTFLRPFLPQLQRTFAKCLGDPSSEVIRSRAATALGTLITLQT
GCN1_DICDI              ILQTLSLLISKSPASMKIFLHQLQPTFIKCLSD-SHKNVRTNAASALGLLMTLSS
GCN1_MOUSE              LLETLSLLLGKVGIALKPFLPQLQTTFTKALQD-SNRGVRLKAADALGKLISIHV
GCN1_HUMAN              LLETLSLLLAKVGIALKPFLPQLQTTFTKALQD-SNRGVRLKAADALGKLISIHI
M7ANV9_CHEMY            LLETLSLLLAKVGIALKPFLPQLQTTFTKALQD-SNRAVRLKAADALGKLIAIHI
H8X4E7_CANO9            ILVALNNLLIKIPQFLRPFIPQLQRTFVRSLSDVSNDTLRKRAVVALSTLIKHQP
W6A2P0_ICTPU            LLETLTLLLRKVGIALKPFLPQLQTTFLKALQD-TSRPVRLKAAEALGQLVSIHT
B6JZI6_SCHJY            ILFTLNILLTKIPTFLRPFLPQLQRTFAKCLADPSSDVVRNRAAAALGTLITLQT
R4WJK2_RIPPE            VLHTLAGLLAKVGMMLKQFLPQLQTTFLKALND-GNRMVRLKAASALSYLITIHT
W8CCK9_CERCA            VLESLAILLRKVGVMLKQFLPQLQTTFLKALHD-SNRTVRMKAGKAISELVIIHS
B0W357_CULQU            VLETLAILLHKVGIMLKQFLPQLQTTFLKALHD-PNRIVRIKAGHALAELIVIHT
E1ZX97_CAMFO            VLETLAILLGKVGVMLKQFLPQLQTTFLRALND-SNRQVRLKAAYALSNLIVIHT
W5JJ28_ANODA            VLETLAILLHKVGIMLKQFLPQLQTTFLKALHD-PSRVVRIKAGHALAELILIHT
F1KPR4_ASCSU            ILWTLAQLLDKVDVLLRPFLPQLQSTFLKALQEPSSRKVRLYSGGALSRLITIHP
V9K7F0_CALMI            LLDTLSLLLGKVGIALKPFLPQLQTTFTKALSD-ANRAVRLKAAEALGNLIVIHT
W0T7Q3_KLUMA            ILFALNILFAKIPQFLRPFIPQLQRTFVKSLSDPTNEVLRLRAAKALGTLIEYQP
S9R951_SCHOY            ILYTLNIILSKIPTFLRPFLPQLQRTFAKSLGDPSSEVIRSRAATALGTLITLQT
S9W134_SCHCR            ILYTLNIILSKIPTFLRPFLPQLQRTFAKSLGDPSSEVIRSRAATALGTLITLQT
H0GUK0_SACCK            ILFALNVLFIKIPMFLRPFIPQLQRTFVKSLSDATNETLRLRAARALGALIEHQP
E5R2E4_ARTGP            IFLALDKLLEKIPLFVKPFLPQLQRTFARGLADTSSETLRTRAAKGLSILITLTP
J8Q888_SACAR            ILFALNVLFIKIPMFLRPFIPQLQRTFVKSLSDATNETLRLRAAKALGALIEHQP
C5G992_AJEDR            VFLALNKLLEKIPLFVKPFLPQLQRTFARGLADTSSDVLRKRASKGLGILITLTP
G4MR95_MAGO7            ILLTLNNLLEKMPTALKPFLPQLQRTFAKSLADTTSEQLRSRAAKALGTLIKYTP
C9SQ16_VERA1            ILLTLNNLLEKMPIALKPFLPQLQRTFAKSLADTTSELLRARAAKALGTLIKYTP
B2WNI9_PYRTR            ILLTLNNLLEKIPTFLKPFLPQLQRTFAKSLADTSSDVLRARAAKALGTLIKLTP
S3CN37_OPHP1            ILLTLNNLLEKMPMALKPFLPQLQRTFTKSLADTTSELLRSRAAKALGAVIKYTP
G3ALT0_SPAPN            ILVALNSLLLKIPQFLRPFIPQLQRTFVRSLSDASNEKLRARAVVSLGSLIKFQP
Q7S5P0_NEUCR            ILLTLNNLLEKMPTALKPFLPQLQRTFAKSLADTSSDVLRSRAAKALGTLIKFTP
A0A165FVX9_9PEZI        ILFTLNNLLEKIPTFLKPFLPQLQRTFAKSLADASSEDLRNRAAKALGTLITLTP
A0A178DN93_9PLEO        ILLTLNNLLEKIPTFLKPFLPQLQRTFAKSLADTSSDILRARAAKALGTLIKLTP
A0A177D7J2_ALTAL        ILLTLNNLLEKIPTFLKPFLPQLQRTFAKSLADTSSDILRARAAKALGTLIKLTP
A0A0C4DS68_MAGP6        ILLTLNNLLEKMPMALKPFLPQLQRTFAKSLADTTSEQLRTRAAKALGTLIKFTP
A0A151N4J5_ALLMI        LLETLSLLLAKVGIALKPFLPQLQTTFIKALQD-SNRTVRLKAADALGKLIVIHV
A0A146NKU7_FUNHE        LLETLTLLLAKVGIALKPFLPQLQTTFLKALQD-SSRAVRLRAAEALGQLVAIHA
A0A0F7Z917_CROAD        LLETLSLLLAKVGIALKPFLPQLQTTFTKALQD-PNRVVRLKAADALGKLIAIHA
A0A0W0DC37_CANGB        ILLALNILFKKIPQFLRPFIPQLQRTFVKSLSDPTNETLRLRAAKAIGTLIEFQP
A0A0L8RIK7_SACEU        ILFALNVLFVKIPMFLRPFIPQLQRTFVKSLSDASNETLRLRAAKALGALIEHQP
A0A178FRD2_TRIVO        IFLALDKLLEKIPLFVKPFLPQLQRTFARGLADTSSETLRTRAAKGLGILITLTP
A0A175WD14_9PEZI        ILLTLNNLLEKMPTALKPFLPQLQRTFAKSLADTSSDLLRGRAARALGTLIKFTP
A0A194VFU1_9PEZI        ILLALNNLLEKMPTALKPFLPQLQRTFAKSLADTTSELLRTRAAKALGTLIKFTP
A0A151TXV6_CAJCA        ILSTLTMMIKKGGISLKPFLPQLQTTFVKCLQD-STRTVRTSAALALGKLSGLST
A0A0M9ABE9_9HYME        VLETLAILLGKVGVMLKQFLPQLQTTFLKALND-SNRQVRLKAAYAISNLIVIHT
A0A072U5F0_MEDTR        ILSTLTIMIRKGGISLKPFLPQLQTTFVKCLQD-NTRTIRSGAAVALGMLSGLNT
A0A0K8V1F4_BACLA        VLETLAILLRKVGVMLKQFLPQLQTTFLKALHD-SNRSVRMKAGQAISELGIIHS
A0A0A1XE05_BACCU        VLETLAILLKKVGVMLKQFLPQLQTTFLKALHD-SNRSVRMKAGQAISELVIIHS
A0A131YTA4_RHIAP        VLETLALLLAKVGVQLKPFLPQLQTTFLKALND-GNRQVRLKASVALSHLIVIHT
A0A0J6I2R9_COCPO        VFLAINKLLEKIPLFIKPFLPQLQRIFARGLADSSSETLRSRAAKGLGILITLTP
A0A074XMM5_AURPU        ILFTLNNLLEKIPAFLKPFLPQLQRTFAKSLADTSSEILRIRAAKALGTLITLTP
A0A167FV98_9ASCO        ILYTLSLLLTKIPAFLKPFLPQLQRTFAKSLSDPTSELLRSRAAKALGILITLQA
A0A1A7XZM1_9TELE        LLETLTLLLAKVGIALKPFLPQLQTTFLKALQD-SSRAVRLRAAEALGQLVSIHS
A0A0P7V1U6_9TELE        LLETLTLLLAKVGIALKPFLPQLQTTFLKALQD-SSRAVRLRAAEALGQLVTIHS
A0A1A7WWE1_9TELE        LLETLTLLLAKVGIALKPFLPQLQTTFLKALQD-SSRAVRLRAAEALGQLVSIHS
                        :: ::  :: *    :: *: ***  * : * :     :*  :  .:. :
```

```
GCN1_YEAST              RVDPLVIELVTGAKQATDEGVKTAMLKALLEVIMKAGSKLNENSKTNIVNLVEEE 2750
GCN1_SCHPO              RLAPIITELVSGAR-TPDAGVRKAMLNALFAVVSKSGQNMNEASAEAIEQLLDEI
GCN1_DICDI              SVDQLVNSLITGIS-TADSISQESKLRALQSIFEKKPKVEQATLDKAIATIVDFL
GCN1_MOUSE              KVDPLFTELLNGIRAVEDPGIRDTMLQALRFVIQGAGSKVDAAIRKNLVSLLLSM
GCN1_HUMAN              KVDPLFTELLNGIRAMEDPGVRDTMLQALRFVIQGAGAKVDAVIRKNIVSLLLSM
M7ANV9_CHEMY            KVDPLFTELLNGIRSSGDSSIRDTMLQALRFVTQGAGAKVDATVRKTITTALLGM
H8X4E7_CANO9            RVDSLITELVTNSKAIDDSGVKASMLQGMLAVVELKGKELNEASKTSLLSVVEEE
W6A2P0_ICTPU            KVDPLFTEQLSAIRNAEDSGVRETMLQALRFVIQGAGAKVDPAIKKSITTTLLGM
B6JZI6_SCHJY            RVDPLITELVSGSR-SSDAGVRKAMFKALFEVVSKSGKNMSENSMNSVGDLLEET
R4WJK2_RIPPE            RPDPLFSELHNSVKSAEEPAVRETMMQALRGVISAAGDKMSDAVVKQIHTSLLGL
W8CCK9_CERCA            RPDPIFIEIHNGIKSSEDLPIRETMLAAVRSSIHSAGEKMSESLKKQISATLLSI
B0W357_CULQU            RPDPLFVEMHNGIKNADDSTVRETMLQALRGIITPAGDKMTDPLKKQIYATLSGM
E1ZX97_CAMFO            RVDPLFTELHTGIKTGDDPAIRETMLQALRGVLTPAGDKMTDPMKKQVFATLSSM
W5JJ28_ANODA            RPDPLFIEMHNGVRNADDATVRETMLQALRGIMTPAGDKMTEPLRKQIYATLAGM
F1KPR4_ASCSU            KPEPIAAELVKLLVSSDDSSLLETTLVSLRAIVNRVHAKLSDECLKSILSVAEKH
V9K7F0_CALMI            KVDPLFTELLNLIRNAEDSSVRETLLQALRFVTQGAGAKVDGTIRKNITTMLLGM
W0T7Q3_KLUMA            RVDPLIVELVTGAKQSTSDGVKTAMLKALLETVSKAGSKLNQSSKSNILNLIEEE
S9R951_SCHOY            RLAPIVTELVSGAR-TPDAGVRKAMLNALFAVVSKSGQNMNEASADSVDQLLKEI
S9W134_SCHCR            RLAPIVTELVAGAR-TPDAGVRKAMLNALFAVVSKSGQNMNEASAESVDQLLREI
H0GUK0_SACCK            RVDPLVIELVTGAKQATDEGVKTAMLKALLEVIVKAGSKLNEGSKTNIVNLVEEE
E5R2E4_ARTGP            RVDPLVAELITGSK-TTDPGVKNAMLRALHDVVDKAGANMSEASRQAVLGLVDND
J8Q888_SACAR            RVDPLVVELVTGAKQATDEGVKTAMLKALLEVIVKAGSKLNENSKSIIVNLVEEE
C5G992_AJEDR            RVDPLIAELVAGSK-TSDSGVRNAMLRALYEVVSKAGKNMSDTSRQTILTLIDDE
G4MR95_MAGO7            RVDPLIAELVTGSK-TTDPGVRTAMLKALFEVISKAGANMGEPSRAAVLGLIDME
C9SQ16_VERA1            RIDPLIAELVTGSK-TSDAGVKTAMLSALYEVISKAGANMGESSRAAVLGLIDME
B2WNI9_PYRTR            RVDPLIAELVTGSK-TSDEAVKTAMLKALFEVVSKAGKNMSEASRNSILGLIDNE
S3CN37_OPHP1            RIDPLIAELVTGSK-TTDTGVKNAMLKALFEVISKAGANMGEPSRAAVLSLIDMD
G3ALT0_SPAPN            RVDSLITELVTGAKNSNDKGVKASMLKGMLEVVNKAGKSLSEDSKTSIMSLIEDE
Q7S5P0_NEUCR            RVDPLIAELVTGSK-TSDAGVKTAMLKALYEVISKAGANMGEGSRTAVLGLIDTE
A0A165FVX9_9PEZI        RIDPLIAELVAGSK-TPDPGVKNAMLKALYEVVSKAGANMSEASRNAIFGLIDTE
A0A178DN93_9PLEO        RVDPLIAELVTGSK-TSDEAVKNAMLKALFEVVSKAGKNMNEASRNAILSLIDTE
A0A177D7J2_ALTAL        RVDPLIAELVTGSK-TADENVRTAMLKALFEVVSKAGKNMSEASRNAILGLIDNE
A0A0C4DS68_MAGP6        RVDPLIAELVTGSK-TSDPGVKTAMLKALFEVISKAGANMGEPSRAAVLGLIDME
A0A151N4J5_ALLMI        KVDPLFTELLNGIRSSEDSGIRDTMLQALRFVTQGAGAKVDAAIRKNISTVLLGM
A0A146NKU7_FUNHE        KVDPLFTEQLSAIRNAEDSGVRETMLQALRFVIQGAGSKVDPAIRKSITTTLLGM
A0A0F7Z917_CROAD        KVDPLFTELLNGIRTSEDSSIRDTMLQALRFVTQGAGAKVDAAVRKSIITVLLSM
A0A0W0DC37_CANGB        RVDPLVIELVTSAKQTEEEGVKTAMLNALLEVVGKAGSKLNEASKKNIVKLVEEE
A0A0L8RIK7_SACEU        RVDPLVIELVTGAKQATDEGVKTAMLKALLEVIIKAGSKLNESSKIHIVNLVEEE
A0A178FRD2_TRIVO        RVDPLVAELITGSK-TTDPGVKNAMLRALHDVVDKAGTNMSEASRQAVLGLVDND
A0A175WD14_9PEZI        RVDPLIAELVTGSK-TTDAGVRTAMLKALYEVISKAGANMGESSRAAVLGLIDME
A0A194VFU1_9PEZI        RVDPLIAELVTGSK-TSDAGVKTAMFKALYEVISKAGANMSEASRTAVLGLIDME
A0A151TXV6_CAJCA        RVDPLVSDLLSSLQ-GSDGGVREAILTALKGVLKHAGKSVSSAVRNRFYGVLKDL
A0A0M9ABE9_9HYME        RVDPLFTDLHTGIKTGDDPAIRETMLQALRGVLTPAGDRITEPMKKQVFATLSSM
A0A072U5F0_MEDTR        RVDPLVSDLLSSLQ-GSDGGVREAILSALKGVLKHAGKNVSSAVSSRIYSVLKDL
A0A0K8V1F4_BACLA        RPDSIFNEIHNGIKSNDDPSVRETMLAAIRLSINLVGGKMSESLKQQLSATLLNM
A0A0A1XE05_BACCU        RPDSIFNEIHNGIKCNDDPSMRETMLAAIRLSINLAGEKMSVCLKQQICTTLLNM
A0A131YTA4_RHIAP        RCDPVFQELHNSVKNQDDPTVRETMLYALHRVVAAAGHKMSDLMRRSVTASVSSY
A0A0J6I2R9_COCPO        RVDPLISELVAGSK-TSDSGVKSAMLRALHEVVAKAGKNMSDASKQAILELIDDE
A0A074XMM5_AURPU        RIDPLIAELITGSK-TADAGVRNAMLKALYDVVVKCGSSMNEASRTGILSLIDSD
A0A167FV98_9ASCO        RIDPLVNELISGARISTDSGVTEAMLQALYEVVSSVGKNLSDASKNSVIAFIEQI
A0A1A7XZM1_9TELE        KVDPLFTEQLSAIRNAEDSGVRETMLQALRFVIQGAGSKVDPAIRKNITTTLLGM
A0A0P7V1U6_9TELE        KVDPLFSEQLSAIRNAEDSGIRETMLQALRFVIQGAGSKVDSAIRKNITTTLLGM
A0A1A7WWE1_9TELE        KVDPLFTEQLSAIRNAEDSGVRETMLQALRFVIQGAGSKVDPAIRKNITTTLLGM
                            :  .          .      : : .:                    .
```

```
GCN1_YEAST            MLGS-NDKLAVAY--AKLIGSLSEILSN-DEAHKILQDKVLN--ADLDGETGKFA 2805
GCN1_SCHPO            SAES-SEHMVIC---AKLYGALFSHLPD-AQAKQLLESKVLS-----LEIQSEFS
GCN1_DICDI            YQPS-DDLRSMV---AQTIGASSKCFTSLTELNQFIKTNLIS--PSQSVLSRYGK
GCN1_MOUSE            LGHD-EDNTRIST--AGCLGELCAFLTD-EELNTVLQQCLLADVSGIDWMVRHGR
GCN1_HUMAN            LGHD-EDNTRISS--AGCLGELCAFLTE-EELSAVLQQCLLADVSGIDWMVRHGR
M7ANV9_CHEMY          LGHD-EDATRMAS--AGCLAELCAFLSE-EELSTVLQQHLLADVSGIDWMVRHGQ
H8X4E7_CANO9          SIDS------VSS--AKLLGSLANVLTP-EETSVLLQKKVIN-------SQSKFS
W6A2P0_ICTPU          LGHD-EDATRMAS--AGCVGMLCAFLSE-EELRTVLQQHVLADVSGVDWMVRHGR
B6JZI6_SCHJY          EASD-MTDVVNM---AKLYGAWFANLPD-ARASEFLEDKLFS-----VEEDTTLR
R4WJK2_RIPPE          LGHA-EDTTRTGA--AGCLGALTRSLTV-DQLTVTLNDHLLQDDLTSDWTLRHGR
W8CCK9_CERCA          IGHS-DDVTRSAA--GGCLGALLKHLSV-QQVNDILEHHILLDDND-DALLTHGR
B0W357_CULQU          LGHS-EDITRTAA--AGCFGALCRWLNP-DQLDDALNTHLLNEDYGEDAALRHGR
E1ZX97_CAMFO          LSHP-EDVTRNAV--AGCFGALLRWLNP-EQLAIALNDHLLCNDVNVDWMLRHGR
W5JJ28_ANODA          LGHS-EDVTRAAA--AGCFGALVRWLPS-DLLDDALASHLLNEDYGDDATLRHGR
F1KPR4_ASCSU          CGEDADDATLLAS--AALYGEALLRLNA---FTPAFLTPVEN--NGCDVRQRHAH
V9K7F0_CALMI          LGHD-EDATRMAS--AGCLGELCAFISD-DELNTILQQHLLADVSGIDWMVRHGR
W0T7Q3_KLUMA          MLSA-NDKLAVAY--AKLIGSLASILTT-EEAEKILRSKVLE--SSLTEDSGKFG
S9R951_SCHOY          SSES-NEYMIIC---AKMYGALFNQLTD-TRARQLLDTNIFS-----VEALDEYS
S9W134_SCHCR          SSET-NEYMIIC---AKMYGALFNQLTD-TRARQLLDTKIFS-----VETLDEYS
H0GUK0_SACCK          MLGS-NDKLAVAY--AKLIGSLSEILSH-DEAHKILQDRVLN--ADLDGETGMFA
E5R2E4_ARTGP          SVD--DAATTITN--AKLAGVLIKSLPT-PTAIPLIKNRILS------PQLTHQS
J8Q888_SACAR          MLGS-NDKLAVAY--AKLIGSLSEILSN-EEAHKILQDKVLN--ADLDGETGKFA
C5G992_AJEDR          SNGR-DDTMNITN--ARLLGALVKTLPA-TTVVPLIKSRVLS------PHLTHSS
G4MR95_MAGO7          TDEK-DDAMTVTN--AKLLGALVKNVSG-DAAHNLIKSRVLT------PTPTTSS
C9SQ16_VERA1          GDEK-DSAMTITS--AKLLGALIKNVPE-DAAHGLLRNRVIT------NQISKSS
B2WNI9_PYRTR          TDDS-NDAMAITN--ARLLGALISCLPE-DVASSLLKARVLT------THFSKAS
S3CN37_OPHP1          SDVH-DDEMAITN--AKLLGALVKNVNP-EAALGLLKNRVLA------PTANNAS
G3ALT0_SPAPN          ITVV-DDKSAVSY--ARLLGSLAGILSI-DEARNILKSKILE---KVDNSNDKFC
Q7S5P0_NEUCR          ADER-DDTMTITY--AKLFGALVKNVSD-EVAAQLLKNRVLT------RDFSNSS
A0A165FVX9_9PEZI      IDES-DDAMAITN--ARLLGAIIKNLPE-ELAGSLIKNRILL------VHHNKAS
A0A178DN93_9PLEO      TDDS-NDAMAITN--ARLLGALIGCLPE-DIGPGLLKARVLT------THFNKAS
A0A177D7J2_ALTAL      SDDS-NDAMAITN--ARLLGALISCLPE-EIASSLLKARVLT------THYNKAS
A0A0C4DS68_MAGP6      TDEK-DDAMTITN--AKLLGALVKNVPA-DAASHLIKTRVLT------PTPSSSS
A0A151N4J5_ALLMI      LGHD-EDTTRMAS--AGCLAELCAFLSE-EELNSILQQHLLADVSGIDWMVRHGR
A0A146NKU7_FUNHE      LGHD-EDATRMAS--AGCIGELCAFLSE-EELKNLLLQHVLADVSGVDWMVRHGR
A0A0F7Z917_CROAD      LGHE-EDATRMAS--AGCLAEMCAFLTD-EELGGVLQNHLLADVSGIDWMVRHGR
A0A0W0DC37_CANGB      MLSS-NDKLAVAY--AKLIGSLSEILSE-DEAKNILQEKVLD--ADMEGSAGKFA
A0A0L8RIK7_SACEU      MLGS-NDKLAVAY--AKLIGSLSEILSS-QEAHKILQDKVLN--ADLEGETGKFA
A0A178FRD2_TRIVO      SVD--DAATTITN--AKLAGALIKSLST-PTAIPLIKNRILA------TQLTHQS
A0A175WD14_9PEZI      TDER-DNAMTITN--AKLFGALIKNVSA-EVATGLLKNRVMT------KDVSISS
A0A194VFU1_9PEZI      TDER-DDNMTITN--AKLFGALMKNVPT-DLTTNLLKNRVMT------TNFSHSS
A0A151TXV6_CAJCA      IHHD-DDRVRMHA--ASILGILTQYLED-VQLTELIQE-LSNLANSPSWPPRHGS
A0A0M9ABE9_9HYME      LGHP-EDVTRNAV--AGCFGALIRWLSP-DQLNIALNEDLLCNDTSVDWMLRHGR
A0A072U5F0_MEDTR      IHHD-DDRVRVYA--ASILGVLTQYLEA-VQFTELIQE-VTSLANSPNWPPRHGS
A0A0K8V1F4_BACLA      IGHS-EDITRSAS--AGCLGALLKYLSI-QQVEDLLELHIFVAGNG-DGLLKHGR
A0A0A1XE05_BACCU      IGHS-EDITRTAS--AGCLGALLKYLTP-QQVDDLLEHHILIAGNG-DGLLKHGR
A0A131YTA4_RHIAP      LSSS-EDGCRTAA--AGCLGSLCRWLPP-DELAVFAREHLLSDDPSEDWTLRHGC
A0A0J6I2R9_COCPO      SADR-DEATNIAN--AQLVGALIKSLPE-ATAVPLIKNRVLT------SHYTHLS
A0A074XMM5_AURPU      TGDS-DEASAITN--ARLLGALIKVLPE-DVGGGLVKARVLT------PDHTYSS
A0A167FV98_9ASCO      LPSG-NGKPKVLSLLAKILSAIVGTVDG-EQAGKIIKQALQH-------EDENFS
A0A1A7XZM1_9TELE      LGHD-EDATRMAS--AGCIGELCAFLSE-EELKSVLHQHVLADVSGVDWMVRHGR
A0A0P7V1U6_9TELE      LGHD-EDATRMSS--AGCLGELCAFLSE-DELRGILTQHILADMSGVDWMVRHGR
A0A1A7WWE1_9TELE      LGHD-EDATRMAS--AGCIGELCAFLSE-EELKSVLHQHVLADVSGVDWMVRHGR
                                      .    .     .               :
```

```
GCN1_YEAST            ILTLNSFLKDAPTHIFNTGLID--EFVSYILNAIRSPDVYFGENGTIAAGKLLLL 2860
GCN1_SCHPO            VLILNAAVKFGSQKIIELKLSD--IVCSIISTASLQKEVTIAENGILALGKALLA
GCN1_DICDI            SLALGEIFKASGKNLIDSQSPNMPTIIKIIQTDCRDEKGPIRESSAYLAEAILVA
GCN1_MOUSE            SLALSVAVNVAPSRLCAGRYSN--EVQDMILSNAVADRIPIAMSGIRGMGFLMKY
GCN1_HUMAN            SLALSVAVNVAPGRLCAGRYSS--DVQEMILSSATADRIPIAVSGVRGMGFLMRH
M7ANV9_CHEMY          SLALSVAMKVAPSRLCAPKYYN--SVQERILSNATADRIPIAVSGIRGMGFLMKY
H8X4E7_CANO9          ILAINAFLKYAPELVKHNH-----DVVDFVAACSDSATPYISDNATIAIGKLVLS
W6A2P0_ICTPU          SMALAIAVKSAPKQLCAPEYSS--TVMDVVLSNTTADRIPIACSGIRAMGYLMRH
B6JZI6_SCHJY          VLILNAVVRFGFEKIISSGSES--AVAEYISRLCANSDPFISENAVKAAGKYLLT
R4WJK2_RIPPE          STALFVALKEAPERLYKDDFKE--KVQKRLLVFLTADRVPIAMNGVRGCGYLFEY
W8CCK9_CERCA          TTALFVALKECPVSIITSKFEN--KVITIITTSILSDKINIASNGVRALTCLLQY
B0W357_CULQU          TAALFVALKEFPAAIFIDKYET--KVCKTIVSSLASDKIPVALNGVRSAGYLLQH
E1ZX97_CAMFO          SAALFVVLKESSVTVYNPKDKD--RVCAVILSYLAADRVQIVMNGVRACGYLFQY
W5JJ28_ANODA          TAALFVALKEHPATIVTSKYET--KIAKVINGAIVSDKVPVAQNGVRAAGYLLQY
F1KPR4_ASCSU          TVALQHACATDAEALLNAYGIE--KLRSAIASAIQSDKSFIACAGVRAATELLL-
V9K7F0_CALMI          SIALSVAMKAAPGRLCTPEYNS--TVLDMIIASATADRIPIAMSGIRAMGFLLKH
W0T7Q3_KLUMA          ILTLNSFLRDAPEHIFGTGLID--ECVAYMINAAESTNAHFSDNGLIAIGKALLL
S9R951_SCHOY          VLILNAVIKYGSKKIIDLGLSE--SVSQAISDACQQTETYITENAVLAAGKALLA
S9W134_SCHCR          ILILNAVIKYGSKKIIEMGLSE--SVCQAISDACQQKETYITENAVLAAGKALLA
H0GUK0_SACCK          ILTLNSFLKDAPIHIFNTGLTN--EIVSYILKAFHSPDAYFGENGVIAAGKLLLL
E5R2E4_ARTGP          ILRLNAILVESPALL-SENFRS--EVPAAICHAIKNSDVFISDNGVLAAGKYLLS
J8Q888_SACAR          ILTLNSFLKDAPTHIFNTGLID--KFVSYILNALRSSDVYFGETGAIAAGKLLLL
C5G992_AJEDR          VLNLNALLVESAALL-VENFHS--ETASVICNGISNKDTFISDNSVLAAGKYLLS
G4MR95_MAGO7          VLGLNAVLLDAPAAIMEGPFAE--ELPELLVQGISSKIAMIADNSILAAGKYLLN
C9SQ16_VERA1          VLALNAVLVESPESLLGGPLAD--DLPEVLCHGMTNKTPFIADNFILATGKYLLS
B2WNI9_PYRTR          VLALNAILLDAPEAL-TGSFAD--DTITVICQGIAHSQPFISDNAILAAGKYLLS
S3CN37_OPHP1          VLALNAVLLESPEILFESELAE--TLPAYLSHGMVNKTPFIADNFILGTGKFLLS
G3ALT0_SPAPN          VLSINSFLKYSPDHIFNTGLLS--EIVDFVLACSDSTIDYISDNATVAIGKLLLL
Q7S5P0_NEUCR          VLALNAVLLESPETLLESSLVD--DLPELLCQGMASKNTFITENFILATGKYLLS
A0A165FVX9_9PEZI      VLGLNSILAESPLTL--APFTE--EVVGFICQGISNGNAYITEGCILAAGKYLLS
A0A178DN93_9PLEO      VLALNAILLDAPEAL-SESFPE--ETVATICQGIAHSQPFISDNAVLAAGKYLLA
A0A177D7J2_ALTAL      VLALNAILLDAPEAL-TESFAD--DTVSVICQGISHAQPFISDNSILAAGKYLLS
A0A0C4DS68_MAGP6      MLGLNAVLLESPKAILESPWAD--ELPDILCQGMSSKIALIADNSILAAGKYLLC
A0A151N4J5_ALLMI      SLALSVAVNVAPSRLCLPKYYN--TVQEMIFSNATADRIPIAISGIRGMGFLMKY
A0A146NKU7_FUNHE      SLALAIAVKTAPEKLCGKDYCE--TVTETVLTNATADRIPIATSGIRALGYLMRH
A0A0F7Z917_CROAD      SLALSVAVNTAASRLCAPKYSN--SVHEMIFSNTVADRIPIAIAGIRGMGFLMKH
A0A0W0DC37_CANGB      VLILNSFLKDAPSHIFSSGEIH--KFVKFITDAMTSTNVHFVENATLAAGKLLLL
A0A0L8RIK7_SACEU      ILTLNSFLKDAPIHIFNTGLVD--EVISYTVNAIQSPDVYFGENGAIAAGKLLLL
A0A178FRD2_TRIVO      ILRLNAILVESPALL-NENFRS--EVPVAICHAIRNSDVFISDNGVLAAGKYLLS
A0A175WD14_9PEZI      VLALNAVLLESPNTLLDSPLAD--DLPELLCQGMESKDTSIADNFIVAAGKYLLS
A0A194VFU1_9PEZI      VLALNSVLLESRSTLLDSPLAE--DLPELLCQGMAHNEPFIADNFILATGKYLLI
A0A151TXV6_CAJCA      ILTISSLFRYNPATICSSSLFP--TIVDCLRDTLKDEKFPLRETSTKALGRLLLY
A0A0M9ABE9_9HYME      SAALFVALKESPTTIYNSKEKD--RVCVVILSYLSADRIQIIMNGIRACGYLFQY
A0A072U5F0_MEDTR      ILTISSLLYRNPAPIFSSSLFQ--TVVDCLRDALKDEKFPLRESSTKALGRLLLY
A0A0K8V1F4_BACLA      TAVLFVALKESPSTIIISKYES--KLIEFITSSITSDKINIASSGVRAMTCLLHF
A0A0A1XE05_BACCU      TAVLFVSLKESPSMIITPKFES--KVIEFITASILSDKINIASSGVRAMTCLLQY
A0A131YTA4_RHIAP      SVTLSVALKQAPERILTDDWRD--RVVKTLIKYMTADRVPIVIGGVRGTGHCLRH
A0A0J6I2R9_COCPO      VLALNSILAESPRSI-TDTFPD--ETLSTICEGIKHKDVFIADNSVLAAGKYLLT
A0A074XMM5_AURPU      VLALNSILAEAPGSL-TGPFAD--VLPSTICQGISSSQPAIADNCVLASGKYLLT
A0A167FV98_9ASCO      ILILNGVLKYGSETVKNTDLSA--SIYKYFAERASSEHSNVAEASIIGLGKYLLS
A0A1A7XZM1_9TELE      SLAVAIAVKSAPEKLCGKDYCD--TVTETILANATADRIPIATSGIRAMGFLMRH
A0A0P7V1U6_9TELE      SVALAIAVKSAPEQLWAEEYRT--AVLDAILASATADRIPVATSGIRAMGFLMKH
A0A1A7WWE1_9TELE      SLAVAIAVKSAPEKLCGKDYCD--TVTETILANATADRIPIATSGIRAMGFLMRH
                         :           :                            .            .
```

```
GCN1_YEAST          EGEKRSPFVKKDAAEPFKIGDENINLLINELSKAVLQPASNSTDVRRLALVVIRT 2915
GCN1_SCHPO          DIPQSF---------------GNAKNLVEALKVNIEAPPSTSQDSRRLALLIIRV
GCN1_DICDI          ---------------------SPLTYAKDLVPSICHLIGDQSSSVSISALNVIKR
GCN1_MOUSE          HIET-----------------GSGQLPPRLSSLLIKCLQNPCSDIRLVAEKMIWW
GCN1_HUMAN          HIET-----------------GGGQLPAKLSSLFVKCLQNPSSDIRLVAEKMIWW
M7ANV9_CHEMY        HIET-----------------EGGNLPPKLANLFIKCLQNPSSDIKLIAEKMIWW
H8X4E7_CANO9        GIKDES--------------------LYRQLAKNVIQPNSASPDTRRLSLIVIRT
W6A2P0_ICTPU        QLRSA----------------GEDAVSPRIITQLVKSLQNQSSDIRLVTERVLWW
B6JZI6_SCHJY        EMNQNF---------------NDAKRLIESLAECIQAPVSGSNDCKRLALVVLHT
R4WJK2_RIPPE        LMS------------------NNEPVPQPLLSPFVRSMNHQSNEVKQLLARVCSY
W8CCK9_CERCA        YLI------------------NNLTFSPIIVPCLARAMNHKSNEIKQIVAKSCNY
B0W357_CULQU        GMST-----------------EGAKLPQPIIGPFVKSMNHVSNEVKQLLAKTCIY
E1ZX97_CAMFO        LMN------------------ESLPIPQQILSPFVRSMNNNSNDVKQLLARVCIH
W5JJ28_ANODA        CMTDA----------------EGVKLPMTVIGPFVKSMNHSSHEVKQLLAKTCTY
F1KPR4_ASCSU        ---------------------NERTMDITLLSALVRGINHPSNDVKRIAAIGVHH
V9K7F0_CALMI        QMQL-----------------KDESVSPKLLSLLVKSLQNSASDLKLIAEKMIWL
W0T7Q3_KLUMA        EGETKSPYSSGDVKEPFHLGEENIQKMVSELAKSMLKPNSNSLDSRRLALVVVRT
S9R951_SCHOY        DIPQNF---------------NESKTLFEALRVSIEVSPSGSQDAKRLSLVIIRI
S9W134_SCHCR        DVPQNF---------------NESKVLFEALRISLEVSPSGSQDSKRLSLVIIRI
H0GUK0_SACCK        EGERKSPFVRTEAAEPFDIGDENINLLISELSKAILQPASNSTDVRRLSLVVIRT
E5R2E4_ARTGP        PQMDRKP--------------EDEKEVFEALAGIV--QPGKPVDTRRLALVVLRT
J8Q888_SACAR        EGEKKSPFVKTEAAEPFKIGDENINALINELSKAVLEPASNSTDVRRLTLVVIRT
C5G992_AJEDR        EDITRNF--------------ETDKPLVEALAPAI--KPGGPTDTRRIALVIVRT
G4MR95_MAGO7        EQS-KPF--------------EHTKAVFEALANTV--GPGNPTDSRRLSLVVVRT
C9SQ16_VERA1        ESP-KTF--------------EANKKIFETLAGII--QPGQPTDSRRLALVVART
B2WNI9_PYRTR        EKSNKSF--------------DHTKPIFEALAPVV--EPGHPVDTRRLALVVLRT
S3CN37_OPHP1        QQA-KTF--------------EDTKEIFETLAKII--PPGAAADSRRLSLVLVRT
G3ALT0_SPAPN        HKENKSPFSKEAT-SIYEVDEESLDKLVRQLCVLVIQPKSSSPDTRRLALVVLRT
Q7S5P0_NEUCR        PVP-KSF--------------EATKPLFSTISTLL--PPGQPTDSRRLALVLVRT
A0A165FVX9_9PEZI    ESGDKSF--------------ETTKSIFEVLAPII--KPGSPADARRLALVVLRT
A0A178DN93_9PLEO    EKTNKTF--------------EHTKPLFEALAPVI--EPGHPVDTRRLALVVLRT
A0A177D7J2_ALTAL    EKSNKSF--------------DHTKPIFEALAPVV--EPGHPVDTRRLALVVLRT
A0A0C4DS68_MAGP6    DSP-KSF--------------ESTKSVFEALADII--QPGRPTDSRRLSLVIVRT
A0A151N4J5_ALLMI    HIEA-----------------EGGNLPLKLSNFFIKCLQNPSSDIRLVAEKMIWW
A0A146NKU7_FUNHE    QLRTE----------------GGSSISQRTITQFVKCLQNQSSDIRLVSERVLWW
A0A0F7Z917_CROAD    HIEA-----------------NGGNLPPKLSNLFIKCLQNPSSDIKLIAEKMIWW
A0A0W0DC37_CANGB    HNETKSPYTNMKNDVTFIVPEEDIKSLVEELAKSALVPSSNTTDQRRLSLVVLRT
A0A0L8RIK7_SACEU    EGERRSPFVKTDAPEAFKIGDENISLLIKELSKAVLQPASNSTDVRRLALVVIRT
A0A178FRD2_TRIVO    AHMDRKP--------------EDEKEVFEALAGIV--QPGKPVDTRRLTLVVLRT
A0A175WD14_9PEZI    DAP-KAF--------------EATKPIFTTLSKII--PPGGPTDSRRLALVLVRT
A0A194VFU1_9PEZI    DTP-KSF--------------ESTKAIFESLAVLI--PPGAALDSRRLSLVVVRT
A0A151TXV6_CAJCA    RAQTDP---------------SDTLLYKDVLSLLVSSTHDDSSEVRRRALSAIKA
A0A0M9ABE9_9HYME    LMN------------------EGQPIPQQILPPFIRSMNNNNNDVKQLLAKVCIH
A0A072U5F0_MEDTR    RAQEDP---------------SDTVLYKDVLSLLVTSTRDESSEVRRRALSAIKA
A0A0K8V1F4_BACLA    YMS------------------NNIMFSPIIVSCFTRAMNHKSNEIKQIVAKSCNY
A0A0A1XE05_BACCU    YMS------------------NDIIFPLTIVSCFIRAMNHKSNEIKQVVAISCNY
A0A131YTA4_RHIAP    MLLT-----------------NSEELPQVLLTTFAKCLNHGSNEVKQLVAQTVQW
A0A0J6I2R9_COCPO    TDIDRDA--------------ETDRVVLEALTSVI--PPGNPADTRRVALVVLRT
A0A074XMM5_AURPU    PTSDTSF--------------EPVKQIMEALAAAV--APGKPVDTRRLALVVLRT
A0A167FV98_9ASCO    ISIPES---------------EETEEIIKKLAESIEKSESRSTDTRRLALVVLRT
A0A1A7XZM1_9TELE    HLKTE----------------GGSSVLQRIVTQLVKCLQNQSSDIRLVSERVLWW
A0A0P7V1U6_9TELE    HIVTE----------------GGAGVSPRIVTQFIKCLQNQSSDIRLVAERMLWW
A0A1A7WWE1_9TELE    HLKTE----------------GGSSVLQRIVTQLVKCLQNQSSDIRLVSERVLWW
                                                                .
```

```
GCN1_YEAST          LARFKFDECIKQ-YFDVVGPSVFSCLRDPVIPIKLAAEKAYLALFKLV-EEDDMH 2970
GCN1_SCHPO          VSKENYSL-IKP-HISILAPAIFGCVRAIVIPVKLAAEAAFLALFQLV---EDDS
GCN1_DICDI          FCKSNQQL-SRQYLRDIVVPTMNRLKERTNLPLKLAAERTLVHSLQIF---KESI
GCN1_MOUSE          ANKEPRPP-LEPQTIKPILKALLDNTKDKNTVVRAYSDQAIVNLLKMR---RGEE
GCN1_HUMAN          ANKDPLPP-LDPQAIKPILKALLDNTKDKNTVVRAYSDQAIVNLLKMR---QGEE
M7ANV9_CHEMY        ANKNHLPS-MDPQTIKPILKALLDNTKDKNTSVRAYSDQAIVNLLKMR---EGEE
H8X4E7_CANO9        AAHKKA---IPQEYLDFLVPSVFSSVRDPIIPIKLAAEKAYLEVFSIV--DQELK
W6A2P0_ICTPU        VWKEADTPVLEPSLIKPIIKALLDNTKDKNTSVRAQSEHTIVNLLRLR---QGDS
B6JZI6_SCHJY        VANSNFDV-VRA-HLPTLVPAVFGCVRATVIPVKLAAETTFLSLLQLR---ENEA
R4WJK2_RIPPE        LARR---EINSPEFLKAVIPMLVNGTKEKNSYVKANSELSLVSVLRLR---NGDE
W8CCK9_CERCA        LAKELPIEKMSPDVIKLLVPMLVNGTKEKNGYVKSNSEIALISLLHLR---EDDT
B0W357_CULQU        LAKTVPADTTAPEYLRLVIPMLVNGTKEKNGYVKSNSEIALVYVLRLR---EGDE
E1ZX97_CAMFO        LARNIPSENMSTELLRALLPMLVNGTKEKNGYVKANSELALIAVLRLR---QGED
W5JJ28_ANODA        LARVVPPAKTAPEYLKLAIPMLVNGTKEKNGYVKSNSEIALVHVLRLR---DGEE
F1KPR4_ASCSU        IAVRD----LSPVQMKAIIPMMVNGTKEKNTAVRVACEHALCDALKLR---VNST
V9K7F0_CALMI        VNKDNLQP-LEIQTIKPILKALLDNTKDKNTSVRAYSEQAIVSLLRLR---EGEE
W0T7Q3_KLUMA        LARFKFEEAIHS-NYDILGPSVFSCLRDPIIPIKLAAEKAYLAMFHLV-EEEGME
S9R951_SCHOY        LSKEKYDI-VRP-HLNILAPAVFSCIRAITIPIKLAAEAAFLAMFKLV---DDDS
S9W134_SCHCR        LSKEKYDI-VRP-HLNILAPAVFSCIRAIVIPIKLAAEAAFLAMFKLV---DDDS
H0GUK0_SACCK        LARFKFDECLKQ-YYDVVGPSVFACLRDPVIPIKLAAEKAYLALFKLV-EEDDMN
E5R2E4_ARTGP        VARENQDM-IAP-YRSLVVPPVFGGVRDTVIPVKLAAEAAFLGIFSVV--ECEGE
J8Q888_SACAR        LARFKFDECIKP-YYDVLGPSVFACLRDPVIPIKLAAEKAYLALFELV-DEDNMD
C5G992_AJEDR        VSRLHPEL-LRP-HLPLLAPPIFASVRDVVIPVKLAAEAAFLALFSVV--ESEST
G4MR95_MAGO7        VSRVNMDL-ARP-HLPQLAGPVFASVRDPVIPVKLAAEAAFVSLFNVA--DDESK
C9SQ16_VERA1        LSRVDMDM-VRP-HTPLLATPVFASVRDPVIPVKLAAEAAFVSLFNVA--DEESK
B2WNI9_PYRTR        LAREHNEL-VRP-HIALVVPVVFASVRDPVIPVKLSAEAAFLSIFSVV--DEEGA
S3CN37_OPHP1        LARVHPDM-VRP-HLPLLAPPVFASVRDLVIPVKLSAEAAFMALFDVI--DEETK
G3ALT0_SPAPN        VARLKFDE-VHD-NLDLIVPSIFASIRDPIIPIKLAAEKAYLAVFQLV-EDQDMK
Q7S5P0_NEUCR        LARTNPDL-VRP-HLALLAPPVFASVRDMVIPVKLAAEAAFVQLFAVA--DEESK
A0A165FVX9_9PEZI    VSRMDNEL-VRP-HLALLAPPTFASVRDMVIPVKLSAEAAFLALFSVV--DDESA
A0A178DN93_9PLEO    VAREHNEL-VRP-HIPLLTPAVFASVRDPVIPVKLSAEAAFLTIFSVV--DEETA
A0A177D7J2_ALTAL    VAREHNDL-VRP-HIPLLIPVVFASVRDPVIPVKLSAEAAFLAIFSVV--DEEAA
A0A0C4DS68_MAGP6    VSRANIDM-ARA-HLPLLAPPVFASVRDPVIPVKLAAEAAFVALFNVA--DDESK
A0A151N4J5_ALLMI    ANKKQMPS-LDPQTIKPILKALLDNTKDKNTSVRAYSDQAIVNLLKMR---EGEE
A0A146NKU7_FUNHE    VFKDPDTPSMDASQIKPLLKSLLDNTKDKNTTVRAQSEHTIVNLLRLR---QGED
A0A0F7Z917_CROAD    ANKAPLPP-LDPPVAKPILKALLDNTKDKNTSVRAYSDQAIVNLLKMR---EGEE
A0A0W0DC37_CANGB    IARLKNEETVKP-YLDVLAPAVFSCVRDVVIPIKLAAEKAFLALFGLI-EEEDMA
A0A0L8RIK7_SACEU    LARFKFEECIKP-YYNLLGPSVFACLRDPVIPIKLAAEKAYLALFKLA-EENDMH
A0A178FRD2_TRIVO    VARENQEM-IAR-YRSLVVPPVFGGVRDTVIPVKLAAEAAFLAIFSVV--ESEGE
A0A175WD14_9PEZI    LARTHPDM-ARP-HLGLLAPPVFASVRDMVIPVKLAAEAAFVQLFAVA--DEEGK
A0A194VFU1_9PEZI    VSRANMDM-VRP-HLGLLAPPIFASVRDMVIPVKLAAEAAFVGLFNVV--DDESK
A0A151TXV6_CAJCA    VAKANPSAIML--HGTIVGPALAECLKDANTPVRLAAERCALHAFQLTKGSDNVQ
A0A0M9ABE9_9HYME    LARNIPPEKMSPELLKSLLPMLVNGTKEKNGYVKANSELALIAVLRLK---QGEE
A0A072U5F0_MEDTR    VAKANPSAIMS--HGTVIGPALAECLKDANTPVRLAAERCAIHAFQLTKGSENVQ
A0A0K8V1F4_BACLA    LAKTLAIDKMSPDVIKQLVPMLVNGTKEKNGYVKSNSEIALVSILHLR---ENDT
A0A0A1XE05_BACCU    LAKMLALDKMSPDVIKQLVPMLVNGTKEKNGYVKSNSEIALVSILHLR---DNDT
A0A131YTA4_RHIAP    LSRSLDKG-VPPQLLRTLVPQLVNGTKEKNTMVRANSEYALVALLHLR---TSTQ
A0A0J6I2R9_COCPO    VSRLEPGL-VGP-HLSLLIPPVFSSVRDLVIPIKLGAEATFLALLQVV--DAGSV
A0A074XMM5_AURPU    VSREQNDL-IRP-HLALLTPAVFGSVRDMVIPVKLAAEAAFLAMFSVV--EEEDA
A0A167FV98_9ASCO    IAREKYQL-VSP-YIDLIAVPLFGCVRDMIIPVKLAAEKVYLTIFKLV-EDPEGK
A0A1A7XZM1_9TELE    VFRDPSVPPMDTSLIKPLLKSLLDNTKDKNTTVRAQSEHTIVNLLQLR---QGEE
A0A0P7V1U6_9TELE    VCRDPSTPALESSIIKPLLKSLLDNTKDKNTGVRAQSEHTIVSLLRLR---QGEQ
A0A1A7WWE1_9TELE    VFRDPSVPPMDTSLIKPLLKSLLDNTKDKNTTVRAQSEHTIVNLLQLR---QGEE
                                              .     ::  .:      : :
```

```
GCN1_YEAST          TFNEWFAKISDRGNSIETVTGTTIQLRSVGDYTKRVGKRLANVERERIAAGGD-- 3025
GCN1_SCHPO          VLNKYIETLEGP------------RARSFVDYSRRVAVKLAAAERDRINSGSE--
GCN1_DICDI          VMDDLIKQLELSGDSS--------MANSLIDYHKRVLMKLSPDSD----------
GCN1_MOUSE          LLQSLSKILDVA------------SLEALNECSRRSLRKLACQ------------
GCN1_HUMAN          VFQSLSKILDVA------------SLEVLNEVNRRSLKKLASQ------------
M7ANV9_CHEMY        VLQSVSKILDAA------------SLELLNESCRRSLKKLASQ------------
H8X4E7_CANO9        VFDEWFNG----RTEITTVTGTPIVPRSIGDYTKRVAVRLANVERERIEQGGD--
W6A2P0_ICTPU        VMQNISAILDTA------------SNELLSECYRRSLKKISGL------------
B6JZI6_SCHJY        FAEKFISTLQTP------------RARSISDYTRRVAFKLAAAERDRIASGSG--
R4WJK2_RIPPE        KLQSCIAVLDPG------------AAEALTDVITKVLRKVALQ------------
W8CCK9_CERCA        TYNNVCQILEVG------------AKDSLNEVVHKVLRKVAVQ------------
B0W357_CULQU        VHQKCIALLEPG------------ARDFLAEVVSKVLRKVALQ------------
E1ZX97_CAMFO        EHQRCMAFLDIG------------AKESLSDVVSKVLRKVLSQ------------
W5JJ28_ANODA        FHQRCLTLLEPG------------ARESLSEVVSRALRKVAMQSV----------
F1KPR4_ASCSU        VYDQYLATLEGA------------AREVLIETHRQLLKLIKQQ------------
V9K7F0_CALMI        IYQNVSKILDTA------------SLDLLAESYRRSLKKLASQ------------
W0T7Q3_KLUMA        TFNSWFSKLEGSTVSNSIG--DTLQLRSIGDYTKRVGKRLASVEREKISAGGD--
S9R951_SCHOY        VINKYMETLEGP------------RARSFGDYTRRVAFKLAAGERDRIASGSG--
S9W134_SCHCR        IINKYMETLEGP------------RARSFTDYTRRVAFKLAAGERDRIASGSG--
H0GUK0_SACCK        TFNEWFSRASDGADRIETITGTSIQLRSIGDYTKRVGKRLANVERERIAAGGD--
E5R2E4_ARTGP        VFEEYMKG-PGAELPAG-------PKRSMQDYFKRVALRLGGQARERREAEGG-Q
J8Q888_SACAR        TFNEWFSRISDGKSTIESVTGTIIQLRSIGDYTKRVGKRLANVERERIAAGGD--
C5G992_AJEDR        VFEKYMNG-PGAELPTG-------TKRSLQDYFKRVALRLANQARERREAEGG-Q
G4MR95_MAGO7        VFDKFMASPAGESMPAN-------AKRSMQDYFKRVALRLGAQVRERREAEGG-A
C9SQ16_VERA1        VFDKYVDN---AGLAPN-------AKRSMQDYFKRVALRLGAQVRERREAEGG-Q
B2WNI9_PYRTR        VFDKYMSG-PGKELSPG-------QQRSMGDYFKRVATRLAGQARERKEAEGG-A
S3CN37_OPHP1        VFDKYMASPSAAELPPA-------TKRVMGDYFKRVTLRLATQARERRDAEGG-T
G3ALT0_SPAPN        LFNQWFEG----KSTVTTVTGTSIVPRSIGDYTKRVASRLAGVERERIEAGGD--
Q7S5P0_NEUCR        VFEKYLES-QEGNMAPN-------VKRSMQDYFKRVALRLGAQVRERREAEGG-T
A0A165FVX9_9PEZI    VFDKYMAG-AGAELPPN-------VKRSMGDYFKRVAIRLGNQARERKEAEGG-H
A0A178DN93_9PLEO    VFDKYMSG-PGKSLSAG-------QQRSMGDYFKRVALRLAGQARERKEAEGG-A
A0A177D7J2_ALTAL    VFDKYMAG-PGKTLSAG-------QQRSMGDYFKRVAMRLAGQARERKEAEGG-A
A0A0C4DS68_MAGP6    VFDKFMAG-AGAELPPN-------TKRSMQDYFKRVALRLGSQVRERREAEGG-A
A0A151N4J5_ALLMI    VLQSVSKILDAA------------SLDLLNESCRRSLKKLASQ------------
A0A146NKU7_FUNHE    TMQSVTAILDSA------------SNELLSECHRRSLKKIASL------------
A0A0F7Z917_CROAD    MLQSISKILDAA------------SLELLNESCRRSLKKLANQ------------
A0A0W0DC37_CANGB    TFNNWFESVSSNGATIDNIVGTTIQLRSIGDYTKRVAKRLAAVERERTAAGGD--
A0A0L8RIK7_SACEU    TFNEWFSGISGGESTIESITGTTIQLRSIGDYTKRVGKRLANVERERIAAGGD--
A0A178FRD2_TRIVO    VFEEYMKG-PGAELPAG-------PKRSMQDYFKRVALRLGGQARERREAEGG-Q
A0A175WD14_9PEZI    VFDKWIAG---AELPPT-------VKRSMQDYFKRVALRLGAQVRERREAEGG-A
A0A194VFU1_9PEZI    VFDKFMASDIGTALPPN-------VKRSMGDYFKRVALRLGSQARERREAEGGMS
A0A151TXV6_CAJCA    AAQKYITGLDAR------------RLSKFPEY--RYLFNQICI------------
A0A0M9ABE9_9HYME    EYQRCMAFLDVG------------ARESLSDVVSKVLRKVLSQ------------
A0A072U5F0_MEDTR    AVQKYITGLDAR------------RLSKFPEFSDDSGDSDEDM------------
A0A0K8V1F4_BACLA    TFSYICQLLEVG------------ARDSLNEVVNKVLRKVAVQ------------
A0A0A1XE05_BACCU    TFSYVCQILEVG------------ARDSLNEVVNKVLRKVAVQ------------
A0A131YTA4_RHIAP    GLDECLAVLDPG------------ARESLQDVYTKVLRKVASH------------
A0A0J6I2R9_COCPO    IFDTYLQG-PGAGLPMT-------TKRSMQDYFKRVALRLATQARERRDAEGG-A
A0A074XMM5_AURPU    IFAKYMAG-PGAELAPG-------PKRSIQDYFKRIATRLGAQARERREAEGG-Q
A0A167FV98_9ASCO    IFEEWFNNAKNSGVIPA------PQQRSINDYTRRVAFRLAQAERDRIAAGGD--
A0A1A7XZM1_9TELE    TMQSMTAILDSA------------SNELLSECHRRSLKKIASL------------
A0A0P7V1U6_9TELE    LMQV------------------------------RAGTYINTQ------------
A0A1A7WWE1_9TELE    TMQSMTAILDSA------------SNELLSECHRRSLKKIASL------------
```

```
GCN1_YEAST          -AETMFSDRFEDEREIWAVGGVEL------TTDI 3059
GCN1_SCHPO          ---RVKLEEVEDLAEINAVGRDNE----VSTNDP
GCN1_DICDI          --------------------------------IEK
GCN1_MOUSE          ---ADSVEQVDDT------------------ILT
GCN1_HUMAN          ---ADSTEQVDDT------------------ILT
M7ANV9_CHEMY        ---ADSVEQIDDT------------------ILT
H8X4E7_CANO9        -EETLFSDRIEDEKEIWSVGI-------------
W6A2P0_ICTPU        ---PDSSEEIDDT------------------ILT
B6JZI6_SCHJY        ---RIQIEEAENLAEINAIGADDG---MFSQQDA
R4WJK2_RIPPE        --PEGKVEELDDT------------------LLT
W8CCK9_CERCA        --PLGKDEEFDDT------------------ILN
B0W357_CULQU        --PVGKEEELDDT------------------ILT
E1ZX97_CAMFO        --PEGKIEELDDT------------------LLT
W5JJ28_ANODA        ---IGKDEELDDT------------------ILT
F1KPR4_ASCSU        --SDAGIEDISDIP-----------------CVP
V9K7F0_CALMI        ---PESTEHIDDT------------------ILT
W0T7Q3_KLUMA        -AEAMFSDRFEDENEIWAVGGIEL------NPDA
S9R951_SCHOY        ---RVQLEEREDLAEITAVGRDNE---VVSNESW
S9W134_SCHCR        ---RIQLEEKEDLAEITAVGRDNE---VISNESW
H0GUK0_SACCK        -AETMYSDRFEDEREIWAVGGVEL------TTDI
E5R2E4_ARTGP        GGLGLSSDEVEDEKEVWSVGKVDL---GESFGDD
J8Q888_SACAR        -AETMFSDRFEDEREIWAVGGVEL------PTDI
C5G992_AJEDR        GGLGLSSDELEDEKEVWSVGKIDL---DESTSED
G4MR95_MAGO7        GGLGLSNDEVEDEKEIWSVGRIDS--GEDVFGKD
C9SQ16_VERA1        GGLGLSNDEVEDEKEIWSVGKVDV--GEDIFGK-
B2WNI9_PYRTR        GGLGLSSDEVEDEREIWSVGKVEL---GDVFGDN
S3CN37_OPHP1        GGLGLFDDEVEDEREIWSVGKVDV--GADVFGKE
G3ALT0_SPAPN        -AETMFSDRFEDETEVWAVGGTEI-------AST
Q7S5P0_NEUCR        GGLGLSNDEVEDEKELMSVGRVDVAGGGEMFD--
A0A165FVX9_9PEZI    GGLGLSADELEDEREIWSVGRVDLG-GDLGFGDA
A0A178DN93_9PLEO    GGLGLSSDEMEDEREIWSVGKVEL---GDVFGEN
A0A177D7J2_ALTAL    GGLGLSSDEMEDEREIWSVGKVEL---GDVFGEN
A0A0C4DS68_MAGP6    GGLGLSNDEVEDEKEIWSVGKMDD--GANVFGQD
A0A151N4J5_ALLMI    ---SDSDEQIDDT------------------ILT
A0A146NKU7_FUNHE    ---PDSNEEIDDT------------------ILT
A0A0F7Z917_CROAD    ---ADSVEQIDDT------------------ILT
A0A0W0DC37_CANGB    -DEMMFSDRYEDEREIWAVGGVEL------PTDI
A0A0L8RIK7_SACEU    -AETMFSDRFEDEREIWAVGGVEL------TTDI
A0A178FRD2_TRIVO    GGLGLSSDEVEDEKEVWSVGKVDL---GESFGDD
A0A175WD14_9PEZI    GGLGLSNDEVEDEKELMAVGKVDV--GSDIFAAE
A0A194VFU1_9PEZI    GTLGLSGDEVEDEKEIWSVGKVDV--GADVFGK-
A0A151TXV6_CAJCA    --------------------------------SIE
A0A0M9ABE9_9HYME    --PEGKIEELDDT------------------LLT
A0A072U5F0_MEDTR    --------------------------------STS
A0A0K8V1F4_BACLA    --AIDKDEELDDT------------------ILN
A0A0A1XE05_BACCU    --SIGKDEEFDDT------------------MLN
A0A131YTA4_RHIAP    --PEPKEEDLDDG------------------ILS
A0A0J6I2R9_COCPO    GGLDLASDEADDEKELWSVGKVDL----GDFSED
A0A074XMM5_AURPU    GGLGLSSDEQEDMKEVWSVGKVDV--ESGAFKDE
A0A167FV98_9ASCO    -DETLFSDRIEDMNEIWSVGNVEV------SSRN
A0A1A7XZM1_9TELE    ---PDSNEEVDDT------------------ILT
A0A0P7V1U6_9TELE    -----ACPDCRDA------------------AAP
A0A1A7WWE1_9TELE    ---PDSNEEVDDT------------------ILT
```
