# Supplementary material for: Recognition of a structural domain (RWDBD) in Gcn1 proteins that interacts with the RWD domain containing proteins
Source: Biol Direct. 2017 May 19;12:12. doi: 10.1186/s13062-017-0184-3 (PMC5438488; doi:10.1186/s13062-017-0184-3)

Additional file 2: Sequence-structural alignment between yeast Gcn1 sequence and the template PDB ID 3W3W Chain A represented using JOY

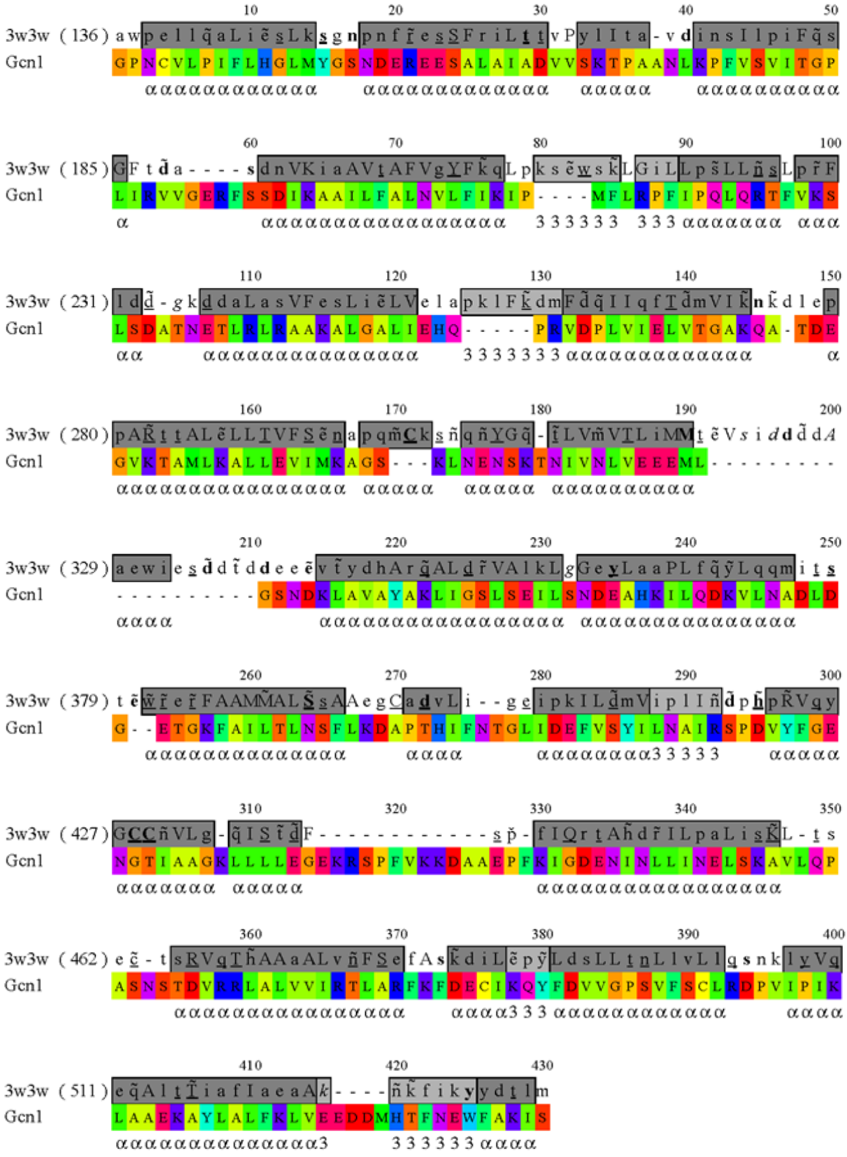

Supplement: Supplementary file 2 — Sequence-structural alignment between yeast Gcn1 sequence and the template PDB ID 3W3W Chain A represented using JOY. (PDF 2278 kb) [file 13062_2017_184_MOESM2_ESM.pdf]
