# Supplementary material for: Recognition of a structural domain (RWDBD) in Gcn1 proteins that interacts with the RWD domain containing proteins
Source: Biol Direct. 2017 May 19;12:12. doi: 10.1186/s13062-017-0184-3 (PMC5438488; doi:10.1186/s13062-017-0184-3)
Supplement: Supplementary file 5 — Hidden Markov model of the RWD Binding Domain (RWDBD) region. (PDF 65 kb) [file 13062_2017_184_MOESM5_ESM.pdf]

Additional file 5: Hidden Markov model of the RWD Binding Domain (RWDBD) region

```
HMMER3/f [3.1b2 | February 2015]
NAME  Multiple_alignment
LENG  379
ALPH  amino
RF    no
MM    no
CONS  yes
CS    no
MAP   yes
DATE  Tue Mar  7 03:12:44 2017
NSEQ  52
EFFN  1.431396
CKSUM 2957843090
STATS LOCAL MSV      -11.1835  0.69959
STATS LOCAL VITERBI  -12.0138  0.69959
STATS LOCAL FORWARD   -5.1609  0.69959
HMM          A        C        D        E        F        G        H        I        K        L        M        N        P        Q        R        S        T        V        W        Y   
            m->m     m->i     m->d     i->m     i->i     d->m     d->d
  COMPO   2.42433  4.43724  3.07892  2.72750  3.46054  3.04464  3.86553  2.65944  2.66095  2.21926  3.58847  3.16498  3.40899  3.07859  2.94273  2.64578  2.79474  2.52167  5.17398  3.91393
          2.68618  4.42225  2.77519  2.73123  3.46354  2.40513  3.72494  3.29354  2.67741  2.69355  4.24690  2.90347  2.73739  3.18146  2.89801  2.37887  2.77519  2.98518  4.58477  3.61503
          0.01839  4.40100  5.12334  0.61958  0.77255  0.00000        *
      1   2.18197  4.40304  3.66090  3.54745  4.83204  0.72975  4.68932  4.27991  3.76235  3.97874  4.78775  3.61522  3.94725  3.99244  4.02736  2.43048  2.99428  3.59071  6.16205  4.97911      1 g - - -
          2.68618  4.42225  2.77519  2.73123  3.46354  2.40513  3.72494  3.29354  2.67741  2.69355  4.24690  2.90347  2.73739  3.18146  2.89801  2.37887  2.77519  2.98518  4.58477  3.61503
          0.01839  4.40100  5.12334  0.61958  0.77255  0.48576  0.95510
      2   2.97952  4.37844  4.55451  3.99223  3.48440  4.18609  4.56893  1.37997  3.87270  1.95845  3.38622  4.20121  2.64941  4.09930  4.05261  3.50419  3.22238  1.65740  5.18399  4.00032      2 i - - -
          2.68618  4.42225  2.77519  2.73123  3.46354  2.40513  3.72494  3.29354  2.67741  2.69355  4.24690  2.90347  2.73739  3.18146  2.89801  2.37887  2.77519  2.98518  4.58477  3.61503
          0.01839  4.40100  5.12334  0.61958  0.77255  0.48576  0.95510
      3   2.70612  5.00456  2.92978  2.48641  4.40819  3.14352  3.76380  3.85569  2.54838  3.40940  4.19748  2.00347  3.89877  2.48077  3.01777  2.31232  2.05867  3.46236  5.60024  4.23466      3 n - - -
          2.68618  4.42225  2.77519  2.73123  3.46354  2.40513  3.72494  3.29354  2.67741  2.69355  4.24690  2.90347  2.73739  3.18146  2.89801  2.37887  2.77519  2.98518  4.58477  3.61503
          0.01839  4.40100  5.12334  0.61958  0.77255  0.48576  0.95510
      4   1.72099  2.18300  3.94156  3.49266  4.21137  3.22558  4.39737  3.59652  3.44076  3.30532  4.15328  3.60135  1.95479  3.71433  3.73732  1.90245  2.89321  3.16063  5.60971  4.41376      4 a - - -
          2.68618  4.42225  2.77519  2.73123  3.46354  2.40513  3.72494  3.29354  2.67741  2.69355  4.24690  2.90347  2.73739  3.18146  2.89801  2.37887  2.77519  2.98518  4.58477  3.61503
          0.01839  4.40100  5.12334  0.61958  0.77255  0.48576  0.95510
      5   3.41623  4.65238  5.34794  4.83317  3.66723  4.89949  5.43184  0.98406  4.73231  1.65568  3.43443  5.01316  5.10681  4.89662  4.85499  4.29383  3.66242  1.50881  5.73857  4.60029      5 i - - -
          2.68618  4.42225  2.77519  2.73123  3.46354  2.40513  3.72494  3.29354  2.67741  2.69355  4.24690  2.90347  2.73739  3.18146  2.89801  2.37887  2.77519  2.98518  4.58477  3.61503
          0.01839  4.40100  5.12334  0.61958  0.77255  0.48576  0.95510
      6   3.76630  5.04431  5.29637  4.81488  2.81269  4.94598  5.07792  2.47885  4.64636  0.52971  3.06006  5.01406  5.09740  4.67216  4.69390  4.37811  3.99237  2.69660  5.19945  3.93258      6 L - - -
          2.68618  4.42225  2.77519  2.73123  3.46354  2.40513  3.72494  3.29354  2.67741  2.69355  4.24690  2.90347  2.73739  3.18146  2.89801  2.37887  2.77519  2.98518  4.58477  3.61503
          0.01839  4.40100  5.12334  0.61958  0.77255  0.48576  0.95510
      7   2.67829  4.60835  3.55623  3.43304  4.66592  3.30738  4.59374  4.20880  3.56368  3.88348  4.77608  3.63569  0.68013  3.90140  3.83593  2.53935  3.18159  3.65522  5.98614  4.74958      7 P - - -
          2.68618  4.42225  2.77519  2.73123  3.46354  2.40513  3.72494  3.29354  2.67741  2.69355  4.24690  2.90347  2.73739  3.18146  2.89801  2.37887  2.77519  2.98518  4.58477  3.61503
          0.01839  4.40100  5.12334  0.61958  0.77255  0.48576  0.95510
      8   3.38217  4.61645  5.33294  4.83949  3.83239  4.89521  5.52028  0.91705  4.74869  2.27395  3.40812  5.01834  5.13456  4.97731  4.90828  4.30238  3.63902  1.24676  5.88864  4.70143      8 i - - -
          2.68618  4.42225  2.77519  2.73123  3.46354  2.40513  3.72494  3.29354  2.67741  2.69355  4.24690  2.90347  2.73739  3.18146  2.89801  2.37887  2.77519  2.98518  4.58477  3.61503
          0.01839  4.40100  5.12334  0.61958  0.77255  0.48576  0.95510
      9   3.72095  4.99011  5.15693  4.73108  0.97398  4.77075  4.39252  2.71342  4.56084  1.38289  3.31418  4.70281  4.99856  4.55450  4.58327  4.16754  3.94793  2.85347  4.53368  2.95112      9 f - - -
          2.68618  4.42225  2.77519  2.73123  3.46354  2.40513  3.72494  3.29354  2.67741  2.69355  4.24690  2.90347  2.73739  3.18146  2.89801  2.37887  2.77519  2.98518  4.58477  3.61503
          0.01839  4.40100  5.12334  0.61958  0.77255  0.48576  0.95510
     10   3.03490  4.70767  3.97289  3.34680  3.58536  3.96710  4.11913  2.70519  2.81395  1.32084  3.49981  3.70718  4.31893  3.40824  1.78025  3.27677  3.25539  2.78288  5.19060  3.97793     10 l - - -
          2.68618  4.42225  2.77519  2.73123  3.46354  2.40513  3.72494  3.29354  2.67741  2.69355  4.24690  2.90347  2.73739  3.18146  2.89801  2.37887  2.77519  2.98518  4.58477  3.61503
          0.01839  4.40100  5.12334  0.61958  0.77255  0.48576  0.95510
     11   2.80554  5.23520  2.84758  1.69834  4.52097  3.49110  2.59546  4.01636  2.43667  3.51562  4.29466  2.96636  3.92344  1.95752  2.88307  2.76892  3.03791  3.61684  5.64482  3.96710     11 e - - -
          2.68618  4.42225  2.77519  2.73123  3.46354  2.40513  3.72494  3.29354  2.67741  2.69355  4.24690  2.90347  2.73739  3.18146  2.89801  2.37887  2.77519  2.98518  4.58477  3.61503
          0.01839  4.40100  5.12334  0.61958  0.77255  0.48576  0.95510
     12   1.78802  4.37479  3.75282  3.59441  4.79177  0.84597  4.68420  4.22369  3.75626  3.92582  4.73108  3.63202  3.93537  3.98668  4.02047  2.47717  2.96737  3.54810  6.12644  4.95274     12 g - - -
          2.68618  4.42225  2.77519  2.73123  3.46354  2.40513  3.72494  3.29354  2.67741  2.69355  4.24690  2.90347  2.73739  3.18146  2.89801  2.37887  2.77519  2.98518  4.58477  3.61503
          0.01839  4.40100  5.12334  0.61958  0.77255  0.48576  0.95510
     13   3.46663  4.71779  5.34467  4.80314  3.46437  4.87905  5.31325  1.51449  4.68423  1.01970  3.23377  4.98836  5.05643  4.77185  4.76404  4.25852  3.70083  1.66297  5.55898  4.48172     13 l - - -
          2.68618  4.42225  2.77519  2.73123  3.46354  2.40513  3.72494  3.29354  2.67741  2.69355  4.24690  2.90347  2.73739  3.18146  2.89801  2.37887  2.77519  2.98518  4.58477  3.61503
          0.01839  4.40100  5.12334  0.61958  0.77255  0.48576  0.95510
     14   3.64604  4.91392  5.37953  4.80121  3.21287  4.95735  5.24075  2.13061  4.65119  0.92804  1.53080  5.03454  5.05396  4.64320  4.68647  4.32243  3.85480  2.55172  5.38250  4.38768     14 l - - -
          2.68618  4.42225  2.77519  2.73123  3.46354  2.40513  3.72494  3.29354  2.67741  2.69355  4.24690  2.90347  2.73739  3.18146  2.89801  2.37887  2.77519  2.98518  4.58477  3.61503
          0.01839  4.40100  5.12334  0.61958  0.77255  0.48576  0.95510
     15   2.68004  4.77516  3.12910  2.58676  3.96290  3.52398  3.34079  3.24848  2.57397  3.02858  3.86404  2.08952  3.91995  2.92341  3.01764  2.42767  2.44921  3.10005  5.28218  2.73856     15 n - - -
          2.68618  4.42225  2.77519  2.73123  3.46354  2.40513  3.72494  3.29354  2.67741  2.69355  4.24690  2.90347  2.73739  3.18146  2.89801  2.37887  2.77519  2.98518  4.58477  3.61503
          0.01839  4.40100  5.12334  0.61958  0.77255  0.48576  0.95510
     16   3.45332  5.14341  4.23085  4.20971  5.28041  0.23980  5.26278  5.04240  4.45988  4.61372  5.61620  4.39801  4.54631  4.74306  4.60648  3.64487  3.97331  4.46803  6.20302  5.41097     16 G - - -
          2.68618  4.42225  2.77519  2.73123  3.46354  2.40513  3.72494  3.29354  2.67741  2.69355  4.24690  2.90347  2.73739  3.18146  2.89801  2.37887  2.77519  2.98518  4.58477  3.61503
          0.01839  4.40100  5.12334  0.61958  0.77255  0.48576  0.95510
     17   2.65245  4.64903  3.23629  2.68330  3.88470  3.20673  3.79571  3.27024  2.66332  2.67252  3.33394  2.69145  3.93379  3.00162  3.09136  1.88781  2.28455  2.88263  5.23479  3.94696     17 s - - -
          2.68618  4.42225  2.77519  2.73123  3.46354  2.40513  3.72494  3.29354  2.67741  2.69355  4.24690  2.90347  2.73739  3.18146  2.89801  2.37887  2.77519  2.98518  4.58477  3.61503
          0.01839  4.40100  5.12334  0.61958  0.77255  0.48576  0.95510
     18   2.28313  4.62741  3.25484  2.69980  3.85971  3.36564  3.80275  3.04309  2.68075  2.90964  3.59798  2.74768  2.30257  3.01514  3.10647  2.46336  2.76867  2.58103  5.21762  3.93388     18 a - - -
          2.68618  4.42225  2.77519  2.73123  3.46354  2.40513  3.72494  3.29354  2.67741  2.69355  4.24690  2.90347  2.73739  3.18146  2.89801  2.37887  2.77519  2.98518  4.58477  3.61503
```

```
          0.01839  4.40100  5.12334  0.61958  0.77255  0.48576  0.95510
     19   3.25272  5.92120  1.34597  1.09452  5.20114  3.43368  4.02634  4.76018  3.10713  4.22290  5.09183  2.88510  4.08606  3.19806  3.75151  3.10428  3.55216  4.30216  6.35861  4.80601     19 e - - -
          2.68618  4.42225  2.77519  2.73123  3.46354  2.40513  3.72494  3.29354  2.67741  2.69355  4.24690  2.90347  2.73739  3.18146  2.89801  2.37887  2.77519  2.98518  4.58477  3.61503
          0.01839  4.40100  5.12334  0.61958  0.77255  0.48576  0.95510
     20   2.58455  4.85215  3.09640  2.21523  4.08290  3.51699  3.72098  3.47389  2.51238  2.75108  3.50293  3.05674  3.90789  1.79573  2.95980  2.72828  2.83549  2.91369  5.36066  4.03600     20 q - - -
          2.68618  4.42225  2.77519  2.73123  3.46354  2.40513  3.72494  3.29354  2.67741  2.69355  4.24690  2.90347  2.73739  3.18146  2.89801  2.37887  2.77519  2.98518  4.58477  3.61503
          0.01839  4.40100  5.12334  0.61958  0.77255  0.48576  0.95510
     21   3.44067  5.51596  3.98317  3.15992  5.08727  3.99428  3.80770  4.37540  1.17317  3.73111  4.61552  3.50693  4.32589  2.93423  1.16469  3.41637  3.56098  4.06143  5.69694  4.60218     21 r - - -
          2.68618  4.42225  2.77519  2.73123  3.46354  2.40513  3.72494  3.29354  2.67741  2.69355  4.24690  2.90347  2.73739  3.18146  2.89801  2.37887  2.77519  2.98518  4.58477  3.61503
          0.01839  4.40100  5.12334  0.61958  0.77255  0.48576  0.95510
     22   2.74590  4.89868  3.03974  1.35559  4.20869  3.51588  3.82250  3.32376  2.62129  3.17429  4.04394  3.10034  3.96183  2.98103  3.06538  2.79476  2.52314  2.72002  5.50335  4.17027     22 e - - -
          2.68618  4.42225  2.77519  2.73123  3.46354  2.40513  3.72494  3.29354  2.67741  2.69355  4.24690  2.90347  2.73739  3.18146  2.89801  2.37887  2.77519  2.98518  4.58477  3.61503
          0.01839  4.40100  5.12334  0.61958  0.77255  0.48576  0.95510
     23   2.42264  5.06775  2.96230  2.02486  4.36742  3.46500  3.53862  3.69358  2.36557  3.22005  4.12206  2.64568  3.85968  2.04083  2.88917  2.62871  2.86191  3.06444  5.52781  4.14834     23 e - - -
          2.68618  4.42225  2.77519  2.73123  3.46354  2.40513  3.72494  3.29354  2.67741  2.69355  4.24690  2.90347  2.73739  3.18146  2.89801  2.37887  2.77519  2.98518  4.58477  3.61503
          0.01839  4.40100  5.12334  0.61958  0.77255  0.48576  0.95510
     24   1.01221  4.35236  3.86406  3.59004  4.66238  3.01224  4.60442  4.08122  3.63767  3.78055  4.58855  3.62803  3.92203  3.89537  3.91735  1.39240  2.93163  3.46023  6.00458  4.82327     24 a - - -
          2.68618  4.42225  2.77519  2.73123  3.46354  2.40513  3.72494  3.29354  2.67741  2.69355  4.24690  2.90347  2.73739  3.18146  2.89801  2.37887  2.77519  2.98518  4.58477  3.61503
          0.01839  4.40100  5.12334  0.61958  0.77255  0.48576  0.95510
     25   0.58106  4.40695  3.92648  3.75462  4.72894  2.68825  4.75731  4.08535  3.83972  3.85366  4.70478  3.74399  3.99542  4.09278  4.06706  2.69867  3.03103  3.49381  6.06944  4.92711     25 A - - -
          2.68618  4.42225  2.77519  2.73123  3.46354  2.40513  3.72494  3.29354  2.67741  2.69355  4.24690  2.90347  2.73739  3.18146  2.89801  2.37887  2.77519  2.98518  4.58477  3.61503
          0.01839  4.40100  5.12334  0.61958  0.77255  0.48576  0.95510
     26   2.72488  4.78660  3.19759  2.39645  3.68695  3.56690  3.75548  3.38804  2.19745  1.79685  3.86412  3.13301  3.95412  2.47614  2.85597  2.79638  2.95120  3.10329  5.29431  4.00080     26 l - - -
          2.68618  4.42225  2.77519  2.73123  3.46354  2.40513  3.72494  3.29354  2.67741  2.69355  4.24690  2.90347  2.73739  3.18146  2.89801  2.37887  2.77519  2.98518  4.58477  3.61503
          0.01839  4.40100  5.12334  0.61958  0.77255  0.48576  0.95510
     27   1.07075  4.35042  3.86607  3.59115  4.64986  1.52214  4.60445  4.03723  3.64291  3.75380  4.56807  3.63026  3.92418  3.89687  3.92275  2.60718  2.55748  3.43438  5.99662  4.82043     27 a - - -
          2.68618  4.42225  2.77519  2.73123  3.46354  2.40513  3.72494  3.29354  2.67741  2.69355  4.24690  2.90347  2.73739  3.18146  2.89801  2.37887  2.77519  2.98518  4.58477  3.61503
          0.01839  4.40100  5.12334  0.61958  0.77255  0.48576  0.95510
     28   3.52149  4.76755  5.38141  4.84440  3.44258  4.93610  5.36296  1.21234  4.71644  1.08798  3.20849  5.04340  5.09553  4.79743  4.79411  4.32362  3.75498  1.95730  5.57406  4.50398     28 l - - -
          2.68618  4.42225  2.77519  2.73123  3.46354  2.40513  3.72494  3.29354  2.67741  2.69355  4.24690  2.90347  2.73739  3.18146  2.89801  2.37887  2.77519  2.98518  4.58477  3.61503
          0.01839  4.40100  5.12334  0.61958  0.77255  0.48576  0.95510
     29   1.47670  4.36531  3.79637  3.52802  4.71438  1.26210  4.58499  4.16001  3.61420  3.83043  4.62406  3.59649  3.91390  3.86071  3.91403  1.90507  2.92974  3.50655  6.03956  4.85442     29 g - - -
          2.68618  4.42225  2.77519  2.73123  3.46354  2.40513  3.72494  3.29354  2.67741  2.69355  4.24690  2.90347  2.73739  3.18146  2.89801  2.37887  2.77519  2.98518  4.58477  3.61503
          0.01839  4.40100  5.12334  0.61958  0.77255  0.48576  0.95510
     30   2.71201  5.19614  1.70819  1.89056  4.50300  3.19531  3.72499  3.96228  2.52527  2.97619  4.26737  2.93264  3.89868  2.84727  3.02651  2.73507  2.79082  3.44826  5.66281  4.25955     30 d - - -
          2.68618  4.42225  2.77519  2.73123  3.46354  2.40513  3.72494  3.29354  2.67741  2.69355  4.24690  2.90347  2.73739  3.18146  2.89801  2.37887  2.77519  2.98518  4.58477  3.61503
          0.01839  4.40100  5.12334  0.61958  0.77255  0.48576  0.95510
     31   3.38771  4.61430  5.35839  4.86089  3.81682  4.91603  5.52089  1.12171  4.77498  2.01922  3.57917  5.03578  5.14177  4.98971  4.92529  4.32052  3.64079  1.08545  5.87142  4.68873     31 v - - -
          2.68618  4.42225  2.77519  2.73123  3.46354  2.40513  3.72494  3.29354  2.67741  2.69355  4.24690  2.90347  2.73739  3.18146  2.89801  2.37887  2.77519  2.98518  4.58477  3.61503
          0.01839  4.40100  5.12334  0.61958  0.77255  0.48576  0.95510
     32   3.38471  4.61883  5.34718  4.87777  3.90222  4.90040  5.59894  0.97806  4.79344  2.34600  3.66021  5.04942  5.16068  5.04619  4.96401  4.32488  3.65006  1.10609  5.97423  4.76321     32 i - - -
          2.68618  4.42225  2.77519  2.73123  3.46354  2.40513  3.72494  3.29354  2.67741  2.69355  4.24690  2.90347  2.73739  3.18146  2.89801  2.37887  2.77519  2.98518  4.58477  3.61503
          0.01839  4.40100  5.12334  0.61958  0.77255  0.48576  0.95510
     33   2.54825  5.15562  2.39725  2.15514  4.48838  3.44868  3.63936  3.96525  2.12476  3.34561  3.98790  2.81937  3.84416  2.69041  2.53626  2.17545  2.84402  3.53977  5.59493  4.19208     33 k - - -
          2.68618  4.42225  2.77519  2.73123  3.46354  2.40513  3.72494  3.29354  2.67741  2.69355  4.24690  2.90347  2.73739  3.18146  2.89801  2.37887  2.77519  2.98518  4.58477  3.61503
          0.01839  4.40100  5.12334  0.61958  0.77255  0.48576  0.95510
     34   2.77360  4.67688  3.42624  2.82903  3.84782  3.65637  3.82216  3.19401  2.10150  1.97708  3.33034  3.27794  4.03033  3.04348  2.04244  2.90170  2.99431  2.69551  5.20027  3.95129     34 l - - -
          2.68618  4.42225  2.77519  2.73123  3.46354  2.40513  3.72494  3.29354  2.67741  2.69355  4.24690  2.90347  2.73739  3.18146  2.89801  2.37887  2.77519  2.98518  4.58477  3.61503
          0.01839  4.40100  5.12334  0.61958  0.77255  0.48576  0.95510
     35   2.07465  4.35558  3.86782  3.56241  4.50544  3.17969  4.54541  3.82653  3.55888  3.59124  4.43576  3.62825  3.94197  3.84611  3.83357  2.27614  0.86918  3.31201  5.87829  4.69099     35 t - - -
          2.68618  4.42225  2.77519  2.73123  3.46354  2.40513  3.72494  3.29354  2.67741  2.69355  4.24690  2.90347  2.73739  3.18146  2.89801  2.37887  2.77519  2.98518  4.58477  3.61503
          0.01839  4.40100  5.12334  0.61958  0.77255  0.48576  0.95510
     36   2.75735  5.10632  2.69391  2.22573  4.48378  3.19915  3.77680  3.94124  2.59492  3.48435  4.27243  2.88189  2.75332  2.91031  3.08901  1.51102  2.90813  3.54256  5.67081  4.28320     36 s - - -
          2.68618  4.42225  2.77519  2.73123  3.46354  2.40513  3.72494  3.29354  2.67741  2.69355  4.24690  2.90347  2.73739  3.18146  2.89801  2.37887  2.77519  2.98518  4.58477  3.61503
          0.01839  4.40100  5.12334  0.61958  0.77255  0.48576  0.95510
     37   1.63909  5.04331  2.72933  2.05156  4.40146  3.44197  3.76967  3.84499  2.58474  3.40755  4.20257  2.98897  2.71929  2.90752  3.07080  2.64128  2.89234  3.46426  5.60955  4.23631     37 a - - -
          2.68618  4.42225  2.77519  2.73123  3.46354  2.40513  3.72494  3.29354  2.67741  2.69355  4.24690  2.90347  2.73739  3.18146  2.89801  2.37887  2.77519  2.98518  4.58477  3.61503
          0.01839  4.40100  5.12334  0.61958  0.77255  0.48576  0.95510
     38   2.17092  5.15652  2.42736  1.98980  4.47962  3.44930  3.65722  3.95248  2.33695  3.45277  4.20602  2.87695  3.85522  2.38182  2.90265  2.55912  2.73962  3.25723  5.60242  4.20070     38 e - - -
          2.68618  4.42225  2.77519  2.73123  3.46354  2.40513  3.72494  3.29354  2.67741  2.69355  4.24690  2.90347  2.73739  3.18146  2.89801  2.37887  2.77519  2.98518  4.58477  3.61503
          0.01839  4.40100  5.12334  0.61958  0.77255  0.48576  0.95510
     39   1.81161  4.85323  3.04479  2.59503  4.36356  3.22601  3.83857  3.79956  2.44788  3.38164  4.18026  2.46452  3.90359  2.98772  3.09713  1.78883  2.89725  3.39997  5.59258  4.25382     39 s - - -
          2.68618  4.42225  2.77519  2.73123  3.46354  2.40513  3.72494  3.29354  2.67741  2.69355  4.24690  2.90347  2.73739  3.18146  2.89801  2.37887  2.77519  2.98518  4.58477  3.61503
          0.01839  4.40100  5.12334  0.61958  0.77255  0.48576  0.95510
     40   3.81140  5.13421  5.04062  4.71305  3.43289  4.65094  5.21080  2.69028  4.48102  0.42068  3.34229  4.94217  5.01442  4.71464  4.55784  4.33555  4.09106  2.85883  5.46885  4.30476     40 L - - -
          2.68618  4.42225  2.77519  2.73123  3.46354  2.40513  3.72494  3.29354  2.67741  2.69355  4.24690  2.90347  2.73739  3.18146  2.89801  2.37887  2.77519  2.98518  4.58477  3.61503
          0.01839  4.40100  5.12334  0.61958  0.77255  0.48576  0.95510
     41   3.26647  5.46575  3.69731  2.96143  4.98398  3.88264  3.76151  4.30737  1.14489  3.68230  4.53132  3.36773  4.22257  2.11011  1.67788  3.23354  3.41251  3.96454  5.68046  4.52314     41 k - - -
          2.68618  4.42225  2.77519  2.73123  3.46354  2.40513  3.72494  3.29354  2.67741  2.69355  4.24690  2.90347  2.73739  3.18146  2.89801  2.37887  2.77519  2.98518  4.58477  3.61503
          0.01839  4.40100  5.12334  0.61958  0.77255  0.48576  0.95510
     42   2.48786  4.91389  2.93157  2.35785  4.33743  3.44037  3.86305  3.75759  2.68738  3.35902  4.18220  3.07955  1.50102  2.88626  3.13232  2.59772  2.81499  3.39450  5.59174  4.25776     42 p - - -
          2.68618  4.42225  2.77519  2.73123  3.46354  2.40513  3.72494  3.29354  2.67741  2.69355  4.24690  2.90347  2.73739  3.18146  2.89801  2.37887  2.77519  2.98518  4.58477  3.61503
          0.01839  4.40100  5.12334  0.61958  0.77255  0.48576  0.95510
     43   2.81594  4.40364  3.87537  3.32275  1.82326  3.81571  3.37532  2.88941  3.24554  2.48978  3.51791  3.62216  4.18827  3.50491  3.52584  1.94180  3.04828  2.56240  4.55646  2.71257     43 f - - -
          2.68618  4.42225  2.77519  2.73123  3.46354  2.40513  3.72494  3.29354  2.67741  2.69355  4.24690  2.90347  2.73739  3.18146  2.89801  2.37887  2.77519  2.98518  4.58477  3.61503
          0.01839  4.40100  5.12334  0.61958  0.77255  0.48576  0.95510
```

```
 44   2.62584  4.51325  4.67193  4.32752  4.00561  4.02391  5.10406  2.12002  4.22650  2.63379  3.85225  4.39462  4.61124  4.53270  4.41532  3.48441  3.37369  0.69047  5.83733  4.61035     44 V - - -
      2.68618  4.42225  2.77519  2.73123  3.46354  2.40513  3.72494  3.29354  2.67741  2.69355  4.24690  2.90347  2.73739  3.18146  2.89801  2.37887  2.77519  2.98518  4.58477  3.61503
      0.01839  4.40100  5.12334  0.61958  0.77255  0.48576  0.95510
 45   2.74722  4.29177  4.10863  3.55920  3.52176  3.80273  4.26433  1.99432  3.46563  2.49857  3.48031  3.80979  4.23400  3.72979  3.71080  2.67157  1.83326  1.66264  5.06920  3.86415     45 v - - -
      2.68618  4.42225  2.77519  2.73123  3.46354  2.40513  3.72494  3.29354  2.67741  2.69355  4.24690  2.90347  2.73739  3.18146  2.89801  2.37887  2.77519  2.98518  4.58477  3.61503
      0.01839  4.40100  5.12334  0.61958  0.77255  0.48576  0.95510
 46   2.61307  5.03528  2.97352  2.43172  4.33147  3.46793  3.11682  3.77847  2.43033  3.32321  4.10085  2.54714  3.38935  1.97905  2.90588  2.37560  2.80804  2.97841  5.51008  4.13826     46 q - - -
      2.68618  4.42225  2.77519  2.73123  3.46354  2.40513  3.72494  3.29354  2.67741  2.69355  4.24690  2.90347  2.73739  3.18146  2.89801  2.37887  2.77519  2.98518  4.58477  3.61503
      0.01839  4.40100  5.12334  0.61958  0.77255  0.48576  0.95510
 47   3.42007  4.66922  5.28880  4.77117  3.65259  4.87706  5.39621  0.79075  4.65459  1.98503  3.13174  4.97151  5.09137  4.84018  4.79396  4.26879  3.66738  1.71472  5.72730  4.58576     47 i - - -
      2.68618  4.42225  2.77519  2.73123  3.46354  2.40513  3.72494  3.29354  2.67741  2.69355  4.24690  2.90347  2.73739  3.18146  2.89801  2.37887  2.77519  2.98518  4.58477  3.61503
      0.01839  4.40100  5.12334  0.61958  0.77255  0.48576  0.95510
 48   2.80511  4.64090  4.01978  3.83823  4.55911  3.45305  4.79896  3.76157  3.79336  3.60314  4.64119  3.92785  4.20837  4.16478  4.00157  3.01513  0.51881  3.38961  5.93985  4.78487     48 T - - -
      2.68618  4.42225  2.77519  2.73123  3.46354  2.40513  3.72494  3.29354  2.67741  2.69355  4.24690  2.90347  2.73739  3.18146  2.89801  2.37887  2.77519  2.98518  4.58477  3.61503
      0.01839  4.40100  5.12334  0.61958  0.77255  0.48576  0.95510
 49   3.45332  5.14341  4.23085  4.20971  5.28041  0.23980  5.26278  5.04240  4.45988  4.61372  5.61620  4.39801  4.54631  4.74306  4.60648  3.64487  3.97331  4.46803  6.20302  5.41097     49 G - - -
      2.68618  4.42225  2.77519  2.73123  3.46354  2.40513  3.72494  3.29354  2.67741  2.69355  4.24690  2.90347  2.73739  3.18146  2.89801  2.37887  2.77519  2.98518  4.58477  3.61503
      0.01839  4.40100  5.12334  0.61958  0.77255  0.48576  0.95510
 50   3.60697  5.23986  4.27311  4.20790  5.10858  3.89943  5.21019  4.83203  4.31876  4.38068  5.47266  4.44862  0.25115  4.68771  4.47441  3.79739  4.09302  4.40828  6.11154  5.24555     50 P - - -
      2.68618  4.42225  2.77519  2.73123  3.46354  2.40513  3.72494  3.29354  2.67741  2.69355  4.24690  2.90347  2.73739  3.18146  2.89801  2.37887  2.77519  2.98518  4.58477  3.61503
      0.01839  4.40100  5.12334  0.61958  0.77255  0.48576  0.95510
 51   3.81140  5.13421  5.04062  4.71305  3.43289  4.65094  5.21080  2.69028  4.48102  0.42068  3.34229  4.94217  5.01442  4.71464  4.55784  4.33555  4.09106  2.85883  5.46885  4.30476     51 L - - -
      2.68618  4.42225  2.77519  2.73123  3.46354  2.40513  3.72494  3.29354  2.67741  2.69355  4.24690  2.90347  2.73739  3.18146  2.89801  2.37887  2.77519  2.98518  4.58477  3.61503
      0.01839  4.40100  5.12334  0.61958  0.77255  0.48576  0.95510
 52   3.47672  4.78909  5.03466  4.69560  3.79465  4.60354  5.35892  0.59052  4.53840  2.30218  3.67846  4.87491  5.01233  4.85074  4.68047  4.17985  3.77710  2.00603  5.77270  4.53987     52 I - - -
      2.68618  4.42225  2.77519  2.73123  3.46354  2.40513  3.72494  3.29354  2.67741  2.69355  4.24690  2.90347  2.73739  3.18146  2.89801  2.37887  2.77519  2.98518  4.58477  3.61503
      0.01839  4.40100  5.12334  0.61958  0.77255  0.48576  0.95510
 53   3.71813  5.45349  4.27179  3.70832  4.95149  4.05914  4.36096  4.56183  2.65019  3.98021  4.99212  4.01352  4.55960  3.57806  0.40629  3.79394  3.96286  4.26823  5.83146  4.79499     53 R - - -
      2.68618  4.42225  2.77519  2.73123  3.46354  2.40513  3.72494  3.29354  2.67741  2.69355  4.24690  2.90347  2.73739  3.18146  2.89801  2.37887  2.77519  2.98518  4.58477  3.61503
      0.01839  4.40100  5.12334  0.61958  0.77255  0.48576  0.95510
 54   3.30236  4.56273  5.22389  4.73683  3.90281  4.76436  5.42762  1.09268  4.64547  2.41386  3.67751  4.90208  5.05625  4.90411  4.81962  4.16743  3.24917  1.04954  5.88304  4.67102     54 v - - -
      2.68618  4.42225  2.77519  2.73123  3.46354  2.40513  3.72494  3.29354  2.67741  2.69355  4.24690  2.90347  2.73739  3.18146  2.89801  2.37887  2.77519  2.98518  4.58477  3.61503
      0.01839  4.40100  5.12334  0.61958  0.77255  0.48576  0.95510
 55   3.16798  4.47746  4.99223  4.44149  3.55006  4.49611  4.93156  1.56134  4.30995  1.40854  3.39731  4.58897  4.78530  4.49424  4.42931  3.59066  3.41105  1.35373  5.39165  4.22611     55 v - - -
      2.68618  4.42225  2.77519  2.73123  3.46354  2.40513  3.72494  3.29354  2.67741  2.69355  4.24690  2.90347  2.73739  3.18146  2.89801  2.37887  2.77519  2.98518  4.58477  3.61503
      0.01839  4.40100  5.12334  0.61958  0.77255  0.48576  0.95510
 56   2.44104  4.39804  3.67958  3.47762  4.76159  0.92786  4.59234  4.21405  3.61753  3.88488  4.68362  3.57769  3.92864  3.86939  3.91414  1.85653  2.57943  3.55033  6.08142  4.88876     56 g - - -
      2.68618  4.42225  2.77519  2.73123  3.46354  2.40513  3.72494  3.29354  2.67741  2.69355  4.24690  2.90347  2.73739  3.18146  2.89801  2.37887  2.77519  2.98518  4.58477  3.61503
      0.01839  4.40100  5.12334  0.61958  0.77255  0.48576  0.95510
 57   3.25928  5.93271  1.24211  1.17613  5.21114  3.43307  4.02997  4.77201  3.11661  4.23370  5.10490  2.88395  4.08801  3.20254  3.76491  3.10887  3.55980  4.31298  6.36965  4.81357     57 e - - -
      2.68618  4.42225  2.77519  2.73123  3.46354  2.40513  3.72494  3.29354  2.67741  2.69355  4.24690  2.90347  2.73739  3.18146  2.89801  2.37887  2.77519  2.98518  4.58477  3.61503
      0.01839  4.40100  5.12334  0.61958  0.77255  0.48576  0.95510
 58   3.46009  5.45543  3.97010  3.22978  4.99040  3.98497  3.87624  4.32691  1.88291  3.72214  4.63091  3.56386  4.35537  3.01784  0.73287  3.46094  3.60515  4.02503  5.69788  4.59985     58 r - - -
      2.68618  4.42225  2.77519  2.73123  3.46354  2.40513  3.72494  3.29354  2.67741  2.69355  4.24690  2.90347  2.73739  3.18146  2.89801  2.37887  2.77519  2.98518  4.58477  3.61503
      0.18149  4.40100  1.87270  0.61958  0.77255  0.48576  0.95510
 59   3.39349  4.73478  4.65785  4.23052  0.95795  4.34060  3.82734  2.94144  4.08007  2.36366  3.61624  4.17164  4.66316  4.14760  4.16572  3.68419  3.62702  2.50552  4.03409  2.14360     59 f - - -
      2.68618  4.42225  2.77519  2.73123  3.46354  2.40513  3.72494  3.29354  2.67741  2.69355  4.24690  2.90347  2.73739  3.18146  2.89801  2.37887  2.77519  2.98518  4.58477  3.61503
      0.02161  4.24112  4.96347  0.61958  0.77255  0.37558  1.16120
 60   2.39833  4.90130  2.99228  2.56533  4.41836  3.39994  3.83387  3.86271  2.55037  3.43207  4.22503  2.28682  2.43803  2.97775  3.09902  1.73575  2.97066  3.45183  5.63111  4.28083     60 s - - -
      2.68618  4.42225  2.77519  2.73123  3.46354  2.40513  3.72494  3.29354  2.67741  2.69355  4.24690  2.90347  2.73739  3.18146  2.89801  2.37887  2.77519  2.98518  4.58477  3.61503
      0.01839  4.40100  5.12334  0.61958  0.77255  0.48576  0.95510
 61   2.38107  4.28371  3.72366  3.15465  3.25758  3.66417  3.96473  2.77490  3.08890  2.50455  3.40841  3.50026  3.38543  3.36913  3.39310  2.32132  2.47901  2.14821  2.73161  3.67653     61 v - - -
      2.68618  4.42225  2.77519  2.73123  3.46354  2.40513  3.72494  3.29354  2.67741  2.69355  4.24690  2.90347  2.73739  3.18146  2.89801  2.37887  2.77519  2.98518  4.58477  3.61503
      0.01839  4.40100  5.12334  0.61958  0.77255  0.48576  0.95510
 62   2.75095  5.26235  2.09639  1.95038  4.59309  2.91864  3.69236  4.08051  2.47056  3.56263  4.31475  2.56643  3.43625  2.58454  2.97690  2.45396  2.67363  3.64684  5.70039  4.27808     62 e - - -
      2.68618  4.42225  2.77519  2.73123  3.46354  2.40513  3.72494  3.29354  2.67741  2.69355  4.24690  2.90347  2.73739  3.18146  2.89801  2.37887  2.77519  2.98518  4.58477  3.61503
      0.01839  4.40100  5.12334  0.61958  0.77255  0.48576  0.95510
 63   3.36578  4.61254  5.29791  4.82990  3.90525  4.85253  5.55457  1.41721  4.73976  2.36461  3.67142  5.00260  5.13147  5.00255  4.91673  4.27557  3.63452  0.77245  5.95883  4.73964     63 v - - -
      2.68618  4.42225  2.77519  2.73123  3.46354  2.40513  3.72494  3.29354  2.67741  2.69355  4.24690  2.90347  2.73739  3.18146  2.89801  2.37887  2.77519  2.98518  4.58477  3.61503
      0.01839  4.40100  5.12334  0.61958  0.77255  0.48576  0.95510
 64   3.56346  5.47109  3.77076  3.33036  4.98569  3.95359  4.18651  4.46086  0.48937  3.92230  4.88467  3.71851  4.44041  3.36882  2.69650  3.58853  3.79043  4.14978  5.83940  4.74900     64 K - - -
      2.68618  4.42225  2.77519  2.73123  3.46354  2.40513  3.72494  3.29354  2.67741  2.69355  4.24690  2.90347  2.73739  3.18146  2.89801  2.37887  2.77519  2.98518  4.58477  3.61503
      0.01839  4.40100  5.12334  0.61958  0.77255  0.48576  0.95510
 65   1.62477  3.14760  3.82808  3.28801  3.62658  3.50881  4.11165  2.92921  3.22153  2.50177  3.60084  3.55778  4.02670  3.49843  3.51957  2.08652  2.59104  2.37020  5.09124  3.88013     65 a - - -
      2.68618  4.42225  2.77519  2.73123  3.46354  2.40513  3.72494  3.29354  2.67741  2.69355  4.24690  2.90347  2.73739  3.18146  2.89801  2.37887  2.77519  2.98518  4.58477  3.61503
      0.01839  4.40100  5.12334  0.61958  0.77255  0.48576  0.95510
 66   0.64187  4.35039  3.92808  3.72776  4.65117  3.14929  4.70576  3.99782  3.77375  3.77514  4.62320  3.69960  3.95210  4.03501  4.00442  2.23498  2.96553  3.41513  6.02850  4.84972     66 A - - -
      2.68618  4.42225  2.77519  2.73123  3.46354  2.40513  3.72494  3.29354  2.67741  2.69355  4.24690  2.90347  2.73739  3.18146  2.89801  2.37887  2.77519  2.98518  4.58477  3.61503
      0.01839  4.40100  5.12334  0.61958  0.77255  0.48576  0.95510
 67   3.40211  4.63526  5.35008  4.83961  3.71194  4.89925  5.45159  1.01523  4.74379  1.74560  3.47862  5.01548  5.11306  4.92205  4.87322  4.29558  3.65018  1.37990  5.77303  4.62173     67 i - - -
      2.68618  4.42225  2.77519  2.73123  3.46354  2.40513  3.72494  3.29354  2.67741  2.69355  4.24690  2.90347  2.73739  3.18146  2.89801  2.37887  2.77519  2.98518  4.58477  3.61503
      0.01839  4.40100  5.12334  0.61958  0.77255  0.48576  0.95510
 68   3.76602  5.03414  5.32904  4.83437  2.60608  4.96314  5.02891  2.48689  4.67566  0.55439  3.04874  5.01459  5.09705  4.67061  4.70998  4.37834  3.98559  2.71249  5.13973  3.84573     68 L - - -
      2.68618  4.42225  2.77519  2.73123  3.46354  2.40513  3.72494  3.29354  2.67741  2.69355  4.24690  2.90347  2.73739  3.18146  2.89801  2.37887  2.77519  2.98518  4.58477  3.61503
      0.01839  4.40100  5.12334  0.61958  0.77255  0.48576  0.95510
 69   2.66465  4.36801  3.44949  2.17173  2.79853  3.65025  3.68534  2.86251  2.95208  2.09565  3.47210  3.39784  4.02893  3.25041  3.29822  2.67084  2.89635  2.48381  4.00768  3.13223     69 l - - -
```

```
        2.68618  4.42225  2.77519  2.73123  3.46354  2.40513  3.72494  3.29354  2.67741  2.69355  4.24690  2.90347  2.73739  3.18146  2.89801  2.37887  2.77519  2.98518  4.58477  3.61503
        0.01839  4.40100  5.12334  0.61958  0.77255  0.48576  0.95510
   70   1.60727  4.34806  3.90762  3.58590  4.47066  3.18756  4.54822  3.75223  3.56877  3.54661  4.40131  3.64246  3.94662  3.85420  3.84064  2.52013  1.01170  3.26176  5.85499  4.66878     70 t - - -
        2.68618  4.42225  2.77519  2.73123  3.46354  2.40513  3.72494  3.29354  2.67741  2.69355  4.24690  2.90347  2.73739  3.18146  2.89801  2.37887  2.77519  2.98518  4.58477  3.61503
        0.01839  4.40100  5.12334  0.61958  0.77255  0.48576  0.95510
   71   3.71050  5.01565  5.23808  4.76072  3.24677  4.89577  5.22978  2.30595  4.51221  0.53313  3.07839  4.99744  5.08348  4.65385  4.59137  4.33969  3.95210  2.56920  5.40677  4.27537     71 L - - -
        2.68618  4.42225  2.77519  2.73123  3.46354  2.40513  3.72494  3.29354  2.67741  2.69355  4.24690  2.90347  2.73739  3.18146  2.89801  2.37887  2.77519  2.98518  4.58477  3.61503
        0.01839  4.40100  5.12334  0.61958  0.77255  0.48576  0.95510
   72   2.02004  4.89455  2.83143  2.55939  4.40591  3.39526  3.83762  3.84732  2.65841  3.42340  4.21779  1.78986  3.90205  2.98337  3.13009  2.31677  2.43167  3.43938  5.62880  4.27639     72 n - - -
        2.68618  4.42225  2.77519  2.73123  3.46354  2.40513  3.72494  3.29354  2.67741  2.69355  4.24690  2.90347  2.73739  3.18146  2.89801  2.37887  2.77519  2.98518  4.58477  3.61503
        0.01839  4.40100  5.12334  0.61958  0.77255  0.48576  0.95510
   73   2.66422  4.39414  3.52614  2.95893  3.53328  3.47043  3.88922  2.13748  2.67407  2.12818  3.29296  2.61188  4.01831  2.97545  3.25997  2.77315  2.89582  2.48150  4.97499  3.73930     73 l - - -
        2.68618  4.42225  2.77519  2.73123  3.46354  2.40513  3.72494  3.29354  2.67741  2.69355  4.24690  2.90347  2.73739  3.18146  2.89801  2.37887  2.77519  2.98518  4.58477  3.61503
        0.01839  4.40100  5.12334  0.61958  0.77255  0.48576  0.95510
   74   3.73490  4.98543  5.45445  4.88374  3.22069  5.06985  5.34678  2.14373  4.72550  0.59862  2.57160  5.14188  5.13069  4.71017  4.76042  4.44975  3.94228  2.55126  5.43778  4.45185     74 L - - -
        2.68618  4.42225  2.77519  2.73123  3.46354  2.40513  3.72494  3.29354  2.67741  2.69355  4.24690  2.90347  2.73739  3.18146  2.89801  2.37887  2.77519  2.98518  4.58477  3.61503
        0.01839  4.40100  5.12334  0.61958  0.77255  0.48576  0.95510
   75   3.68886  4.94270  5.42603  4.86519  2.43673  4.98835  5.14841  2.15327  4.72735  0.65290  2.98847  5.05797  5.07868  4.67712  4.73921  4.35949  3.89658  2.59838  5.25432  4.10574     75 L - - -
        2.68618  4.42225  2.77519  2.73123  3.46354  2.40513  3.72494  3.29354  2.67741  2.69355  4.24690  2.90347  2.73739  3.18146  2.89801  2.37887  2.77519  2.98518  4.58477  3.61503
        0.01839  4.40100  5.12334  0.61958  0.77255  0.48576  0.95510
   76   2.26683  5.07312  2.79004  2.02245  4.37641  3.12517  3.40987  3.33299  2.25816  3.20742  4.12523  2.94540  3.85225  2.76374  2.62662  2.51167  2.73832  3.34703  5.53159  4.14765     76 e - - -
        2.68618  4.42225  2.77519  2.73123  3.46354  2.40513  3.72494  3.29354  2.67741  2.69355  4.24690  2.90347  2.73739  3.18146  2.89801  2.37887  2.77519  2.98518  4.58477  3.61503
        0.01839  4.40100  5.12334  0.61958  0.77255  0.48576  0.95510
   77   3.56346  5.47109  3.77076  3.33036  4.98569  3.95359  4.18651  4.46086  0.48937  3.92230  4.88467  3.71851  4.44041  3.36882  2.69650  3.58853  3.79043  4.14978  5.83940  4.74900     77 K - - -
        2.68618  4.42225  2.77519  2.73123  3.46354  2.40513  3.72494  3.29354  2.67741  2.69355  4.24690  2.90347  2.73739  3.18146  2.89801  2.37887  2.77519  2.98518  4.58477  3.61503
        0.01839  4.40100  5.12334  0.61958  0.77255  0.48576  0.95510
   78   2.94755  4.32094  4.65472  4.09011  3.49206  3.36341  4.58066  1.41481  3.95645  2.32115  2.80468  4.24394  4.52269  4.16834  4.09903  3.50301  3.19196  1.35186  5.15719  3.96821     78 v - - -
        2.68618  4.42225  2.77519  2.73123  3.46354  2.40513  3.72494  3.29354  2.67741  2.69355  4.24690  2.90347  2.73739  3.18146  2.89801  2.37887  2.77519  2.98518  4.58477  3.61503
        0.01839  4.40100  5.12334  0.61958  0.77255  0.48576  0.95510
   79   2.73916  4.89775  2.55072  2.64849  4.66695  1.67422  4.07398  4.13412  3.01094  3.72000  4.53555  3.12592  1.60794  3.25538  3.49100  2.68078  3.11797  3.65358  5.91513  4.56011     79 p - - -
        2.68618  4.42225  2.77519  2.73123  3.46354  2.40513  3.72494  3.29354  2.67741  2.69355  4.24690  2.90347  2.73739  3.18146  2.89801  2.37887  2.77519  2.98518  4.58477  3.61503
        0.01839  4.40100  5.12334  0.61958  0.77255  0.48576  0.95510
   80   2.55738  4.20118  4.07985  3.49886  3.30737  3.80445  4.10689  1.93234  3.39233  2.18681  2.74081  3.74705  4.17084  2.97327  3.60568  3.08726  2.35159  1.98480  4.83449  3.63956     80 i - - -
        2.68618  4.42225  2.77519  2.73123  3.46354  2.40513  3.72494  3.29354  2.67741  2.69355  4.24690  2.90347  2.73739  3.18146  2.89801  2.37887  2.77519  2.98518  4.58477  3.61503
        0.01839  4.40100  5.12334  0.61958  0.77255  0.48576  0.95510
   81   2.06625  4.20327  3.97889  3.40268  2.02490  3.74455  4.04252  2.64008  3.30151  2.24446  2.61693  3.66864  4.12101  3.35905  3.53180  2.75374  2.91122  2.44866  4.80161  3.59289     81 f - - -
        2.68618  4.42225  2.77519  2.73123  3.46354  2.40513  3.72494  3.29354  2.67741  2.69355  4.24690  2.90347  2.73739  3.18146  2.89801  2.37887  2.77519  2.98518  4.58477  3.61503
        0.01839  4.40100  5.12334  0.61958  0.77255  0.48576  0.95510
   82   3.64088  4.93261  5.30221  4.77120  3.28091  4.91935  5.27842  2.22212  4.58291  0.59862  3.06505  5.00989  5.07649  4.66870  4.66263  4.31586  3.87466  2.24615  5.45698  4.39344     82 L - - -
        2.68618  4.42225  2.77519  2.73123  3.46354  2.40513  3.72494  3.29354  2.67741  2.69355  4.24690  2.90347  2.73739  3.18146  2.89801  2.37887  2.77519  2.98518  4.58477  3.61503
        0.01839  4.40100  5.12334  0.61958  0.77255  0.48576  0.95510
   83   3.42117  5.50734  3.94064  3.13854  5.07056  3.97973  3.80722  4.36609  0.94844  3.72650  4.60831  3.49308  4.31558  2.93375  1.47382  3.39698  3.54660  4.04919  5.69642  4.59399     83 k - - -
        2.68618  4.42225  2.77519  2.73123  3.46354  2.40513  3.72494  3.29354  2.67741  2.69355  4.24690  2.90347  2.73739  3.18146  2.89801  2.37887  2.77519  2.98518  4.58477  3.61503
        0.01839  4.40100  5.12334  0.61958  0.77255  0.48576  0.95510
   84   2.98401  5.04684  3.08900  2.86259  4.55100  3.53860  4.12102  4.08468  2.85294  3.63348  4.54103  3.32085  0.99958  2.25726  3.18577  3.04159  3.32338  3.70886  5.77443  4.47903     84 p - - -
        2.68618  4.42225  2.77519  2.73123  3.46354  2.40513  3.72494  3.29354  2.67741  2.69355  4.24690  2.90347  2.73739  3.18146  2.89801  2.37887  2.77519  2.98518  4.58477  3.61503
        0.01839  4.40100  5.12334  0.61958  0.77255  0.48576  0.95510
   85   4.02016  5.27198  4.97864  4.81932  0.43074  4.52395  4.23259  3.51708  4.72358  2.77411  4.18113  4.66399  4.99003  4.75030  4.71058  4.24120  4.33123  3.54194  4.37576  2.69773     85 F - - -
        2.68618  4.42225  2.77519  2.73123  3.46354  2.40513  3.72494  3.29354  2.67741  2.69355  4.24690  2.90347  2.73739  3.18146  2.89801  2.37887  2.77519  2.98518  4.58477  3.61503
        0.01839  4.40100  5.12334  0.61958  0.77255  0.48576  0.95510
   86   3.67549  4.92978  5.40376  4.87111  3.29135  5.01082  5.34682  1.77119  4.69758  0.67424  3.06774  5.10497  5.12692  4.74556  4.75276  4.41361  3.89947  2.36952  5.47884  4.42797     86 L - - -
        2.68618  4.42225  2.77519  2.73123  3.46354  2.40513  3.72494  3.29354  2.67741  2.69355  4.24690  2.90347  2.73739  3.18146  2.89801  2.37887  2.77519  2.98518  4.58477  3.61503
        0.01839  4.40100  5.12334  0.61958  0.77255  0.48576  0.95510
   87   3.60697  5.23986  4.27311  4.20790  5.10858  3.89943  5.21019  4.83203  4.31876  4.38068  5.47266  4.44862  0.25115  4.68771  4.47441  3.79739  4.09302  4.40828  6.11154  5.24555     87 P - - -
        2.68618  4.42225  2.77519  2.73123  3.46354  2.40513  3.72494  3.29354  2.67741  2.69355  4.24690  2.90347  2.73739  3.18146  2.89801  2.37887  2.77519  2.98518  4.58477  3.61503
        0.01839  4.40100  5.12334  0.61958  0.77255  0.48576  0.95510
   88   3.45218  5.37802  3.40010  3.19887  4.61975  3.82039  4.34832  4.37414  2.98900  3.81621  4.86293  3.65063  4.40224  0.52593  3.24715  3.49335  3.76488  4.07854  5.80307  4.55473     88 Q - - -
        2.68618  4.42225  2.77519  2.73123  3.46354  2.40513  3.72494  3.29354  2.67741  2.69355  4.24690  2.90347  2.73739  3.18146  2.89801  2.37887  2.77519  2.98518  4.58477  3.61503
        0.01839  4.40100  5.12334  0.61958  0.77255  0.48576  0.95510
   89   3.81140  5.13421  5.04062  4.71305  3.43289  4.65094  5.21080  2.69028  4.48102  0.42068  3.34229  4.94217  5.01442  4.71464  4.55784  4.33555  4.09106  2.85883  5.46885  4.30476     89 L - - -
        2.68618  4.42225  2.77519  2.73123  3.46354  2.40513  3.72494  3.29354  2.67741  2.69355  4.24690  2.90347  2.73739  3.18146  2.89801  2.37887  2.77519  2.98518  4.58477  3.61503
        0.01839  4.40100  5.12334  0.61958  0.77255  0.48576  0.95510
   90   3.45218  5.37802  3.40010  3.19887  4.61975  3.82039  4.34832  4.37414  2.98900  3.81621  4.86293  3.65063  4.40224  0.52593  3.24715  3.49335  3.76488  4.07854  5.80307  4.55473     90 Q - - -
        2.68618  4.42225  2.77519  2.73123  3.46354  2.40513  3.72494  3.29354  2.67741  2.69355  4.24690  2.90347  2.73739  3.18146  2.89801  2.37887  2.77519  2.98518  4.58477  3.61503
        0.01839  4.40100  5.12334  0.61958  0.77255  0.48576  0.95510
   91   2.76969  4.82827  3.41012  2.87524  4.38453  3.51437  3.90680  3.76960  2.49129  3.36799  4.20907  3.28641  4.01777  3.07558  1.50806  2.60388  1.74320  3.40424  5.56327  4.30819     91 r - - -
        2.68618  4.42225  2.77519  2.73123  3.46354  2.40513  3.72494  3.29354  2.67741  2.69355  4.24690  2.90347  2.73739  3.18146  2.89801  2.37887  2.77519  2.98518  4.58477  3.61503
        0.01839  4.40100  5.12334  0.61958  0.77255  0.48576  0.95510
   92   2.67680  4.49570  3.95001  3.61763  4.11843  3.44959  4.53501  2.91301  3.48230  3.03279  4.11612  3.76786  4.14067  3.86791  3.73273  2.90818  0.79027  2.84880  5.66375  4.41094     92 t - - -
        2.68618  4.42225  2.77519  2.73123  3.46354  2.40513  3.72494  3.29354  2.67741  2.69355  4.24690  2.90347  2.73739  3.18146  2.89801  2.37887  2.77519  2.98518  4.58477  3.61503
        0.01839  4.40100  5.12334  0.61958  0.77255  0.48576  0.95510
   93   4.02016  5.27198  4.97864  4.81932  0.43074  4.52395  4.23259  3.51708  4.72358  2.77411  4.18113  4.66399  4.99003  4.75030  4.71058  4.24120  4.33123  3.54194  4.37576  2.69773     93 F - - -
        2.68618  4.42225  2.77519  2.73123  3.46354  2.40513  3.72494  3.29354  2.67741  2.69355  4.24690  2.90347  2.73739  3.18146  2.89801  2.37887  2.77519  2.98518  4.58477  3.61503
        0.01839  4.40100  5.12334  0.61958  0.77255  0.48576  0.95510
   94   1.90848  4.31048  4.22750  3.66742  3.47924  3.91880  4.33003  2.29992  3.56622  1.73117  3.42004  3.91181  4.31343  3.81755  3.79276  3.22876  2.64303  1.71303  5.07278  3.87691     94 v - - -
        2.68618  4.42225  2.77519  2.73123  3.46354  2.40513  3.72494  3.29354  2.67741  2.69355  4.24690  2.90347  2.73739  3.18146  2.89801  2.37887  2.77519  2.98518  4.58477  3.61503
```

```
        0.01839  4.40100  5.12334  0.61958  0.77255  0.48576  0.95510
     95   3.40251  5.48727  3.87467  3.12104  5.03348  3.95910  3.81934  4.34563  0.80664  3.71980  4.60317  3.48377  4.30830  2.94873  1.79805  3.38223  3.53833  4.02790  5.69745  4.58484     95 k - - -
          2.68618  4.42225  2.77519  2.73123  3.46354  2.40513  3.72494  3.29354  2.67741  2.69355  4.24690  2.90347  2.73739  3.18146  2.89801  2.37887  2.77519  2.98518  4.58477  3.61503
          0.01839  4.40100  5.12334  0.61958  0.77255  0.48576  0.95510
     96   1.32924  2.79786  4.06407  3.65457  4.39968  2.62945  4.53738  3.80417  3.60252  3.50620  4.33449  3.66006  3.91806  3.85741  3.87371  1.38653  2.89015  3.28384  5.78242  4.60654     96 a - - -
          2.68618  4.42225  2.77519  2.73123  3.46354  2.40513  3.72494  3.29354  2.67741  2.69355  4.24690  2.90347  2.73739  3.18146  2.89801  2.37887  2.77519  2.98518  4.58477  3.61503
          0.01839  4.40100  5.12334  0.61958  0.77255  0.48576  0.95510
     97   3.81140  5.13421  5.04062  4.71305  3.43289  4.65094  5.21080  2.69028  4.48102  0.42068  3.34229  4.94217  5.01442  4.71464  4.55784  4.33555  4.09106  2.85883  5.46885  4.30476     97 L - - -
          2.68618  4.42225  2.77519  2.73123  3.46354  2.40513  3.72494  3.29354  2.67741  2.69355  4.24690  2.90347  2.73739  3.18146  2.89801  2.37887  2.77519  2.98518  4.58477  3.61503
          0.01839  4.40100  5.12334  0.61958  0.77255  0.48576  0.95510
     98   2.12973  5.06490  2.93352  2.42919  4.38621  3.13772  3.09073  3.83890  2.45272  3.37579  4.15158  2.67010  3.87485  2.17317  2.92964  2.17605  2.93612  3.45155  5.55509  4.17770     98 a - - -
          2.68618  4.42225  2.77519  2.73123  3.46354  2.40513  3.72494  3.29354  2.67741  2.69355  4.24690  2.90347  2.73739  3.18146  2.89801  2.37887  2.77519  2.98518  4.58477  3.61503
          0.01839  4.40100  5.12334  0.61958  0.77255  0.48576  0.95510
     99   3.35767  5.96035  0.66686  2.12146  5.26211  3.45897  4.12214  4.83789  3.27046  4.32772  5.24690  2.93522  4.14371  3.31448  3.93443  3.20404  3.67855  4.39127  6.43609  4.89819     99 D - - -
          2.68618  4.42225  2.77519  2.73123  3.46354  2.40513  3.72494  3.29354  2.67741  2.69355  4.24690  2.90347  2.73739  3.18146  2.89801  2.37887  2.77519  2.98518  4.58477  3.61503
          0.33491  4.40100  1.30072  0.61958  0.77255  0.48576  0.95510
    100   1.97290  4.30613  3.41893  2.95537  4.03241  3.15725  4.00978  3.37850  2.93481  3.09203  3.94151  3.25797  2.22478  3.25421  3.30278  2.42332  1.96274  2.79929  5.39508  4.15196    100 t - - -
          2.68618  4.42225  2.77519  2.73123  3.46354  2.40513  3.72494  3.29354  2.67741  2.69355  4.24690  2.90347  2.73739  3.18146  2.89801  2.37887  2.77519  2.98518  4.58477  3.61503
          0.02515  4.09124  4.81358  0.61958  0.77255  0.31798  1.30056
    101   2.45236  4.56699  3.37229  2.94703  4.43926  2.89386  4.11420  3.87398  2.99002  3.49664  4.29969  3.02432  3.23941  3.30584  3.40188  1.26154  2.05067  3.39249  5.72244  4.44348    101 s - - -
          2.68618  4.42225  2.77519  2.73123  3.46354  2.40513  3.72494  3.29354  2.67741  2.69355  4.24690  2.90347  2.73739  3.18146  2.89801  2.37887  2.77519  2.98518  4.58477  3.61503
          0.01839  4.40100  5.12334  0.61958  0.77255  0.48576  0.95510
    102   2.62960  4.73177  3.09372  2.79850  4.57609  3.30387  4.08674  4.03211  2.95073  3.63604  4.44218  1.64076  3.93983  3.26760  3.37600  1.42854  2.62237  3.53995  5.82636  4.50672    102 s - - -
          2.68618  4.42225  2.77519  2.73123  3.46354  2.40513  3.72494  3.29354  2.67741  2.69355  4.24690  2.90347  2.73739  3.18146  2.89801  2.37887  2.77519  2.98518  4.58477  3.61503
          0.01839  4.40100  5.12334  0.61958  0.77255  0.48576  0.95510
    103   2.88493  5.37190  2.29375  1.62439  4.70400  3.47642  3.76761  4.18215  2.52333  3.66306  4.44422  2.94381  3.95249  2.89024  1.73509  2.82650  3.12653  3.76260  5.79451  4.38534    103 e - - -
          2.68618  4.42225  2.77519  2.73123  3.46354  2.40513  3.72494  3.29354  2.67741  2.69355  4.24690  2.90347  2.73739  3.18146  2.89801  2.37887  2.77519  2.98518  4.58477  3.61503
          0.01839  4.40100  5.12334  0.61958  0.77255  0.48576  0.95510
    104   2.44202  4.56818  3.22879  2.73984  3.73775  3.39349  3.80390  2.88331  2.49010  2.54267  3.49616  3.20739  3.80720  2.72544  3.12654  2.67409  2.21103  2.37329  5.12382  3.85351    104 t - - -
          2.68618  4.42225  2.77519  2.73123  3.46354  2.40513  3.72494  3.29354  2.67741  2.69355  4.24690  2.90347  2.73739  3.18146  2.89801  2.37887  2.77519  2.98518  4.58477  3.61503
          0.01839  4.40100  5.12334  0.61958  0.77255  0.48576  0.95510
    105   3.39708  4.65704  5.26636  4.73297  3.54971  4.81217  5.27889  1.77895  4.61688  1.22967  3.33117  4.91442  5.02515  4.75600  4.72555  4.18993  3.63805  1.18298  5.59453  4.47735    105 v - - -
          2.68618  4.42225  2.77519  2.73123  3.46354  2.40513  3.72494  3.29354  2.67741  2.69355  4.24690  2.90347  2.73739  3.18146  2.89801  2.37887  2.77519  2.98518  4.58477  3.61503
          0.01839  4.40100  5.12334  0.61958  0.77255  0.48576  0.95510
    106   3.71813  5.45349  4.27179  3.70832  4.95149  4.05914  4.36096  4.56183  2.65019  3.98021  4.99212  4.01352  4.55960  3.57806  0.40629  3.79394  3.96286  4.26823  5.83146  4.79499    106 R - - -
          2.68618  4.42225  2.77519  2.73123  3.46354  2.40513  3.72494  3.29354  2.67741  2.69355  4.24690  2.90347  2.73739  3.18146  2.89801  2.37887  2.77519  2.98518  4.58477  3.61503
          0.01839  4.40100  5.12334  0.61958  0.77255  0.48576  0.95510
    107   2.44262  4.35442  3.58734  3.01967  3.49110  3.54653  3.91285  2.66581  2.80574  1.80661  3.11684  3.20368  4.03094  3.26430  3.30404  2.36018  2.60025  2.62449  4.94397  3.71514    107 l - - -
          2.68618  4.42225  2.77519  2.73123  3.46354  2.40513  3.72494  3.29354  2.67741  2.69355  4.24690  2.90347  2.73739  3.18146  2.89801  2.37887  2.77519  2.98518  4.58477  3.61503
          0.01839  4.40100  5.12334  0.61958  0.77255  0.48576  0.95510
    108   2.95558  5.20373  3.33142  2.70946  4.56837  3.37031  3.72818  3.96711  1.65688  3.45834  4.27637  3.18330  4.05197  2.85429  1.43710  2.79256  3.15378  3.61851  5.56789  3.58751    108 r - - -
          2.68618  4.42225  2.77519  2.73123  3.46354  2.40513  3.72494  3.29354  2.67741  2.69355  4.24690  2.90347  2.73739  3.18146  2.89801  2.37887  2.77519  2.98518  4.58477  3.61503
          0.01839  4.40100  5.12334  0.61958  0.77255  0.48576  0.95510
    109   0.61929  4.35533  3.92972  3.74026  4.64834  3.15489  4.71707  3.97898  3.78606  3.77168  4.62884  3.70852  3.95917  4.05069  4.01125  2.33810  2.97432  3.40620  6.03107  4.84999    109 A - - -
          2.68618  4.42225  2.77519  2.73123  3.46354  2.40513  3.72494  3.29354  2.67741  2.69355  4.24690  2.90347  2.73739  3.18146  2.89801  2.37887  2.77519  2.98518  4.58477  3.61503
          0.01839  4.40100  5.12334  0.61958  0.77255  0.48576  0.95510
    110   0.91413  4.33969  3.89369  3.52786  4.42543  2.23446  4.49219  3.79139  3.52198  3.51981  4.35437  3.60953  3.92823  3.79089  3.81581  2.39931  2.91310  2.77800  5.80045  4.60867    110 a - - -
          2.68618  4.42225  2.77519  2.73123  3.46354  2.40513  3.72494  3.29354  2.67741  2.69355  4.24690  2.90347  2.73739  3.18146  2.89801  2.37887  2.77519  2.98518  4.58477  3.61503
          0.01839  4.40100  5.12334  0.61958  0.77255  0.48576  0.95510
    111   2.61380  5.09385  2.72726  2.23441  4.40461  3.19092  3.40691  3.86652  1.84295  3.21988  4.14392  2.93932  3.85005  2.64591  2.78521  2.59185  2.72373  2.93851  5.54718  3.80026    111 k - - -
          2.68618  4.42225  2.77519  2.73123  3.46354  2.40513  3.72494  3.29354  2.67741  2.69355  4.24690  2.90347  2.73739  3.18146  2.89801  2.37887  2.77519  2.98518  4.58477  3.61503
          0.01839  4.40100  5.12334  0.61958  0.77255  0.48576  0.95510
    112   0.67128  4.34304  3.87989  3.67266  4.69712  2.57017  4.68221  4.10463  3.75688  3.82383  4.63804  3.66553  3.93185  3.99754  4.00606  2.42664  2.94615  3.47223  6.05013  4.88368    112 A - - -
          2.68618  4.42225  2.77519  2.73123  3.46354  2.40513  3.72494  3.29354  2.67741  2.69355  4.24690  2.90347  2.73739  3.18146  2.89801  2.37887  2.77519  2.98518  4.58477  3.61503
          0.01839  4.40100  5.12334  0.61958  0.77255  0.48576  0.95510
    113   3.70078  4.96323  5.38109  4.85466  3.26979  5.00743  5.33029  1.96345  4.66583  0.60932  3.05341  5.09656  5.12625  4.72494  4.72497  4.41592  3.92541  2.43564  5.46126  4.40175    113 L - - -
          2.68618  4.42225  2.77519  2.73123  3.46354  2.40513  3.72494  3.29354  2.67741  2.69355  4.24690  2.90347  2.73739  3.18146  2.89801  2.37887  2.77519  2.98518  4.58477  3.61503
          0.01839  4.40100  5.12334  0.61958  0.77255  0.48576  0.95510
    114   2.24578  4.39446  3.68192  3.49664  4.79689  0.97819  4.61976  4.25840  3.66316  3.92694  4.72099  3.58535  3.92748  3.89984  3.95785  1.67222  2.96295  3.57213  6.11529  4.92600    114 g - - -
          2.68618  4.42225  2.77519  2.73123  3.46354  2.40513  3.72494  3.29354  2.67741  2.69355  4.24690  2.90347  2.73739  3.18146  2.89801  2.37887  2.77519  2.98518  4.58477  3.61503
          0.01839  4.40100  5.12334  0.61958  0.77255  0.48576  0.95510
    115   2.46730  4.97060  3.01240  2.25830  4.23935  3.47897  3.49620  3.12684  2.24362  3.24220  3.72742  2.80485  3.87003  2.60717  2.75355  2.59574  2.19781  3.31958  5.45139  3.86317    115 t - - -
          2.68618  4.42225  2.77519  2.73123  3.46354  2.40513  3.72494  3.29354  2.67741  2.69355  4.24690  2.90347  2.73739  3.18146  2.89801  2.37887  2.77519  2.98518  4.58477  3.61503
          0.01839  4.40100  5.12334  0.61958  0.77255  0.48576  0.95510
    116   3.64226  4.99633  5.10499  4.67589  3.27956  4.73635  5.17510  2.44916  4.42727  0.52998  3.13172  4.89086  5.01194  4.61212  4.51879  4.20738  3.91064  2.52090  5.40820  4.25244    116 L - - -
          2.68618  4.42225  2.77519  2.73123  3.46354  2.40513  3.72494  3.29354  2.67741  2.69355  4.24690  2.90347  2.73739  3.18146  2.89801  2.37887  2.77519  2.98518  4.58477  3.61503
          0.01839  4.40100  5.12334  0.61958  0.77255  0.48576  0.95510
    117   2.84510  4.32849  4.39716  3.88683  3.59835  3.66106  4.50935  1.06722  3.76344  2.49121  3.53917  4.05814  4.38901  4.02493  3.95566  2.85572  3.13423  1.82356  5.21616  4.00977    117 i - - -
          2.68618  4.42225  2.77519  2.73123  3.46354  2.40513  3.72494  3.29354  2.67741  2.69355  4.24690  2.90347  2.73739  3.18146  2.89801  2.37887  2.77519  2.98518  4.58477  3.61503
          0.01839  4.40100  5.12334  0.61958  0.77255  0.48576  0.95510
    118   2.55411  4.82580  3.09486  2.28965  4.05651  3.15083  3.71681  3.14406  2.15241  2.99901  3.90360  3.05196  3.89827  2.87675  2.98203  2.52967  2.18428  2.74503  5.33970  4.01496    118 k - - -
          2.68618  4.42225  2.77519  2.73123  3.46354  2.40513  3.72494  3.29354  2.67741  2.69355  4.24690  2.90347  2.73739  3.18146  2.89801  2.37887  2.77519  2.98518  4.58477  3.61503
          0.01839  4.40100  5.12334  0.61958  0.77255  0.48576  0.95510
    119   2.79843  4.21575  4.41392  3.83367  2.55832  3.91896  3.19266  1.71375  3.67904  1.66645  3.25252  3.94490  4.27235  3.85080  3.79128  3.21987  3.02881  2.43172  4.60461  2.88506    119 l - - -
          2.68618  4.42225  2.77519  2.73123  3.46354  2.40513  3.72494  3.29354  2.67741  2.69355  4.24690  2.90347  2.73739  3.18146  2.89801  2.37887  2.77519  2.98518  4.58477  3.61503
          0.01839  4.40100  5.12334  0.61958  0.77255  0.48576  0.95510
```

```
120   2.75562  5.05445  2.97310  2.49300  4.35307  3.50026  2.16508  3.79917  2.45188  3.35153  4.15003  2.87159  3.92170  2.10320  2.88328  2.64447  2.28022  3.43703  5.53450  4.18089    120 q - - -
      2.68618  4.42225  2.77519  2.73123  3.46354  2.40513  3.72494  3.29354  2.67741  2.69355  4.24690  2.90347  2.73739  3.18146  2.89801  2.37887  2.77519  2.98518  4.58477  3.61503
      0.01839  4.40100  5.12334  0.61958  0.77255  0.48576  0.95510
121   2.35724  4.49747  3.47850  2.96115  4.01583  3.39121  4.01930  3.15950  2.93310  3.07284  3.92769  3.33260  1.87840  3.25719  3.32302  2.33893  1.93759  2.88221  5.38574  4.12893    121 p - - -
      2.68618  4.42225  2.77519  2.73123  3.46354  2.40513  3.72494  3.29354  2.67741  2.69355  4.24690  2.90347  2.73739  3.18146  2.89801  2.37887  2.77519  2.98518  4.58477  3.61503
      0.01839  4.40100  5.12334  0.61958  0.77255  0.48576  0.95510
122   3.45910  5.50629  4.00267  3.18856  5.07202  4.00202  3.82252  4.36479  1.51967  3.72868  4.62059  3.52772  4.33959  2.95262  0.89642  3.44066  3.58086  4.05692  5.69628  4.60614    122 r - - -
      2.68618  4.42225  2.77519  2.73123  3.46354  2.40513  3.72494  3.29354  2.67741  2.69355  4.24690  2.90347  2.73739  3.18146  2.89801  2.37887  2.77519  2.98518  4.58477  3.61503
      0.01839  4.40100  5.12334  0.61958  0.77255  0.48576  0.95510
123   2.87188  3.86490  4.43358  3.86738  3.47567  4.04166  4.43787  1.99785  3.74970  2.20574  3.41504  4.06708  2.79910  3.98177  3.93447  3.35603  3.12010  1.18579  5.09419  3.89872    123 v - - -
      2.68618  4.42225  2.77519  2.73123  3.46354  2.40513  3.72494  3.29354  2.67741  2.69355  4.24690  2.90347  2.73739  3.18146  2.89801  2.37887  2.77519  2.98518  4.58477  3.61503
      0.01839  4.40100  5.12334  0.61958  0.77255  0.48576  0.95510
124   2.75648  5.78225  0.81506  2.11747  5.11671  3.42260  4.03221  4.62309  3.11266  4.14011  5.01515  2.89639  4.07827  3.20882  3.74357  3.08241  3.51691  4.18506  6.31180  4.77650    124 d - - -
      2.68618  4.42225  2.77519  2.73123  3.46354  2.40513  3.72494  3.29354  2.67741  2.69355  4.24690  2.90347  2.73739  3.18146  2.89801  2.37887  2.77519  2.98518  4.58477  3.61503
      0.01839  4.40100  5.12334  0.61958  0.77255  0.48576  0.95510
125   2.59855  4.54550  3.53540  3.38046  4.66678  3.25349  4.54333  4.18042  3.51423  3.84919  4.70793  3.57861  0.79562  3.83170  3.80519  2.19627  3.09968  3.60460  5.98840  4.75221    125 p - - -
      2.68618  4.42225  2.77519  2.73123  3.46354  2.40513  3.72494  3.29354  2.67741  2.69355  4.24690  2.90347  2.73739  3.18146  2.89801  2.37887  2.77519  2.98518  4.58477  3.61503
      0.01839  4.40100  5.12334  0.61958  0.77255  0.48576  0.95510
126   3.62700  4.88063  5.40581  4.85862  3.31958  4.99039  5.35009  1.75521  4.71108  0.71595  3.08283  5.08579  5.11058  4.74770  4.76907  4.37820  3.85135  2.20129  5.49906  4.46620    126 l - - -
      2.68618  4.42225  2.77519  2.73123  3.46354  2.40513  3.72494  3.29354  2.67741  2.69355  4.24690  2.90347  2.73739  3.18146  2.89801  2.37887  2.77519  2.98518  4.58477  3.61503
      0.01839  4.40100  5.12334  0.61958  0.77255  0.48576  0.95510
127   2.75503  4.37955  4.86507  4.30090  1.98344  4.31109  4.69991  1.50691  4.15974  2.17343  3.35179  4.41312  4.62817  4.32802  4.25818  3.64593  3.28435  1.51671  5.17715  3.98458    127 i - - -
      2.68618  4.42225  2.77519  2.73123  3.46354  2.40513  3.72494  3.29354  2.67741  2.69355  4.24690  2.90347  2.73739  3.18146  2.89801  2.37887  2.77519  2.98518  4.58477  3.61503
      0.01839  4.40100  5.12334  0.61958  0.77255  0.48576  0.95510
128   2.06813  4.53358  3.35216  2.79828  3.74520  3.55375  3.84653  2.66632  2.77051  2.80438  3.67378  2.91425  3.96549  2.97705  3.17258  2.42627  2.08916  2.65301  5.13876  3.87542    128 a - - -
      2.68618  4.42225  2.77519  2.73123  3.46354  2.40513  3.72494  3.29354  2.67741  2.69355  4.24690  2.90347  2.73739  3.18146  2.89801  2.37887  2.77519  2.98518  4.58477  3.61503
      0.01839  4.40100  5.12334  0.61958  0.77255  0.48576  0.95510
129   3.24484  5.83416  2.03498  0.77242  5.13390  3.45364  4.03828  4.67860  3.08911  4.16528  5.03762  2.92213  4.09770  3.21227  3.69886  3.11273  3.54337  4.23902  6.29109  4.77872    129 e - - -
      2.68618  4.42225  2.77519  2.73123  3.46354  2.40513  3.72494  3.29354  2.67741  2.69355  4.24690  2.90347  2.73739  3.18146  2.89801  2.37887  2.77519  2.98518  4.58477  3.61503
      0.01839  4.40100  5.12334  0.61958  0.77255  0.48576  0.95510
130   3.25199  4.67611  4.62839  4.04980  3.24758  4.39612  4.65779  2.26993  3.86114  0.80640  2.83390  4.33253  4.65244  3.21745  4.03579  3.70877  3.47222  2.57890  5.17518  4.07263    130 l - - -
      2.68618  4.42225  2.77519  2.73123  3.46354  2.40513  3.72494  3.29354  2.67741  2.69355  4.24690  2.90347  2.73739  3.18146  2.89801  2.37887  2.77519  2.98518  4.58477  3.61503
      0.01839  4.40100  5.12334  0.61958  0.77255  0.48576  0.95510
131   2.81483  4.28867  4.20903  3.63333  3.31570  3.92804  2.76748  2.23759  3.49714  1.81551  3.31134  3.87571  4.28404  3.75048  3.70121  3.22073  3.04882  1.47994  4.90849  3.69137    131 v - - -
      2.68618  4.42225  2.77519  2.73123  3.46354  2.40513  3.72494  3.29354  2.67741  2.69355  4.24690  2.90347  2.73739  3.18146  2.89801  2.37887  2.77519  2.98518  4.58477  3.61503
      0.01839  4.40100  5.12334  0.61958  0.77255  0.48576  0.95510
132   2.44079  4.87406  3.04278  2.56824  4.31441  3.42715  3.80050  3.74456  2.43563  3.32946  4.13054  2.25182  3.90157  2.94751  3.04846  2.09369  1.91291  3.36438  5.54512  4.20557    132 t - - -
      2.68618  4.42225  2.77519  2.73123  3.46354  2.40513  3.72494  3.29354  2.67741  2.69355  4.24690  2.90347  2.73739  3.18146  2.89801  2.37887  2.77519  2.98518  4.58477  3.61503
      0.01839  4.40100  5.12334  0.61958  0.77255  0.48576  0.95510
133   2.26983  4.52842  3.40215  3.01108  4.29346  1.29585  4.14552  3.70209  3.05560  2.83041  4.19832  3.11509  3.93543  3.37196  3.44033  2.14009  2.94709  3.27914  5.62712  4.36518    133 g - - -
      2.68618  4.42225  2.77519  2.73123  3.46354  2.40513  3.72494  3.29354  2.67741  2.69355  4.24690  2.90347  2.73739  3.18146  2.89801  2.37887  2.77519  2.98518  4.58477  3.61503
      0.01839  4.40100  5.12334  0.61958  0.77255  0.48576  0.95510
134   2.08462  4.27337  3.98157  3.41680  3.43776  3.77534  4.13861  1.90962  3.33105  2.15503  3.40957  3.70420  4.17383  3.59522  3.59098  2.16140  2.96942  2.16736  4.96230  3.75638    134 i - - -
      2.68618  4.42225  2.77519  2.73123  3.46354  2.40513  3.72494  3.29354  2.67741  2.69355  4.24690  2.90347  2.73739  3.18146  2.89801  2.37887  2.77519  2.98518  4.58477  3.61503
      0.01839  4.40100  5.12334  0.61958  0.77255  0.48576  0.95510
135   3.12462  5.34641  3.55215  2.85536  4.79183  3.79787  3.74488  4.14703  1.20361  3.57820  4.41313  3.29118  4.14867  2.55914  1.66794  3.10548  3.29472  3.26156  5.62947  4.42590    135 k - - -
      2.68618  4.42225  2.77519  2.73123  3.46354  2.40513  3.72494  3.29354  2.67741  2.69355  4.24690  2.90347  2.73739  3.18146  2.89801  2.37887  2.77519  2.98518  4.58477  3.61503
      0.30222  4.40100  1.39206  0.61958  0.77255  0.48576  0.95510
136   2.42777  4.30879  3.03359  2.48952  4.01543  3.40273  3.66739  3.23078  2.47471  3.04157  3.86196  2.38618  3.82007  2.45998  2.92429  2.25570  2.64318  3.09992  5.29505  3.97321    136 s - - -
      2.68618  4.42225  2.77519  2.73123  3.46354  2.40513  3.72494  3.29354  2.67741  2.69355  4.24690  2.90347  2.73739  3.18146  2.89801  2.37887  2.77519  2.98518  4.58477  3.61503
      0.02435  4.12313  4.84547  0.61958  0.77255  0.32805  1.27415
137   2.14130  4.71092  3.18724  2.63745  3.99714  2.94377  3.78336  3.24124  2.62453  3.03655  3.74113  2.94306  3.91333  2.86137  3.06581  2.17349  2.10621  3.02426  5.31498  4.01173    137 t - - -
      2.68618  4.42225  2.77519  2.73123  3.46354  2.40513  3.72494  3.29354  2.67741  2.69355  4.24690  2.90347  2.73739  3.18146  2.89801  2.37887  2.77519  2.98518  4.58477  3.61503
      0.01839  4.40100  5.12334  0.61958  0.77255  0.48576  0.95510
138   2.64610  5.20830  2.22191  1.96668  4.53868  3.35011  3.69303  4.01641  2.47051  3.51469  4.27313  2.80166  3.31474  2.80426  2.97178  2.19663  2.37268  3.59522  5.66458  4.25344    138 e - - -
      2.68618  4.42225  2.77519  2.73123  3.46354  2.40513  3.72494  3.29354  2.67741  2.69355  4.24690  2.90347  2.73739  3.18146  2.89801  2.37887  2.77519  2.98518  4.58477  3.61503
      0.01839  4.40100  5.12334  0.61958  0.77255  0.48576  0.95510
139   3.27172  5.89833  0.74236  2.11523  5.20648  3.42155  4.06114  4.76207  3.18428  4.25158  5.14381  2.88735  4.09756  3.24466  3.84237  2.92091  3.58920  4.30803  6.40394  4.83669    139 d - - -
      2.68618  4.42225  2.77519  2.73123  3.46354  2.40513  3.72494  3.29354  2.67741  2.69355  4.24690  2.90347  2.73739  3.18146  2.89801  2.37887  2.77519  2.98518  4.58477  3.61503
      0.01839  4.40100  5.12334  0.61958  0.77255  0.48576  0.95510
140   2.28492  5.06222  2.84544  2.16931  4.37203  3.10701  3.67996  3.82490  2.37934  3.24999  4.13657  2.95697  2.72598  2.79885  2.93258  2.05779  2.86719  3.43879  5.54398  4.16394    140 s - - -
      2.68618  4.42225  2.77519  2.73123  3.46354  2.40513  3.72494  3.29354  2.67741  2.69355  4.24690  2.90347  2.73739  3.18146  2.89801  2.37887  2.77519  2.98518  4.58477  3.61503
      0.01839  4.40100  5.12334  0.61958  0.77255  0.48576  0.95510
141   2.11616  4.43661  3.60212  3.25894  4.58847  1.15239  4.37494  4.03059  3.33010  3.67636  4.47541  3.31621  3.54967  3.60972  3.68919  2.02796  2.51679  3.45586  5.89741  4.66931    141 g - - -
      2.68618  4.42225  2.77519  2.73123  3.46354  2.40513  3.72494  3.29354  2.67741  2.69355  4.24690  2.90347  2.73739  3.18146  2.89801  2.37887  2.77519  2.98518  4.58477  3.61503
      0.01839  4.40100  5.12334  0.61958  0.77255  0.48576  0.95510
142   3.35425  4.60126  5.25647  4.74322  3.76423  4.83428  5.38726  1.59833  4.64710  2.07228  3.32458  4.92999  5.07190  4.86101  4.80274  4.22236  3.60467  0.80462  5.77909  4.60269    142 v - - -
      2.68618  4.42225  2.77519  2.73123  3.46354  2.40513  3.72494  3.29354  2.67741  2.69355  4.24690  2.90347  2.73739  3.18146  2.89801  2.37887  2.77519  2.98518  4.58477  3.61503
      0.01839  4.40100  5.12334  0.61958  0.77255  0.48576  0.95510
143   3.18273  5.33342  3.67634  2.94410  4.77942  3.84635  3.76532  4.11136  1.44928  3.09481  4.41168  3.35478  4.19213  2.89296  1.25906  3.17590  3.09949  3.78857  5.61559  4.43854    143 r - - -
      2.68618  4.42225  2.77519  2.73123  3.46354  2.40513  3.72494  3.29354  2.67741  2.69355  4.24690  2.90347  2.73739  3.18146  2.89801  2.37887  2.77519  2.98518  4.58477  3.61503
      0.01839  4.40100  5.12334  0.61958  0.77255  0.48576  0.95510
144   2.56933  5.23276  2.55765  1.67475  4.55621  3.44576  3.69631  4.03515  2.28394  3.53021  4.28935  2.56651  3.88131  2.80784  2.97458  2.63628  2.31707  3.61366  5.67787  4.26430    144 e - - -
      2.68618  4.42225  2.77519  2.73123  3.46354  2.40513  3.72494  3.29354  2.67741  2.69355  4.24690  2.90347  2.73739  3.18146  2.89801  2.37887  2.77519  2.98518  4.58477  3.61503
      0.01839  4.40100  5.12334  0.61958  0.77255  0.48576  0.95510
145   1.15237  4.34477  3.89772  3.54412  4.46329  3.17953  4.51167  3.80632  3.52975  3.55127  4.38825  3.61751  3.93161  3.80784  3.81772  2.32766  1.48071  3.29590  5.83504  4.64599    145 a - - -
```

```
        2.68618  4.42225  2.77519  2.73123  3.46354  2.40513  3.72494  3.29354  2.67741  2.69355  4.24690  2.90347  2.73739  3.18146  2.89801  2.37887  2.77519  2.98518  4.58477  3.61503
        0.01839  4.40100  5.12334  0.61958  0.77255  0.48576  0.95510
    146   3.15467  4.58416  4.62251  4.06820  3.37677  4.30166  4.68614  2.13324  3.89229  1.86586  1.15287  4.31214  4.62430  4.13572  4.07208  3.63699  2.98725  2.30967  5.24894  4.11201    146 m - - -
        2.68618  4.42225  2.77519  2.73123  3.46354  2.40513  3.72494  3.29354  2.67741  2.69355  4.24690  2.90347  2.73739  3.18146  2.89801  2.37887  2.77519  2.98518  4.58477  3.61503
        0.01839  4.40100  5.12334  0.61958  0.77255  0.48576  0.95510
    147   3.76218  5.02417  5.39901  4.84989  2.86206  5.04070  5.20485  2.42611  4.69086  0.54436  2.81822  5.08745  5.11844  4.67538  4.72936  4.42401  3.97110  2.66451  5.30170  4.16850    147 L - - -
        2.68618  4.42225  2.77519  2.73123  3.46354  2.40513  3.72494  3.29354  2.67741  2.69355  4.24690  2.90347  2.73739  3.18146  2.89801  2.37887  2.77519  2.98518  4.58477  3.61503
        0.01839  4.40100  5.12334  0.61958  0.77255  0.48576  0.95510
    148   2.53243  5.09552  2.98304  2.42463  4.40815  3.47591  3.65664  3.86265  1.90747  3.38159  4.14820  2.69398  3.86748  2.09435  2.63837  2.54978  2.81084  3.19019  5.54380  3.88260    148 k - - -
        2.68618  4.42225  2.77519  2.73123  3.46354  2.40513  3.72494  3.29354  2.67741  2.69355  4.24690  2.90347  2.73739  3.18146  2.89801  2.37887  2.77519  2.98518  4.58477  3.61503
        0.01839  4.40100  5.12334  0.61958  0.77255  0.48576  0.95510
    149   0.68442  4.34009  3.88549  3.66733  4.69126  2.64104  4.67346  4.10523  3.74579  3.81699  4.62843  3.66174  3.92836  3.98630  3.99847  2.31610  2.94083  3.47126  6.04299  4.87635    149 A - - -
        2.68618  4.42225  2.77519  2.73123  3.46354  2.40513  3.72494  3.29354  2.67741  2.69355  4.24690  2.90347  2.73739  3.18146  2.89801  2.37887  2.77519  2.98518  4.58477  3.61503
        0.01839  4.40100  5.12334  0.61958  0.77255  0.48576  0.95510
    150   3.70245  4.95994  5.42082  4.84947  3.23326  5.03516  5.32712  2.19118  4.70161  0.60568  2.62840  5.10568  5.11082  4.69398  4.74519  4.41047  3.91230  2.42521  5.43996  4.45384    150 L - - -
        2.68618  4.42225  2.77519  2.73123  3.46354  2.40513  3.72494  3.29354  2.67741  2.69355  4.24690  2.90347  2.73739  3.18146  2.89801  2.37887  2.77519  2.98518  4.58477  3.61503
        0.01839  4.40100  5.12334  0.61958  0.77255  0.48576  0.95510
    151   2.71148  4.53157  3.44210  2.87096  2.76489  3.63908  3.24361  3.06018  2.55715  2.27888  3.62853  3.30585  4.01811  3.13019  2.07950  2.87767  2.94013  2.82121  4.99108  2.92431    151 r - - -
        2.68618  4.42225  2.77519  2.73123  3.46354  2.40513  3.72494  3.29354  2.67741  2.69355  4.24690  2.90347  2.73739  3.18146  2.89801  2.37887  2.77519  2.98518  4.58477  3.61503
        0.01839  4.40100  5.12334  0.61958  0.77255  0.48576  0.95510
    152   2.35190  4.93664  2.84292  1.84279  3.07951  2.77015  3.69587  3.62052  2.48623  3.02834  4.00678  3.00183  3.88238  2.83526  2.86304  2.63753  2.91120  3.27973  5.43050  4.08045    152 e - - -
        2.68618  4.42225  2.77519  2.73123  3.46354  2.40513  3.72494  3.29354  2.67741  2.69355  4.24690  2.90347  2.73739  3.18146  2.89801  2.37887  2.77519  2.98518  4.58477  3.61503
        0.01839  4.40100  5.12334  0.61958  0.77255  0.48576  0.95510
    153   2.94971  4.39464  4.56057  4.05061  3.71210  4.09887  4.70663  1.88156  3.93196  2.50532  3.60734  4.22582  4.53468  4.20114  4.13230  2.97765  3.05840  0.90950  5.39886  4.18914    153 v - - -
        2.68618  4.42225  2.77519  2.73123  3.46354  2.40513  3.72494  3.29354  2.67741  2.69355  4.24690  2.90347  2.73739  3.18146  2.89801  2.37887  2.77519  2.98518  4.58477  3.61503
        0.01839  4.40100  5.12334  0.61958  0.77255  0.48576  0.95510
    154   3.19841  4.48379  5.06514  4.52641  3.68678  4.58905  5.07000  1.31729  4.41526  2.00381  3.31136  4.68083  4.87093  4.62107  4.55257  3.94673  2.96186  1.16177  5.54083  4.35837    154 v - - -
        2.68618  4.42225  2.77519  2.73123  3.46354  2.40513  3.72494  3.29354  2.67741  2.69355  4.24690  2.90347  2.73739  3.18146  2.89801  2.37887  2.77519  2.98518  4.58477  3.61503
        0.01839  4.40100  5.12334  0.61958  0.77255  0.48576  0.95510
    155   2.54710  5.08177  2.85430  2.34956  4.38807  3.32452  3.51283  3.70907  2.27504  3.37033  3.96646  2.58295  3.85138  2.37991  2.88701  2.04654  2.67638  3.19702  5.53850  4.15253    155 s - - -
        2.68618  4.42225  2.77519  2.73123  3.46354  2.40513  3.72494  3.29354  2.67741  2.69355  4.24690  2.90347  2.73739  3.18146  2.89801  2.37887  2.77519  2.98518  4.58477  3.61503
        0.01839  4.40100  5.12334  0.61958  0.77255  0.48576  0.95510
    156   2.56245  5.05843  3.00296  2.44687  4.36075  2.78337  3.25577  3.80586  1.71283  3.02711  4.11859  2.97893  3.34069  2.78670  2.68642  2.55032  2.92647  3.42765  5.51784  4.15226    156 k - - -
        2.68618  4.42225  2.77519  2.73123  3.46354  2.40513  3.72494  3.29354  2.67741  2.69355  4.24690  2.90347  2.73739  3.18146  2.89801  2.37887  2.77519  2.98518  4.58477  3.61503
        0.01839  4.40100  5.12334  0.61958  0.77255  0.48576  0.95510
    157   1.11224  4.02415  3.75985  3.26009  3.93517  3.37605  4.18996  3.22485  3.00142  2.99946  3.88138  3.51527  3.98011  3.50252  3.51933  2.39784  2.91303  2.42129  5.35879  4.13895    157 a - - -
        2.68618  4.42225  2.77519  2.73123  3.46354  2.40513  3.72494  3.29354  2.67741  2.69355  4.24690  2.90347  2.73739  3.18146  2.89801  2.37887  2.77519  2.98518  4.58477  3.61503
        0.01839  4.40100  5.12334  0.61958  0.77255  0.48576  0.95510
    158   3.00738  4.95827  3.26841  3.14321  4.48475  0.64113  3.21007  4.33010  3.33282  3.90682  4.80921  3.53773  4.20689  3.69880  3.66071  3.11313  3.42900  3.86854  5.78351  4.45593    158 G - - -
        2.68618  4.42225  2.77519  2.73123  3.46354  2.40513  3.72494  3.29354  2.67741  2.69355  4.24690  2.90347  2.73739  3.18146  2.89801  2.37887  2.77519  2.98518  4.58477  3.61503
        0.01839  4.40100  5.12334  0.61958  0.77255  0.48576  0.95510
    159   2.12508  5.15654  2.59567  2.29723  4.47979  3.33379  3.47508  3.95163  1.97515  3.45224  4.20647  2.92682  3.85750  2.59952  2.89780  2.21812  2.87436  3.53611  5.60189  4.20180    159 k - - -
        2.68618  4.42225  2.77519  2.73123  3.46354  2.40513  3.72494  3.29354  2.67741  2.69355  4.24690  2.90347  2.73739  3.18146  2.89801  2.37887  2.77519  2.98518  4.58477  3.61503
        0.01839  4.40100  5.12334  0.61958  0.77255  0.48576  0.95510
    160   2.87275  5.26811  2.94207  2.42044  4.63065  3.54226  3.73283  4.07351  1.50294  3.55669  4.34516  1.96016  3.97066  2.85216  2.66580  2.53671  3.09881  3.67768  5.67858  4.33059    160 k - - -
        2.68618  4.42225  2.77519  2.73123  3.46354  2.40513  3.72494  3.29354  2.67741  2.69355  4.24690  2.90347  2.73739  3.18146  2.89801  2.37887  2.77519  2.98518  4.58477  3.61503
        0.01839  4.40100  5.12334  0.61958  0.77255  0.48576  0.95510
    161   3.37640  4.68325  5.12580  4.55279  3.30534  4.66662  5.01028  2.18697  4.41540  1.30959  1.56317  4.74233  4.87228  4.49850  4.49496  4.01209  3.60110  1.71212  5.32301  4.25614    161 l - - -
        2.68618  4.42225  2.77519  2.73123  3.46354  2.40513  3.72494  3.29354  2.67741  2.69355  4.24690  2.90347  2.73739  3.18146  2.89801  2.37887  2.77519  2.98518  4.58477  3.61503
        0.01839  4.40100  5.12334  0.61958  0.77255  0.48576  0.95510
    162   2.77042  5.16643  2.24514  2.41214  4.59341  2.91424  3.78953  4.06869  2.61382  3.58718  4.36358  2.18896  3.91132  2.91732  3.12000  1.72510  2.69538  3.63865  5.75190  4.34501    162 s - - -
        2.68618  4.42225  2.77519  2.73123  3.46354  2.40513  3.72494  3.29354  2.67741  2.69355  4.24690  2.90347  2.73739  3.18146  2.89801  2.37887  2.77519  2.98518  4.58477  3.61503
        0.01839  4.40100  5.12334  0.61958  0.77255  0.48576  0.95510
    163   2.53596  5.25737  2.19364  1.56155  4.58247  3.32717  3.71276  4.06420  2.50482  3.55880  4.32052  2.91289  3.40470  2.73655  3.01237  2.47178  3.01070  3.47799  5.70734  4.28816    163 e - - -
        2.68618  4.42225  2.77519  2.73123  3.46354  2.40513  3.72494  3.29354  2.67741  2.69355  4.24690  2.90347  2.73739  3.18146  2.89801  2.37887  2.77519  2.98518  4.58477  3.61503
        0.01839  4.40100  5.12334  0.61958  0.77255  0.48576  0.95510
    164   1.81445  4.46790  2.93062  2.38459  4.16650  3.30903  3.70740  3.59006  2.50096  3.06405  3.98677  2.82242  3.13441  2.85094  2.96796  2.41432  2.74656  3.18472  5.41405  4.07184    164 a - - -
        2.68618  4.42225  2.77519  2.73123  3.46354  2.40513  3.72494  3.29354  2.67741  2.69355  4.24690  2.90347  2.73739  3.18146  2.89801  2.37887  2.77519  2.98518  4.58477  3.61503
        0.01839  4.40100  5.12334  0.61958  0.77255  0.48576  0.95510
    165   2.71549  3.59637  4.13611  3.55630  3.31737  3.82011  4.14240  2.17086  3.44680  2.12134  2.96160  3.78767  4.19162  3.67919  3.64946  1.79181  2.95239  2.04972  4.85153  3.65717    165 s - - -
        2.68618  4.42225  2.77519  2.73123  3.46354  2.40513  3.72494  3.29354  2.67741  2.69355  4.24690  2.90347  2.73739  3.18146  2.89801  2.37887  2.77519  2.98518  4.58477  3.61503
        0.01839  4.40100  5.12334  0.61958  0.77255  0.48576  0.95510
    166   2.60562  5.04124  3.20384  2.60647  4.34443  3.58857  3.71125  3.74679  1.70580  3.01663  3.84243  3.10439  3.96817  2.84523  1.77997  2.69605  3.02233  3.25987  5.48005  4.17725    166 k - - -
        2.68618  4.42225  2.77519  2.73123  3.46354  2.40513  3.72494  3.29354  2.67741  2.69355  4.24690  2.90347  2.73739  3.18146  2.89801  2.37887  2.77519  2.98518  4.58477  3.61503
        0.01839  4.40100  5.12334  0.61958  0.77255  0.48576  0.95510
    167   2.50768  5.13640  2.87078  2.31235  4.46167  3.45623  3.64599  3.77919  1.83932  3.43144  4.18401  2.54416  3.85157  2.47362  2.77997  2.47078  2.53665  3.51671  5.57956  4.18471    167 k - - -
        2.68618  4.42225  2.77519  2.73123  3.46354  2.40513  3.72494  3.29354  2.67741  2.69355  4.24690  2.90347  2.73739  3.18146  2.89801  2.37887  2.77519  2.98518  4.58477  3.61503
        0.01839  4.40100  5.12334  0.61958  0.77255  0.48576  0.95510
    168   2.22479  5.04279  2.97640  2.43007  4.34394  3.37594  3.54736  3.65694  2.42212  3.33292  4.10815  2.44961  3.86506  2.38442  2.68846  2.09234  2.81138  3.41234  5.51571  4.14272    168 s - - -
        2.68618  4.42225  2.77519  2.73123  3.46354  2.40513  3.72494  3.29354  2.67741  2.69355  4.24690  2.90347  2.73739  3.18146  2.89801  2.37887  2.77519  2.98518  4.58477  3.61503
        0.01839  4.40100  5.12334  0.61958  0.77255  0.48576  0.95510
    169   3.34656  4.59355  5.28093  4.75842  3.29926  4.80643  5.30969  1.02605  4.66026  1.97846  3.46325  4.91745  5.03705  4.82855  4.78002  4.18759  3.59197  1.32475  5.66034  4.49144    169 i - - -
        2.68618  4.42225  2.77519  2.73123  3.46354  2.40513  3.72494  3.29354  2.67741  2.69355  4.24690  2.90347  2.73739  3.18146  2.89801  2.37887  2.77519  2.98518  4.58477  3.61503
        0.01839  4.40100  5.12334  0.61958  0.77255  0.48576  0.95510
    170   2.65622  3.92265  3.60832  3.27597  3.02474  3.55094  3.77729  2.49833  3.29876  1.81584  3.12017  3.65891  4.10571  3.54056  3.52418  2.79346  2.47937  2.17116  4.78144  2.97947    170 l - - -
        2.68618  4.42225  2.77519  2.73123  3.46354  2.40513  3.72494  3.29354  2.67741  2.69355  4.24690  2.90347  2.73739  3.18146  2.89801  2.37887  2.77519  2.98518  4.58477  3.61503
```

```
      0.01839  4.40100  5.12334  0.61958  0.77255  0.48576  0.95510
171   2.27945  5.07309  2.84720  2.36487  4.38247  2.70023  3.67799  3.83712  2.39096  3.37155  4.14385  2.65488  3.86649  2.62295  2.93038  2.19433  2.39273  3.44871  5.55014  4.16793    171 s - - -
      2.68618  4.42225  2.77519  2.73123  3.46354  2.40513  3.72494  3.29354  2.67741  2.69355  4.24690  2.90347  2.73739  3.18146  2.89801  2.37887  2.77519  2.98518  4.58477  3.61503
      0.01839  4.40100  5.12334  0.61958  0.77255  0.48576  0.95510
172   2.67582  4.20772  4.16637  3.58697  3.12216  3.83287  4.15921  2.48987  3.47248  1.52801  3.18516  3.81069  4.20404  3.70146  3.66778  2.88297  2.28564  1.95883  4.85386  3.66266    172 l - - -
      2.68618  4.42225  2.77519  2.73123  3.46354  2.40513  3.72494  3.29354  2.67741  2.69355  4.24690  2.90347  2.73739  3.18146  2.89801  2.37887  2.77519  2.98518  4.58477  3.61503
      0.01839  4.40100  5.12334  0.61958  0.77255  0.48576  0.95510
173   2.87380  4.53697  5.07164  4.52374  3.54219  4.58947  5.03351  1.47576  4.40181  1.26828  3.35997  4.68376  4.85577  4.56677  4.51815  3.94428  3.48198  1.57496  5.45265  4.31135    173 l - - -
      2.68618  4.42225  2.77519  2.73123  3.46354  2.40513  3.72494  3.29354  2.67741  2.69355  4.24690  2.90347  2.73739  3.18146  2.89801  2.37887  2.77519  2.98518  4.58477  3.61503
      0.01839  4.40100  5.12334  0.61958  0.77255  0.48576  0.95510
174   2.65386  5.14253  2.22935  1.90665  4.44941  3.45738  3.67995  3.91210  2.26017  2.49695  4.19778  2.93736  3.87285  2.79434  2.86163  2.47041  2.95031  3.51303  5.59608  4.20258    174 e - - -
      2.68618  4.42225  2.77519  2.73123  3.46354  2.40513  3.72494  3.29354  2.67741  2.69355  4.24690  2.90347  2.73739  3.18146  2.89801  2.37887  2.77519  2.98518  4.58477  3.61503
      0.01839  4.40100  5.12334  0.61958  0.77255  0.48576  0.95510
175   2.68657  5.15839  2.51199  1.99870  4.48480  2.72414  3.65073  3.95984  2.29613  3.45578  3.48049  2.63384  3.85071  2.66720  2.89248  2.32596  2.79348  3.53930  5.60163  4.19847    175 e - - -
      2.68618  4.42225  2.77519  2.73123  3.46354  2.40513  3.72494  3.29354  2.67741  2.69355  4.24690  2.90347  2.73739  3.18146  2.89801  2.37887  2.77519  2.98518  4.58477  3.61503
      0.01839  4.40100  5.12334  0.61958  0.77255  0.48576  0.95510
176   2.66682  4.66954  2.94576  1.98302  3.85704  3.54688  3.39904  2.80477  2.63236  2.62381  2.55736  3.13910  3.93382  2.97038  3.06505  2.76430  2.82280  2.97157  5.20789  3.68333    176 e - - -
      2.68618  4.42225  2.77519  2.73123  3.46354  2.40513  3.72494  3.29354  2.67741  2.69355  4.24690  2.90347  2.73739  3.18146  2.89801  2.37887  2.77519  2.98518  4.58477  3.61503
      0.01839  4.40100  5.12334  0.61958  0.77255  0.48576  0.95510
177   2.62284  3.59220  4.10675  3.36776  3.24965  3.63355  4.06592  2.16496  3.40338  1.78989  2.74376  3.73589  4.13107  3.62763  3.59028  2.43104  2.57827  2.38087  4.75858  3.56584    177 l - - -
      2.68618  4.42225  2.77519  2.73123  3.46354  2.40513  3.72494  3.29354  2.67741  2.69355  4.24690  2.90347  2.73739  3.18146  2.89801  2.37887  2.77519  2.98518  4.58477  3.61503
      0.01839  4.40100  5.12334  0.61958  0.77255  0.48576  0.95510
178   2.50620  4.91928  2.58019  2.48109  4.17706  2.29364  3.28066  3.43405  2.48056  2.76064  3.99110  2.94795  3.63101  2.83180  2.95042  2.48137  2.79896  3.12392  5.41666  4.06938    178 g - - -
      2.68618  4.42225  2.77519  2.73123  3.46354  2.40513  3.72494  3.29354  2.67741  2.69355  4.24690  2.90347  2.73739  3.18146  2.89801  2.37887  2.77519  2.98518  4.58477  3.61503
      0.01839  4.40100  5.12334  0.61958  0.77255  0.48576  0.95510
179   2.73740  5.18829  2.43008  1.98808  4.50227  3.00311  2.22519  3.97344  2.47330  3.48304  4.24810  2.92638  3.87970  2.80913  2.97080  2.35832  2.97526  3.25865  5.64207  4.23763    179 e - - -
      2.68618  4.42225  2.77519  2.73123  3.46354  2.40513  3.72494  3.29354  2.67741  2.69355  4.24690  2.90347  2.73739  3.18146  2.89801  2.37887  2.77519  2.98518  4.58477  3.61503
      0.03841  4.40100  3.67237  0.61958  0.77255  0.48576  0.95510
180   2.55305  5.11284  2.22016  2.35234  4.42949  3.27893  3.53995  3.89514  2.32093  3.40727  4.16585  2.92879  3.56433  2.75885  2.65303  1.98660  2.84454  3.31063  5.56636  4.17306    180 s - - -
      2.68607  4.42226  2.77520  2.73124  3.46354  2.40513  3.72495  3.29355  2.67741  2.69355  4.24690  2.90347  2.73740  3.18147  2.89801  2.37887  2.77520  2.98519  4.58477  3.61504
      0.07297  3.05899  3.75345  0.45404  1.00801  0.46763  0.98480
181   2.88870  5.42874  1.71599  1.66002  4.74163  3.42842  3.78657  4.23991  2.64160  3.72323  4.05814  2.13853  3.93617  2.91337  3.17583  2.72761  3.14158  3.80658  5.86863  4.41712    182 e - - -
      2.68618  4.42225  2.77519  2.73123  3.46354  2.40513  3.72494  3.29354  2.67741  2.69355  4.24690  2.90347  2.73739  3.18146  2.89801  2.37887  2.77519  2.98518  4.58477  3.61503
      0.01871  4.38383  5.10617  0.61958  0.77255  0.51179  0.91484
182   2.87172  5.65640  1.01980  1.99785  4.97539  3.12552  3.93259  4.49247  2.92805  3.97973  4.80835  2.79527  4.01819  3.08954  3.52313  2.88232  3.16274  4.05183  6.14160  4.63768    183 d - - -
      2.68618  4.42225  2.77519  2.73123  3.46354  2.40513  3.72494  3.29354  2.67741  2.69355  4.24690  2.90347  2.73739  3.18146  2.89801  2.37887  2.77519  2.98518  4.58477  3.61503
      0.01871  4.38383  5.10617  0.61958  0.77255  0.51179  0.91484
183   2.00552  4.88578  2.96514  2.45655  4.13060  3.33583  3.55067  3.22381  2.14328  3.14692  3.95478  2.86422  3.87926  2.83614  2.73132  2.69135  2.69466  2.90516  5.38352  3.69593    184 a - - -
      2.68618  4.42225  2.77519  2.73123  3.46354  2.40513  3.72494  3.29354  2.67741  2.69355  4.24690  2.90347  2.73739  3.18146  2.89801  2.37887  2.77519  2.98518  4.58477  3.61503
      0.01871  4.38383  5.10617  0.61958  0.77255  0.51179  0.91484
184   2.65777  3.86062  3.94260  3.36669  3.32694  3.71777  4.03733  2.63261  3.27273  2.14276  2.45413  3.64146  3.75051  3.52523  3.51479  2.80483  1.88444  2.22513  4.82702  3.62581    185 t - - -
      2.68618  4.42225  2.77519  2.73123  3.46354  2.40513  3.72494  3.29354  2.67741  2.69355  4.24690  2.90347  2.73739  3.18146  2.89801  2.37887  2.77519  2.98518  4.58477  3.61503
      0.01871  4.38383  5.10617  0.61958  0.77255  0.51179  0.91484
185   2.16271  4.73915  3.18190  2.61802  3.95507  3.53606  3.74617  3.14312  2.47863  2.79616  3.82842  2.95191  3.92566  2.92944  2.00499  2.75462  2.56704  2.88892  5.26929  3.97049    186 r - - -
      2.68618  4.42225  2.77519  2.73123  3.46354  2.40513  3.72494  3.29354  2.67741  2.69355  4.24690  2.90347  2.73739  3.18146  2.89801  2.37887  2.77519  2.98518  4.58477  3.61503
      0.01871  4.38383  5.10617  0.61958  0.77255  0.46982  0.98113
186   2.62401  4.15475  4.18904  3.60386  3.27177  3.80952  4.12473  1.87821  3.48014  2.21654  2.57764  3.32788  4.17752  3.69984  3.65471  2.90826  2.60466  1.84044  4.79523  3.60345    187 v - - -
      2.68618  4.42225  2.77519  2.73123  3.46354  2.40513  3.72494  3.29354  2.67741  2.69355  4.24690  2.90347  2.73739  3.18146  2.89801  2.37887  2.77519  2.98518  4.58477  3.61503
      0.01839  4.40100  5.12334  0.61958  0.77255  0.48576  0.95510
187   1.77288  3.71177  3.49838  2.93409  3.56947  3.44725  3.59448  2.93347  2.89121  2.55468  3.38181  3.34799  4.00540  3.19966  3.25617  2.54497  2.31231  2.70075  5.00433  3.49533    188 a - - -
      2.68618  4.42225  2.77519  2.73123  3.46354  2.40513  3.72494  3.29354  2.67741  2.69355  4.24690  2.90347  2.73739  3.18146  2.89801  2.37887  2.77519  2.98518  4.58477  3.61503
      0.07255  4.40100  2.85223  0.61958  0.77255  0.48576  0.95510
188   2.19711  4.66935  3.17901  2.64822  3.93606  3.48104  3.78533  3.33793  2.64206  2.98882  3.83212  2.41543  3.91069  2.98149  3.07547  2.00453  2.83717  2.88122  5.27217  2.90768    189 s - - -
      2.68619  4.42226  2.77520  2.73124  3.46355  2.40513  3.72495  3.29355  2.67741  2.69342  4.24690  2.90347  2.73740  3.18147  2.89801  2.37888  2.77520  2.98519  4.58478  3.61504
      0.04089  3.38775  5.07020  0.63655  0.75314  0.44039  1.03223
189   0.53116  4.44050  3.96945  3.81119  4.72559  2.98321  4.79722  4.04027  3.87884  3.84687  4.72685  3.79327  4.03247  4.14634  4.09115  2.74882  3.07817  3.48357  6.06760  4.93231    192 A - - -
      2.68618  4.42225  2.77519  2.73123  3.46354  2.40513  3.72494  3.29354  2.67741  2.69355  4.24690  2.90347  2.73739  3.18146  2.89801  2.37887  2.77519  2.98518  4.58477  3.61503
      0.01839  4.40100  5.12334  0.61958  0.77255  0.48576  0.95510
190   2.60205  5.12341  3.05684  2.51684  4.47946  2.21988  3.70255  3.91540  1.70014  3.42859  4.21357  3.03442  3.93115  2.73844  2.40533  2.54890  3.00730  3.53132  5.57729  4.23623    193 k - - -
      2.68618  4.42225  2.77519  2.73123  3.46354  2.40513  3.72494  3.29354  2.67741  2.69355  4.24690  2.90347  2.73739  3.18146  2.89801  2.37887  2.77519  2.98518  4.58477  3.61503
      0.01839  4.40100  5.12334  0.61958  0.77255  0.48576  0.95510
191   3.08688  2.12860  4.85496  4.27396  3.30229  4.31088  4.66826  2.01064  4.11654  1.19737  2.92486  4.40479  4.60794  4.25633  4.20298  3.63934  3.32090  2.20970  5.11330  3.97881    194 l - - -
      2.68618  4.42225  2.77519  2.73123  3.46354  2.40513  3.72494  3.29354  2.67741  2.69355  4.24690  2.90347  2.73739  3.18146  2.89801  2.37887  2.77519  2.98518  4.58477  3.61503
      0.01839  4.40100  5.12334  0.61958  0.77255  0.48576  0.95510
192   2.75247  4.27590  4.63996  4.04697  2.59519  4.06135  4.34731  2.04019  3.88315  1.28525  3.14309  4.14370  4.38864  4.02227  3.95831  3.37172  3.12140  2.25358  4.82411  2.75976    195 l - - -
      2.68618  4.42225  2.77519  2.73123  3.46354  2.40513  3.72494  3.29354  2.67741  2.69355  4.24690  2.90347  2.73739  3.18146  2.89801  2.37887  2.77519  2.98518  4.58477  3.61503
      0.01839  4.40100  5.12334  0.61958  0.77255  0.48576  0.95510
193   2.23783  4.41309  3.63923  3.53609  4.83702  0.71177  4.68983  4.28824  3.76159  3.98871  4.80108  3.61258  3.95271  3.99381  4.02632  2.45514  3.00426  3.59995  6.16663  4.97990    196 g - - -
      2.68618  4.42225  2.77519  2.73123  3.46354  2.40513  3.72494  3.29354  2.67741  2.69355  4.24690  2.90347  2.73739  3.18146  2.89801  2.37887  2.77519  2.98518  4.58477  3.61503
      0.01839  4.40100  5.12334  0.61958  0.77255  0.48576  0.95510
194   1.76552  4.73229  3.16748  2.17527  3.98632  3.51139  3.77456  3.17345  2.61190  3.01781  3.71485  3.11669  3.92304  2.95678  3.05303  2.24099  2.90985  2.86168  5.30768  4.00259    197 a - - -
      2.68618  4.42225  2.77519  2.73123  3.46354  2.40513  3.72494  3.29354  2.67741  2.69355  4.24690  2.90347  2.73739  3.18146  2.89801  2.37887  2.77519  2.98518  4.58477  3.61503
      0.01839  4.40100  5.12334  0.61958  0.77255  0.48576  0.95510
195   2.81382  4.53150  4.69577  4.11141  3.20434  4.29862  4.57935  2.30556  3.94748  0.83283  2.98559  4.31365  4.58073  4.11469  4.07642  3.61336  3.36037  2.49083  4.31727  3.90306    198 l - - -
      2.68618  4.42225  2.77519  2.73123  3.46354  2.40513  3.72494  3.29354  2.67741  2.69355  4.24690  2.90347  2.73739  3.18146  2.89801  2.37887  2.77519  2.98518  4.58477  3.61503
      0.01839  4.40100  5.12334  0.61958  0.77255  0.48576  0.95510
```

```
196   2.43119  2.75450  4.38991  3.79205  2.82010  3.81648  4.14153  2.10288  3.62994  2.01439  3.12843  3.89422  4.18227  3.80814  3.72696  2.72855  2.58520  2.04439  4.71696  3.53404    199 l - - -
      2.68618  4.42225  2.77519  2.73123  3.46354  2.40513  3.72494  3.29354  2.67741  2.69355  4.24690  2.90347  2.73739  3.18146  2.89801  2.37887  2.77519  2.98518  4.58477  3.61503
      0.01839  4.40100  5.12334  0.61958  0.77255  0.48576  0.95510
197   2.27884  5.13167  2.95109  2.22428  4.45630  3.09516  3.64773  3.92240  1.95859  3.15982  4.17996  2.73217  3.85642  2.57285  2.50751  2.49221  2.91624  3.51176  5.57369  4.18374    200 k - - -
      2.68618  4.42225  2.77519  2.73123  3.46354  2.40513  3.72494  3.29354  2.67741  2.69355  4.24690  2.90347  2.73739  3.18146  2.89801  2.37887  2.77519  2.98518  4.58477  3.61503
      0.01839  4.40100  5.12334  0.61958  0.77255  0.48576  0.95510
198   2.65695  3.78859  3.89809  3.31970  2.64606  3.71927  3.61040  2.28726  3.23199  2.40296  3.31537  2.89071  4.09210  3.24546  3.16849  2.70788  2.72306  2.36083  3.49754  2.98527    201 i - - -
      2.68618  4.42225  2.77519  2.73123  3.46354  2.40513  3.72494  3.29354  2.67741  2.69355  4.24690  2.90347  2.73739  3.18146  2.89801  2.37887  2.77519  2.98518  4.58477  3.61503
      0.01839  4.40100  5.12334  0.61958  0.77255  0.48576  0.95510
199   3.55930  4.85108  5.26370  4.72682  3.32938  4.86082  5.24700  2.18089  4.56315  0.69252  3.11109  4.95223  5.03901  4.65724  4.65202  4.24649  3.79493  1.91871  5.47035  4.40287    202 L - - -
      2.68618  4.42225  2.77519  2.73123  3.46354  2.40513  3.72494  3.29354  2.67741  2.69355  4.24690  2.90347  2.73739  3.18146  2.89801  2.37887  2.77519  2.98518  4.58477  3.61503
      0.01839  4.40100  5.12334  0.61958  0.77255  0.48576  0.95510
200   2.72137  5.10270  2.74744  2.26192  4.43707  3.44718  3.71602  3.89491  2.50266  3.42891  4.20566  2.59165  2.58785  2.83871  2.99282  1.93262  2.40472  3.50001  5.60744  4.21997    203 s - - -
      2.68618  4.42225  2.77519  2.73123  3.46354  2.40513  3.72494  3.29354  2.67741  2.69355  4.24690  2.90347  2.73739  3.18146  2.89801  2.37887  2.77519  2.98518  4.58477  3.61503
      0.01839  4.40100  5.12334  0.61958  0.77255  0.48576  0.95510
201   2.37794  5.09676  2.46655  1.99924  4.40735  3.22407  3.53598  3.49173  2.40472  3.38795  4.14860  2.82871  3.03760  2.76153  2.89173  2.55773  2.69623  3.23614  5.55227  4.16266    204 e - - -
      2.68618  4.42225  2.77519  2.73123  3.46354  2.40513  3.72494  3.29354  2.67741  2.69355  4.24690  2.90347  2.73739  3.18146  2.89801  2.37887  2.77519  2.98518  4.58477  3.61503
      0.05338  4.40100  3.22608  0.61958  0.77255  0.48576  0.95510
202   2.52840  5.17904  2.05326  1.82882  4.49791  3.43695  3.67384  3.97048  2.44621  3.47484  4.23522  2.91174  3.59560  2.54537  2.94373  2.68155  2.75407  3.15237  5.62915  4.22303    206 e - - -
      2.68618  4.42225  2.77519  2.73123  3.46354  2.40513  3.72494  3.29354  2.67741  2.69355  4.24690  2.90347  2.73739  3.18146  2.89801  2.37887  2.77519  2.98518  4.58477  3.61503
      0.01904  4.36666  5.08900  0.61958  0.77255  0.53673  0.87864
203   2.52579  4.99667  2.99349  1.85598  4.27751  3.46988  3.66294  3.53837  2.41868  3.05134  4.05717  2.97104  3.86224  2.20034  2.69719  2.67070  2.68665  3.09941  5.46967  4.10733    207 e - - -
      2.68618  4.42225  2.77519  2.73123  3.46354  2.40513  3.72494  3.29354  2.67741  2.69355  4.24690  2.90347  2.73739  3.18146  2.89801  2.37887  2.77519  2.98518  4.58477  3.61503
      0.01904  4.36666  5.08900  0.61958  0.77255  0.45516  1.00607
204   1.76708  4.21796  4.02599  3.45513  2.88366  3.50458  4.10174  2.58830  3.35726  1.78113  3.33428  3.71004  4.14566  3.60296  3.58630  3.04385  2.76698  2.21344  4.86432  3.66459    208 a - - -
      2.68618  4.42225  2.77519  2.73123  3.46354  2.40513  3.72494  3.29354  2.67741  2.69355  4.24690  2.90347  2.73739  3.18146  2.89801  2.37887  2.77519  2.98518  4.58477  3.61503
      0.01839  4.40100  5.12334  0.61958  0.77255  0.48576  0.95510
205   2.53056  5.10714  2.75969  2.30495  4.42319  3.08861  3.13520  3.65060  2.23556  3.31676  4.15567  2.67768  3.74953  2.75085  2.59560  2.39149  2.45318  3.27742  5.55756  4.16500    209 k - - -
      2.68618  4.42225  2.77519  2.73123  3.46354  2.40513  3.72494  3.29354  2.67741  2.69355  4.24690  2.90347  2.73739  3.18146  2.89801  2.37887  2.77519  2.98518  4.58477  3.61503
      0.01839  4.40100  5.12334  0.61958  0.77255  0.48576  0.95510
206   2.63547  5.13734  2.63570  2.21700  4.46416  2.98717  3.55613  3.64205  2.16562  3.43480  4.18307  2.63945  3.13525  2.56045  2.87222  2.45918  2.71625  3.13335  5.58102  4.18100    210 k - - -
      2.68618  4.42225  2.77519  2.73123  3.46354  2.40513  3.72494  3.29354  2.67741  2.69355  4.24690  2.90347  2.73739  3.18146  2.89801  2.37887  2.77519  2.98518  4.58477  3.61503
      0.01839  4.40100  5.12334  0.61958  0.77255  0.48576  0.95510
207   2.42296  4.32406  4.68942  4.10963  3.01877  4.17265  4.53102  1.71786  3.96841  1.40671  3.26803  4.24546  4.49747  4.13950  4.07889  3.49059  3.04084  1.87986  5.05311  3.88920    211 l - - -
      2.68618  4.42225  2.77519  2.73123  3.46354  2.40513  3.72494  3.29354  2.67741  2.69355  4.24690  2.90347  2.73739  3.18146  2.89801  2.37887  2.77519  2.98518  4.58477  3.61503
      0.01839  4.40100  5.12334  0.61958  0.77255  0.48576  0.95510
208   3.15006  4.69414  5.16448  4.59294  2.88412  4.68392  5.01132  1.74047  4.45918  0.86591  3.08648  4.76954  4.88331  4.51979  4.52344  4.03154  3.61703  2.25549  5.29733  4.21469    212 l - - -
      2.68618  4.42225  2.77519  2.73123  3.46354  2.40513  3.72494  3.29354  2.67741  2.69355  4.24690  2.90347  2.73739  3.18146  2.89801  2.37887  2.77519  2.98518  4.58477  3.61503
      0.01839  4.40100  5.12334  0.61958  0.77255  0.48576  0.95510
209   2.63602  5.17034  2.80620  2.19395  4.50838  3.45989  3.46645  3.98282  1.91944  3.13111  4.21504  2.68674  3.85509  2.12632  2.64604  2.65896  2.86989  3.55767  5.60242  4.20510    213 k - - -
      2.68618  4.42225  2.77519  2.73123  3.46354  2.40513  3.72494  3.29354  2.67741  2.69355  4.24690  2.90347  2.73739  3.18146  2.89801  2.37887  2.77519  2.98518  4.58477  3.61503
      0.01839  4.40100  5.12334  0.61958  0.77255  0.48576  0.95510
210   2.55308  5.17430  2.52516  2.12323  4.50728  3.44590  3.38550  3.98683  2.33940  3.35688  4.21946  2.48935  3.84808  2.33243  2.88565  2.36066  2.56401  3.55869  5.61306  4.20594    214 e - - -
      2.68618  4.42225  2.77519  2.73123  3.46354  2.40513  3.72494  3.29354  2.67741  2.69355  4.24690  2.90347  2.73739  3.18146  2.89801  2.37887  2.77519  2.98518  4.58477  3.61503
      0.06328  4.40100  3.01491  0.61958  0.77255  0.48576  0.95510
211   2.60775  4.43861  2.92126  2.43367  4.38697  3.47670  2.45629  3.83499  1.97977  3.35827  4.13125  2.89382  3.46187  2.76557  2.20931  2.68033  2.92199  3.44940  5.52262  4.15618    215 k - - -
      2.68618  4.42225  2.77519  2.73123  3.46354  2.40513  3.72494  3.29354  2.67741  2.69355  4.24690  2.90347  2.73739  3.18146  2.89801  2.37887  2.77519  2.98518  4.58477  3.61503
      0.01923  4.35695  5.07929  0.61958  0.77255  0.44737  1.01972
212   3.38015  4.62644  5.30190  4.77870  3.64283  4.83782  5.34751  1.54531  4.67391  1.47035  3.42041  4.94963  5.05687  4.83449  4.79112  4.22253  3.62483  1.09765  5.67703  4.53834    216 v - - -
      2.68618  4.42225  2.77519  2.73123  3.46354  2.40513  3.72494  3.29354  2.67741  2.69355  4.24690  2.90347  2.73739  3.18146  2.89801  2.37887  2.77519  2.98518  4.58477  3.61503
      0.01839  4.40100  5.12334  0.61958  0.77255  0.48576  0.95510
213   2.68588  4.21860  3.95569  3.08146  2.90357  3.75531  4.04214  2.51751  3.27685  1.39167  3.14393  3.65854  4.12466  3.32029  3.51592  2.88843  2.78586  2.45171  4.82032  3.62045    217 l - - -
      2.68618  4.42225  2.77519  2.73123  3.46354  2.40513  3.72494  3.29354  2.67741  2.69355  4.24690  2.90347  2.73739  3.18146  2.89801  2.37887  2.77519  2.98518  4.58477  3.61503
      0.01839  4.40100  5.12334  0.61958  0.77255  0.48576  0.95510
214   2.25091  4.25490  2.94458  2.36228  4.17331  3.48642  3.48128  3.46916  2.47527  3.03844  3.98666  2.40364  3.87773  2.75672  2.94498  2.30037  2.47808  3.16525  5.41242  4.06518    218 a - - -
      2.68607  4.42237  2.77443  2.73126  3.46366  2.40518  3.72506  3.29366  2.67753  2.69352  4.24686  2.90349  2.73751  3.18158  2.89813  2.37899  2.77531  2.98488  4.58489  3.61515
      0.77880  1.27782  1.33789  0.67855  0.70796  0.48576  0.95510
215   2.32421  4.65472  3.06086  2.52827  3.92514  3.40721  3.68733  3.32659  2.52157  2.86430  3.79838  2.39636  3.57652  2.86728  2.96303  2.17126  2.75079  2.93902  5.23753  3.55052    221 s - - -
      2.68618  4.42225  2.77519  2.73123  3.46354  2.40513  3.72494  3.29354  2.67741  2.69355  4.24690  2.90347  2.73739  3.18146  2.89801  2.37887  2.77519  2.98518  4.58477  3.61503
      0.02481  4.10469  4.82703  0.61958  0.77255  0.77081  0.62108
216   2.69977  5.04143  2.22038  2.32387  4.44363  2.06282  3.72176  3.90413  2.45290  3.45028  4.24880  2.77409  3.83055  2.86454  3.07495  2.19809  2.84376  3.50063  5.63986  4.24499    222 g - - -
      2.68618  4.42225  2.77519  2.73123  3.46354  2.40513  3.72494  3.29354  2.67741  2.69355  4.24690  2.90347  2.73739  3.18146  2.89801  2.37887  2.77519  2.98518  4.58477  3.61503
      0.07877  4.13141  2.81864  0.61958  0.77255  0.79769  0.59851
217   2.56932  3.59164  3.46269  2.85745  3.31517  3.59489  3.86860  2.37917  2.99448  2.17738  3.15299  3.41177  3.36197  3.27488  3.29830  2.76398  2.80198  2.11045  4.79096  3.57440    223 v - - -
      2.68618  4.42225  2.77519  2.73123  3.46354  2.40513  3.72494  3.29354  2.67741  2.69355  4.24690  2.90347  2.73739  3.18146  2.89801  2.37887  2.77519  2.98518  4.58477  3.61503
      0.02549  4.07813  4.80047  0.61958  0.77255  0.63455  0.75539
218   2.72242  5.08499  1.62487  2.22423  4.38166  3.38306  3.69278  3.82414  2.51982  3.23849  4.18136  2.73222  3.85017  2.82956  3.01790  2.57760  2.85113  3.03862  5.58091  4.18996    224 d - - -
      2.68618  4.42225  2.77519  2.73123  3.46354  2.40513  3.72494  3.29354  2.67741  2.69355  4.24690  2.90347  2.73739  3.18146  2.89801  2.37887  2.77519  2.98518  4.58477  3.61503
      0.02260  4.19699  4.91933  0.61958  0.77255  0.42092  1.06840
219   2.50164  4.73649  3.13359  2.33860  3.94751  2.88377  3.73063  3.34638  2.53279  2.98450  3.82084  2.90359  3.28236  2.91097  2.96462  2.66127  2.68185  2.80910  2.70454  3.95781    225 e - - -
      2.68618  4.42225  2.77519  2.73123  3.46354  2.40513  3.72494  3.29354  2.67741  2.69355  4.24690  2.90347  2.73739  3.18146  2.89801  2.37887  2.77519  2.98518  4.58477  3.61503
      0.01910  4.36351  5.08586  0.61958  0.77255  0.45259  1.01054
220   2.57319  5.00226  2.77661  2.21057  4.28116  3.47136  3.17518  3.58168  2.43458  3.01497  2.92669  2.92151  3.40003  2.66533  2.74857  2.46957  2.53272  3.35683  5.47666  4.10973    226 e - - -
      2.68618  4.42225  2.77519  2.73123  3.46354  2.40513  3.72494  3.29354  2.67741  2.69355  4.24690  2.90347  2.73739  3.18146  2.89801  2.37887  2.77519  2.98518  4.58477  3.61503
      0.01839  4.40100  5.12334  0.61958  0.77255  0.48576  0.95510
221   2.55218  4.52073  3.08746  2.78849  3.35018  3.58888  3.58233  2.99602  2.76018  2.13031  3.61605  3.09472  3.28327  2.72362  3.16088  2.73893  2.67503  2.35268  5.08299  3.54306    227 l - - -
```

```
        2.68618  4.42225  2.77519  2.73123  3.46354  2.40513  3.72494  3.29354  2.67741  2.69355  4.24690  2.90347  2.73739  3.18146  2.89801  2.37887  2.77519  2.98518  4.58477  3.61503
        0.01839  4.40100  5.12334  0.61958  0.77255  0.48576  0.95510
  222   2.69489  5.14159  2.69072  2.32805  4.46527  2.91621  3.65897  3.93309  2.31888  3.43773  4.19472  2.75413  3.85994  2.76663  2.00661  2.37030  2.51497  3.52211  5.58990  4.19550   228 r - - -
        2.68618  4.42225  2.77519  2.73123  3.46354  2.40513  3.72494  3.29354  2.67741  2.69355  4.24690  2.90347  2.73739  3.18146  2.89801  2.37887  2.77519  2.98518  4.58477  3.61503
        0.01839  4.40100  5.12334  0.61958  0.77255  0.48576  0.95510
  223   2.71346  5.04734  3.01881  2.33841  4.33789  3.49881  2.00098  3.66622  2.00038  3.32127  3.91692  2.78480  3.89129  2.80144  2.83748  2.67227  2.81417  3.41034  5.50354  3.96579   229 k - - -
        2.68618  4.42225  2.77519  2.73123  3.46354  2.40513  3.72494  3.29354  2.67741  2.69355  4.24690  2.90347  2.73739  3.18146  2.89801  2.37887  2.77519  2.98518  4.58477  3.61503
        0.01839  4.40100  5.12334  0.61958  0.77255  0.48576  0.95510
  224   2.35473  4.41785  3.49241  2.93631  2.57097  2.16243  3.88675  2.93736  2.89644  2.51862  3.53357  3.35039  4.01076  3.05752  3.26028  2.51946  2.90040  2.70556  4.99540  3.44707   230 g - - -
        2.68618  4.42225  2.77519  2.73123  3.46354  2.40513  3.72494  3.29354  2.67741  2.69355  4.24690  2.90347  2.73739  3.18146  2.89801  2.37887  2.77519  2.98518  4.58477  3.61503
        0.01839  4.40100  5.12334  0.61958  0.77255  0.48576  0.95510
  225   2.42922  3.91154  3.12537  2.56961  4.12150  3.34205  3.36064  3.53397  2.51666  3.14106  3.95914  3.07295  3.90670  2.84194  2.11697  1.88710  2.91789  3.20799  5.38474  4.06378   231 s - - -
        2.68618  4.42225  2.77519  2.73123  3.46354  2.40513  3.72494  3.29354  2.67741  2.69355  4.24690  2.90347  2.73739  3.18146  2.89801  2.37887  2.77519  2.98518  4.58477  3.61503
        0.01839  4.40100  5.12334  0.61958  0.77255  0.48576  0.95510
  226   2.75034  4.26682  4.10846  3.54392  3.44957  3.83554  4.21812  1.85065  3.44788  2.44283  3.32351  3.80183  4.23181  3.70358  3.68482  2.23961  2.42272  1.68413  4.99255  3.78962   232 v - - -
        2.68618  4.42225  2.77519  2.73123  3.46354  2.40513  3.72494  3.29354  2.67741  2.69355  4.24690  2.90347  2.73739  3.18146  2.89801  2.37887  2.77519  2.98518  4.58477  3.61503
        0.01839  4.40100  5.12334  0.61958  0.77255  0.48576  0.95510
  227   2.45691  4.39453  4.46315  3.89519  3.40052  4.11265  4.48806  2.24972  3.77399  1.13053  3.17787  4.11947  4.45959  3.99496  3.96079  3.42601  2.92812  1.96837  5.11499  3.95173   233 l - - -
        2.68618  4.42225  2.77519  2.73123  3.46354  2.40513  3.72494  3.29354  2.67741  2.69355  4.24690  2.90347  2.73739  3.18146  2.89801  2.37887  2.77519  2.98518  4.58477  3.61503
        0.01839  4.40100  5.12334  0.61958  0.77255  0.48576  0.95510
  228   1.67294  4.43214  3.49720  2.94416  3.66090  3.27648  3.91871  2.52215  2.90100  2.72923  3.61160  3.24923  3.99216  3.21553  3.10133  2.71222  2.32298  2.62794  5.08476  3.84242   234 a - - -
        2.68618  4.42225  2.77519  2.73123  3.46354  2.40513  3.72494  3.29354  2.67741  2.69355  4.24690  2.90347  2.73739  3.18146  2.89801  2.37887  2.77519  2.98518  4.58477  3.61503
        0.01839  4.40100  5.12334  0.61958  0.77255  0.48576  0.95510
  229   3.64477  4.90256  5.39816  4.85149  3.30313  4.99446  5.34477  1.87920  4.69756  0.66895  3.06787  5.08561  5.11198  4.73526  4.75703  4.38334  3.86863  2.23950  5.48918  4.45718   235 L - - -
        2.68618  4.42225  2.77519  2.73123  3.46354  2.40513  3.72494  3.29354  2.67741  2.69355  4.24690  2.90347  2.73739  3.18146  2.89801  2.37887  2.77519  2.98518  4.58477  3.61503
        0.01839  4.40100  5.12334  0.61958  0.77255  0.48576  0.95510
  230   2.47811  4.78777  3.08874  2.59746  2.93063  3.49620  3.78164  3.46420  2.62071  3.09404  3.92914  1.83393  3.92665  2.75876  3.06590  2.25852  2.93843  3.15431  5.34764  4.02295   236 n - - -
        2.68618  4.42225  2.77519  2.73123  3.46354  2.40513  3.72494  3.29354  2.67741  2.69355  4.24690  2.90347  2.73739  3.18146  2.89801  2.37887  2.77519  2.98518  4.58477  3.61503
        0.01839  4.40100  5.12334  0.61958  0.77255  0.48576  0.95510
  231   2.02853  4.40134  3.53067  2.97593  3.59230  3.36951  3.45467  2.67986  2.93129  2.66396  3.55309  3.37389  4.00878  3.23903  3.28906  2.11630  2.89846  2.05096  5.02970  3.79253   237 a - - -
        2.68618  4.42225  2.77519  2.73123  3.46354  2.40513  3.72494  3.29354  2.67741  2.69355  4.24690  2.90347  2.73739  3.18146  2.89801  2.37887  2.77519  2.98518  4.58477  3.61503
        0.01839  4.40100  5.12334  0.61958  0.77255  0.48576  0.95510
  232   1.84464  4.19069  4.36066  3.77671  2.52015  3.90958  4.24381  2.09916  3.64336  2.09858  3.27979  3.94183  4.27085  3.84644  3.79037  3.09298  2.99796  1.82318  4.86110  3.67141   238 v - - -
        2.68618  4.42225  2.77519  2.73123  3.46354  2.40513  3.72494  3.29354  2.67741  2.69355  4.24690  2.90347  2.73739  3.18146  2.89801  2.37887  2.77519  2.98518  4.58477  3.61503
        0.01839  4.40100  5.12334  0.61958  0.77255  0.48576  0.95510
  233   3.09153  3.57518  4.90389  4.31848  3.04501  4.33061  4.67597  2.15360  4.16585  1.04216  2.95112  4.43305  4.61617  4.28214  4.23274  3.65727  3.32129  1.81883  5.09787  3.97054   239 l - - -
        2.68618  4.42225  2.77519  2.73123  3.46354  2.40513  3.72494  3.29354  2.67741  2.69355  4.24690  2.90347  2.73739  3.18146  2.89801  2.37887  2.77519  2.98518  4.58477  3.61503
        0.01839  4.40100  5.12334  0.61958  0.77255  0.48576  0.95510
  234   2.43226  4.89902  3.11690  2.54949  4.14736  3.52916  3.71052  3.55774  1.64467  2.71270  3.97155  2.83856  3.91619  2.86006  2.62236  2.74204  2.93553  2.96733  5.38730  3.72841   240 k - - -
        2.68618  4.42225  2.77519  2.73123  3.46354  2.40513  3.72494  3.29354  2.67741  2.69355  4.24690  2.90347  2.73739  3.18146  2.89801  2.37887  2.77519  2.98518  4.58477  3.61503
        0.01839  4.40100  5.12334  0.61958  0.77255  0.48576  0.95510
  235   2.62591  5.01600  2.50784  1.88908  3.84550  3.46923  3.66657  3.74241  2.43311  3.29411  4.07414  2.96649  3.86254  2.71097  2.80281  2.52390  2.65763  3.10785  5.48807  3.07937   241 e - - -
        2.68618  4.42225  2.77519  2.73123  3.46354  2.40513  3.72494  3.29354  2.67741  2.69355  4.24690  2.90347  2.73739  3.18146  2.89801  2.37887  2.77519  2.98518  4.58477  3.61503
        0.01839  4.40100  5.12334  0.61958  0.77255  0.48576  0.95510
  236   1.86075  4.42008  2.86147  2.52833  3.94988  2.92180  3.56778  3.58398  2.53198  3.18214  3.98998  2.78878  3.88682  2.87895  2.99598  2.09123  2.90447  3.24311  5.41847  4.08153   242 a - - -
        2.68618  4.42225  2.77519  2.73123  3.46354  2.40513  3.72494  3.29354  2.67741  2.69355  4.24690  2.90347  2.73739  3.18146  2.89801  2.37887  2.77519  2.98518  4.58477  3.61503
        0.01839  4.40100  5.12334  0.61958  0.77255  0.48576  0.95510
  237   2.26236  4.53034  3.42643  2.98508  3.77277  3.35039  4.08081  3.55212  2.97927  3.22419  4.07391  3.34223  1.32561  3.31411  3.23493  2.29169  2.94082  3.17875  5.50296  4.23522   243 p - - -
        2.68618  4.42225  2.77519  2.73123  3.46354  2.40513  3.72494  3.29354  2.67741  2.69355  4.24690  2.90347  2.73739  3.18146  2.89801  2.37887  2.77519  2.98518  4.58477  3.61503
        0.01839  4.40100  5.12334  0.61958  0.77255  0.48576  0.95510
  238   2.30627  5.07632  2.85109  1.87892  4.38043  3.21951  3.65288  3.57998  2.27849  3.29205  4.12888  2.89891  3.85287  2.70839  2.81840  2.34638  2.74089  3.24416  5.53487  4.15033   244 e - - -
        2.68618  4.42225  2.77519  2.73123  3.46354  2.40513  3.72494  3.29354  2.67741  2.69355  4.24690  2.90347  2.73739  3.18146  2.89801  2.37887  2.77519  2.98518  4.58477  3.61503
        0.01839  4.40100  5.12334  0.61958  0.77255  0.48576  0.95510
  239   2.36546  4.95311  3.02731  2.46873  4.21678  3.48532  2.93849  3.53888  2.21893  2.92848  3.82535  2.99647  3.60760  2.72163  2.52516  2.50111  2.39118  3.29937  5.43698  4.08464   245 k - - -
        2.68618  4.42225  2.77519  2.73123  3.46354  2.40513  3.72494  3.29354  2.67741  2.69355  4.24690  2.90347  2.73739  3.18146  2.89801  2.37887  2.77519  2.98518  4.58477  3.61503
        0.01839  4.40100  5.12334  0.61958  0.77255  0.48576  0.95510
  240   3.48119  4.72616  5.36561  4.83112  3.49163  4.91082  5.36116  1.18327  4.71253  1.18397  3.25827  5.02086  5.08521  4.80993  4.79862  4.29682  3.71746  1.80956  5.60034  4.51673   246 i - - -
        2.68618  4.42225  2.77519  2.73123  3.46354  2.40513  3.72494  3.29354  2.67741  2.69355  4.24690  2.90347  2.73739  3.18146  2.89801  2.37887  2.77519  2.98518  4.58477  3.61503
        0.11021  4.40100  2.38499  0.61958  0.77255  0.48576  0.95510
  241   2.63415  2.82438  4.40244  3.80027  2.35644  3.78136  4.09982  2.13438  3.21749  1.93957  3.07668  3.87646  4.14568  3.79223  3.70220  3.08413  2.86617  2.23555  4.33300  3.09297   247 l - - -
        2.68618  4.42225  2.77519  2.73123  3.46354  2.40513  3.72494  3.29354  2.67741  2.69355  4.24690  2.90347  2.73739  3.18146  2.89801  2.37887  2.77519  2.98518  4.58477  3.61503
        0.02014  4.31093  5.03327  0.61958  0.77255  0.43359  1.04463
  242   2.45512  5.07162  2.79778  2.22768  4.37450  3.07645  3.47897  3.54747  2.36108  3.28888  4.12496  2.34224  3.85125  2.76480  2.89247  2.33877  2.45310  3.35277  5.53147  4.14736   248 e - - -
        2.68618  4.42225  2.77519  2.73123  3.46354  2.40513  3.72494  3.29354  2.67741  2.69355  4.24690  2.90347  2.73739  3.18146  2.89801  2.37887  2.77519  2.98518  4.58477  3.61503
        0.01856  4.39193  5.11427  0.61958  0.77255  0.47717  0.96899
  243   2.50805  5.08515  2.67240  2.23971  4.39306  3.06292  3.65242  3.85300  2.32682  3.18385  3.93673  2.85315  3.16053  2.76322  2.89148  2.12895  2.48575  3.45614  5.54306  4.15611   249 s - - -
        2.68618  4.42225  2.77519  2.73123  3.46354  2.40513  3.72494  3.29354  2.67741  2.69355  4.24690  2.90347  2.73739  3.18146  2.89801  2.37887  2.77519  2.98518  4.58477  3.61503
        0.01839  4.40100  5.12334  0.61958  0.77255  0.48576  0.95510
  244   2.67645  5.15465  2.57114  2.25441  4.48540  2.65483  3.47044  3.96199  2.04549  3.45363  4.19975  2.77398  3.23379  2.74320  2.78723  2.38331  2.83437  3.53771  5.59541  3.69666   250 k - - -
        2.68618  4.42225  2.77519  2.73123  3.46354  2.40513  3.72494  3.29354  2.67741  2.69355  4.24690  2.90347  2.73739  3.18146  2.89801  2.37887  2.77519  2.98518  4.58477  3.61503
        0.03588  4.40100  3.77310  0.61958  0.77255  0.48576  0.95510
  245   2.67006  4.17265  3.82704  3.17842  2.42530  3.34204  4.01973  2.62707  3.34385  1.75473  3.28648  3.69000  4.12229  3.57727  3.55379  2.93405  2.90192  2.43005  4.02862  2.51909   251 l - - -
        2.68618  4.42225  2.77519  2.73123  3.46354  2.40513  3.72494  3.29354  2.67741  2.69355  4.24690  2.90347  2.73739  3.18146  2.89801  2.37887  2.77519  2.98518  4.58477  3.61503
        0.01871  4.38383  5.10617  0.61958  0.77255  0.51179  0.91484
  246   2.35876  4.00861  3.27220  2.39894  3.30794  3.56153  3.65922  2.65957  2.52678  2.71620  3.67715  3.10562  3.64236  3.01834  2.82845  2.39306  2.78342  2.78221  5.13769  3.62723   252 a - - -
        2.68618  4.42225  2.77519  2.73123  3.46354  2.40513  3.72494  3.29354  2.67741  2.69355  4.24690  2.90347  2.73739  3.18146  2.89801  2.37887  2.77519  2.98518  4.58477  3.61503
```

```
        0.01871  4.38383  5.10617  0.61958  0.77255  0.51179  0.91484
247     2.64135  5.24363  2.05992  1.97650  4.57175  3.43623  3.49089  4.05669  2.45970  3.54226  4.29540  2.57855  3.57409  2.67819  2.96419  2.22444  2.77013  3.62642  5.68251  4.26316    253 e - - -
        2.68618  4.42225  2.77519  2.73123  3.46354  2.40513  3.72494  3.29354  2.67741  2.69355  4.24690  2.90347  2.73739  3.18146  2.89801  2.37887  2.77519  2.98518  4.58477  3.61503
        0.01871  4.38383  5.10617  0.61958  0.77255  0.46982  0.98113
248     2.58079  5.14015  2.58713  1.98761  4.46681  3.45103  3.64335  3.76655  2.02254  3.43718  4.18699  2.92614  3.84692  2.74687  2.68402  2.41580  2.45456  3.42744  5.58386  4.18498    254 e - - -
        2.68618  4.42225  2.77519  2.73123  3.46354  2.40513  3.72494  3.29354  2.67741  2.69355  4.24690  2.90347  2.73739  3.18146  2.89801  2.37887  2.77519  2.98518  4.58477  3.61503
        0.01839  4.40100  5.12334  0.61958  0.77255  0.48576  0.95510
249     2.93974  3.87879  4.76842  4.19170  3.01523  4.18989  4.57195  1.84038  4.04189  1.85018  3.35023  4.29147  4.52088  4.21711  4.13490  3.51450  2.77022  1.25283  5.08659  3.90653    255 v - - -
        2.68618  4.42225  2.77519  2.73123  3.46354  2.40513  3.72494  3.29354  2.67741  2.69355  4.24690  2.90347  2.73739  3.18146  2.89801  2.37887  2.77519  2.98518  4.58477  3.61503
        0.01839  4.40100  5.12334  0.61958  0.77255  0.48576  0.95510
250     2.49388  3.37294  3.78055  3.20606  3.36976  3.69558  3.80463  2.46553  3.13286  2.32620  3.26889  3.53721  3.05086  2.99306  3.12463  2.88263  2.74645  1.94709  4.84949  3.41542    256 v - - -
        2.68618  4.42225  2.77519  2.73123  3.46354  2.40513  3.72494  3.29354  2.67741  2.69355  4.24690  2.90347  2.73739  3.18146  2.89801  2.37887  2.77519  2.98518  4.58477  3.61503
        0.01839  4.40100  5.12334  0.61958  0.77255  0.48576  0.95510
251     2.47755  5.17442  2.35571  1.98351  4.50349  3.36503  3.65570  3.98068  2.13747  3.47324  4.22259  2.91917  3.85413  2.64844  2.90051  2.20820  2.81875  3.34606  5.61655  4.21065    257 e - - -
        2.68618  4.42225  2.77519  2.73123  3.46354  2.40513  3.72494  3.29354  2.67741  2.69355  4.24690  2.90347  2.73739  3.18146  2.89801  2.37887  2.77519  2.98518  4.58477  3.61503
        0.01839  4.40100  5.12334  0.61958  0.77255  0.48576  0.95510
252     2.29453  3.44434  4.34128  3.74336  2.62508  3.79232  4.11023  2.37663  3.58547  2.16029  2.85345  3.85789  4.15881  3.76788  3.40031  3.09057  2.45388  2.07050  4.69741  2.72131    258 v - - -
        2.68618  4.42225  2.77519  2.73123  3.46354  2.40513  3.72494  3.29354  2.67741  2.69355  4.24690  2.90347  2.73739  3.18146  2.89801  2.37887  2.77519  2.98518  4.58477  3.61503
        0.01839  4.40100  5.12334  0.61958  0.77255  0.48576  0.95510
253     3.27228  4.56152  5.13091  4.57126  3.17095  4.61730  5.02043  1.11223  4.44521  1.54057  3.07281  4.71873  4.85949  4.56612  4.53051  3.96815  3.33174  1.80348  5.38611  4.26693    259 i - - -
        2.68618  4.42225  2.77519  2.73123  3.46354  2.40513  3.72494  3.29354  2.67741  2.69355  4.24690  2.90347  2.73739  3.18146  2.89801  2.37887  2.77519  2.98518  4.58477  3.61503
        0.01839  4.40100  5.12334  0.61958  0.77255  0.48576  0.95510
254     2.36210  2.82460  4.00191  3.42009  3.11986  3.73721  4.03495  2.43437  3.31769  1.90008  3.28442  3.50845  4.11017  3.55699  3.12823  2.65805  2.59721  2.30847  4.77935  3.58172    260 l - - -
        2.68618  4.42225  2.77519  2.73123  3.46354  2.40513  3.72494  3.29354  2.67741  2.69355  4.24690  2.90347  2.73739  3.18146  2.89801  2.37887  2.77519  2.98518  4.58477  3.61503
        0.01839  4.40100  5.12334  0.61958  0.77255  0.48576  0.95510
255     2.36318  5.15386  2.55061  2.27306  4.48483  3.33410  3.28786  3.96155  2.29906  3.45303  4.19886  2.65076  3.84397  2.42396  2.80444  2.19986  2.73932  3.40012  5.59466  4.19128    261 s - - -
        2.68618  4.42225  2.77519  2.73123  3.46354  2.40513  3.72494  3.29354  2.67741  2.69355  4.24690  2.90347  2.73739  3.18146  2.89801  2.37887  2.77519  2.98518  4.58477  3.61503
        0.01839  4.40100  5.12334  0.61958  0.77255  0.48576  0.95510
256     2.02071  3.83631  3.26726  2.70728  3.60616  2.68033  3.79391  3.17650  2.68589  2.77393  3.70654  2.74243  3.94651  3.01774  2.97797  2.43981  2.78209  2.91163  5.16509  3.40423    262 a - - -
        2.68618  4.42225  2.77519  2.73123  3.46354  2.40513  3.72494  3.29354  2.67741  2.69355  4.24690  2.90347  2.73739  3.18146  2.89801  2.37887  2.77519  2.98518  4.58477  3.61503
        0.01839  4.40100  5.12334  0.61958  0.77255  0.48576  0.95510
257     2.20019  3.49366  4.33423  3.74039  3.11737  3.81693  4.14018  1.85452  3.59043  1.96971  2.65076  3.87163  4.18330  3.78024  3.71012  2.77724  2.78727  2.29167  4.73994  3.55530    263 i - - -
        2.68618  4.42225  2.77519  2.73123  3.46354  2.40513  3.72494  3.29354  2.67741  2.69355  4.24690  2.90347  2.73739  3.18146  2.89801  2.37887  2.77519  2.98518  4.58477  3.61503
        0.01839  4.40100  5.12334  0.61958  0.77255  0.48576  0.95510
258     2.41968  5.05140  2.78239  2.34075  4.34691  3.46394  3.54124  3.79882  2.21869  3.06069  4.10580  2.95410  3.85646  2.49572  2.74499  2.35692  2.31351  3.09645  5.51473  4.13664    264 k - - -
        2.68618  4.42225  2.77519  2.73123  3.46354  2.40513  3.72494  3.29354  2.67741  2.69355  4.24690  2.90347  2.73739  3.18146  2.89801  2.37887  2.77519  2.98518  4.58477  3.61503
        0.01839  4.40100  5.12334  0.61958  0.77255  0.48576  0.95510
259     2.13675  5.12699  2.63714  2.39999  4.44041  3.45372  3.21636  3.90134  2.47507  3.42815  4.20091  2.57221  3.87994  2.62845  2.96503  1.84936  2.96119  3.50616  5.60074  4.20959    265 s - - -
        2.68618  4.42225  2.77519  2.73123  3.46354  2.40513  3.72494  3.29354  2.67741  2.69355  4.24690  2.90347  2.73739  3.18146  2.89801  2.37887  2.77519  2.98518  4.58477  3.61503
        0.01839  4.40100  5.12334  0.61958  0.77255  0.48576  0.95510
260     2.60899  5.21375  1.94659  2.16708  4.54269  3.35687  3.67791  4.02307  2.12089  3.51377  4.26668  2.86242  3.46192  2.78539  2.94319  2.40005  2.62512  3.59731  5.65698  4.24464    266 d - - -
        2.68618  4.42225  2.77519  2.73123  3.46354  2.40513  3.72494  3.29354  2.67741  2.69355  4.24690  2.90347  2.73739  3.18146  2.89801  2.37887  2.77519  2.98518  4.58477  3.61503
        0.01839  4.40100  5.12334  0.61958  0.77255  0.48576  0.95510
261     2.68209  5.16530  2.46046  2.24313  4.50215  3.45300  3.44290  3.52720  2.00676  3.46463  4.20935  2.69306  3.84790  2.60259  2.26689  2.64845  2.73594  3.55178  5.60035  4.19899    267 k - - -
        2.68618  4.42225  2.77519  2.73123  3.46354  2.40513  3.72494  3.29354  2.67741  2.69355  4.24690  2.90347  2.73739  3.18146  2.89801  2.37887  2.77519  2.98518  4.58477  3.61503
        0.01839  4.40100  5.12334  0.61958  0.77255  0.48576  0.95510
262     2.43451  4.25054  3.53527  3.22808  3.02123  3.69986  3.98755  2.09374  3.15497  2.45531  3.36598  3.55350  3.03316  3.42408  3.43761  2.66608  2.62305  2.01247  4.85871  3.64798    268 v - - -
        2.68618  4.42225  2.77519  2.73123  3.46354  2.40513  3.72494  3.29354  2.67741  2.69355  4.24690  2.90347  2.73739  3.18146  2.89801  2.37887  2.77519  2.98518  4.58477  3.61503
        0.01839  4.40100  5.12334  0.61958  0.77255  0.48576  0.95510
263     2.62322  4.51317  3.36953  2.80830  2.60554  3.59756  3.45140  3.04407  2.77597  2.69525  3.51238  2.86082  2.49004  2.94543  3.17119  2.78396  2.82907  2.79995  5.05906  2.83308    269 p - - -
        2.68618  4.42225  2.77519  2.73123  3.46354  2.40513  3.72494  3.29354  2.67741  2.69355  4.24690  2.90347  2.73739  3.18146  2.89801  2.37887  2.77519  2.98518  4.58477  3.61503
        0.01839  4.40100  5.12334  0.61958  0.77255  0.48576  0.95510
264     3.33632  4.60986  5.20807  4.66089  2.63504  4.70511  5.09125  0.98820  4.53872  1.79133  3.28651  4.80708  4.93237  4.65149  4.62219  4.06675  3.57220  1.79067  5.41249  4.24873    270 i - - -
        2.68618  4.42225  2.77519  2.73123  3.46354  2.40513  3.72494  3.29354  2.67741  2.69355  4.24690  2.90347  2.73739  3.18146  2.89801  2.37887  2.77519  2.98518  4.58477  3.61503
        0.01839  4.40100  5.12334  0.61958  0.77255  0.48576  0.95510
265     1.62273  4.58191  3.33393  2.78979  3.93947  3.04112  3.88063  3.20252  2.76204  2.99233  3.84185  3.22758  3.93435  3.09741  2.84572  2.20625  2.64786  2.72391  5.29456  4.01894    271 a - - -
        2.68618  4.42225  2.77519  2.73123  3.46354  2.40513  3.72494  3.29354  2.67741  2.69355  4.24690  2.90347  2.73739  3.18146  2.89801  2.37887  2.77519  2.98518  4.58477  3.61503
        0.01839  4.40100  5.12334  0.61958  0.77255  0.48576  0.95510
266     2.66712  3.89832  2.29163  1.97759  4.14375  3.49151  3.69435  3.24594  2.48705  3.08241  3.32129  3.01524  3.88224  2.77373  2.95433  2.53558  2.72575  3.10717  5.39437  4.05235    272 e - - -
        2.68618  4.42225  2.77519  2.73123  3.46354  2.40513  3.72494  3.29354  2.67741  2.69355  4.24690  2.90347  2.73739  3.18146  2.89801  2.37887  2.77519  2.98518  4.58477  3.61503
        0.01839  4.40100  5.12334  0.61958  0.77255  0.48576  0.95510
267     2.22359  4.70379  3.14945  2.78065  4.52504  2.98603  4.03592  3.97168  2.89307  3.56655  4.36093  1.46042  3.91185  3.20552  3.33376  1.95309  2.68725  3.48704  5.76931  4.45401    273 n - - -
        2.68618  4.42225  2.77519  2.73123  3.46354  2.40513  3.72494  3.29354  2.67741  2.69355  4.24690  2.90347  2.73739  3.18146  2.89801  2.37887  2.77519  2.98518  4.58477  3.61503
        0.01839  4.40100  5.12334  0.61958  0.77255  0.48576  0.95510
268     1.99393  3.79509  3.71880  3.21620  3.17032  1.53928  4.17058  3.34439  3.17763  3.05024  3.91467  3.47945  3.95266  3.47084  3.51945  2.08312  2.89475  3.00094  5.37482  4.14774    274 g - - -
        2.68618  4.42225  2.77519  2.73123  3.46354  2.40513  3.72494  3.29354  2.67741  2.69355  4.24690  2.90347  2.73739  3.18146  2.89801  2.37887  2.77519  2.98518  4.58477  3.61503
        0.01839  4.40100  5.12334  0.61958  0.77255  0.48576  0.95510
269     2.86913  4.42009  4.81153  4.27098  3.70348  4.38198  4.86734  1.37660  4.16332  2.34629  3.56838  4.45072  4.71742  4.40243  4.33930  3.72742  2.43996  1.21509  5.45266  4.24921    275 v - - -
        2.68618  4.42225  2.77519  2.73123  3.46354  2.40513  3.72494  3.29354  2.67741  2.69355  4.24690  2.90347  2.73739  3.18146  2.89801  2.37887  2.77519  2.98518  4.58477  3.61503
        0.01839  4.40100  5.12334  0.61958  0.77255  0.48576  0.95510
270     2.76326  4.52036  3.58996  2.99449  3.65557  3.70626  3.90374  2.43935  2.45860  1.96240  3.58687  3.40367  4.07753  3.18883  1.96003  2.96316  2.98737  2.58926  5.08266  3.85478    276 r - - -
        2.68618  4.42225  2.77519  2.73123  3.46354  2.40513  3.72494  3.29354  2.67741  2.69355  4.24690  2.90347  2.73739  3.18146  2.89801  2.37887  2.77519  2.98518  4.58477  3.61503
        0.01839  4.40100  5.12334  0.61958  0.77255  0.48576  0.95510
271     0.76025  4.35045  3.85420  3.63929  4.70420  2.08859  4.66461  4.11715  3.73180  3.82883  4.64012  3.65129  3.93018  3.97177  3.99233  2.50138  2.94621  3.48126  6.05169  4.88143    277 a - - -
        2.68618  4.42225  2.77519  2.73123  3.46354  2.40513  3.72494  3.29354  2.67741  2.69355  4.24690  2.90347  2.73739  3.18146  2.89801  2.37887  2.77519  2.98518  4.58477  3.61503
        0.01839  4.40100  5.12334  0.61958  0.77255  0.48576  0.95510
```

272	1.82574	3.52282	4.16788	3.58386	3.27020	3.79019	4.11454	2.30877	3.46262	2.02191	2.51038	3.78591	4.16435	3.68311	3.64147	3.00585	2.61626	2.33187	4.79280	3.60172	278 a - - -
	2.68618	4.42225	2.77519	2.73123	3.46354	2.40513	3.72494	3.29354	2.67741	2.69355	4.24690	2.90347	2.73739	3.18146	2.89801	2.37887	2.77519	2.98518	4.58477	3.61503	
	0.01839	4.40100	5.12334	0.61958	0.77255	0.48576	0.95510														
273	2.54256	4.47199	3.71712	3.57267	4.78186	0.72728	4.68334	4.18845	3.71420	3.91267	4.75985	3.66828	4.00615	3.98965	3.97546	2.72753	2.25586	3.57825	6.09389	4.92899	279 g - - -
	2.68618	4.42225	2.77519	2.73123	3.46354	2.40513	3.72494	3.29354	2.67741	2.69355	4.24690	2.90347	2.73739	3.18146	2.89801	2.37887	2.77519	2.98518	4.58477	3.61503	
	0.01839	4.40100	5.12334	0.61958	0.77255	0.48576	0.95510														
274	2.68197	3.85281	3.20099	2.50076	3.07351	3.54948	3.54969	3.30798	1.72502	2.95420	3.79987	3.12899	3.93521	2.94820	2.76692	2.76668	2.91168	3.02689	5.23572	3.10144	280 k - - -
	2.68618	4.42225	2.77519	2.73123	3.46354	2.40513	3.72494	3.29354	2.67741	2.69355	4.24690	2.90347	2.73739	3.18146	2.89801	2.37887	2.77519	2.98518	4.58477	3.61503	
	0.01839	4.40100	5.12334	0.61958	0.77255	0.48576	0.95510														
275	2.48410	3.84552	4.28846	3.71083	3.05745	3.88427	4.14685	2.55553	3.57404	1.35243	3.26226	3.88634	4.24842	3.78024	3.73265	3.18287	3.01415	2.40447	4.73406	2.30420	281 l - - -
	2.68618	4.42225	2.77519	2.73123	3.46354	2.40513	3.72494	3.29354	2.67741	2.69355	4.24690	2.90347	2.73739	3.18146	2.89801	2.37887	2.77519	2.98518	4.58477	3.61503	
	0.01839	4.40100	5.12334	0.61958	0.77255	0.48576	0.95510														
276	3.65511	4.92329	5.39265	4.81104	2.74202	4.95039	5.19337	2.41645	4.67765	0.67851	2.24856	5.03063	5.04177	4.63156	4.69512	4.31084	3.86016	2.51661	5.31915	4.29403	282 L - - -
	2.68618	4.42225	2.77519	2.73123	3.46354	2.40513	3.72494	3.29354	2.67741	2.69355	4.24690	2.90347	2.73739	3.18146	2.89801	2.37887	2.77519	2.98518	4.58477	3.61503	
	0.01839	4.40100	5.12334	0.61958	0.77255	0.48576	0.95510														
277	2.86647	4.90295	3.36752	2.67940	4.14873	3.66842	3.57004	3.52660	2.13519	1.59264	3.98400	3.22874	4.04264	2.50156	2.35641	2.93207	3.07506	3.24396	5.36018	4.10692	283 l - - -
	2.68618	4.42225	2.77519	2.73123	3.46354	2.40513	3.72494	3.29354	2.67741	2.69355	4.24690	2.90347	2.73739	3.18146	2.89801	2.37887	2.77519	2.98518	4.58477	3.61503	
	0.05338	4.40100	3.22608	0.61958	0.77255	0.48576	0.95510														
278	2.46047	4.18176	3.52876	2.96161	3.36926	3.62903	2.90564	2.81931	2.91444	2.23886	3.47548	3.28701	4.00913	3.21713	3.26756	2.32583	2.67840	2.64530	4.94884	2.61670	284 l - - -
	2.68618	4.42225	2.77519	2.73123	3.46354	2.40513	3.72494	3.29354	2.67741	2.69355	4.24690	2.90347	2.73739	3.18146	2.89801	2.37887	2.77519	2.98518	4.58477	3.61503	
	0.01904	4.36666	5.08900	0.61958	0.77255	0.53673	0.87864														
279	2.61829	4.57583	2.65927	2.05072	4.33764	3.22182	2.85562	3.56572	2.40332	3.05683	3.83576	2.94272	3.53097	2.59449	2.68542	2.64668	2.81681	3.40573	5.50517	3.59206	285 e - - -
	2.68618	4.42225	2.77519	2.73123	3.46354	2.40513	3.72494	3.29354	2.67741	2.69355	4.24690	2.90347	2.73739	3.18146	2.89801	2.37887	2.77519	2.98518	4.58477	3.61503	
	0.01904	4.36666	5.08900	0.61958	0.77255	0.53673	0.87864														
280	2.44581	4.54991	3.10897	2.73635	3.71747	3.06285	3.66694	2.57693	2.52690	2.48769	2.77982	3.08767	3.95016	2.86782	3.12170	2.58080	2.78212	2.73861	5.10625	3.83741	286 a - - -
	2.68618	4.42225	2.77519	2.73123	3.46354	2.40513	3.72494	3.29354	2.67741	2.69355	4.24690	2.90347	2.73739	3.18146	2.89801	2.37887	2.77519	2.98518	4.58477	3.61503	
	0.01904	4.36666	5.08900	0.61958	0.77255	0.53673	0.87864														
281	2.61785	5.02412	2.96967	2.03734	4.31373	3.37724	3.65053	3.21958	2.30590	3.17822	3.79282	2.76030	3.11251	2.54648	2.79768	2.34766	2.78649	3.30437	5.49137	4.11724	287 e - - -
	2.68618	4.42225	2.77519	2.73123	3.46354	2.40513	3.72494	3.29354	2.67741	2.69355	4.24690	2.90347	2.73739	3.18146	2.89801	2.37887	2.77519	2.98518	4.58477	3.61503	
	0.17860	4.36666	1.89136	0.61958	0.77255	0.53673	0.87864														
282	2.55679	5.07188	2.48019	2.26894	4.38514	3.40258	3.60701	3.84992	2.29138	3.26806	4.12360	2.66518	3.55837	2.49592	2.73253	2.57017	2.24950	3.44582	5.52315	4.13030	288 t - - -
	2.68618	4.42225	2.77519	2.73123	3.46354	2.40513	3.72494	3.29354	2.67741	2.69355	4.24690	2.90347	2.73739	3.18146	2.89801	2.37887	2.77519	2.98518	4.58477	3.61503	
	0.13485	4.21035	2.19545	0.61958	0.77255	0.57967	0.82117														
283	2.55622	5.13189	2.59267	2.09817	4.46281	3.39890	3.60687	3.93364	1.86899	3.42538	4.18284	2.65842	3.81174	2.71461	2.49075	2.57129	2.83700	3.51623	5.56612	4.16980	289 k - - -
	2.68618	4.42225	2.77519	2.73123	3.46354	2.40513	3.72494	3.29354	2.67741	2.69355	4.24690	2.90347	2.73739	3.18146	2.89801	2.37887	2.77519	2.98518	4.58477	3.61503	
	0.10131	4.18037	2.51261	0.61958	0.77255	0.75327	0.63644														
284	2.54253	4.66482	2.93199	2.51549	3.25714	3.41526	3.68314	3.31681	2.44094	2.95429	3.79020	2.94180	3.21238	2.86453	2.96952	1.97398	2.67713	3.01494	5.23098	3.92187	290 s - - -
	2.68613	4.42260	2.77508	2.73098	3.46206	2.40529	3.72515	3.29341	2.67734	2.69385	4.24700	2.90369	2.73708	3.18181	2.89831	2.37904	2.77524	2.98504	4.58512	3.61495	
	0.46885	1.06901	3.47599	1.68647	0.20478	0.32186	1.29027														
285	2.90405	5.43329	2.46555	1.43077	4.75161	2.70671	3.80217	4.24816	2.59801	3.73514	4.51955	2.07993	3.94507	2.93255	3.20091	2.54813	3.16036	3.81720	5.88487	4.43391	306 e - - -
	2.68618	4.42225	2.77519	2.73123	3.46354	2.40513	3.72494	3.29354	2.67741	2.69355	4.24690	2.90347	2.73739	3.18146	2.89801	2.37887	2.77519	2.98518	4.58477	3.61503	
	0.01871	4.38383	5.10617	0.61958	0.77255	0.51179	0.91484														
286	2.60530	5.22031	2.27717	2.08549	4.54906	2.68271	3.33124	4.03139	2.44668	3.52021	4.27286	2.38558	3.71733	2.78365	2.94852	2.37514	2.76851	3.60368	5.66240	4.24711	307 e - - -
	2.68618	4.42225	2.77519	2.73123	3.46354	2.40513	3.72494	3.29354	2.67741	2.69355	4.24690	2.90347	2.73739	3.18146	2.89801	2.37887	2.77519	2.98518	4.58477	3.61503	
	0.01871	4.38383	5.10617	0.61958	0.77255	0.51179	0.91484														
287	2.47490	4.83132	2.87745	2.31513	4.06138	3.12479	3.70595	2.79736	2.51502	2.83053	3.90510	2.98382	3.88898	2.80114	2.79590	2.50236	2.31212	3.00721	5.34049	4.01247	308 t - - -
	2.68618	4.42225	2.77519	2.73123	3.46354	2.40513	3.72494	3.29354	2.67741	2.69355	4.24690	2.90347	2.73739	3.18146	2.89801	2.37887	2.77519	2.98518	4.58477	3.61503	
	0.01871	4.38383	5.10617	0.61958	0.77255	0.51179	0.91484														
288	2.62418	5.10125	2.83933	2.27847	4.41695	3.36544	3.63982	3.71655	1.85584	3.22799	3.93692	2.64915	3.42865	2.60722	2.80126	2.46659	2.67786	3.29256	5.55170	4.15984	309 k - - -
	2.68618	4.42225	2.77519	2.73123	3.46354	2.40513	3.72494	3.29354	2.67741	2.69355	4.24690	2.90347	2.73739	3.18146	2.89801	2.37887	2.77519	2.98518	4.58477	3.61503	
	0.01871	4.38383	5.10617	0.61958	0.77255	0.51179	0.91484														
289	2.53085	4.39546	3.50144	2.67289	3.17261	3.62611	3.87635	2.72572	2.66006	2.06841	3.19106	3.23850	3.37849	3.12082	3.14165	2.69731	2.81755	2.32144	4.97464	3.73661	310 l - - -
	2.68618	4.42225	2.77519	2.73123	3.46354	2.40513	3.72494	3.29354	2.67741	2.69355	4.24690	2.90347	2.73739	3.18146	2.89801	2.37887	2.77519	2.98518	4.58477	3.61503	
	0.01871	4.38383	5.10617	0.61958	0.77255	0.46982	0.98113														
290	2.66147	4.28896	3.33352	3.13688	3.41112	3.68265	3.95248	2.36965	3.07299	1.92387	3.25159	3.49238	2.75631	3.35415	3.38066	2.59517	2.89353	2.35260	4.88387	3.22359	311 l - - -
	2.68618	4.42225	2.77519	2.73123	3.46354	2.40513	3.72494	3.29354	2.67741	2.69355	4.24690	2.90347	2.73739	3.18146	2.89801	2.37887	2.77519	2.98518	4.58477	3.61503	
	0.01839	4.40100	5.12334	0.61958	0.77255	0.48576	0.95510														
291	2.61908	4.23466	3.82710	3.25115	2.51088	3.70740	3.98660	2.25813	2.87649	2.30064	3.14233	3.56758	3.08534	2.75992	3.44444	2.97397	2.89177	2.22939	4.83453	3.43248	312 v - - -
	2.68618	4.42225	2.77519	2.73123	3.46354	2.40513	3.72494	3.29354	2.67741	2.69355	4.24690	2.90347	2.73739	3.18146	2.89801	2.37887	2.77519	2.98518	4.58477	3.61503	
	0.01839	4.40100	5.12334	0.61958	0.77255	0.48576	0.95510														
292	2.67244	5.14619	2.72308	1.90176	4.47654	3.44926	3.63860	3.62028	2.16153	3.44469	4.19115	2.81626	3.54224	2.64124	2.48808	2.51239	2.59888	3.37505	5.58723	4.18607	313 e - - -
	2.68618	4.42225	2.77519	2.73123	3.46354	2.40513	3.72494	3.29354	2.67741	2.69355	4.24690	2.90347	2.73739	3.18146	2.89801	2.37887	2.77519	2.98518	4.58477	3.61503	
	0.01839	4.40100	5.12334	0.61958	0.77255	0.48576	0.95510														
293	2.19400	4.45596	3.43492	2.51996	3.60570	3.61400	3.85745	2.44071	2.73067	2.22362	3.55577	3.30507	3.99592	2.90540	3.21134	2.73052	2.66858	2.45385	5.02932	3.78152	314 a - - -
	2.68618	4.42225	2.77519	2.73123	3.46354	2.40513	3.72494	3.29354	2.67741	2.69355	4.24690	2.90347	2.73739	3.18146	2.89801	2.37887	2.77519	2.98518	4.58477	3.61503	
	0.01839	4.40100	5.12334	0.61958	0.77255	0.48576	0.95510														
294	2.88022	4.38048	4.52212	3.95759	3.38640	4.11965	4.50218	2.07954	3.82012	1.09848	3.29233	4.15435	4.46918	4.03697	3.98528	3.01718	3.20377	1.93754	5.09844	3.92924	315 l - - -
	2.68618	4.42225	2.77519	2.73123	3.46354	2.40513	3.72494	3.29354	2.67741	2.69355	4.24690	2.90347	2.73739	3.18146	2.89801	2.37887	2.77519	2.98518	4.58477	3.61503	
	0.01839	4.40100	5.12334	0.61958	0.77255	0.48576	0.95510														
295	1.96612	4.16926	3.15998	2.61171	4.10605	3.21776	3.78090	3.51851	2.54314	3.13731	3.95660	2.90511	3.53704	2.94620	2.88511	1.95305	2.50686	3.18296	5.39208	4.07555	316 s - - -
	2.68618	4.42225	2.77519	2.73123	3.46354	2.40513	3.72494	3.29354	2.67741	2.69355	4.24690	2.90347	2.73739	3.18146	2.89801	2.37887	2.77519	2.98518	4.58477	3.61503	
	0.01839	4.40100	5.12334	0.61958	0.77255	0.48576	0.95510														
296	2.49384	3.90583	3.06766	2.43900	3.86641	3.26541	3.72605	3.29849	2.19927	2.58498	3.86069	3.01578	2.96715	2.67655	3.00351	2.66037	2.77071	2.79592	5.30195	3.98578	317 k - - -
	2.68618	4.42225	2.77519	2.73123	3.46354	2.40513	3.72494	3.29354	2.67741	2.69355	4.24690	2.90347	2.73739	3.18146	2.89801	2.37887	2.77519	2.98518	4.58477	3.61503	
	0.01839	4.40100	5.12334	0.61958	0.77255	0.48576	0.95510														
297	2.38547	3.85297	4.20092	3.61138	2.33915	3.77515	4.08829	2.29380	3.47949	1.83118	3.23460	3.41094	4.14518	3.68763	3.63559	2.61642	2.84043	2.22524	4.73660	3.54739	318 l - - -

```
        2.68618  4.42225  2.77519  2.73123  3.46354  2.40513  3.72494  3.29354  2.67741  2.69355  4.24690  2.90347  2.73739  3.18146  2.89801  2.37887  2.77519  2.98518  4.58477  3.61503
        0.01839  4.40100  5.12334  0.61958  0.77255  0.48576  0.95510
298     2.72102  4.45221  4.97779  4.43923  3.72107  4.52033  5.00624  1.33067  4.33130  2.28192  3.32112  4.60188  4.82263  4.55640  4.48605  3.87340  3.22206  1.11250  5.52670  4.32871    319 v - - -
        2.68618  4.42225  2.77519  2.73123  3.46354  2.40513  3.72494  3.29354  2.67741  2.69355  4.24690  2.90347  2.73739  3.18146  2.89801  2.37887  2.77519  2.98518  4.58477  3.61503
        0.18149  4.40100  1.87270  0.61958  0.77255  0.48576  0.95510
299     2.67765  4.93979  3.04286  2.26825  4.21555  3.47820  3.64518  3.25005  1.93722  2.72255  4.00474  2.98997  3.86453  2.70557  2.29813  2.59086  2.77747  3.29317  5.40459  4.07166    320 k - - -
        2.68618  4.42225  2.77519  2.73123  3.46354  2.40513  3.72494  3.29354  2.67741  2.69355  4.24690  2.90347  2.73739  3.18146  2.89801  2.37887  2.77519  2.98518  4.58477  3.61503
        0.02161  4.24112  4.96347  0.61958  0.77255  0.69187  0.69443
300     2.34974  3.15483  3.15757  2.55574  3.85584  3.16979  3.72769  3.24696  2.43684  2.89992  3.74617  3.09037  3.88183  2.56741  3.01305  2.21502  2.85398  2.73113  5.19560  3.90257    321 s - - -
        2.68618  4.42225  2.77519  2.73123  3.46354  2.40513  3.72494  3.29354  2.67741  2.69355  4.24690  2.90347  2.73739  3.18146  2.89801  2.37887  2.77519  2.98518  4.58477  3.61503
        0.02161  4.24112  4.96347  0.61958  0.77255  0.37558  1.16120
301     2.61981  4.53715  3.33653  2.66585  3.70070  3.49113  3.81783  2.86966  2.65431  2.29559  2.83657  3.23314  2.55187  2.85028  3.14574  2.60498  2.67797  2.82593  5.09727  3.83369    322 l - - -
        2.68618  4.42225  2.77519  2.73123  3.46354  2.40513  3.72494  3.29354  2.67741  2.69355  4.24690  2.90347  2.73739  3.18146  2.89801  2.37887  2.77519  2.98518  4.58477  3.61503
        0.01839  4.40100  5.12334  0.61958  0.77255  0.48576  0.95510
302     2.51689  5.11972  2.93796  2.33908  4.43560  3.46298  3.43794  3.89782  2.33252  3.41008  4.17131  2.22234  2.57339  2.36921  2.76360  2.60258  2.92274  3.36469  5.56819  4.18054    323 n - - -
        2.68618  4.42225  2.77519  2.73123  3.46354  2.40513  3.72494  3.29354  2.67741  2.69355  4.24690  2.90347  2.73739  3.18146  2.89801  2.37887  2.77519  2.98518  4.58477  3.61503
        0.01839  4.40100  5.12334  0.61958  0.77255  0.48576  0.95510
303     2.77085  5.20561  2.53655  2.38244  4.53980  2.44752  2.63154  4.01409  2.53706  3.52838  4.30185  2.30344  3.90044  2.85753  3.03890  2.02097  3.01889  3.60336  5.69088  4.28311    324 s - - -
        2.68618  4.42225  2.77519  2.73123  3.46354  2.40513  3.72494  3.29354  2.67741  2.69355  4.24690  2.90347  2.73739  3.18146  2.89801  2.37887  2.77519  2.98518  4.58477  3.61503
        0.01839  4.40100  5.12334  0.61958  0.77255  0.48576  0.95510
304     2.53382  5.15570  2.81530  2.30808  4.48894  2.93468  3.41686  3.96666  2.18535  3.45570  4.19996  2.42293  3.11849  2.44525  2.73749  2.44728  2.84039  3.43240  5.59514  4.19126    325 k - - -
        2.68618  4.42225  2.77519  2.73123  3.46354  2.40513  3.72494  3.29354  2.67741  2.69355  4.24690  2.90347  2.73739  3.18146  2.89801  2.37887  2.77519  2.98518  4.58477  3.61503
        0.01839  4.40100  5.12334  0.61958  0.77255  0.48576  0.95510
305     2.27799  4.06998  3.62091  3.16824  4.41818  3.22933  4.24391  3.83937  3.15423  3.49258  4.30356  3.22641  2.34909  3.46691  3.52622  1.11481  2.74944  3.34298  5.73478  4.49243    326 s - - -
        2.68618  4.42225  2.77519  2.73123  3.46354  2.40513  3.72494  3.29354  2.67741  2.69355  4.24690  2.90347  2.73739  3.18146  2.89801  2.37887  2.77519  2.98518  4.58477  3.61503
        0.01839  4.40100  5.12334  0.61958  0.77255  0.48576  0.95510
306     2.53243  4.89894  3.04716  2.49565  4.15797  3.48727  3.55084  3.58061  2.49052  3.01065  3.97839  2.35330  3.45308  2.66751  2.95637  2.19739  2.36409  2.90831  5.40541  4.06317    327 s - - -
        2.68618  4.42225  2.77519  2.73123  3.46354  2.40513  3.72494  3.29354  2.67741  2.69355  4.24690  2.90347  2.73739  3.18146  2.89801  2.37887  2.77519  2.98518  4.58477  3.61503
        0.01839  4.40100  5.12334  0.61958  0.77255  0.48576  0.95510
307     3.30423  5.98219  0.86507  1.65223  5.25765  3.43569  4.06126  4.82655  3.18204  4.29096  5.18118  2.88896  4.10639  3.24113  3.84788  3.14541  3.61269  4.36757  6.42480  4.85856    328 d - - -
        2.68618  4.42225  2.77519  2.73123  3.46354  2.40513  3.72494  3.29354  2.67741  2.69355  4.24690  2.90347  2.73739  3.18146  2.89801  2.37887  2.77519  2.98518  4.58477  3.61503
        0.01839  4.40100  5.12334  0.61958  0.77255  0.48576  0.95510
308     2.56745  3.91210  3.98719  3.41149  3.33693  3.74082  4.06621  2.10817  3.31534  2.36492  3.33248  3.67656  4.12588  3.38333  3.54993  2.46497  2.28286  1.81650  4.84094  3.64032    329 v - - -
        2.68618  4.42225  2.77519  2.73123  3.46354  2.40513  3.72494  3.29354  2.67741  2.69355  4.24690  2.90347  2.73739  3.18146  2.89801  2.37887  2.77519  2.98518  4.58477  3.61503
        0.01839  4.40100  5.12334  0.61958  0.77255  0.48576  0.95510
309     3.44955  5.51518  3.99640  3.17159  5.08657  3.99924  3.81167  4.37432  1.31133  3.73132  4.61824  3.51506  4.33157  2.93914  1.03747  3.42673  3.56916  4.06262  5.69689  4.60489    330 r - - -
        2.68618  4.42225  2.77519  2.73123  3.46354  2.40513  3.72494  3.29354  2.67741  2.69355  4.24690  2.90347  2.73739  3.18146  2.89801  2.37887  2.77519  2.98518  4.58477  3.61503
        0.01839  4.40100  5.12334  0.61958  0.77255  0.48576  0.95510
310     3.07467  5.16622  3.55873  2.89575  4.45151  3.78829  3.78382  3.84266  2.16879  2.47727  4.22831  3.32411  4.15072  2.20916  1.26693  3.10015  3.25969  3.55862  5.50802  4.28293    331 r - - -
        2.68618  4.42225  2.77519  2.73123  3.46354  2.40513  3.72494  3.29354  2.67741  2.69355  4.24690  2.90347  2.73739  3.18146  2.89801  2.37887  2.77519  2.98518  4.58477  3.61503
        0.01839  4.40100  5.12334  0.61958  0.77255  0.48576  0.95510
311     3.02249  4.42335  4.59506  4.01312  3.32770  4.20371  4.52829  1.93087  3.84384  1.15380  3.21400  4.21528  4.51572  4.06004  3.26366  3.51649  3.25422  1.88235  5.08508  3.93556    332 l - - -
        2.68618  4.42225  2.77519  2.73123  3.46354  2.40513  3.72494  3.29354  2.67741  2.69355  4.24690  2.90347  2.73739  3.18146  2.89801  2.37887  2.77519  2.98518  4.58477  3.61503
        0.01839  4.40100  5.12334  0.61958  0.77255  0.48576  0.95510
312     1.46570  4.38498  3.67592  3.14954  3.77865  3.47820  4.08662  3.03563  3.09871  2.43680  3.72752  3.47167  4.00731  3.40290  3.44009  2.13127  2.68960  2.42815  5.21975  3.99124    333 a - - -
        2.68618  4.42225  2.77519  2.73123  3.46354  2.40513  3.72494  3.29354  2.67741  2.69355  4.24690  2.90347  2.73739  3.18146  2.89801  2.37887  2.77519  2.98518  4.58477  3.61503
        0.01839  4.40100  5.12334  0.61958  0.77255  0.48576  0.95510
313     2.07796  4.57915  3.37799  2.35430  3.73612  3.64872  3.99061  2.96207  2.92214  1.50839  3.66531  3.37331  4.08525  3.26887  3.28282  2.95879  3.03110  2.75912  5.20733  3.95864    334 l - - -
        2.68618  4.42225  2.77519  2.73123  3.46354  2.40513  3.72494  3.29354  2.67741  2.69355  4.24690  2.90347  2.73739  3.18146  2.89801  2.37887  2.77519  2.98518  4.58477  3.61503
        0.01839  4.40100  5.12334  0.61958  0.77255  0.48576  0.95510
314     2.69905  4.54779  3.40354  2.82930  3.71535  3.61795  3.83281  2.69668  2.23357  2.70197  3.64484  3.27470  3.99892  2.97125  2.65283  2.63504  2.92755  1.84003  5.10445  3.85175    335 v - - -
        2.68618  4.42225  2.77519  2.73123  3.46354  2.40513  3.72494  3.29354  2.67741  2.69355  4.24690  2.90347  2.73739  3.18146  2.89801  2.37887  2.77519  2.98518  4.58477  3.61503
        0.01839  4.40100  5.12334  0.61958  0.77255  0.48576  0.95510
315     2.48091  4.17175  4.11142  3.53035  3.29596  3.35779  4.10383  2.30177  3.41838  2.26550  2.79646  3.75555  4.15847  3.64932  3.61665  2.80507  2.67461  1.54691  4.81237  3.61740    336 v - - -
        2.68618  4.42225  2.77519  2.73123  3.46354  2.40513  3.72494  3.29354  2.67741  2.69355  4.24690  2.90347  2.73739  3.18146  2.89801  2.37887  2.77519  2.98518  4.58477  3.61503
        0.01839  4.40100  5.12334  0.61958  0.77255  0.48576  0.95510
316     2.96127  2.79131  4.94183  4.37843  3.51469  4.35034  4.77581  1.48486  4.23326  1.62981  3.40427  4.47283  4.66550  4.40704  4.32215  3.69055  3.29023  1.53616  5.25322  4.07687    337 i - - -
        2.68618  4.42225  2.77519  2.73123  3.46354  2.40513  3.72494  3.29354  2.67741  2.69355  4.24690  2.90347  2.73739  3.18146  2.89801  2.37887  2.77519  2.98518  4.58477  3.61503
        0.01839  4.40100  5.12334  0.61958  0.77255  0.48576  0.95510
317     2.72959  4.90920  3.14907  2.57338  4.15864  3.54754  3.22140  3.22845  2.27923  3.16134  3.98096  2.83311  3.93133  2.77160  1.82112  2.69050  2.87405  3.24985  3.27260  4.07413    338 r - - -
        2.68618  4.42225  2.77519  2.73123  3.46354  2.40513  3.72494  3.29354  2.67741  2.69355  4.24690  2.90347  2.73739  3.18146  2.89801  2.37887  2.77519  2.98518  4.58477  3.61503
        0.01839  4.40100  5.12334  0.61958  0.77255  0.48576  0.95510
318     2.47906  4.27636  3.75760  3.18482  3.35848  3.69726  3.25871  2.62704  3.10954  2.43420  3.38543  3.52388  4.07273  3.38814  3.40278  2.95925  1.87143  2.48748  3.10838  2.91922    339 t - - -
        2.68618  4.42225  2.77519  2.73123  3.46354  2.40513  3.72494  3.29354  2.67741  2.69355  4.24690  2.90347  2.73739  3.18146  2.89801  2.37887  2.77519  2.98518  4.58477  3.61503
        0.01839  4.40100  5.12334  0.61958  0.77255  0.48576  0.95510
319     2.59779  4.46816  4.91584  4.36542  3.56844  4.45505  4.89593  1.78070  4.24523  1.44927  3.41529  4.53210  4.75543  4.44099  4.38494  3.79949  3.38873  1.28233  5.39292  4.22589    340 v - - -
        2.68618  4.42225  2.77519  2.73123  3.46354  2.40513  3.72494  3.29354  2.67741  2.69355  4.24690  2.90347  2.73739  3.18146  2.89801  2.37887  2.77519  2.98518  4.58477  3.61503
        0.01839  4.40100  5.12334  0.61958  0.77255  0.48576  0.95510
320     1.55091  4.22068  3.41909  2.86677  3.40075  3.53776  3.88582  3.10672  2.83319  2.79886  3.67195  2.85542  3.97227  3.15421  3.22270  2.22337  2.89858  2.84493  4.65519  3.86718    341 a - - -
        2.68618  4.42225  2.77519  2.73123  3.46354  2.40513  3.72494  3.29354  2.67741  2.69355  4.24690  2.90347  2.73739  3.18146  2.89801  2.37887  2.77519  2.98518  4.58477  3.61503
        0.01839  4.40100  5.12334  0.61958  0.77255  0.48576  0.95510
321     3.08256  5.30303  3.50088  2.82340  4.71923  3.77073  3.45579  4.08936  1.52080  3.54063  4.37194  3.10919  4.12652  2.86561  1.34203  3.06888  3.25983  3.23951  5.60750  4.38855    342 r - - -
        2.68618  4.42225  2.77519  2.73123  3.46354  2.40513  3.72494  3.29354  2.67741  2.69355  4.24690  2.90347  2.73739  3.18146  2.89801  2.37887  2.77519  2.98518  4.58477  3.61503
        0.01839  4.40100  5.12334  0.61958  0.77255  0.48576  0.95510
322     2.40956  4.94693  2.73775  2.06920  3.44289  3.48145  3.68038  3.63908  2.37668  2.85347  3.70914  2.81915  3.87241  2.81398  2.69869  2.55585  2.70280  3.01270  5.43328  4.07946    343 e - - -
        2.68618  4.42225  2.77519  2.73123  3.46354  2.40513  3.72494  3.29354  2.67741  2.69355  4.24690  2.90347  2.73739  3.18146  2.89801  2.37887  2.77519  2.98518  4.58477  3.61503
```

```
        0.03369  4.40100  3.86955  0.61958  0.77255  0.48576  0.95510
 323    2.62659  5.09918  2.69460  2.34539  4.41305  3.45156  3.13069  3.59399  1.99095  2.99650  4.14850  2.38924  3.22686  2.66913  2.87454  2.64519  2.89857  3.26586  5.55046  4.15986    344 k - - -
        2.68618  4.42225  2.77519  2.73123  3.46354  2.40513  3.72494  3.29354  2.67741  2.69355  4.24690  2.90347  2.73739  3.18146  2.89801  2.37887  2.77519  2.98518  4.58477  3.61503
        0.05421  4.38597  3.21105  0.61958  0.77255  0.50860  0.91964
 324    2.47458  4.59333  2.99410  2.68514  2.95420  3.54689  3.77212  3.09546  2.66422  2.63677  3.31766  2.80843  2.68473  2.88175  3.02505  2.64234  2.87986  2.89014  5.14185  3.24855    345 a - - -
        2.68618  4.42225  2.77519  2.73123  3.46354  2.40513  3.72494  3.29354  2.67741  2.69355  4.24690  2.90347  2.73739  3.18146  2.89801  2.37887  2.77519  2.98518  4.58477  3.61503
        0.03775  4.35111  3.72321  0.61958  0.77255  0.55845  0.84886
 325    2.60882  5.09002  2.23156  2.13093  4.40373  3.31030  3.63122  3.55061  2.32900  3.27520  4.14020  2.91534  3.15025  2.60867  2.87000  2.39020  2.74787  3.31407  5.54224  4.14996    346 e - - -
        2.68618  4.42225  2.77519  2.73123  3.46354  2.40513  3.72494  3.29354  2.67741  2.69355  4.24690  2.90347  2.73739  3.18146  2.89801  2.37887  2.77519  2.98518  4.58477  3.61503
        0.01970  4.33305  5.05539  0.61958  0.77255  0.54582  0.86600
 326    2.43444  4.76532  2.87879  2.09853  3.98124  3.34262  3.71309  3.18365  2.53940  2.47556  3.19601  3.05714  3.07690  2.88412  2.99154  2.63195  2.88261  2.99383  5.28551  3.97003    347 e - - -
        2.68615  4.42149  2.77525  2.73129  3.46360  2.40519  3.72500  3.29324  2.67725  2.69361  4.24696  2.90348  2.73739  3.18152  2.89807  2.37891  2.77515  2.98520  4.58483  3.61509
        0.35718  1.22392  5.07123  0.16138  1.90360  0.44116  1.03082
 327    2.63654  4.18998  4.47367  3.88651  3.30362  3.96165  4.30162  1.95267  3.73939  1.88616  2.37093  3.73732  4.31506  3.93106  3.86012  3.26757  2.83867  1.63716  4.88360  3.70056    349 v - - -
        2.68618  4.42225  2.77519  2.73123  3.46354  2.40513  3.72494  3.29354  2.67741  2.69355  4.24690  2.90347  2.73739  3.18146  2.89801  2.37887  2.77519  2.98518  4.58477  3.61503
        0.01839  4.40100  5.12334  0.61958  0.77255  0.48576  0.95510
 328    2.50620  5.14414  2.63879  2.26311  4.47283  3.32755  3.31087  3.94646  2.17944  3.26780  4.18995  2.92510  3.46862  2.74388  2.29701  2.16331  2.90668  3.52612  5.58595  4.18631    350 s - - -
        2.68618  4.42225  2.77519  2.73123  3.46354  2.40513  3.72494  3.29354  2.67741  2.69355  4.24690  2.90347  2.73739  3.18146  2.89801  2.37887  2.77519  2.98518  4.58477  3.61503
        0.06328  4.40100  3.01491  0.61958  0.77255  0.48576  0.95510
 329    2.53190  4.87124  2.87149  2.52430  4.18403  3.45936  3.74691  3.46951  2.54801  3.20309  4.01811  3.03605  1.76912  2.68081  2.93307  2.58816  2.75915  3.26300  5.44054  4.10641    351 p - - -
        2.68624  4.42231  2.77511  2.73101  3.46360  2.40519  3.72501  3.29360  2.67747  2.69361  4.24696  2.90353  2.73742  3.18117  2.89807  2.37882  2.77526  2.98509  4.58483  3.61509
        0.36449  1.20656  5.07929  0.15825  1.92168  0.44737  1.01972
 330    2.62711  4.48323  3.40466  2.84065  3.45829  3.60649  2.38423  2.94479  2.80339  2.42209  3.58099  3.01811  3.98859  2.85738  3.19018  2.84025  2.69908  2.54185  5.03970  2.69798    353 h - - -
        2.68618  4.42225  2.77519  2.73123  3.46354  2.40513  3.72494  3.29354  2.67741  2.69355  4.24690  2.90347  2.73739  3.18146  2.89801  2.37887  2.77519  2.98518  4.58477  3.61503
        0.01839  4.40100  5.12334  0.61958  0.77255  0.48576  0.95510
 331    2.62100  4.12223  4.23122  3.63993  3.12670  3.27806  4.10060  1.93160  3.50161  1.62608  2.96197  3.80948  4.15774  3.70708  3.41655  3.08372  2.84750  2.35413  4.73613  3.07992    354 l - - -
        2.68618  4.42225  2.77519  2.73123  3.46354  2.40513  3.72494  3.29354  2.67741  2.69355  4.24690  2.90347  2.73739  3.18146  2.89801  2.37887  2.77519  2.98518  4.58477  3.61503
        0.01839  4.40100  5.12334  0.61958  0.77255  0.48576  0.95510
 332    2.54692  5.15802  2.40925  2.37650  4.49088  3.29757  3.64734  3.96676  1.86318  3.45914  4.20696  2.74427  3.13235  2.75020  2.68915  2.47356  2.70625  3.54316  5.60141  4.19888    355 k - - -
        2.68618  4.42225  2.77519  2.73123  3.46354  2.40513  3.72494  3.29354  2.67741  2.69355  4.24690  2.90347  2.73739  3.18146  2.89801  2.37887  2.77519  2.98518  4.58477  3.61503
        0.01839  4.40100  5.12334  0.61958  0.77255  0.48576  0.95510
 333    2.40810  4.22141  3.87684  3.30027  3.18532  3.72085  4.00767  2.35577  3.21558  1.78980  3.33039  3.60145  2.96545  3.15140  3.47533  2.91165  2.72074  2.21830  4.82825  3.62320    356 l - - -
        2.68618  4.42225  2.77519  2.73123  3.46354  2.40513  3.72494  3.29354  2.67741  2.69355  4.24690  2.90347  2.73739  3.18146  2.89801  2.37887  2.77519  2.98518  4.58477  3.61503
        0.01839  4.40100  5.12334  0.61958  0.77255  0.48576  0.95510
 334    3.16420  4.62693  5.19307  4.64713  3.49524  4.72046  5.15846  1.52875  4.52495  1.08718  3.28620  4.82003  4.94761  4.65540  4.62560  4.08521  3.58676  1.65985  5.49940  4.39184    357 l - - -
        2.68618  4.42225  2.77519  2.73123  3.46354  2.40513  3.72494  3.29354  2.67741  2.69355  4.24690  2.90347  2.73739  3.18146  2.89801  2.37887  2.77519  2.98518  4.58477  3.61503
        0.01839  4.40100  5.12334  0.61958  0.77255  0.48576  0.95510
 335    2.08803  4.22610  4.08212  3.51012  3.37537  2.80174  4.15063  2.09020  3.41129  2.00814  3.35762  3.76190  4.18927  3.65687  3.63672  3.09614  2.83911  1.88379  4.90323  3.70386    358 v - - -
        2.68618  4.42225  2.77519  2.73123  3.46354  2.40513  3.72494  3.29354  2.67741  2.69355  4.24690  2.90347  2.73739  3.18146  2.89801  2.37887  2.77519  2.98518  4.58477  3.61503
        0.01839  4.40100  5.12334  0.61958  0.77255  0.48576  0.95510
 336    2.75217  4.83642  3.19050  2.75967  4.34063  3.35309  3.90927  3.72145  2.10247  3.34367  4.18573  3.20948  1.39210  3.08316  2.95658  2.82532  2.96785  3.12699  5.57080  4.28466    359 p - - -
        2.68618  4.42225  2.77519  2.73123  3.46354  2.40513  3.72494  3.29354  2.67741  2.69355  4.24690  2.90347  2.73739  3.18146  2.89801  2.37887  2.77519  2.98518  4.58477  3.61503
        0.01839  4.40100  5.12334  0.61958  0.77255  0.48576  0.95510
 337    1.96780  4.54342  3.35178  2.80398  3.79199  3.52835  3.86101  3.16640  2.77456  2.84889  2.62871  3.24703  2.71676  2.97867  3.17687  2.22699  2.90149  2.80535  5.17831  3.91332    360 a - - -
        2.68618  4.42225  2.77519  2.73123  3.46354  2.40513  3.72494  3.29354  2.67741  2.69355  4.24690  2.90347  2.73739  3.18146  2.89801  2.37887  2.77519  2.98518  4.58477  3.61503
        0.01839  4.40100  5.12334  0.61958  0.77255  0.48576  0.95510
 338    3.27833  4.57419  5.10307  4.54240  3.44294  4.61313  5.01347  1.88993  4.41496  1.22217  2.88019  4.70405  4.85498  4.54222  4.50952  3.96239  3.36676  1.33810  5.38747  4.27591    361 l - - -
        2.68618  4.42225  2.77519  2.73123  3.46354  2.40513  3.72494  3.29354  2.67741  2.69355  4.24690  2.90347  2.73739  3.18146  2.89801  2.37887  2.77519  2.98518  4.58477  3.61503
        0.01839  4.40100  5.12334  0.61958  0.77255  0.48576  0.95510
 339    2.66295  4.38749  4.67584  4.10685  1.64533  4.18336  4.49785  2.31767  3.96511  1.72146  3.23731  4.24682  4.51164  4.12838  4.08020  3.50556  3.24047  1.71773  4.98296  3.75623    362 f - - -
        2.68618  4.42225  2.77519  2.73123  3.46354  2.40513  3.72494  3.29354  2.67741  2.69355  4.24690  2.90347  2.73739  3.18146  2.89801  2.37887  2.77519  2.98518  4.58477  3.61503
        0.01839  4.40100  5.12334  0.61958  0.77255  0.48576  0.95510
 340    2.11669  5.25481  2.26266  2.20481  4.59190  2.80271  3.73603  4.07220  2.54289  3.57350  4.34142  2.19334  3.90064  2.85479  3.05360  2.29093  3.03167  3.65029  5.72797  4.30896    363 a - - -
        2.68618  4.42225  2.77519  2.73123  3.46354  2.40513  3.72494  3.29354  2.67741  2.69355  4.24690  2.90347  2.73739  3.18146  2.89801  2.37887  2.77519  2.98518  4.58477  3.61503
        0.01839  4.40100  5.12334  0.61958  0.77255  0.48576  0.95510
 341    2.48818  2.12249  3.59415  3.15506  4.29252  1.98574  4.22051  3.70763  3.15828  3.37285  4.20136  2.39540  3.92121  3.46256  3.52633  1.80269  2.91188  3.26141  5.63848  4.39756    364 s - - -
        2.68618  4.42225  2.77519  2.73123  3.46354  2.40513  3.72494  3.29354  2.67741  2.69355  4.24690  2.90347  2.73739  3.18146  2.89801  2.37887  2.77519  2.98518  4.58477  3.61503
        0.01839  4.40100  5.12334  0.61958  0.77255  0.48576  0.95510
 342    2.99358  4.39763  4.54442  3.98709  3.55355  4.20445  4.61181  1.95245  3.87708  1.91928  3.44510  4.21205  4.54992  4.11770  4.07685  3.52544  1.84880  1.40307  5.25748  4.06701    365 v - - -
        2.68618  4.42225  2.77519  2.73123  3.46354  2.40513  3.72494  3.29354  2.67741  2.69355  4.24690  2.90347  2.73739  3.18146  2.89801  2.37887  2.77519  2.98518  4.58477  3.61503
        0.01839  4.40100  5.12334  0.61958  0.77255  0.48576  0.95510
 343    3.43987  5.51585  3.98178  3.15895  5.08703  3.99378  3.80748  4.37530  1.16211  3.73101  4.61525  3.50628  4.32542  2.93396  1.17647  3.41549  3.56031  4.06116  5.69693  4.60190    366 k - - -
        2.68618  4.42225  2.77519  2.73123  3.46354  2.40513  3.72494  3.29354  2.67741  2.69355  4.24690  2.90347  2.73739  3.18146  2.89801  2.37887  2.77519  2.98518  4.58477  3.61503
        0.01839  4.40100  5.12334  0.61958  0.77255  0.48576  0.95510
 344    2.62258  5.75748  1.07896  1.66428  5.06192  3.42382  3.96858  4.58176  2.99255  4.06816  4.91015  2.88094  4.04632  3.13003  3.60446  3.02478  3.43647  4.13724  6.22703  4.70231    367 d - - -
        2.68618  4.42225  2.77519  2.73123  3.46354  2.40513  3.72494  3.29354  2.67741  2.69355  4.24690  2.90347  2.73739  3.18146  2.89801  2.37887  2.77519  2.98518  4.58477  3.61503
        0.01839  4.40100  5.12334  0.61958  0.77255  0.48576  0.95510
 345    2.49599  4.65272  3.24035  2.67844  3.84161  3.55890  3.78062  2.95318  1.89236  2.75303  3.13064  3.16177  2.74398  2.99006  3.06738  2.77920  2.73043  2.79777  5.19987  3.91491    368 k - - -
        2.68618  4.42225  2.77519  2.73123  3.46354  2.40513  3.72494  3.29354  2.67741  2.69355  4.24690  2.90347  2.73739  3.18146  2.89801  2.37887  2.77519  2.98518  4.58477  3.61503
        0.01839  4.40100  5.12334  0.61958  0.77255  0.48576  0.95510
 346    2.79211  4.45969  3.74773  3.23192  3.69544  3.77900  4.16771  2.20401  3.17408  2.63373  3.62730  1.88142  4.20285  3.49781  3.50920  3.07732  2.95204  1.60735  5.22220  3.97977    369 v - - -
        2.68618  4.42225  2.77519  2.73123  3.46354  2.40513  3.72494  3.29354  2.67741  2.69355  4.24690  2.90347  2.73739  3.18146  2.89801  2.37887  2.77519  2.98518  4.58477  3.61503
        0.01839  4.40100  5.12334  0.61958  0.77255  0.48576  0.95510
 347    2.62783  4.31411  3.85667  3.36720  3.65984  2.66766  4.18813  1.54766  3.29922  2.70192  3.63056  3.62340  4.08085  3.58756  3.57812  2.78535  1.94331  2.58062  5.14598  3.93546    370 i - - -
        2.68618  4.42225  2.77519  2.73123  3.46354  2.40513  3.72494  3.29354  2.67741  2.69355  4.24690  2.90347  2.73739  3.18146  2.89801  2.37887  2.77519  2.98518  4.58477  3.61503
        0.01839  4.40100  5.12334  0.61958  0.77255  0.48576  0.95510
```

```
348   2.49711  4.50000  3.39553  2.84417  3.70894  3.44785  3.86781  3.08113  2.80965  2.77508  3.44588  3.27908  1.91884  3.13418  3.19964  2.56458  2.75178  2.72782  5.11407  2.87535    371 p - - -
      2.68618  4.42225  2.77519  2.73123  3.46354  2.40513  3.72494  3.29354  2.67741  2.69355  4.24690  2.90347  2.73739  3.18146  2.89801  2.37887  2.77519  2.98518  4.58477  3.61503
      0.01839  4.40100  5.12334  0.61958  0.77255  0.48576  0.95510
349   3.36766  4.61243  5.30475  4.83640  3.90708  4.85933  5.56108  1.39102  4.74747  2.36526  3.67200  5.00892  5.13568  5.00937  4.92392  4.28240  3.63587  0.78483  5.96234  4.74367    372 v - - -
      2.68618  4.42225  2.77519  2.73123  3.46354  2.40513  3.72494  3.29354  2.67741  2.69355  4.24690  2.90347  2.73739  3.18146  2.89801  2.37887  2.77519  2.98518  4.58477  3.61503
      0.01839  4.40100  5.12334  0.61958  0.77255  0.48576  0.95510
350   3.42108  5.50727  3.94039  3.13844  5.07043  3.97965  3.80724  4.36602  0.94756  3.72646  4.60827  3.49302  4.31554  2.93378  1.47540  3.39689  3.54654  4.04910  5.69642  4.59395    373 k - - -
      2.68618  4.42225  2.77519  2.73123  3.46354  2.40513  3.72494  3.29354  2.67741  2.69355  4.24690  2.90347  2.73739  3.18146  2.89801  2.37887  2.77519  2.98518  4.58477  3.61503
      0.01839  4.40100  5.12334  0.61958  0.77255  0.48576  0.95510
351   1.96973  4.30964  3.96559  3.44172  3.54693  3.64247  4.20013  2.71518  3.34760  1.39912  3.51309  3.69220  4.13181  3.62778  3.60812  2.53111  2.97756  2.34379  5.07744  3.87316    374 l - - -
      2.68618  4.42225  2.77519  2.73123  3.46354  2.40513  3.72494  3.29354  2.67741  2.69355  4.24690  2.90347  2.73739  3.18146  2.89801  2.37887  2.77519  2.98518  4.58477  3.61503
      0.01839  4.40100  5.12334  0.61958  0.77255  0.48576  0.95510
352   1.68354  4.85244  3.04442  2.54739  4.12396  3.34020  3.75408  3.54273  2.56773  3.15412  3.97558  2.43864  3.91173  2.66674  3.02128  2.53699  2.93569  3.21864  5.40148  3.31797    375 a - - -
      2.68618  4.42225  2.77519  2.73123  3.46354  2.40513  3.72494  3.29354  2.67741  2.69355  4.24690  2.90347  2.73739  3.18146  2.89801  2.37887  2.77519  2.98518  4.58477  3.61503
      0.01839  4.40100  5.12334  0.61958  0.77255  0.48576  0.95510
353   1.04121  3.28804  4.14173  3.76076  4.44773  3.15146  4.61151  3.81232  3.69484  3.55396  4.38874  3.70474  3.92442  3.94601  3.94028  1.46947  2.89812  3.28373  5.83780  4.67093    376 a - - -
      2.68618  4.42225  2.77519  2.73123  3.46354  2.40513  3.72494  3.29354  2.67741  2.69355  4.24690  2.90347  2.73739  3.18146  2.89801  2.37887  2.77519  2.98518  4.58477  3.61503
      0.01839  4.40100  5.12334  0.61958  0.77255  0.48576  0.95510
354   3.24755  5.82248  2.08591  0.75626  5.12609  3.45814  4.04366  4.66914  3.09092  4.16007  5.03451  2.93043  4.10189  3.21866  3.69503  3.11792  3.54655  4.23265  6.28315  4.77779    377 e - - -
      2.68618  4.42225  2.77519  2.73123  3.46354  2.40513  3.72494  3.29354  2.67741  2.69355  4.24690  2.90347  2.73739  3.18146  2.89801  2.37887  2.77519  2.98518  4.58477  3.61503
      0.01839  4.40100  5.12334  0.61958  0.77255  0.48576  0.95510
355   2.08645  4.87162  3.08725  2.52569  4.11112  3.50957  3.02798  3.01996  2.09780  2.89365  3.94294  3.04180  3.89804  2.59540  2.71712  2.71578  2.83460  3.20449  5.37025  3.78384    378 a - - -
      2.68618  4.42225  2.77519  2.73123  3.46354  2.40513  3.72494  3.29354  2.67741  2.69355  4.24690  2.90347  2.73739  3.18146  2.89801  2.37887  2.77519  2.98518  4.58477  3.61503
      0.01839  4.40100  5.12334  0.61958  0.77255  0.48576  0.95510
356   0.94786  3.19394  4.13275  3.69078  4.11417  3.25241  4.48457  3.36986  3.60808  3.18908  4.06982  3.70803  3.96792  3.86107  3.85291  2.55109  2.28740  2.76970  5.55622  4.37677    379 a - - -
      2.68618  4.42225  2.77519  2.73123  3.46354  2.40513  3.72494  3.29354  2.67741  2.69355  4.24690  2.90347  2.73739  3.18146  2.89801  2.37887  2.77519  2.98518  4.58477  3.61503
      0.01839  4.40100  5.12334  0.61958  0.77255  0.48576  0.95510
357   2.56820  4.23044  4.54261  3.95705  1.98981  3.98015  4.20507  2.08597  3.79324  1.69806  3.20549  4.04256  4.32397  3.94384  3.87714  3.28713  3.06539  2.38509  4.67972  2.49904    380 l - - -
      2.68618  4.42225  2.77519  2.73123  3.46354  2.40513  3.72494  3.29354  2.67741  2.69355  4.24690  2.90347  2.73739  3.18146  2.89801  2.37887  2.77519  2.98518  4.58477  3.61503
      0.01839  4.40100  5.12334  0.61958  0.77255  0.48576  0.95510
358   3.12225  3.56510  4.98797  4.42371  3.47616  4.43119  4.84188  1.84995  4.28454  1.33389  3.20572  4.54089  4.72248  4.44093  4.37489  3.77242  3.36119  1.34563  5.28621  4.13162    381 l - - -
      2.68618  4.42225  2.77519  2.73123  3.46354  2.40513  3.72494  3.29354  2.67741  2.69355  4.24690  2.90347  2.73739  3.18146  2.89801  2.37887  2.77519  2.98518  4.58477  3.61503
      0.01839  4.40100  5.12334  0.61958  0.77255  0.48576  0.95510
359   1.93921  5.07752  2.75810  2.35273  4.37633  3.29756  3.16136  3.83137  2.43145  3.36405  4.13473  2.56469  3.86248  2.70750  2.91687  2.26921  2.77724  3.44391  5.54136  3.95848    382 a - - -
      2.68618  4.42225  2.77519  2.73123  3.46354  2.40513  3.72494  3.29354  2.67741  2.69355  4.24690  2.90347  2.73739  3.18146  2.89801  2.37887  2.77519  2.98518  4.58477  3.61503
      0.01839  4.40100  5.12334  0.61958  0.77255  0.48576  0.95510
360   2.49364  4.42506  4.81331  4.23705  3.36566  4.31796  4.67992  1.87492  4.10084  1.25499  2.75965  4.38878  4.61487  4.25599  4.20921  3.64264  3.30747  1.77506  5.15747  4.01709    383 l - - -
      2.68618  4.42225  2.77519  2.73123  3.46354  2.40513  3.72494  3.29354  2.67741  2.69355  4.24690  2.90347  2.73739  3.18146  2.89801  2.37887  2.77519  2.98518  4.58477  3.61503
      0.01839  4.40100  5.12334  0.61958  0.77255  0.48576  0.95510
361   3.70547  4.97161  5.24178  4.77253  1.29172  4.83223  4.59833  2.60907  4.60988  1.02833  3.17414  4.81011  5.01711  4.58762  4.62534  4.22115  3.92481  2.78901  4.73279  3.23287    384 l - - -
      2.68618  4.42225  2.77519  2.73123  3.46354  2.40513  3.72494  3.29354  2.67741  2.69355  4.24690  2.90347  2.73739  3.18146  2.89801  2.37887  2.77519  2.98518  4.58477  3.61503
      0.01839  4.40100  5.12334  0.61958  0.77255  0.48576  0.95510
362   2.62780  5.17221  2.88764  2.35870  4.51168  3.32477  3.04287  3.98442  1.95672  3.46887  4.21724  2.75760  3.85914  2.29844  2.40929  2.34357  2.92687  3.56047  5.60218  4.20786    385 k - - -
      2.68618  4.42225  2.77519  2.73123  3.46354  2.40513  3.72494  3.29354  2.67741  2.69355  4.24690  2.90347  2.73739  3.18146  2.89801  2.37887  2.77519  2.98518  4.58477  3.61503
      0.01839  4.40100  5.12334  0.61958  0.77255  0.48576  0.95510
363   3.47635  4.75757  5.25542  4.68721  3.31505  4.80523  5.15490  2.14200  4.55596  0.86608  2.64448  4.88893  4.97263  4.60936  4.62297  4.16300  3.69896  1.69089  5.40122  4.35864    386 l - - -
      2.68618  4.42225  2.77519  2.73123  3.46354  2.40513  3.72494  3.29354  2.67741  2.69355  4.24690  2.90347  2.73739  3.18146  2.89801  2.37887  2.77519  2.98518  4.58477  3.61503
      0.01839  4.40100  5.12334  0.61958  0.77255  0.48576  0.95510
364   2.45907  4.51011  3.48329  2.91210  3.69830  3.63630  3.88910  2.80266  2.71112  2.73538  3.63114  3.33871  4.02929  3.16216  2.00870  2.88841  2.69186  1.90925  5.10993  3.86501    387 v - - -
      2.68622  4.42229  2.77520  2.73091  3.46358  2.40506  3.72499  3.29358  2.67731  2.69359  4.24694  2.90351  2.73744  3.18151  2.89805  2.37891  2.77524  2.98523  4.58481  3.61507
      0.66352  1.77074  1.15596  0.37141  1.17041  0.48576  0.95510
365   2.89478  5.44102  1.53313  1.64364  4.74577  3.24711  3.74852  4.25311  2.72387  3.75435  4.58359  2.71283  3.84236  2.90517  3.30205  2.41809  3.17569  3.82590  5.91815  4.42836    390 d - - -
      2.68618  4.42225  2.77519  2.73123  3.46354  2.40513  3.72494  3.29354  2.67741  2.69355  4.24690  2.90347  2.73739  3.18146  2.89801  2.37887  2.77519  2.98518  4.58477  3.61503
      0.02672  4.03167  4.75402  0.61958  0.77255  0.30147  1.34603
366   2.61996  4.76104  2.35447  1.84060  4.48064  3.44803  3.64551  3.95580  2.39131  3.45066  4.19898  2.79057  3.60910  2.32838  2.83563  2.56931  2.82412  3.24483  5.59526  4.19281    391 e - - -
      2.68618  4.42225  2.77519  2.73123  3.46354  2.40513  3.72494  3.29354  2.67741  2.69355  4.24690  2.90347  2.73739  3.18146  2.89801  2.37887  2.77519  2.98518  4.58477  3.61503
      0.01839  4.40100  5.12334  0.61958  0.77255  0.48576  0.95510
367   3.00045  5.58616  1.93056  1.69235  4.89729  2.04302  3.86481  4.41323  2.78700  3.88554  4.68256  2.12566  3.98518  3.00375  3.35408  2.76119  3.26713  3.96756  6.03081  4.54606    392 e - - -
      2.68618  4.42225  2.77519  2.73123  3.46354  2.40513  3.72494  3.29354  2.67741  2.69355  4.24690  2.90347  2.73739  3.18146  2.89801  2.37887  2.77519  2.98518  4.58477  3.61503
      0.01839  4.40100  5.12334  0.61958  0.77255  0.48576  0.95510
368   2.63815  4.99556  2.44830  2.04795  4.27313  3.05579  3.67384  3.71212  2.44593  3.14982  3.10135  2.97505  3.86695  2.79971  2.92377  2.29128  2.73262  3.04356  5.47484  4.10980    393 e - - -
      2.68618  4.42225  2.77519  2.73123  3.46354  2.40513  3.72494  3.29354  2.67741  2.69355  4.24690  2.90347  2.73739  3.18146  2.89801  2.37887  2.77519  2.98518  4.58477  3.61503
      0.01839  4.40100  5.12334  0.61958  0.77255  0.48576  0.95510
369   2.41433  5.15399  2.76206  1.96786  4.48357  3.44902  3.43221  3.95903  2.06946  3.45222  4.19978  2.86428  3.84688  2.47180  2.87577  2.46972  2.54389  3.40148  5.59522  4.19301    394 e - - -
      2.68618  4.42225  2.77519  2.73123  3.46354  2.40513  3.72494  3.29354  2.67741  2.69355  4.24690  2.90347  2.73739  3.18146  2.89801  2.37887  2.77519  2.98518  4.58477  3.61503
      0.01839  4.40100  5.12334  0.61958  0.77255  0.48576  0.95510
370   2.47518  4.19348  3.96377  3.17009  3.07890  3.52174  4.03640  2.33693  3.16095  2.25117  3.21549  3.65696  4.11530  3.53694  3.52442  3.01976  2.27256  1.74707  4.80825  3.60750    395 v - - -
      2.68618  4.42225  2.77519  2.73123  3.46354  2.40513  3.72494  3.29354  2.67741  2.69355  4.24690  2.90347  2.73739  3.18146  2.89801  2.37887  2.77519  2.98518  4.58477  3.61503
      0.01839  4.40100  5.12334  0.61958  0.77255  0.48576  0.95510
371   2.52054  4.13066  4.30149  3.71102  1.84682  3.81856  3.51305  2.44203  3.56510  2.01367  2.87344  3.85131  4.18221  3.75494  3.69331  3.11416  2.92692  2.29067  4.68122  2.85638    396 f - - -
      2.68618  4.42225  2.77519  2.73123  3.46354  2.40513  3.72494  3.29354  2.67741  2.69355  4.24690  2.90347  2.73739  3.18146  2.89801  2.37887  2.77519  2.98518  4.58477  3.61503
      0.01839  4.40100  5.12334  0.61958  0.77255  0.48576  0.95510
372   2.81787  5.44914  1.90539  2.04864  4.76684  3.43520  3.78762  4.26925  2.63609  3.74330  4.51737  2.13458  3.94028  1.93463  3.16938  2.64618  3.14511  3.82892  5.88222  4.42794    397 d - - -
      2.68618  4.42225  2.77519  2.73123  3.46354  2.40513  3.72494  3.29354  2.67741  2.69355  4.24690  2.90347  2.73739  3.18146  2.89801  2.37887  2.77519  2.98518  4.58477  3.61503
      0.01839  4.40100  5.12334  0.61958  0.77255  0.48576  0.95510
373   2.69134  5.14964  2.94732  2.07563  4.48047  3.46346  3.64651  3.94951  1.84176  3.44411  4.19634  2.69177  3.85752  2.52845  2.66843  2.30165  2.84431  3.43888  5.58630  3.78140    398 k - - -
```

```
          2.68618  4.42225  2.77519  2.73123  3.46354  2.40513  3.72494  3.29354  2.67741  2.69355  4.24690  2.90347  2.73739  3.18146  2.89801  2.37887  2.77519  2.98518  4.58477  3.61503
          0.02842  4.40100  4.15064  0.61958  0.77255  0.48576  0.95510
    374   2.68758  3.06514  4.49881  3.89855  2.80482  3.83734  4.12221  2.36144  3.71429  2.18023  3.04386  3.94343  4.19956  3.86613  3.77094  3.14341  2.91940  2.10608  2.78722  2.14087    399 v - - -
          2.68618  4.42225  2.77519  2.73123  3.46354  2.40513  3.72494  3.29354  2.67741  2.69355  4.24690  2.90347  2.73739  3.18146  2.89801  2.37887  2.77519  2.98518  4.58477  3.61503
          0.01857  4.39115  5.11350  0.61958  0.77255  0.50083  0.93148
    375   2.67740  3.46757  4.45202  3.85041  2.41099  3.82949  4.15508  2.09632  3.67684  1.97179  2.32326  3.92718  4.19212  3.84237  3.75272  2.69018  2.75203  2.25586  4.70516  3.52582    400 l - - -
          2.68618  4.42225  2.77519  2.73123  3.46354  2.40513  3.72494  3.29354  2.67741  2.69355  4.24690  2.90347  2.73739  3.18146  2.89801  2.37887  2.77519  2.98518  4.58477  3.61503
          0.01857  4.39115  5.11350  0.61958  0.77255  0.50083  0.93148
    376   2.00810  5.12869  2.85360  2.14999  4.44511  3.45238  3.65993  3.91080  2.18182  3.42219  4.18239  2.69567  3.85774  2.53686  2.89886  2.37306  2.64806  3.50494  5.58075  4.18701    401 a - - -
          2.68618  4.42225  2.77519  2.73123  3.46354  2.40513  3.72494  3.29354  2.67741  2.69355  4.24690  2.90347  2.73739  3.18146  2.89801  2.37887  2.77519  2.98518  4.58477  3.61503
          0.01857  4.39115  5.11350  0.61958  0.77255  0.50083  0.93148
    377   2.65843  4.45888  3.42159  2.85727  3.36506  2.42004  3.84957  2.41947  2.69084  2.51571  3.55940  3.07279  3.98830  3.13685  3.04316  2.59355  2.49225  2.59358  5.03139  3.78209    402 i - - -
          2.68618  4.42225  2.77519  2.73123  3.46354  2.40513  3.72494  3.29354  2.67741  2.69355  4.24690  2.90347  2.73739  3.18146  2.89801  2.37887  2.77519  2.98518  4.58477  3.61503
          0.19460  4.39115  1.80517  0.61958  0.77255  0.50083  0.93148
    378   2.53221  4.27927  3.71778  3.34133  3.36618  3.75993  4.10052  2.33022  3.25000  1.35986  3.31412  3.65379  3.73254  3.52960  3.51848  3.05153  2.96025  2.25745  4.93819  3.73632    403 l - - -
          2.68618  4.42225  2.77519  2.73123  3.46354  2.40513  3.72494  3.29354  2.67741  2.69355  4.24690  2.90347  2.73739  3.18146  2.89801  2.37887  2.77519  2.98518  4.58477  3.61503
          0.02211  4.21866  4.94101  0.61958  0.77255  0.48686  0.95335
    379   2.59261  5.20324  2.05933  1.91102  4.52690  3.42336  3.66622  3.85010  2.36584  3.50009  4.25665  2.89325  3.04869  2.66269  2.94103  2.38138  2.95496  3.58368  5.64636  4.23299    404 e - - -
          2.68618  4.42225  2.77519  2.73123  3.46354  2.40513  3.72494  3.29354  2.67741  2.69355  4.24690  2.90347  2.73739  3.18146  2.89801  2.37887  2.77519  2.98518  4.58477  3.61503
          0.01329  4.32774        *  0.61958  0.77255  0.00000        *
//
```
